# DOT1L promotes angiogenesis through cooperative regulation of VEGFR2 with ETS-1

**DOI:** 10.18632/oncotarget.11939

**Published:** 2016-09-10

**Authors:** Yang Duan, Xue Wu, Qiang Zhao, Jie Gao, Dawei Huo, Xinhua Liu, Zheng Ye, Xu Dong, Zheng Fu, Yongfeng Shang, Chenghao Xuan

**Affiliations:** ^1^ Tianjin Key Laboratory of Medical Epigenetics, Department of Biochemistry and Molecular Biology, Tianjin Medical University, Tianjin 300070, China; ^2^ Geneseeq Technology Inc., Toronto, M5G1L7, Canada; ^3^ College of Life Sciences, Nankai University, Tianjin 300071, China; ^4^ Department of Immunology, Tianjin Medical University, Tianjin 300070, China

**Keywords:** DOT1L, angiogenesis, VEGFR2, ETS-1, HUVEC

## Abstract

Histone methyltransferase DOT1L is implicated in various biological processes including cell proliferation, differentiation and embryogenesis. Gene ablation of *Dot1l* results in embryonic lethality and cardiovascular defects including decreased vasculature. However, how DOT1L might contribute to the development of vasculature is not clear. Here, we report that DOT1L is required for angiogenesis. We demonstrated that silencing of DOT1L in human umbilical vein endothelial cells (HUVECs) leads to decreased cell viability, migration, tube formation, and capillary sprout formation *in vitro*, as well as reduced formation of functional vascular networks in matrigel plugs *in vivo*. Genome-wide analysis of DOT1L targets via H3K79me2 ChIP-seq annotation in HUVECs identified a number of genes including *VEGFR2* that are critically involved in angiogenesis. We showed that DOT1L cooperates with transcription factor ETS-1 to stimulate the expression of VEGFR2, thereby activating ERK1/2 and AKT signaling pathways and promoting angiogenesis. Our study revealed a mechanistic role for DOT1L in the promotion of angiogenesis, adding to the understanding of the biological function of this histone methyltransferase.

## INTRODUCTION

The circulatory system consists of a highly organized network of blood vessels which deliver nutrients, gases, and hormones throughout the body. The formation of blood vessels requires the coordination of two distinct cellular processes. During embryogenesis, proliferating endothelial precursor cells migrate and differentiate to form a primitive lumenized vascular plexus, a process known as vasculogenesis. Through angiogenesis, the vascular plexus significantly sprouts due to capillary branching and is transformed into the highly organized vascular net [1].

In addition to modulating various physiological processes, such as embryonic development and injury response, angiogenesis is also involved in pathophysiological conditions, including diabetic retinopathy and tumor growth [2]. Aberrant activation of angiogenesis contributes to tumor development; without blood supply the volume of a tumor nodule cannot exceed 2–3 mm^3^ due to hypoxia that results in the death of tumor cells [3]. Strategies targeting angiogenesis have proven to be valuable therapeutic approaches in preventing tumor progression [4], therefore an insightful understanding of the molecular mechanisms of angiogenesis has important implications.

The establishment and remodeling of the vascular system depends on a precisely orchestrated interplay between attractants and repellants. During past two decades, extensive studies have identified several secreted signaling molecules, including vascular endothelial growth factor (VEGF), fibroblast growth factor (FGF), and platelet-derived growth factor (PDGF), which transduce signal through their corresponding receptor to modulate endothelial cell behavior. As the most potent angiogenic signaling factor, VEGF functions by binding to one of three cognate receptor tyrosine kinases (VEGFR1-3) [5, 6]. Among them, VEGFR2 which has strong tyrosine kinase activity is thought to dominate the angiogenic response [7, 8]. The particularly important role of VEGFR2 in VEGF-mediated signaling had been demonstrated via gene-targeting approaches in mice, since *VEGFR2*-null mice die at embryonic day 8.5–9 due to defective blood-island formation and near-absence of vasculature [9]. Stimulation of VEGFR2 with VEGF results in VEGFR2 autophosphorylation and activation of several well-studied signal transduction pathways that regulate endothelial cell proliferation, migration, and tube formation.

Dot1 (disruptor of telomeric silencing) was initially discovered in budding yeast for its essential role in the regulation of telomeric silencing [10]. Dot1 and its mammalian homolog, DOT1L (DOT1-like), possess histone methyltranferase activity toward histone H3K79 [11]. Great progress has been made in characterizing the role of DOT1L-mediated H3K79 methylation in transcriptional regulation, cell cycle regulation, the DNA damage response, the development of leukemia, and so on [12]. Biologically, germline knockout of mDOT1L results in lethality by embryonic day 10.5 (E10.5) during organogenesis of the cardiovascular system [13]. Consistently, in *Dot1L*^1lox/1lox^ embryos, the yolk sac vasculature was present but was frequently underdeveloped and disorganized [13], suggesting a critical role for DOT1L in angiogenesis. However, the exact mechanism by which DOT1L is implicated in angiogenesis is still unclear.

In this study, we found that DOT1L promotes angiogenesis *in vitro* and *in vivo*. We showed that silencing of DOT1L in HUVECs results in decreased cell viability, migration, tube formation, sprouting *in vitro*, and reduced vascularization in matrigel plugs *in vivo*. Moreover, we demonstrated that DOT1L together with ETS-1 activates the transcription of VEGFR2, a key factor controlling angiogenesis.

## RESULTS

### Silencing of DOT1L leads to defective angiogenesis *in vitro*

**Figure 1:**
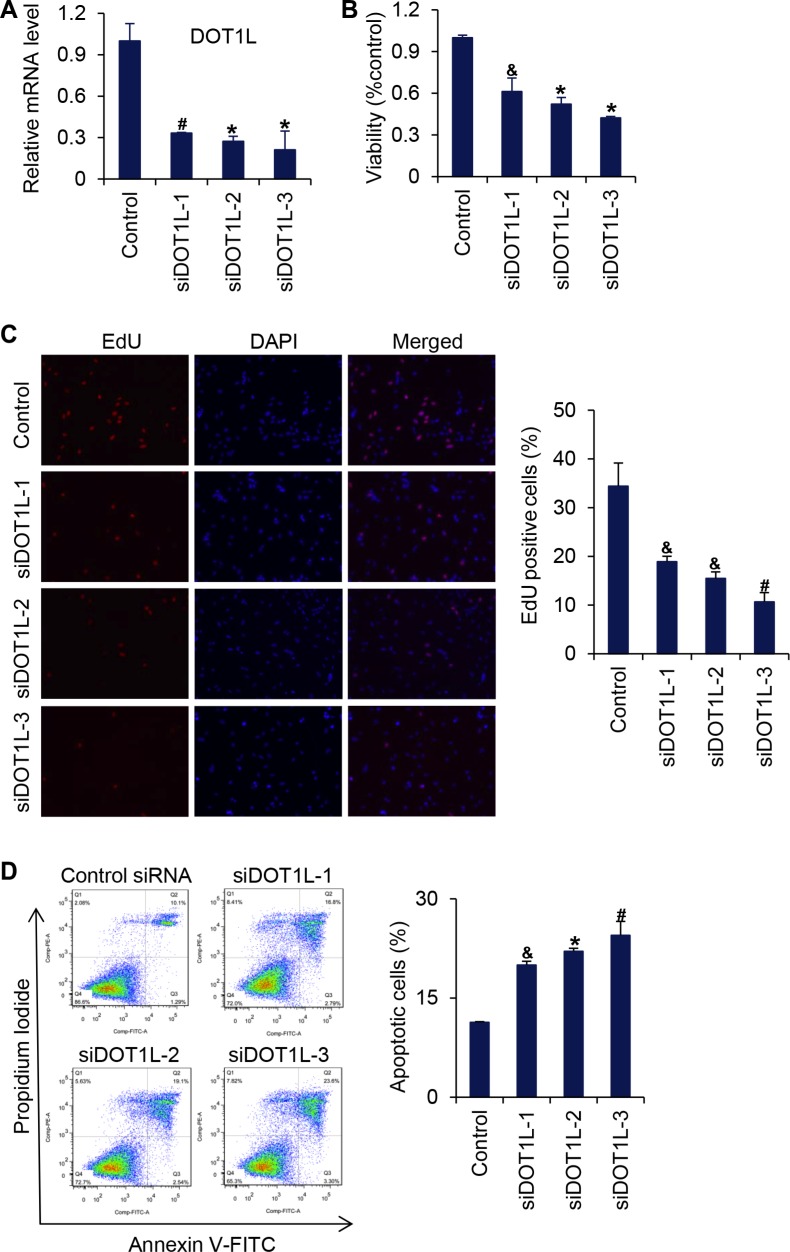
Knockdown of DOT1L inhibits cell viability of endothelial cells (**A**) Effects of three siRNAs against DOT1L in HUVECs on DOT1L mRNA expression as measured by quantitative real-time RT-PCR. Data are mean ± SD for *n* = 3; *P < 0.001, ^#^*P* < 0.01 vs. control (Student's *t* test). (**B**) Cell viability was measured using MTT assay. Data are mean ± SD for *n* = 3; *P < 0.001, ^&^*P* < 0.05 vs. control (Student's *t* test). (**C**) EdU incorporation assay. Data are mean ± SD for *n* = 3; ^#^*P* < 0.01, ^&^*P* < 0.05 vs. control (Student's *t* test). (**D**) HUVECs transfected with indicated siRNAs were stained with FITC-labeled annexin V and propidium iodide, followed by apoptotic analysis using flow cytometry. Data are mean ± SD for *n* = 3; *P < 0.001, ^#^*P* < 0.01, ^&^*P* < 0.05 vs. control (Student's *t* test).

As stated above, in *Dot1L*^1lox/1lox^ embryos, the yolk sac vasculature was underdeveloped and disorganized [13], suggesting that loss-of-function of DOT1L is associated with defective angiogenesis. Angiogenesis begins from local destruction of the wall of preexisting blood vessel, activation of endothelial cell (EC) proliferation, and migration toward stimuli in the microenvironment. Endothelial cells are assembled in tubular structures around which blood vessel walls are then formed [14–19]. To investigate whether or not DOT1L regulates angiogenesis by controlling the behavior of endothelial cells, we examined the effect of loss-of-function of DOT1L in HUVECs on the cell viability, migration, tube formation, and capillary sprout formation. For this purpose, three different siRNA oligonucleotides targeting DOT1L were transfected into HUVECs, and the expression of DOT1L was analyzed by real-time RT-PCR assays seventy-two hours after transfection. The results showed that these three siRNAs all caused a significant suppression of DOT1L in HUVECs (Figure 1A). MTT assays were then carried out to assess the viability of DOT1L silencing endothelial cells. As shown in Figure 1B, compared with control, knockdown of DOT1L resulted in a remarkable decrease in the viability of HUVECs. In addition, to investigate the effect of DOT1L knockdown on cell proliferation, EdU incorporation assays were performed. The results showed that DOT1L silencing resulted in a significant decrease in cell proliferation of HUVECs (Figure 1C). Furthermore, apoptotic analysis using FITC-labeled annexin V and propidium iodide showed that knockdown of DOT1L promoted apoptosis of HUVECs (Figure 1D).

**Figure 2:**
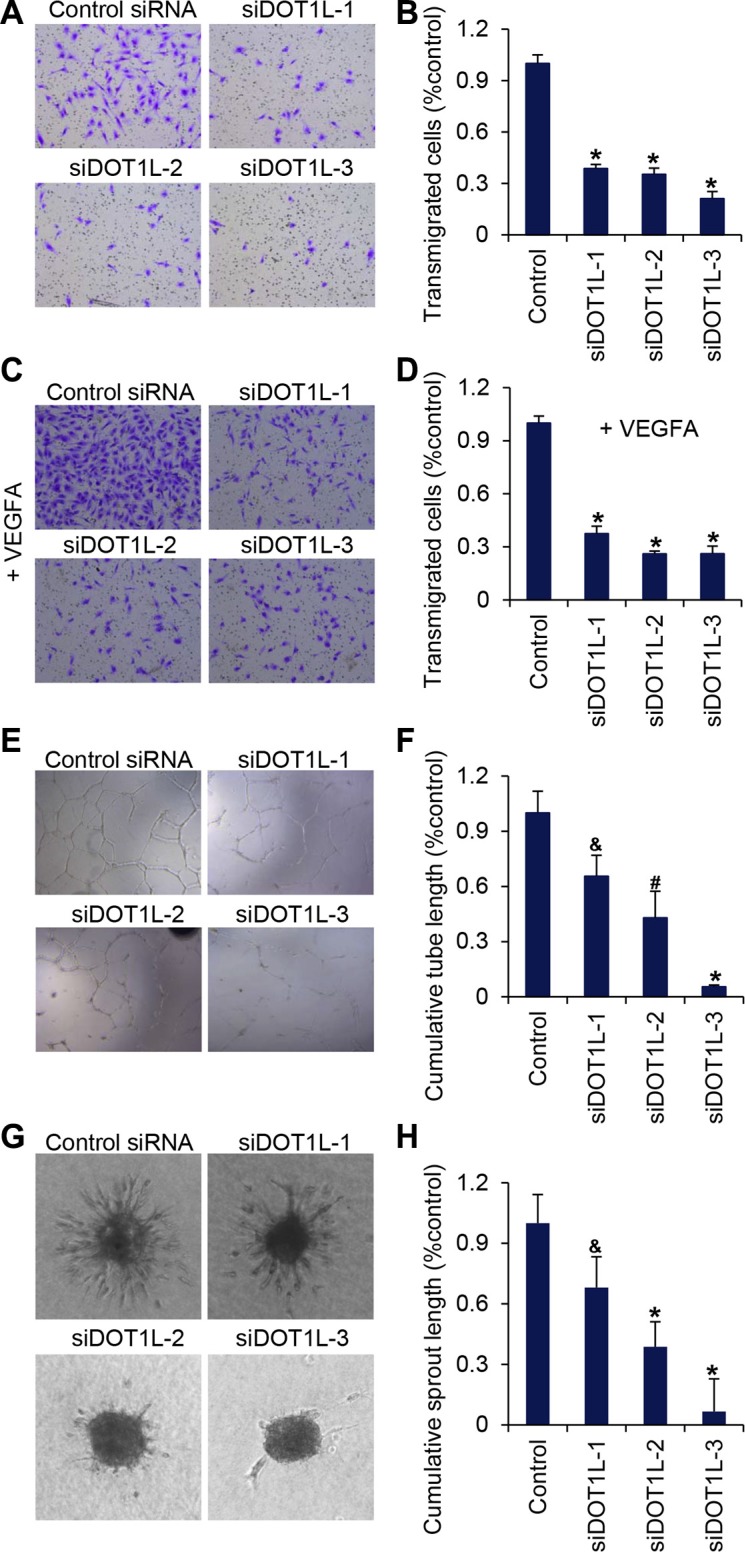
DOT1L silencing suppresses migration of HUVECs, tube formation and angiogenic sprouting DOT1L was silenced in HUVECs by three different siRNAs (DOT1L-1, −2, and −3) and the functional activity of the cells was compared to cells transfected with control siRNAs. (**A**) Transmigration of HUVECs was determined using transwell assays. The images represent one field under microscopy in control and DOT1L-depleted groups, respectively. (**B**) The transmigrated cells were counted. Data are mean ± SD for *n* = 5; *P < 0.001 vs. control (Student's *t* test). (**C**) Migration of HUVECs towards VEGFA was determined using transwell assays. (**D**) The transmigrated cells were counted. Data are mean ± SD for *n* = 5; *P < 0.001 vs. control (Student's *t* test). (**E**) The matrigel network assay was detected *in vitro*. (**F**) Endothelial tube formation capacity is shown as total tube length per field under microscopy. Data are mean ± SD for *n* = 4; *P < 0.001, ^#^*P* < 0.01, ^&^*P* < 0.05 vs. control (Student's *t* test). (**G**) A spheroid assay was performed to analyze basal endothelial sprouting capacity. Representative spheroids are shown. (**H**) Endothelial sprouting capacity is given as cumulative sprout length per spheroid. Data are mean ± SD for *n* = 6; *P < 0.001, ^&^*P* < 0.05 vs. control (Student's *t* test).

Then the migration ability of HUVECs was measured using transwell assays. The same amount of HUVECs transfected with different DOT1L siRNAs or scrambled siRNAs were placed in the upper chamber coated with matrigel, and the transmigrated cells were counted twenty-four hours later. The results showed that DOT1L deficiency led to a significant decrease in HUVECs migration (Figure 2A and 2B). To simulate stimuli in the microenvironment around endothelial cells *in vivo*, VEGFA was added into the EGM in the lower well. We showed that the migration of HUVECs towards VEGFA was inhibited by DOT1L knockdown by approximately 70% compared with HUVECs transfected with scrambled siRNAs (Figure 2C and 2D).

Next, forty-eight hours after the transfection of DOT1L siRNAs or scrambled siRNAs, the same amount of HUVECs were cultured on matrigel for twenty-four hours. The morphological changes of HUVECs were observed by microscopy and total tube lengths were analyzed. Typical capillary-like structure formed by HUVECs was evident in control cells (Figure 2E). However, the formation of capillary-like structure was inhibited after the expression of DOT1L was silenced (Figure 2E and 2F).

The effect of DOT1L silencing on the angiogenic potential of endothelial cells was further examined by measuring capillary sprouting in a 3-dimensional collagen-embedded spheroid culture assay. HUVECs with DOT1L knockdown or not were generated in hanging drops and suspended in EGM containing rat tail collagen I and methylcellulose. The morphogenesis of HUVECs-containing spheroids was observed twenty-four hours later using a microscope, and the cumulative sprout length of each spheroid was quantified. As shown in Figure 2G and 2H, the suppression of DOT1L expression profoundly decreased sprout length, the number of branch points, and the number of sprouts per spheroid.

### DOT1L knockdown inhibits blood vessel formation *in vivo*

**Figure 3:**
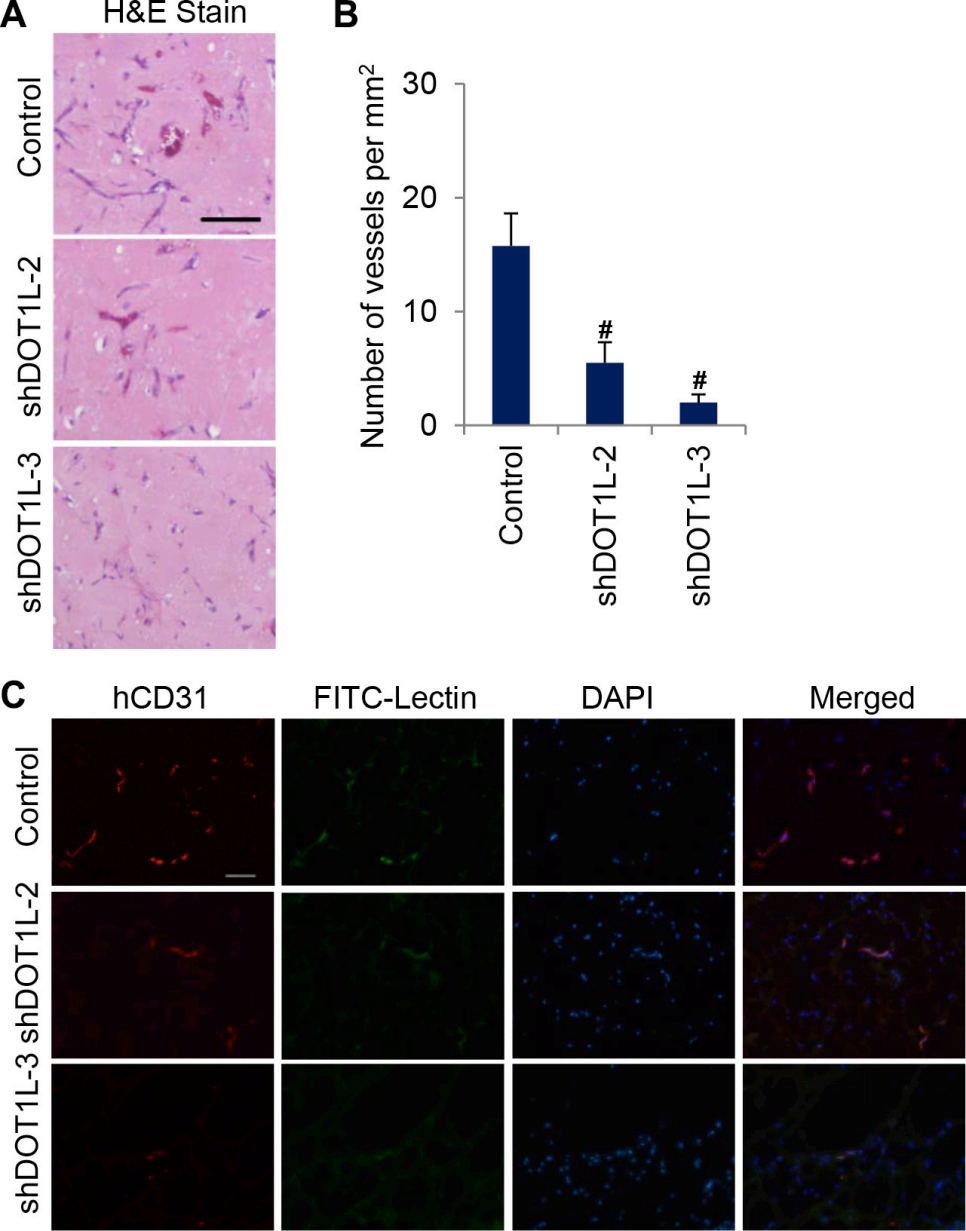
Depletion of DOT1L inhibits angiogenesis *in vivo* Matrigel plugs containing HUVECs expressing DOT1L shRNA2, shRNA3 or control shRNA, were subcutaneously implanted in SCID mice *in vivo*. Eight days later, hematoxylin and eosin staining was performed, and matrigel plugs were stained with anti-hCD31 and FITC-Lectin to analyze vascularization. (**A**) Representative hematoxylin and eosin–stained plug sections. Scale bar, 55 μm. (**B**) Quantification of microvessel density determined by the number of vessels per square milimeter. Data are mean ± SD for *n* = 4; ^#^*P* < 0.01 vs. control (Student's *t* test). (**C**) Fluorescent confocal microscopic images of frozen sections of matrigel plugs. Red, hCD31. Green, FITC-Lectin. Blue, DAPI. Scale bar, 100 μm.

To further support the angiogenic role of DOT1L, we used an *in vivo* spheroid assay in mice [20]. To this end, spheroids of HUVECs stably expressing DOT1L shRNAs or control shRNAs were mixed with VEGFA-containing matrigel, and subcutaneously implanted in CB17 SCID mice. Eight days later, matrigel plugs were removed and analyzed. The hematoxylin and eosin staining of matrigel sections showed that knockdown of DOT1L reduced the overall vascular density in matrigel (Figure 3A and 3B). Furthermore, to analyze human EC–derived neovasculature, the matrigel plugs were stained with anti-hCD31 and FITC-Lectin. As observed under immunofluorescence microscopy, human EC–derived neovasculature was significantly inhibited in response to the knockdown of DOT1L (Figure 3C). Taken together, these data support a notion that DOT1L is required for efficient angiogenesis *in vitro* and *in vivo*.

### Identification of the angiogenic targets of DOT1L in HUVECs

**Figure 4:**
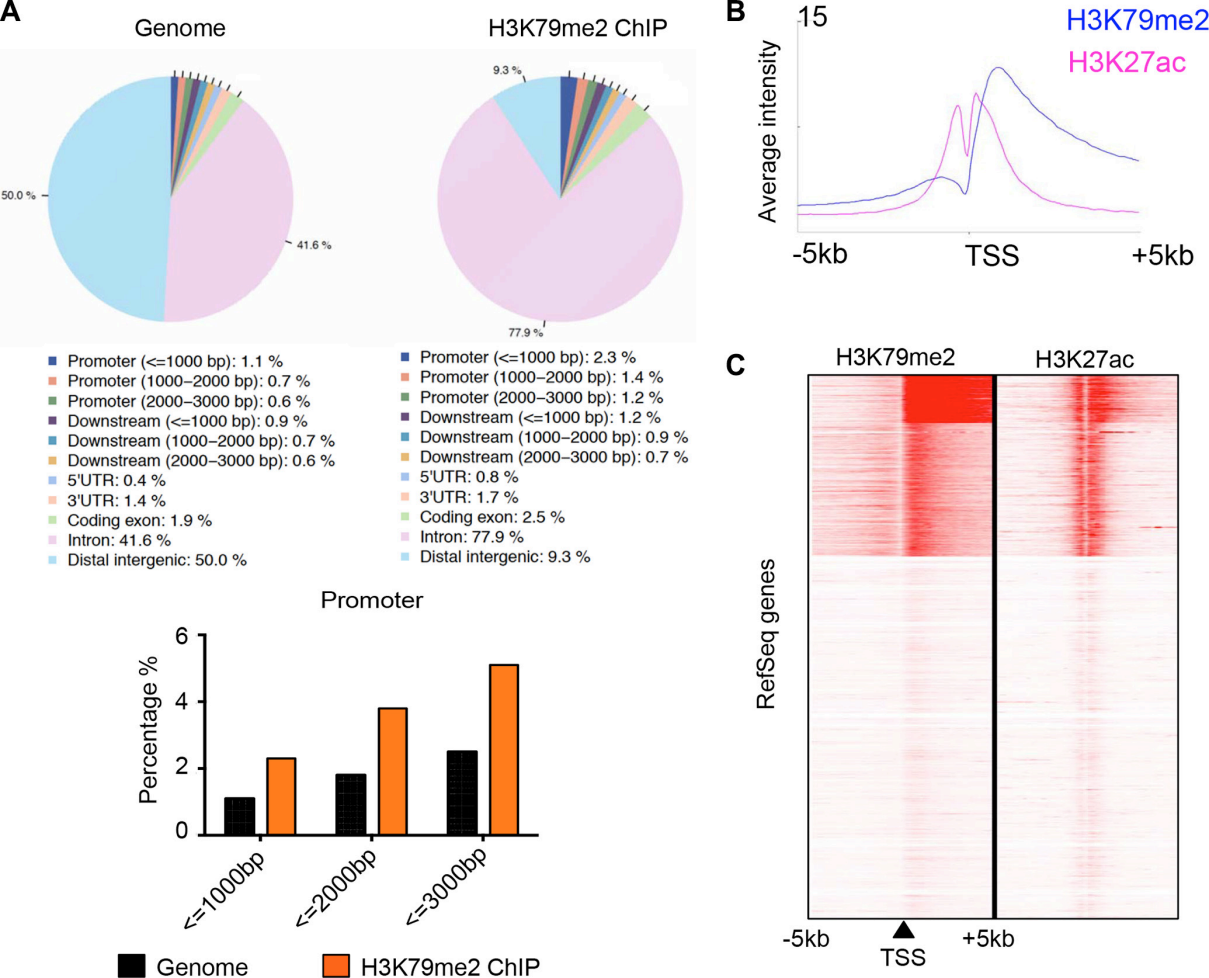
H3K79me2 was enriched in the downstream of active promoters in HUVECs (**A**) Genomic annotation of H3K79me2 profile in HUVECs. HUVEC H3K79me2 ChIP-seq peaks was analyzed by CEAS. (**B**) ChIP-seq profiling of H3K79me2 and H3K27ac in HUVECs over a 5-kb window around TSSs of all RefSeq genes by seqMINER. (**C**) ChIP-seq density heatmaps of H3K79me2 and H3K27ac.

Dot1 and DOT1L appear to be solely responsible for catalyzing mono-, di-, and trimethylation of H3K79 in a nonprocessive manner [21, 22], as knockout of Dot1 in yeast, flies, and mice results in complete loss of H3K79 methylation [10, 13, 23]. To gain a mechanistic insight into the essential role of DOT1L in angiogenesis, we investigated the cellular targets of DOT1L in HUVECs responsible for its angiogenic function through analyzing the distribution of H3K79me2 across the genome. The HUVEC_H3K79me2 ChIP-seq data was downloaded from the ENCODE Project (Broad/MGH ENCODE group; GSM1003555) [24]. ChIP-seq peaks were analyzed by CEAS (Cis-regulation element annotation system) to obtain the genomic annotation of H3K79me2 profile. Similar to the distribution pattern in 3T3-L1 cells [11], our analysis showed that in HUVECs, H3K79me2 is highly enriched in the gene bodies (Figure 4A), especially downstream of the promoter region, which peaks at approximately 1.2 kb downstream of TSSs (Figure 4B). A series of studies have revealed that the methylation of H3K79, especially H3K79me2 and H3K79me3, is a marker of active transcription [11, 25, 26]. In order to investigate the relationship between H3K79me2 and transcription status in HUVECs, ChIP-seq profiling of H3K79me2 over a 5-kb window around TSSs of all RefSeq genes was compared with that of H3K27ac (Broad/MGH ENCODE group; GSM733691) [27], which is a definite active transcription mark. The analysis showed a high correlation between these two histone modifications (Figure 4B and 4C), further indicating that H3K79me2 marks active transcribed genes.

**Figure 5:**
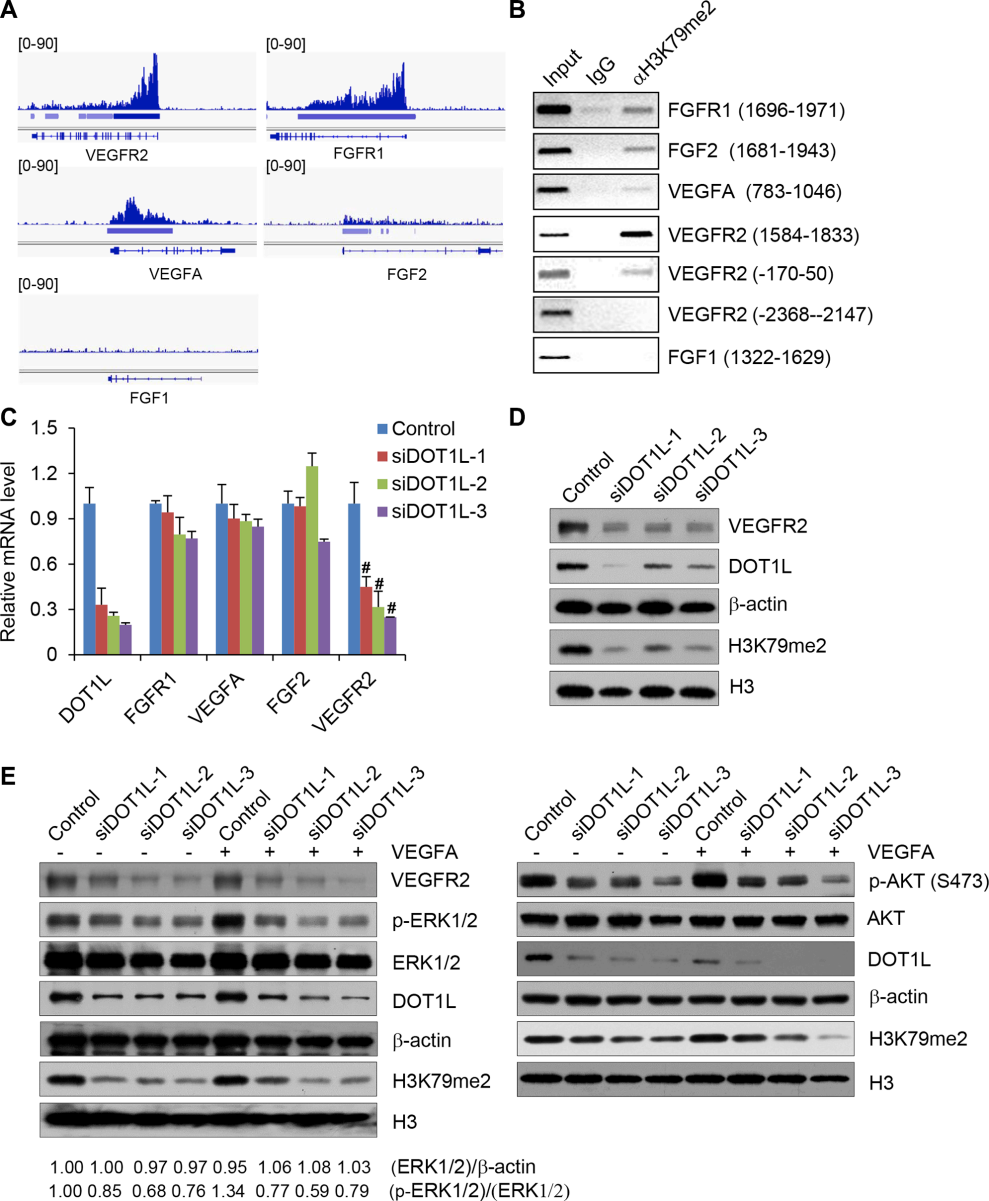
DOT1L regulates the transcription of *VEGFR2* (**A**) Validation of H3K79me2 ChIP-seq enriched regions. ChIP-seq data were displayed on the IGV Browser (http://www.broadinstitute.org/igv). (**B**) ChIP assays were performed in HUVECs using H3K79me2 antibodies and indicated primer pairs. (**C**) DOT1L regulates the transcription of *VEGFR2*. Total mRNAs from HUVECs transfected with DOT1L siRNAs or control siRNAs were extracted and quantitative real-time RT–PCR assays were performed. Data are mean ± SD for *n* = 3; ^#^*P* < 0.01 vs. control (Student's *t* test). (**D**) Decrease of VEGFR2 proteins in HUVECs upon DOT1L knockdown analyzed by western blotting assay. (**E**) Western blotting analysis of ERK1/2 and AKT activation after cultured HUVECs exposure to VEGFA. Confluent HUVECs transfected with DOT1L siRNAs or control siRNAs were stimulated with VEGFA (50 ng/mL) for 15 minutes or not. Cell lysate was obtained, and western blotting was performed with indicated antibodies.

The list of target genes, occupied by H3K79me2, was shown in Table S1, which includes several angiogenic genes such as *VEGFR2*, *FGFR1*, *VEGFA* and *FGF2*. H3K79me2 ChIP-seq data of these selected genes were displayed on the IGV Browser, which showed the occupation of H3K79me2 on the downstream of the TSSs of *VEGFR2*, *FGFR1*, *VEGFA* and *FGF2*, but not *FGF1* which was not enriched in the ChIP-seq data (Figure 5A). Then ChIP assay using H3K79me2 specific antibodies followed by semi-quantitative PCR confirmed the occupation of H3K79me2 on the downstream of TSSs of the selected angiogenic genes but not on an unrelated region of *VEGFR2* (-2368--2147) or *FGF1* (1322–1629) (Figure 5B).

Based on the results that H3K79me2 occupies the downstream of the TSSs of *VEGFR2*, *FGFR1*, *VEGFA* and *FGF2*, we further investigated whether DOT1L regulates the expression of these genes in HUVECs. To this end, total mRNAs from HUVECs transfected with DOT1L siRNAs or control siRNAs were extracted and quantitative real-time RT–PCR assays were performed. The results showed that DOT1L depletion led to no change or a slight decrease in the transcription of *FGF2*, *VEGFA* and *FGFR1*, but a significantly decreased transcription of *VEGFR2* (Figure 5C). Therefore, we focused on *VEGFR2*, which is believed to function as the major positive signal transducer of VEGFA for both physiological and pathological angiogenesis [7, 8], as a target of DOT1L in regulating angiogenesis. Consistent with the results of real-time RT–PCR, western blotting assay indicated that the protein level of VEGFR2 also was decreased in HUVECs upon DOT1L knockdown (Figure 5D). Considering that the activation of downstream ERK1/2 and AKT signaling pathways is an essential step in VEGFR2-mediated angiogenesis in HUVECs, we next analyzed the effect of DOT1L depletion on the ERK1/2 and AKT activation by VEGFA. As shown in Figure 5E, compared with control, DOT1L knockdown in HUVECs resulted in a remarkable inhibition of VEGFA-induced ERK1/2 and AKT phosphorylation/activation. Consistently, overexpression of DOT1L in HUVECs increased the expression of VEGFR2 and the phosphorylation of ERK1/2 and AKT (Supplementary Figure S1A). Furthermore, DOT1L overexpression increased the cell viability, migration, and tube formation ability of HUVECs (Supplementary Figure S1B–S1D).

### DOT1L together with transcription factor ETS-1 activates the transcription of *VEGFR2*

**Figure 6:**
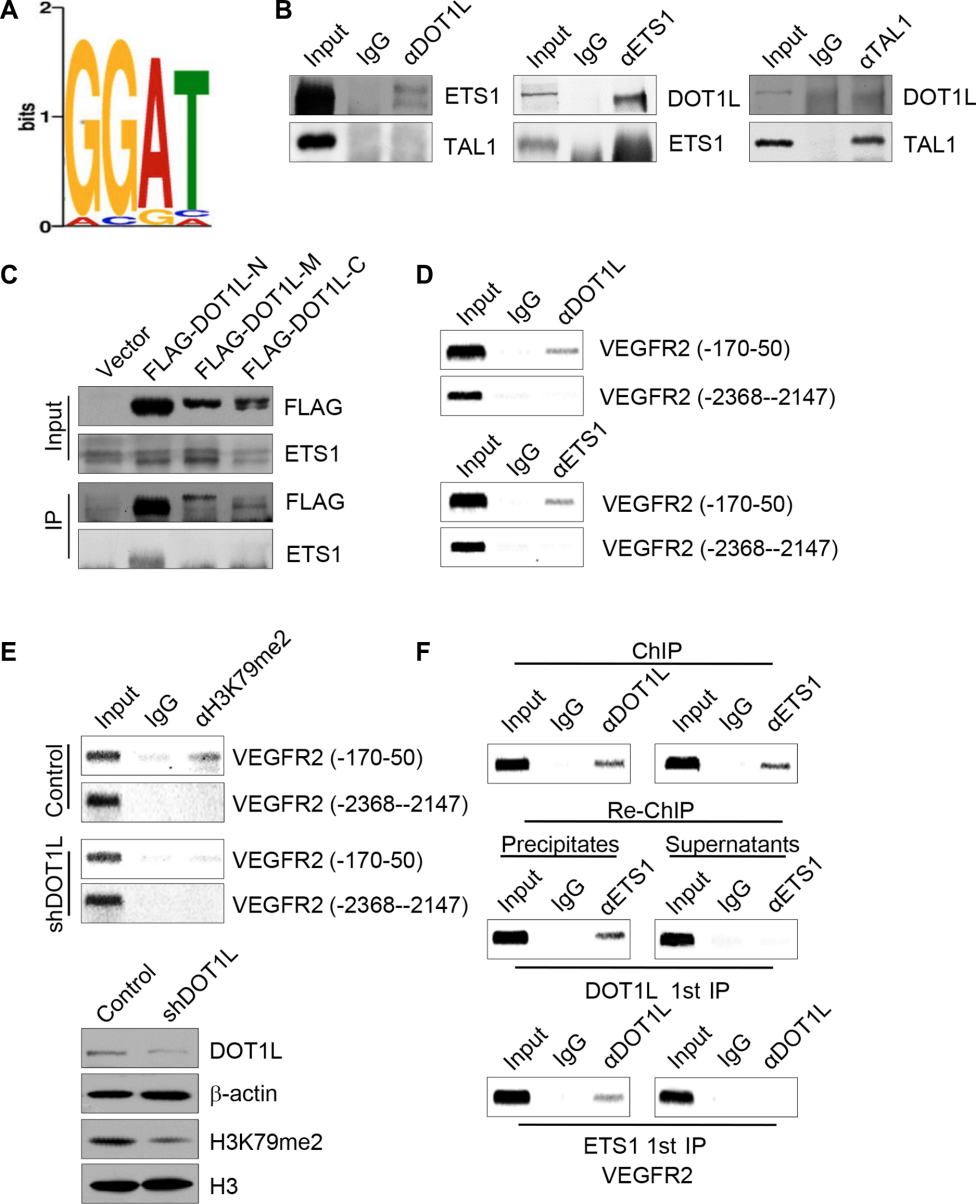

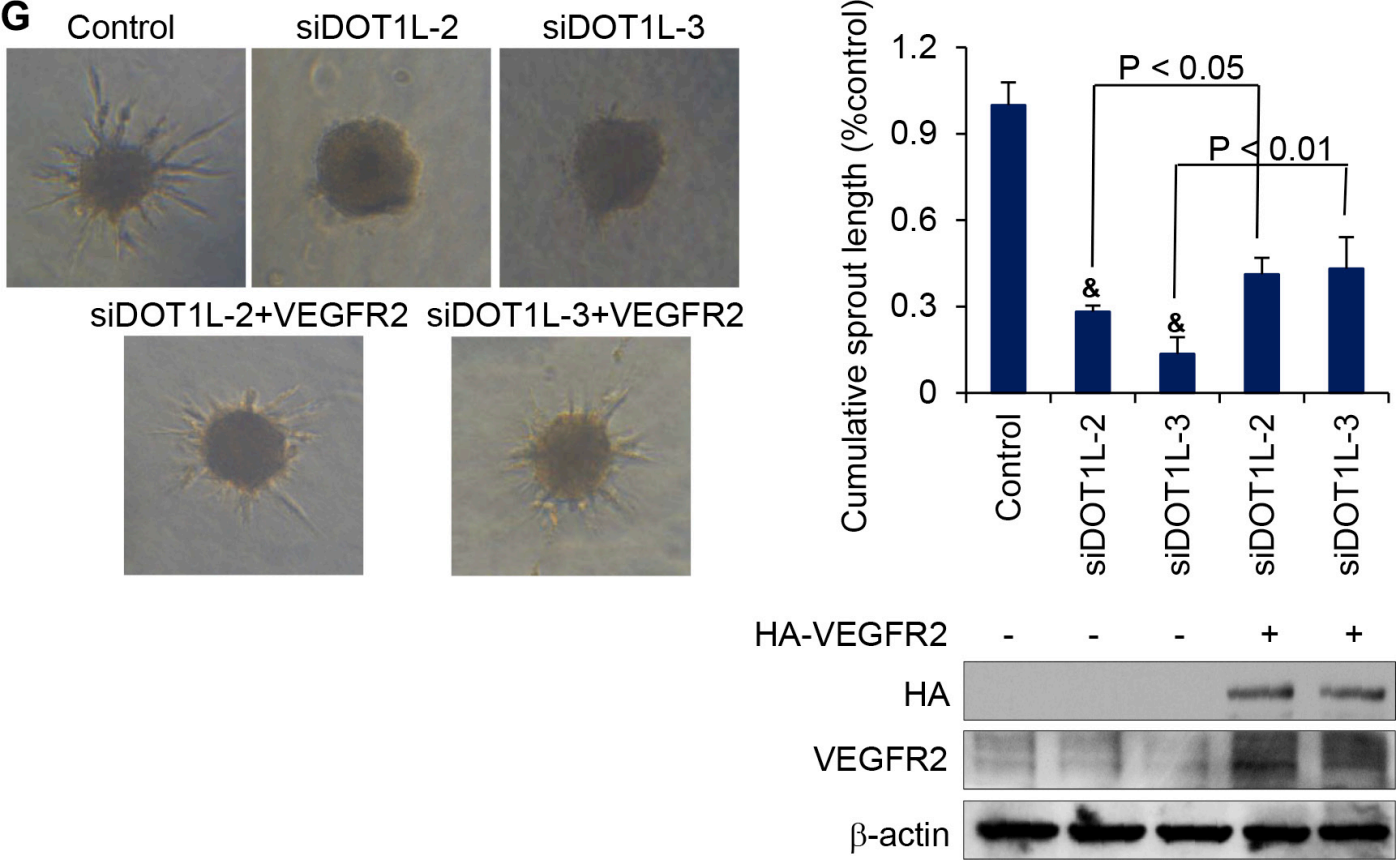
DOT1L cooperates with ETS-1 to regulate the transcription of *VEGFR2* (**A**) H3K79me2 enriched motifs was analyzed using MEME suite. (**B**) Endogeous interaction of DOT1L with ETS-1. Immunoprecipitation assays were performed with antibodies against indicated proteins followed by immunoblotting with antibodies against indicated proteins. (**C**) Identification of domains responsible for the interaction between DOT1L and ETS-1. Whole-cell lysates from HUVECs constantly expressing FLAG-fused DOT1L truncation mutants were prepared and immunoprecipitation was performed with anti-FLAG, followed by immunoblotting with antibodies against ETS-1. (**D**) The recruitment of DOT1L and ETS-1 on the downstream of *VEGFR2* promoter. ChIP assays were performed in HUVECs using the indicated antibodies and primer pairs on *VEGFR2* promoter and gene body. (**E**) DOT1L depletion led to decreased H3K79me2 occupation on *VEGFR2* gene body. ChIP assays were performed in HUVECs expressing DOT1L shRNA or control shRNA using antibodies against H3K79me2 and indicated primers. Western blotting was also performed with anti-DOT1L, anti-β-actin, anti-H3K79me2 or anti-H3. (**F**) DOT1L and ETS-1 exist in the same complex on the downstream of *VEGFR2* promoter. ChIP and Re-ChIP assays were performed with indicated antibodies. (**G**) VEGFR2 was overexpressed in DOT1L depleted-HUVECs, and angiogenic sprouting was compared in the spheroid model. Representative spheroids are shown. Endothelial sprouting capacity is given as cumulative sprout length per spheroid. Data are mean ± SD for *n* = 3; ^&^*P* < 0.05 vs. control (Student's *t* test).

In order to further investigate the mechanism explored by DOT1L to activate the transcription of VEGFR2, the called peaks for H3K79me2 were analyzed by tools in http://meme.sdsc.edu/meme4_4_0/intro.html for consensus elements covered by H3K79me2. Among the elements, we found a GGAT motif (Figure 6A) which is the core binding site of transcription factor ETS-1. The ETS factors are the most important transcription factors which control most aspects of endothelial biology, from early differentiation in the embryo to homeostasis and angiogenesis [28]. Furthermore, by mutational analysis, binding sites for Tal-1 and Ets transcription factors were identified as critical elements for the endothelium-specific *VEGFR2* gene expression in mice [29]. In an effort to elaborate the relationship between DOT1L and ETS-1 mediated angiogenesis, co-immunoprecipitation assay was applied to test whether DOT1L is physically bound to ETS-1. To this end, total proteins from HUVECs were extracted and subjected to co-immunoprecipitation using antibodies against endogenous proteins. Immunoprecipitation with antibodies against DOT1L followed by immunoblotting with antibodies against ETS-1 demonstrated that ETS-1 was efficiently co-immunoprecipitated with DOT1L (Figure 6B). Reciprocally, immunoprecipitation with antibodies against ETS-1 followed by immunoblotting with antibodies against DOT1L also confirmed the interaction between these two proteins (Figure 6B). And this interaction is specific, because the interaction between DOT1L and TAL-1 which also regulates the expression of VEGFR2 could not be detected in HUVECs (Figure 6B).

To illustrate the molecular detail involved in the interaction between DOT1L and ETS-1, co-immunoprecipitation assays were performed using FLAG-fused DOT1L truncation mutants, including FLAG-DOT1L-N (N-terminal fragment, 1–467 aa), FLAG-DOT1L-M (middle region, 468–1002 aa), and FLAG-DOT1L-C (C-terminal fragment, 1003–1537 aa). The result showed that ETS-1 was able to specifically interact with the N-terminal domain of DOT1L (Figure 6C), the overexpression of which could increase the expression of VEGFR2 (Supplementary Figure S2).

In order to detect whether ETS-1 together with DOT1L could indeed bind to the gene body of *VEGFR2*, ChIP assays were performed in HUVECs with antibodies against ETS-1, DOT1L, or control IgG. As shown in Figure 6D, both ETS-1 and DOT1L occupied the gene body of *VEGFR2*. To clarify the essential role of DOT1L in the occupation of H3K79me2 on the gene body of *VEGFR2*, ChIP assays were performed in HUVECs expressing DOT1L shRNA or control shRNA. The results indicated that DOT1L knockdown led to a dramatically decreased occupation of H3K79me2 on *VEGFR2* TSS downstream sequence (Figure 6E). To further support the hypothesis that ETS-1 and DOT1L interact and exist in the same complex on *VEGFR2* gene body, sequential ChIP/Re-ChIP assays were performed. In these experiments, soluble chromatins were first immunoprecipitated with ETS-1 antibodies. Both the supernatants and the immunoprecipitates were subsequently re-immunoprecipitated with antibodies against DOT1L. The results of the experiments indicated that the TSS downstream of *VEGFR2* that was immunoprecipitated with antibodies against ETS-1 could be reimmunoprecipitated with antibodies against DOT1L, whereas in the supernatants, antibodies against DOTL1 found no detectable *VEGFR2* gene sequence (Figure 6F). The same results held when the initial ChIP was done with antibodies against DOT1L; the TSS downstream of *VEGFR2* could only be detected in precipitates, but not in supernatants after Re-ChIP with antibodies against ETS-1 (Figure 6F). Taken together, these experiments support the idea that ETS-1 is physically associated with DOT1L on the *VEGFR2* gene body.

To further substantiate the role of VEGFR2 in DOT1L-promoted angiogenesis, VEGFR2 expression constructs were transfected into DOT1L-depleted HUVECs, and *in vitro* spheroid sprouting assays were performed. The result showed that overexpression of VEGFR2 partially compensated for the impaired sprouting in DOT1L siRNA-transfected endothelial cells (Figure 6G). Collectively, these data support that VEGFR2 as a target of DOT1L in its transcription regulation is responsible for the angiogenic function of DOT1L.

## DISCUSSION

Eukaryotic DNA is wrapped around a histone octamer (H3/H4 heterotetramer and two H2A/H2B dimers) to form the nucleosome, the basic unit of chromatin [30, 31]. Histones are subjected to a variety of post-translational modifications, including phosphorylation, ubiquitination, acetylation, methylation, and so on. These modifications which influence chromatin structure through affecting the recruitment of effector proteins to specific chromatin regions [32, 33], play an important role in the establishment and maintenance of gene expression patterns in different types of cell [34]. Enzymes responsible for adding or removing these modifications regulate all DNA-based processes, such as gene expression. Consequently, abnormal expression patterns of these histone modifiers and erasers or inhibiting their activities can have profound results and lead to the induction or inhibition of some cellular process.

Angiogenesis is a tightly regulated process, which has essential roles in embryonic development, wound healing, and organ regeneration. During tumor progression, an “angiogenic switch” is activated and remains on, causing normally quiescent vasculature to sprout new vessels to supply nutrients and oxygen to tumor cells. Several epigenetic factors have been reported to regulate physiological or pathological angiogenesis. For example, MLL is required for proangiogenic endothelial cell functions through regulating the expression of Hox genes [35]; silencing of HDAC5 exhibits a proangiogenic effect through up-regulating the transcription of FGF2 and SLLT2 [36]; GATA1 recruits SET7 to enhance the expression of VEGF in breast cancer cells, which promotes breast tumor angiogenesis [37]. Understanding new fundamental mechanisms governing angiogenesis will offer novel approaches to prevent vascular associated disease. The present study, for the first time, describes the proangiogenic role of DOT1L in endothelial cells. We showed that silencing of DOT1L in HUVECs leads to decreased cell viability, migration, tube formation, and capillary sprout formation *in vitro*, as well as reduced formation of functional vascular networks in matrigel plugs *in vivo*. By demonstrating the essential angiogenic function of DOT1L in endothelial cells, this study adds to the growing recognition of the physiologic role of DOT1L.

Mechanically, our work, for the first time, represents the DOT1L-dependent transcriptional profile in endothelial cells and provides novel insights into the DOT1L-regulated genes, which are well known to control angiogenesis. From the list of putative targets obtained via genomic annotation of H3K79me2 profile, we here confirmed the DOT1L-dependent regulation of VEGFR2, a well-established proangiogenic factor. At the meantime, the called peaks for H3K79me2 were found to cover the binding motif of ETS-1, which is essential for the regulation of VEGFR2 and angiogenesis. We found that DOT1L and ETS-1 interacts with each other and coexists on the downstream of TSS of *VEGFR2* to promote the transcription of this gene. DOT1L knockdown resulted in a decrease in the mRNA and protein level of VEGFR2, as well as a marked inhibition of VEGFA-induced ERK1/2 and AKT phosphorylation/activation. There are also other targets of DOT1L in HUVECs which have proangiogenic functions. For example, DOT1L depletion was also associated with a slight decrease in the transcription of *FGFR1* (Figure 5C), and a decreased migration of HUVECs and tube formation in response to hFGF-B (Supplementary Figure S3). This is one possible reason why overexpression of VEGFR2 only partially compensated for the impaired sprouting in DOT1L siRNA-transfected HUVECs. The other possible reason is that the efficiency of plasmids transfection into HUVECs is low (Supplementary Figure S4).

In conclusion, the data presented here provide evidence for a novel regulatory mechanism involved in VEGFR2 expression and angiogenesis. We predict that inhibition of DOT1L would have antiangiogenic consequences and eventually lead to regression of tumors due to the disruption of vascular supply. Our findings also point to DOT1L as a potential therapeutic target for other vascular-related disorders.

## MATERIALS AND METHODS

### Cell culture and antibodies

Pooled human umbilical vein endothelial cells (HUVECs) were purchased from Lonza (Walkersville, MD, USA) and cultured in endothelial basal medium (EBM) supplemented with hydrocortisone, fetal bovine serum (FBS), human fibroblast growth factor-basic (hFGF-B), vascular endothelial growth factor (VEGF), R3 insulin like growth factor-1 (R3-IGF-1), ascorbic acid, human epidermal growth factor (hEGF), GA-1000, and heparin (all from Lonza), at 37°C in a humidified atmosphere with 5% CO_2_. Antibodies against VEGFR2 (YM0277) were bought from Immunoway Biotechnology Company (Plano, TX, USA). Antibodies against β-actin (A1978) were from Sigma-Aldrich (St. Louis, MO, USA). Antibodies against ERK1/2 (4695) and phosphorylated ERK1/2 (4370) were from Cell Signaling Technology (Danvers, MA, USA). Antibodies against ETS-1 were from Proteintech Inc. (Chicago, IL, USA). Antibodies against DOT1L (ab57827), H3K79me2 (ab3594), H3 (ab1791) and normal IgG (ab27478) were all from Abcam Inc. (Cambridge, MA, USA).

### RNA interference and plasmid transfection

For siRNA-mediated silencing, DOT1L siRNA-1 (GAGUGUUAUAUUUGUGAAU), DOT1L siRNA-2 (CACCUCUGAACUUCAGAAU), and DOT1L siRNA-3 (GAUCAGCAUUGUGGAGCUA) was transfected into HUVECs respectively using RNAimax (Invitrogen, Carlsbad, CA, USA) according to the manufacturer's instruction. Scrambled siRNA (UUCUCCGAACGU GUCACGU) was used as a control. To perform the spheroid assay in mice, the targeting sequences of DOT1L siRNA-2 and siRNA-3 were cloned into pLL3.7 lentiviral vectors separately. The recombinant construct, together with three assistant vectors (pRRE, VSVG, and RSV/REV), were then transiently transfected into HEK 293T cells. Viral supernatants were collected both 24 hour and 48 hour later, clarified by filtration, and concentrated by ultracentrifugation. The HUVECs which were infected by these concentrated viruses and sorted by EGFP expression were subjected to spheroid assays in mice. For HA-VEGFR2 expression plasmids transfection, HUVECs (3.5 × 10^5^ cells /6-cm well) were grown to 60% to 70% confluence and then transfected with 3 μg of plamid DNA. Transfection was performed using Targefect-HUVEC from Advanced Targeting Systems (San Diego, CA, USA) according to the manufacturer's protocol.

### MTT viability assay

Assessment of cell viability was performed using the 3-(4, 5-dimethylthiazol-2-yl)-2, 5-diphenyl-2H-tetrazolium bromide (MTT) assay. Seventy-two hours after transfection of indicated siRNAs, 0.5 mg/ml MTT was added to each well, and cells were incubated for 4 hours at 37°C. Cells were washed with phosphate-buffered saline (PBS) and lysed for 30 minutes at room temperature with lysis buffer (0.1 M HCl in isopropanol). Absorbance was photometrically measured at 550 nm.

### Apoptosis assay

Apoptosis was measured using FITC annexin V apoptosis detection kit (BD Biosciences, San Jose, California, USA) according to the manufacturer's instruction. Briefly, cells were digested with trypsin-EDTA into single cell suspension and collected by centrifugation at 1,000 rpm for five minutes to remove the supernatant. The cells were resuspended in 100 μl of annexin V binding solution and 3 μl of annexin V-FITC. After incubation at room temperature for 15 minutes in the dark, 400 μl of annexin V binding solution and 3 μl of propidium iodide were added into the cell suspension which was then subjected to flow cytometry.

### EdU incorporation assay

EdU (5-ethynyl-2′-deoxyuridine) assays were performed according to the manufacturer's instruction (RiboBio). Briefly, cells were cultured in 48-well plate forty-eight hours after siRNA transfection. Twenty-four hours later, the culture medium was replaced with medium containing EdU for 2 hours. Cells were fixed in 4% paraformaldehyde and processed for immunofluorescence.

### Real-time RT-PCR

Total RNA was isolated using Trizol reagent (Invitrogen), and then 2 μg of RNA from each sample was reverse-transcribed (RT) into cDNA and subjected to real-time PCR using SYBR Green I Kit (Roche Applied Science, Mannheim, Germany). The primer pairs used for real-time PCR were as follows: *GAPDH* forward, 5′-ACCCAGAAGACTGTGGATGG-3′; *GAPDH* reverse, 5′-TCTAGACGGCAGGTCAGGTC-3′; *VEGFR2* forward, 5′-ATTGCTTCTGTTAGTGACCA-3′; *VEGFR2* reverse, 5′-AAATCCTATACCCTACAACGAC-3′; *FGFR1* forward, 5′-CAATGTTTCAGATGCTCTCCC-3′; *FGFR1* reverse, 5′-CCTTGTAGCCTCCAATTCTG-3′; *FGF2* forward, 5′-TCAAGCTACAACTTCAAGCAG-3′; *FGF2* reverse, 5′-CCGTAACACATTTAGAAGCCAG-3′; *VEGFA* forward, 5′-GAAGTTCATGGATGTCTATCAG-3′; *VEGFA* reverse, 5′-CTTTCTTTGGTCTGCATTCAC-3′.

### Chromatin immunoprecipitation (ChIP)

The ChIP experiments were performed as described previously [39, 40]. Briefly, HUVECs were washed twice with PBS and cross-linked for 10 minutes with 1% formaldehyde. Then cells were rinsed twice with and collected into ice-cold PBS. Cells were pelleted and resuspended in lysis buffer (1% SDS, 10 mM EDTA, 50 mM Tris-HCl, pH 8.1, 1 × protease inhibitor cocktail) and sonicated for 10 cycles with Max amplitude (H mode) (30 seconds on, 30 seconds off) using water-bath sonicator (Fisher Sonic Dismembrantor; Model 300) before centrifugation for 10 minutes. Then immunoprecipitation was performed using antibodies against H3K79me2, DOT1L, ETS-1, or normal IgG as a control. The eluted DNA fragments were purified with a DNA purification kit (QIAquick Spin Kit; Qiagen, Valencia, CA). For regular PCR, 1 μl of the 40 μl DNA extracts was used, and 25–30 cycles were allowed. Primer pairs used in ChIP assays were as follows: *VEGFR2* (1584–1833) ChIP primer, 5′-ATGAAGGTCTGGAACATGTG-3′ (forward) and 5′-AATTTGTGCACTGTTACCCT-3′ (reverse); *VEGFR2* (−170–50) ChIP primer, 5′-GTGTGGGGA AATGGGGAGATG-3′ (forward) and 5′-AAACGCAGC GACCACACATTG-3′ (reverse); *VEGFR2* (−2368–2147) ChIP primer, 5′-GGAACACTCAACACATTTGG-3′ (forward) and 5′-ATCAGTCTACCACATTCCCT-3′ (reverse); *FGF1* (1322–1629) ChIP primer, 5′-ACTATCCACACCC ACATCAC-3′ (forward) and 5′-ACTCGTAAACAAT CGCATCC-3′ (reverse); *FGF2* (1681–1943) ChIP primer, 5′-AACCTGTCCTCCTGTAAGTG-3′ (forward) and 5′-AGTGTATTGCCCATTCTTCTG-3′ (reverse); *VEGFA* (783–1046) ChIP primer, 5′-GAGGAGGAAGAAGAG AAGGA-3′ (forward) and 5′-AAAGTTCATGGTTT CGGAGG-3′ (reverse); *FGFR1* (1696–1971) ChIP primer, 5′-GTCCCAATCTGGTTCCTAGAG-3′ (forward) and 5′-GGGCTCAGAACAAGAAACAG-3′ (reverse).

### Tube formation assay

Forty-eight hours after transfection of siRNAs, HUVECs (5 × 10^4^) were cultured in a 12-well plate (BD Biosciences) coated with 200 μl matrigel basement membrane matrix (BD Biosciences). Pictures were taken 24 hours later in 4 random microscopic fields with a computer-assisted microscope (Leica, Plant, Germany). Tube length was quantified by measuring the cumulative tube length using WimTube quantitative tube formation image analysis.

### Sprouting assay

Spheroids of HUVECs (750 cells) were generated in hanging drops as described [41]. They were suspended in endothelial cell growth medium (EGM) containing 1 mg/ml rat tail collagen I (BD Biosciences) and 0.25% (wt/vol) methylcellulose (Sigma) and distributed in 48- well plates (30 spheroids /well). After solidification at 37 °C for 30 minutes, the mixture was overlaid with 500 μl EGM. Pictures were taken 24 hours later using a microscope (Leica), and the cumulative sprout length of 6 random spheroids was quantified using WimSprout quantitative sprouting spheroid assay image analysis.

### Migration assay

A total of 3 × 10^5^ HUVECs transfected with DOT1L siRNAs or scrambled siRNAs were resuspended in 200 μl EBM medium (Lonza) and placed in the upper chamber (BD Bioscicences, 8-μm pore size) coated with 50 μl matrigel (BD Biosciences). Then, the chamber was placed in a 24-well culture dish (BD Biosciences) containing 500 μl EGM supplemented with 50 ng/ml VEGF or not. After incubation for 24 hours at 37°C, transmigrated cells were counted manually in 5 random microscopic fields.

### *In vivo* spheroid assay in mice

Spheroids of HUVECs expressing DOT1L or control shRNA were generated in EBM medium, which were then mixed with matrigel containing 100 ng/ml VEGFA, and subcutaneously injected (1000 spheroids/plug) into 6- to 8-week-old CB17 SCID (severe combined immunodeficiency) mice [20]. Matrigels were harvested eight days after implantation. HUVECs-derived blood vessel density in matrigel plugs was quantified by hematoxylin and eosin staining. To visualize the formation of a human EC–derived neovasculature, plug sections were stained with anti-hCD31 and FITC-Lectin, and nuclei were stained with 4, 6-diamidino-2-phenylindole (DAPI). Investigation has been conducted in accordance with the ethical standards and according to the Declaration of Helsinki and according to national and international guidelines and has been approved by Institutional Animal Care and Use Committees of Tianjin Medical University.

### ChIP-seq data analysis

The H3K79me2 ChIP-seq data in HUVECs was from the ENCODE project (Broad/MGH ENCODE group; GSM1003555) [24]. H3K79me2 ChIP-seq peaks was called by CEAS (Cis-regulation element annotation system) to obtain the genomic annotation of H3K79me2 profile [42]. ChIP-seq profiling of H3K79me2 and H3K27ac in HUVECs over a 5-kb window around TSSs of all RefSeq genes was performed by seqMINER [43]. The H3K79me2 ChIP-seq enriched regions on tested genes were displayed on the IGV Browser (http://www.broadinstitute.org/igv).

## SUPPLEMENTARY MATERIALS




