# Supplementary material for: DOT1L promotes angiogenesis through cooperative regulation of VEGFR2 with ETS-1
Source: Oncotarget. 2016 Sep 10;7(43):69674–87. doi: 10.18632/oncotarget.11939 (PMC5342507; doi:10.18632/oncotarget.11939)
Supplement: Supplementary file 1 [file oncotarget-07-69674-s001.pdf]

# DOT1L promotes angiogenesis through cooperative regulation of VEGFR2 with ETS-1

## Supplementary Materials

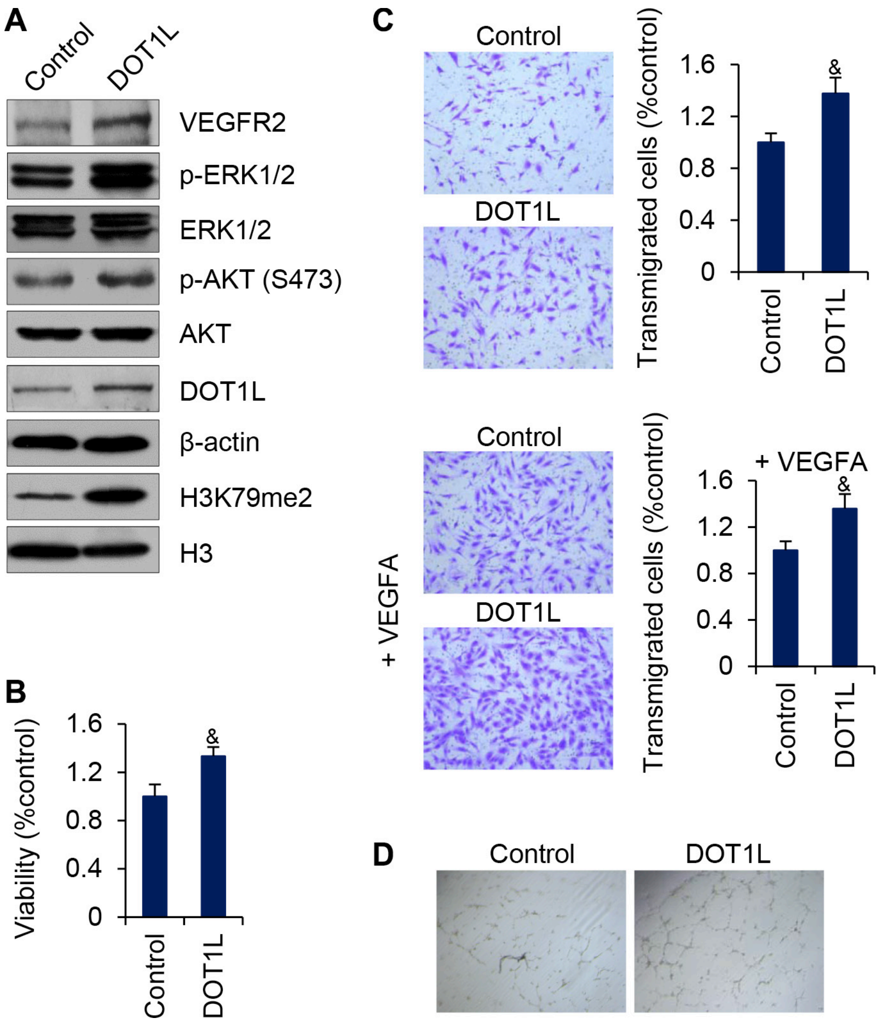


**Supplementary Figure S1: DOT1L overexpression increases VEGFR2 expression as well as cell viability, migration, and tube formation ability of HUVECs.** DOT1L expression constructs were transfected into HUVECs. (**A**) Western blotting was performed with indicated antibodies. (**B**) Cell viability was measured using MTT assay. Data are mean ± SD for $n = 3$, $^{\&}P < 0.05$ vs. control (Student's *t* test). (**C**) Transmigration of HUVECs was determined using transwell assays. Data are mean ± SD for $n = 3$, $^{\&}P < 0.05$ vs. control. (**D**) The matrigel network assay was detected *in vitro*.

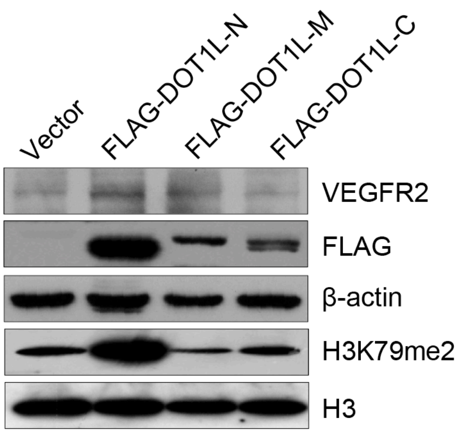


**Supplementary Figure S2: Overexpression of the N-terminal fragment of DOT1L increases the expression of VEGFR2.** DOT1L truncation expression constructs were transfected into HUVECs, and western blotting was performed with indicated antibodies.

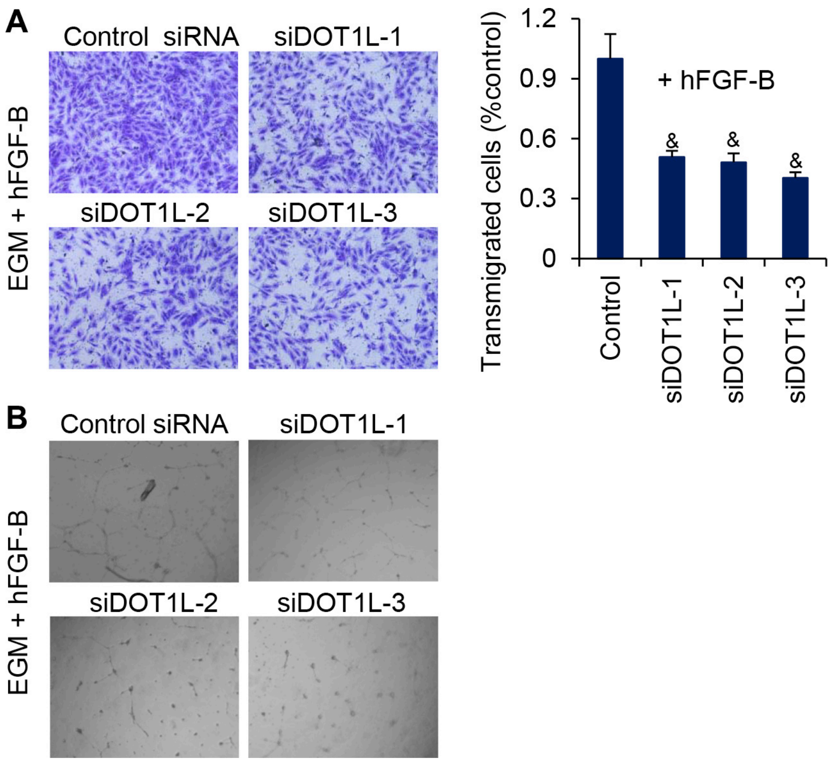


**Supplementary Figure S3: DOT1L silencing suppresses migration of HUVECs and tube formation in response to hFGF-B.** DOT1L was silenced in HUVECs by three different siRNAs (DOT1L-1, –2, and –3). (**A**) Migration of HUVECs towards hFGF-B was determined using transwell assays. The transmigrated cells were counted. Data are mean ± SD for $n = 3$; $^{\&}P < 0.05$ vs. control (Student's *t* test). (**B**) Endothelial tube formation capacity is shown.

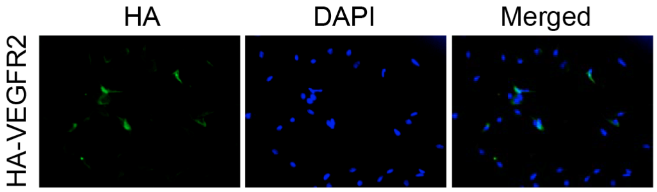


**Supplementary Figure S4: The efficiency of HA-VEGFR2 expression plasmids transfection was tested by immunofluorescence.** HUVECs transfected with HA-VEGFR2 expression plasmids were fixed and stained with anti-HA. Green, HA- VEGFR2. Blue, DAPI.

**Supplementary Table S1: Target genes occupied by H3K79me2 in HUVECs.** See Supplementary_Table_S1
