# Supplementary material for: DOT1L promotes angiogenesis through cooperative regulation of VEGFR2 with ETS-1
Source: Oncotarget. 2016 Sep 10;7(43):69674–87. doi: 10.18632/oncotarget.11939 (PMC5342507; doi:10.18632/oncotarget.11939)
Supplement: Supplementary file 2 [file oncotarget-07-69674-s002.docx]

Table S1 Target genes occupied by H3K79me2 in HUVECs

| HGNC_Symbol | chrom | txStart | txEnd |
| --- | --- | --- | --- |
| UBE2J2 | chr1 | 1189291 | 1209234 |
| SSU72 | chr1 | 1478559 | 1510262 |
| GNB1 | chr1 | 1716724 | 1822526 |
| SKI | chr1 | 2160133 | 2241652 |
| RER1 | chr1 | 2323213 | 2336885 |
| PANK4 | chr1 | 2439974 | 2458035 |
| RPL22 | chr1 | 6245079 | 6259679 |
| KLHL21 | chr1 | 6650783 | 6662929 |
| ERRFI1 | chr1 | 8071778 | 8086393 |
| ENO1 | chr1 | 8921058 | 8939151 |
| SPSB1 | chr1 | 9352940 | 9429590 |
| KIF1B | chr1 | 10271673 | 10441661 |
| TARDBP | chr1 | 11072678 | 11085549 |
| PRDM2 | chr1 | 14075855 | 14114574 |
| EFHD2 | chr1 | 15736390 | 15756839 |
| DDI2 | chr1 | 15943952 | 15987552 |
| FBLIM1 | chr1 | 16083153 | 16113084 |
| SPEN | chr1 | 16174358 | 16266950 |
| EPHA2 | chr1 | 16450831 | 16482582 |
| SZRD1 | chr1 | 16693524 | 16724643 |
| RCC2 | chr1 | 17733250 | 17766250 |
| IFFO2 | chr1 | 19230773 | 19282826 |
| UBR4 | chr1 | 19400999 | 19536746 |
| CAPZB | chr1 | 19665266 | 19812066 |
| HP1BP3 | chr1 | 21069170 | 21101447 |
| ECE1 | chr1 | 21543739 | 21672034 |
| USP48 | chr1 | 22004791 | 22109688 |
| HSPG2 | chr1 | 22148736 | 22263750 |
| CDC42 | chr1 | 22379119 | 22419436 |
| KDM1A | chr1 | 23345940 | 23410184 |
| LUZP1 | chr1 | 23410515 | 23495517 |
| HNRNPR | chr1 | 23636275 | 23670853 |
| ID3 | chr1 | 23884420 | 23886285 |
| RPL11 | chr1 | 24018268 | 24022915 |
| TCEB3 | chr1 | 24069855 | 24088549 |
| PNRC2 | chr1 | 24286300 | 24289949 |
| SRSF10 | chr1 | 24295572 | 24306953 |
| SRRM1 | chr1 | 24969593 | 24999772 |
| CLIC4 | chr1 | 25071759 | 25170815 |
| C1orf63 | chr1 | 25568739 | 25664656 |
| STMN1 | chr1 | 26210676 | 26232993 |
| MIR3917 | chr1 | 26232852 | 26232945 |
| ARID1A | chr1 | 27022521 | 27108601 |
| SLC9A1 | chr1 | 27425299 | 27481621 |
| WASF2 | chr1 | 27730733 | 27816678 |
| STX12 | chr1 | 28099693 | 28150963 |
| PPP1R8 | chr1 | 28157324 | 28178183 |
| RCC1 | chr1 | 28832454 | 28865708 |
| SNHG3 | chr1 | 28832454 | 28837404 |
| SNHG12 | chr1 | 28905049 | 28908366 |
| YTHDF2 | chr1 | 29063461 | 29096287 |
| SRSF4 | chr1 | 29474249 | 29508637 |
| PUM1 | chr1 | 31404352 | 31538763 |
| TINAGL1 | chr1 | 32042085 | 32053287 |
| PTP4A2 | chr1 | 32372021 | 32403988 |
| KHDRBS1 | chr1 | 32479294 | 32509482 |
| KPNA6 | chr1 | 32573888 | 32642168 |
| YARS | chr1 | 33240839 | 33283633 |
| PHC2 | chr1 | 33789223 | 33815499 |
| SMIM12 | chr1 | 35315962 | 35325338 |
| SFPQ | chr1 | 35649200 | 35658743 |
| PSMB2 | chr1 | 36035412 | 36107445 |
| MAP7D1 | chr1 | 36621802 | 36646441 |
| THRAP3 | chr1 | 36690016 | 36770957 |
| ZC3H12A | chr1 | 37940118 | 37949978 |
| FHL3 | chr1 | 38462441 | 38471187 |
| KIAA0754 | chr1 | 39875175 | 39882154 |
| PABPC4 | chr1 | 40026484 | 40042521 |
| CAP1 | chr1 | 40506254 | 40538321 |
| RLF | chr1 | 40627040 | 40706593 |
| CTPS1 | chr1 | 41445006 | 41478235 |
| FOXJ3 | chr1 | 42642209 | 42800636 |
| YBX1 | chr1 | 43148065 | 43168020 |
| TIE1 | chr1 | 43766565 | 43788781 |
| CDC20 | chr1 | 43824778 | 43828873 |
| HYI | chr1 | 43916673 | 43919660 |
| RPS8 | chr1 | 45241245 | 45244412 |
| SNORD55 | chr1 | 45241536 | 45241610 |
| PLK3 | chr1 | 45266035 | 45271667 |
| BTBD19 | chr1 | 45274153 | 45279801 |
| PRDX1 | chr1 | 45976706 | 45987610 |
| NASP | chr1 | 46067732 | 46075197 |
| GPBP1L1 | chr1 | 46092975 | 46152302 |
| LRRC41 | chr1 | 46744071 | 46769038 |
| TAL1 | chr1 | 47681962 | 47695443 |
| RNF11 | chr1 | 51701944 | 51739119 |
| NRD1 | chr1 | 52254865 | 52344609 |
| DHCR24 | chr1 | 55315299 | 55352921 |
| JUN | chr1 | 59246462 | 59249785 |
| USP1 | chr1 | 62902382 | 62917475 |
| JAK1 | chr1 | 65298905 | 65432187 |
| LEPR | chr1 | 65886130 | 66101111 |
| LEPROT | chr1 | 65886130 | 65901690 |
| PDE4B | chr1 | 66258192 | 66840262 |
| MIER1 | chr1 | 67395925 | 67454302 |
| SERBP1 | chr1 | 67873492 | 67896123 |
| GNG12 | chr1 | 68167148 | 68299155 |
| SRSF11 | chr1 | 70695960 | 70717701 |
| ZRANB2 | chr1 | 71528973 | 71546972 |
| RABGGTB | chr1 | 76251878 | 76260775 |
| SNORD45C | chr1 | 76252756 | 76252834 |
| SNORD45A | chr1 | 76253573 | 76253657 |
| ZZZ3 | chr1 | 78030189 | 78148343 |
| FUBP1 | chr1 | 78413590 | 78444777 |
| DNAJB4 | chr1 | 78470635 | 78482995 |
| ELTD1 | chr1 | 79355448 | 79472495 |
| GNG5 | chr1 | 84964005 | 84972262 |
| BCL10 | chr1 | 85731459 | 85742587 |
| DDAH1 | chr1 | 85784167 | 86044046 |
| CYR61 | chr1 | 86046443 | 86049648 |
| SH3GLB1 | chr1 | 87170252 | 87213867 |
| 15-Sep | chr1 | 87328127 | 87380107 |
| LRRC8C | chr1 | 90098643 | 90185094 |
| RPL5 | chr1 | 93297593 | 93307481 |
| MTF2 | chr1 | 93544791 | 93604638 |
| TMED5 | chr1 | 93615298 | 93646246 |
| DR1 | chr1 | 93811477 | 93828148 |
| BCAR3 | chr1 | 94027342 | 94146926 |
| GCLM | chr1 | 94352589 | 94375012 |
| ARHGAP29 | chr1 | 94634462 | 94703307 |
| CNN3 | chr1 | 95362506 | 95392735 |
| PALMD | chr1 | 100111430 | 100160097 |
| HIAT1 | chr1 | 100503788 | 100548929 |
| S1PR1 | chr1 | 101702304 | 101707076 |
| SLC25A24 | chr1 | 108677343 | 108742980 |
| PRPF38B | chr1 | 109234931 | 109244422 |
| SARS | chr1 | 109756514 | 109760113 |
| GNAI3 | chr1 | 110091185 | 110138454 |
| AMPD2 | chr1 | 110162434 | 110174677 |
| RBM15 | chr1 | 110881944 | 110889303 |
| CTTNBP2NL | chr1 | 112938799 | 113003786 |
| CAPZA1 | chr1 | 113162074 | 113214241 |
| RHOC | chr1 | 113243748 | 113250025 |
| SLC16A1 | chr1 | 113454469 | 113498685 |
| HIPK1 | chr1 | 114471995 | 114520491 |
| TRIM33 | chr1 | 114935398 | 115053781 |
| CSDE1 | chr1 | 115259533 | 115300671 |
| ATP1A1 | chr1 | 116916488 | 116947396 |
| ZNF697 | chr1 | 120161999 | 120190390 |
| PDE4DIP | chr1 | 144890590 | 144932032 |
| SEC22B | chr1 | 145096406 | 145116997 |
| NOTCH2NL | chr1 | 145224596 | 145285912 |
| LIX1L | chr1 | 145477084 | 145499091 |
| RPRD2 | chr1 | 150336989 | 150449041 |
| MCL1 | chr1 | 150547026 | 150552214 |
| CERS2 | chr1 | 150937648 | 150947479 |
| CDC42SE1 | chr1 | 151023446 | 151032125 |
| MLLT11 | chr1 | 151032150 | 151040973 |
| PIP5K1A | chr1 | 151171020 | 151222007 |
| S100A10 | chr1 | 151955385 | 151966714 |
| S100A16 | chr1 | 153579366 | 153585514 |
| ILF2 | chr1 | 153634263 | 153643504 |
| GATAD2B | chr1 | 153777202 | 153895451 |
| TPM3 | chr1 | 154127779 | 154155725 |
| C1orf43 | chr1 | 154179182 | 154193273 |
| UBAP2L | chr1 | 154193324 | 154243986 |
| SHE | chr1 | 154451953 | 154474526 |
| UBE2Q1 | chr1 | 154521050 | 154531120 |
| ADAR | chr1 | 154554533 | 154580724 |
| SHC1 | chr1 | 154934774 | 154941090 |
| CKS1B | chr1 | 154947117 | 154951725 |
| FDPS | chr1 | 155278538 | 155290457 |
| ASH1L | chr1 | 155305051 | 155532324 |
| YY1AP1 | chr1 | 155629232 | 155658447 |
| KIAA0907 | chr1 | 155882835 | 155904188 |
| ARHGEF2 | chr1 | 155916629 | 155959864 |
| LMNA | chr1 | 156052368 | 156108548 |
| SMG5 | chr1 | 156219014 | 156252620 |
| CCT3 | chr1 | 156278751 | 156308206 |
| MEF2D | chr1 | 156433519 | 156470634 |
| ISG20L2 | chr1 | 156692412 | 156697705 |
| HDGF | chr1 | 156711898 | 156736717 |
| PRCC | chr1 | 156737273 | 156770609 |
| PEAR1 | chr1 | 156863522 | 156886226 |
| TAGLN2 | chr1 | 159887896 | 159895332 |
| PEA15 | chr1 | 160175124 | 160185162 |
| COPA | chr1 | 160258376 | 160313354 |
| PFDN2 | chr1 | 161070345 | 161087866 |
| ATF6 | chr1 | 161736033 | 161933860 |
| UAP1 | chr1 | 162531295 | 162569633 |
| UCK2 | chr1 | 165796731 | 165880855 |
| ATP1B1 | chr1 | 169075946 | 169101960 |
| PRRC2C | chr1 | 171454665 | 171562650 |
| TNFSF4 | chr1 | 173152869 | 173176471 |
| GAS5-AS1 | chr1 | 173832385 | 173833079 |
| GAS5 | chr1 | 173833038 | 173837125 |
| SNORD74 | chr1 | 173836811 | 173836883 |
| CACYBP | chr1 | 174968891 | 174981163 |
| RFWD2 | chr1 | 175913966 | 176176370 |
| ABL2 | chr1 | 179068461 | 179112224 |
| IER5 | chr1 | 181057637 | 181059979 |
| DHX9 | chr1 | 182808438 | 182857117 |
| LAMC1 | chr1 | 182992594 | 183114727 |
| IVNS1ABP | chr1 | 185265521 | 185286461 |
| NEK7 | chr1 | 198126107 | 198291548 |
| MIR181A1HG | chr1 | 198777131 | 198906558 |
| ZNF281 | chr1 | 200375419 | 200379166 |
| CAMSAP2 | chr1 | 200708685 | 200829831 |
| CSRP1 | chr1 | 201452657 | 201475967 |
| IPO9 | chr1 | 201798287 | 201853422 |
| ZBED6 | chr1 | 203766650 | 203769590 |
| ZC3H11A | chr1 | 203767973 | 203823256 |
| PPP1R15B | chr1 | 204372491 | 204380944 |
| ELK4 | chr1 | 205577070 | 205602000 |
| NUCKS1 | chr1 | 205681946 | 205719372 |
| CD46 | chr1 | 207925382 | 207968861 |
| PLXNA2 | chr1 | 208195587 | 208417665 |
| PTPN14 | chr1 | 214522038 | 214725024 |
| CAPN2 | chr1 | 223900118 | 223963720 |
| DEGS1 | chr1 | 224370909 | 224381142 |
| WDR26 | chr1 | 224572844 | 224622001 |
| LBR | chr1 | 225589203 | 225615815 |
| SRP9 | chr1 | 225965514 | 225978168 |
| SDE2 | chr1 | 226170402 | 226187066 |
| H3F3A | chr1 | 226250407 | 226259703 |
| H3F3AP4 | chr1 | 226250427 | 226259703 |
| PARP1 | chr1 | 226548391 | 226595801 |
| CDC42BPA | chr1 | 227177565 | 227505826 |
| ARF1 | chr1 | 228270850 | 228286913 |
| GUK1 | chr1 | 228327928 | 228336655 |
| TRIM11 | chr1 | 228581376 | 228594517 |
| EXOC8 | chr1 | 231468481 | 231473578 |
| IRF2BP2 | chr1 | 234740014 | 234745271 |
| TOMM20 | chr1 | 235272657 | 235292256 |
| SNORA14B | chr1 | 235291117 | 235291252 |
| ARID4B | chr1 | 235330209 | 235491532 |
| CEP170 | chr1 | 243287729 | 243418708 |
| ADSS | chr1 | 244571793 | 244615436 |
| HNRNPU | chr1 | 245013601 | 245027827 |
| LINC00115 | chr1 | 761585 | 762902 |
| NOC2L | chr1 | 879582 | 893918 |
| SDF4 | chr1 | 1152287 | 1167447 |
| B3GALT6 | chr1 | 1167628 | 1170420 |
| CPSF3L | chr1 | 1246964 | 1260067 |
| GLTPD1 | chr1 | 1260142 | 1264276 |
| AURKAIP1 | chr1 | 1309109 | 1310818 |
| CCNL2 | chr1 | 1321090 | 1328183 |
| LOC148413 | chr1 | 1334909 | 1337426 |
| MRPL20 | chr1 | 1340412 | 1342693 |
| ATAD3B | chr1 | 1413405 | 1431582 |
| ATAD3A | chr1 | 1447522 | 1470067 |
| CDK11B | chr1 | 1571099 | 1655775 |
| SLC35E2B | chr1 | 1592938 | 1624243 |
| CDK11A | chr1 | 1634169 | 1655791 |
| SLC35E2 | chr1 | 1658823 | 1677438 |
| NADK | chr1 | 1682670 | 1709909 |
| MORN1 | chr1 | 2263564 | 2322993 |
| LRRC47 | chr1 | 3696783 | 3713068 |
| C1orf174 | chr1 | 3805696 | 3816857 |
| LOC100133612 | chr1 | 3816967 | 3832011 |
| ICMT | chr1 | 6281252 | 6296044 |
| PLEKHG5 | chr1 | 6526151 | 6537718 |
| NOL9 | chr1 | 6581406 | 6614658 |
| PHF13 | chr1 | 6673755 | 6684093 |
| DNAJC11 | chr1 | 6694227 | 6761966 |
| CAMTA1 | chr1 | 6845383 | 7829766 |
| VAMP3 | chr1 | 7831328 | 7841492 |
| PARK7 | chr1 | 8021713 | 8045342 |
| RERE | chr1 | 8412463 | 8877699 |
| ENO1 | chr1 | 8921058 | 8931635 |
| ENO1-AS1 | chr1 | 8938893 | 8939943 |
| SLC25A33 | chr1 | 9599527 | 9642831 |
| TMEM201 | chr1 | 9648931 | 9665020 |
| NMNAT1 | chr1 | 10003485 | 10045556 |
| UBE4B | chr1 | 10093040 | 10241296 |
| PGD | chr1 | 10459084 | 10480201 |
| PEX14 | chr1 | 10535002 | 10690815 |
| SRM | chr1 | 11114648 | 11120091 |
| EXOSC10 | chr1 | 11126675 | 11159938 |
| MTHFR | chr1 | 11845786 | 11863440 |
| CLCN6 | chr1 | 11866152 | 11903201 |
| KIAA2013 | chr1 | 11979644 | 11986485 |
| PLOD1 | chr1 | 11994723 | 12035599 |
| MFN2 | chr1 | 12040237 | 12073572 |
| TNFRSF1B | chr1 | 12227059 | 12269277 |
| DHRS3 | chr1 | 12627938 | 12677820 |
| DNAJC16 | chr1 | 15853351 | 15898228 |
| PLEKHM2 | chr1 | 16010826 | 16061264 |
| FBLIM1 | chr1 | 16091029 | 16113084 |
| FLJ37453 | chr1 | 16160709 | 16174642 |
| ZBTB17 | chr1 | 16268363 | 16302627 |
| FBXO42 | chr1 | 16576558 | 16678948 |
| NECAP2 | chr1 | 16767166 | 16786584 |
| NBPF1 | chr1 | 16888921 | 16940100 |
| SDHB | chr1 | 17345224 | 17380665 |
| EMC1 | chr1 | 19542157 | 19578053 |
| MRTO4 | chr1 | 19578074 | 19586622 |
| PQLC2 | chr1 | 19638739 | 19658922 |
| MINOS1 | chr1 | 19923470 | 19956315 |
| MINOS1-NBL1 | chr1 | 19923470 | 19984949 |
| OTUD3 | chr1 | 20208887 | 20239437 |
| MUL1 | chr1 | 20825940 | 20834674 |
| DDOST | chr1 | 20978259 | 20988037 |
| EIF4G3 | chr1 | 21132784 | 21503381 |
| ECE1 | chr1 | 21543739 | 21606183 |
| LOC100506801 | chr1 | 21619782 | 21626362 |
| LINC00339 | chr1 | 22351706 | 22357715 |
| ZBTB40 | chr1 | 22778343 | 22857650 |
| EPHB2 | chr1 | 23037330 | 23241823 |
| MIR4684 | chr1 | 23046009 | 23046091 |
| ZNF436 | chr1 | 23685940 | 23696357 |
| C1orf213 | chr1 | 23695463 | 23698330 |
| LOC100506963 | chr1 | 24086871 | 24104787 |
| PITHD1 | chr1 | 24104875 | 24114722 |
| LYPLA2 | chr1 | 24117645 | 24122029 |
| SYF2 | chr1 | 25548766 | 25559013 |
| TMEM50A | chr1 | 25664788 | 25688852 |
| TMEM57 | chr1 | 25757387 | 25826698 |
| CEP85 | chr1 | 26560692 | 26605299 |
| SH3BGRL3 | chr1 | 26606212 | 26608013 |
| HMGN2 | chr1 | 26798901 | 26803133 |
| PIGV | chr1 | 27114678 | 27124894 |
| GPN2 | chr1 | 27205872 | 27216869 |
| GPATCH3 | chr1 | 27216978 | 27226962 |
| NUDC | chr1 | 27248212 | 27273362 |
| TMEM222 | chr1 | 27648635 | 27662891 |
| LOC644961 | chr1 | 27650364 | 27653016 |
| AHDC1 | chr1 | 27860755 | 27930143 |
| SCARNA1 | chr1 | 28160911 | 28161077 |
| EYA3 | chr1 | 28296854 | 28415148 |
| DNAJC8 | chr1 | 28526789 | 28559542 |
| ATPIF1 | chr1 | 28562601 | 28564616 |
| SESN2 | chr1 | 28585962 | 28609002 |
| PHACTR4 | chr1 | 28696092 | 28826881 |
| RCC1 | chr1 | 28832454 | 28865708 |
| SNORD99 | chr1 | 28905254 | 28905334 |
| SNORA61 | chr1 | 28906275 | 28906405 |
| SNORA44 | chr1 | 28906892 | 28907024 |
| SNORA16A | chr1 | 28907431 | 28907565 |
| TAF12 | chr1 | 28929608 | 28969604 |
| RNU11 | chr1 | 28975111 | 28975246 |
| GMEB1 | chr1 | 28995239 | 29042115 |
| LAPTM5 | chr1 | 31205314 | 31230683 |
| SNORD85 | chr1 | 31441009 | 31441084 |
| SNRNP40 | chr1 | 31732414 | 31769644 |
| ZCCHC17 | chr1 | 31769930 | 31837780 |
| PEF1 | chr1 | 32095462 | 32110838 |
| PTP4A2 | chr1 | 32372021 | 32385259 |
| TMEM39B | chr1 | 32538502 | 32568467 |
| TXLNA | chr1 | 32645665 | 32663886 |
| TMEM234 | chr1 | 32680077 | 32687926 |
| EIF3I | chr1 | 32687184 | 32697205 |
| HDAC1 | chr1 | 32795080 | 32799224 |
| MARCKSL1 | chr1 | 32799429 | 32801840 |
| BSDC1 | chr1 | 32830704 | 32860062 |
| ZBTB8OS | chr1 | 33087306 | 33116185 |
| RBBP4 | chr1 | 33116748 | 33151812 |
| S100PBP | chr1 | 33283118 | 33324480 |
| RNF19B | chr1 | 33402049 | 33430286 |
| AK2 | chr1 | 33473540 | 33502512 |
| ZNF362 | chr1 | 33722173 | 33766320 |
| MIR3605 | chr1 | 33797993 | 33798093 |
| SMIM12 | chr1 | 35315962 | 35324646 |
| ZMYM6NB | chr1 | 35447126 | 35450948 |
| ZMYM6 | chr1 | 35451766 | 35497569 |
| ZMYM1 | chr1 | 35544971 | 35581455 |
| ZMYM4 | chr1 | 35734567 | 35887545 |
| C1orf216 | chr1 | 36179476 | 36184790 |
| AGO3 | chr1 | 36396682 | 36522063 |
| ADPRHL2 | chr1 | 36554452 | 36559533 |
| TRAPPC3 | chr1 | 36602169 | 36615115 |
| STK40 | chr1 | 36805224 | 36851485 |
| LSM10 | chr1 | 36859030 | 36863493 |
| MRPS15 | chr1 | 36921361 | 36930040 |
| LOC728431 | chr1 | 37920479 | 37940044 |
| MEAF6 | chr1 | 37955560 | 37979867 |
| SNIP1 | chr1 | 38000049 | 38019945 |
| GNL2 | chr1 | 38032412 | 38061586 |
| C1orf109 | chr1 | 38147241 | 38156175 |
| CDCA8 | chr1 | 38158072 | 38175391 |
| YRDC | chr1 | 38268613 | 38273865 |
| C1orf122 | chr1 | 38273472 | 38275126 |
| MTF1 | chr1 | 38275238 | 38325292 |
| INPP5B | chr1 | 38326368 | 38412729 |
| SF3A3 | chr1 | 38422651 | 38455761 |
| UTP11L | chr1 | 38478383 | 38490497 |
| RRAGC | chr1 | 39303868 | 39340166 |
| AKIRIN1 | chr1 | 39456915 | 39471737 |
| NDUFS5 | chr1 | 39491966 | 39500308 |
| TRIT1 | chr1 | 40306705 | 40349177 |
| ZMPSTE24 | chr1 | 40723721 | 40759856 |
| ZNF684 | chr1 | 40997232 | 41013841 |
| LOC100130557 | chr1 | 41154751 | 41157933 |
| NFYC | chr1 | 41157679 | 41237275 |
| SLFNL1-AS1 | chr1 | 41480261 | 41509562 |
| LEPRE1 | chr1 | 43212005 | 43232755 |
| C1orf50 | chr1 | 43232915 | 43241413 |
| EBNA1BP2 | chr1 | 43629844 | 43736607 |
| WDR65 | chr1 | 43638000 | 43720029 |
| ELOVL1 | chr1 | 43829067 | 43833409 |
| MED8 | chr1 | 43849578 | 43855483 |
| SZT2 | chr1 | 43855555 | 43919918 |
| PTPRF | chr1 | 43996546 | 44089343 |
| KDM4A | chr1 | 44115796 | 44171189 |
| KDM4A-AS1 | chr1 | 44165408 | 44173012 |
| ST3GAL3 | chr1 | 44173203 | 44396837 |
| ATP6V0B | chr1 | 44440601 | 44443972 |
| B4GALT2 | chr1 | 44444873 | 44456843 |
| KIF2C | chr1 | 45212084 | 45233438 |
| SNORD46 | chr1 | 45242163 | 45242261 |
| SNORD38A | chr1 | 45243513 | 45243584 |
| SNORD38B | chr1 | 45244061 | 45244130 |
| TCTEX1D4 | chr1 | 45271585 | 45272957 |
| HECTD3 | chr1 | 45468219 | 45477027 |
| UROD | chr1 | 45477804 | 45481341 |
| MUTYH | chr1 | 45794913 | 45806142 |
| TOE1 | chr1 | 45805341 | 45809650 |
| AKR1A1 | chr1 | 46016454 | 46035723 |
| TMEM69 | chr1 | 46153846 | 46160108 |
| MAST2 | chr1 | 46269284 | 46501796 |
| PIK3R3 | chr1 | 46505811 | 46598380 |
| RAD54L | chr1 | 46713366 | 46744145 |
| UQCRH | chr1 | 46769379 | 46782447 |
| NSUN4 | chr1 | 46805848 | 46830824 |
| EFCAB14 | chr1 | 47140830 | 47184736 |
| STIL | chr1 | 47715810 | 47779819 |
| CMPK1 | chr1 | 47799468 | 47844511 |
| FAF1 | chr1 | 50906934 | 51425936 |
| CDKN2C | chr1 | 51433607 | 51440309 |
| EPS15 | chr1 | 51819934 | 51984995 |
| OSBPL9 | chr1 | 52225295 | 52254891 |
| NRD1 | chr1 | 52254865 | 52343712 |
| TXNDC12 | chr1 | 52497768 | 52521843 |
| KTI12 | chr1 | 52497776 | 52499472 |
| BTF3L4 | chr1 | 52521856 | 52556388 |
| ZFYVE9 | chr1 | 52607765 | 52812358 |
| CC2D1B | chr1 | 52816264 | 52831877 |
| ORC1 | chr1 | 52838500 | 52870143 |
| PRPF38A | chr1 | 52870218 | 52883992 |
| SELRC1 | chr1 | 53152013 | 53164038 |
| ZYG11B | chr1 | 53192130 | 53293013 |
| MAGOH | chr1 | 53692563 | 53704282 |
| LOC100507564 | chr1 | 53704281 | 53708455 |
| LRP8 | chr1 | 53708040 | 53793821 |
| NDC1 | chr1 | 54231133 | 54304225 |
| LRRC42 | chr1 | 54411998 | 54433841 |
| TMEM59 | chr1 | 54497348 | 54510014 |
| TCEANC2 | chr1 | 54519273 | 54565416 |
| MIR4781 | chr1 | 54519751 | 54519827 |
| CYB5RL | chr1 | 54638026 | 54665746 |
| MRPL37 | chr1 | 54665839 | 54684056 |
| SSBP3 | chr1 | 54691103 | 54872068 |
| TTC4 | chr1 | 55181494 | 55208328 |
| USP24 | chr1 | 55532031 | 55681039 |
| PPAP2B | chr1 | 56960418 | 57045257 |
| TACSTD2 | chr1 | 59041094 | 59043166 |
| MYSM1 | chr1 | 59120410 | 59165747 |
| LOC100131060 | chr1 | 59250822 | 59365384 |
| NFIA | chr1 | 61547979 | 61928460 |
| TM2D1 | chr1 | 62146718 | 62191095 |
| DOCK7 | chr1 | 62920396 | 63154039 |
| ITGB3BP | chr1 | 63919111 | 63988812 |
| EFCAB7 | chr1 | 63989012 | 64038364 |
| WDR78 | chr1 | 67278571 | 67390570 |
| MIER1 | chr1 | 67390577 | 67454302 |
| GADD45A | chr1 | 68150859 | 68154021 |
| GNG12-AS1 | chr1 | 68297970 | 68668670 |
| WLS | chr1 | 68591040 | 68698284 |
| DEPDC1 | chr1 | 68939834 | 68962799 |
| LRRC40 | chr1 | 70610484 | 70671361 |
| ANKRD13C | chr1 | 70724684 | 70820417 |
| HHLA3 | chr1 | 70820492 | 70833705 |
| ZRANB2-AS2 | chr1 | 71547006 | 71703406 |
| NEGR1 | chr1 | 71868624 | 72748405 |
| CRYZ | chr1 | 75171171 | 75199092 |
| TYW3 | chr1 | 75198861 | 75232360 |
| ACADM | chr1 | 76190042 | 76229355 |
| SNORD45B | chr1 | 76255161 | 76255232 |
| ST6GALNAC3 | chr1 | 76540388 | 77096669 |
| USP33 | chr1 | 78161673 | 78225564 |
| FAM73A | chr1 | 78245308 | 78345225 |
| NEXN | chr1 | 78354199 | 78409578 |
| PRKACB | chr1 | 84609951 | 84704181 |
| SAMD13 | chr1 | 84767288 | 84816481 |
| RPF1 | chr1 | 84944919 | 84964033 |
| CTBS | chr1 | 85018803 | 85040163 |
| C1orf52 | chr1 | 85715636 | 85725355 |
| LOC646626 | chr1 | 85742040 | 85743771 |
| ZNHIT6 | chr1 | 86115105 | 86174116 |
| ODF2L | chr1 | 86815776 | 86862025 |
| HS2ST1 | chr1 | 87380334 | 87575681 |
| LOC339524 | chr1 | 87458689 | 87634886 |
| PKN2 | chr1 | 89149921 | 89301938 |
| GTF2B | chr1 | 89318320 | 89357301 |
| CCBL2 | chr1 | 89401455 | 89458643 |
| RBMXL1 | chr1 | 89445138 | 89458643 |
| GBP3 | chr1 | 89472359 | 89488549 |
| FLJ27354 | chr1 | 90090407 | 90098453 |
| GEMIN8P4 | chr1 | 90458823 | 90460525 |
| ZNF326 | chr1 | 90460677 | 90494094 |
| ZNF644 | chr1 | 91380856 | 91487812 |
| CDC7 | chr1 | 91966664 | 91991321 |
| GLMN | chr1 | 92711954 | 92764566 |
| RPAP2 | chr1 | 92764521 | 92853732 |
| SNORD21 | chr1 | 93302845 | 93302940 |
| SNORA66 | chr1 | 93306275 | 93306408 |
| CCDC18 | chr1 | 93646280 | 93744287 |
| LOC100131564 | chr1 | 93775665 | 93811368 |
| FNBP1L | chr1 | 93913687 | 94020218 |
| BCAR3 | chr1 | 94027342 | 94312706 |
| LOC100129046 | chr1 | 94057524 | 94065587 |
| MIR760 | chr1 | 94312387 | 94312467 |
| DNTTIP2 | chr1 | 94335013 | 94344762 |
| LOC729970 | chr1 | 95393583 | 95428826 |
| PTBP2 | chr1 | 97187174 | 97280605 |
| LOC729987 | chr1 | 98676266 | 98738214 |
| MIR548AA1 | chr1 | 100178485 | 100178513 |
| SASS6 | chr1 | 100549101 | 100598511 |
| TRMT13 | chr1 | 100598705 | 100616054 |
| RTCA | chr1 | 100731713 | 100758325 |
| CDC14A | chr1 | 100818022 | 100985833 |
| EXTL2 | chr1 | 101337927 | 101360735 |
| SLC30A7 | chr1 | 101361631 | 101447311 |
| DPH5 | chr1 | 101455179 | 101491362 |
| RNPC3 | chr1 | 104068577 | 104096306 |
| SLC25A24 | chr1 | 108677343 | 108735431 |
| STXBP3 | chr1 | 109289284 | 109352148 |
| CLCC1 | chr1 | 109472129 | 109506121 |
| TAF13 | chr1 | 109606997 | 109618624 |
| TMEM167B | chr1 | 109633402 | 109639554 |
| SCARNA2 | chr1 | 109642814 | 109643234 |
| PSMA5 | chr1 | 109941652 | 109969070 |
| AMPD2 | chr1 | 110163535 | 110174677 |
| AHCYL1 | chr1 | 110527386 | 110566364 |
| STRIP1 | chr1 | 110577222 | 110597424 |
| LOC440600 | chr1 | 110828998 | 110881793 |
| LAMTOR5 | chr1 | 110943876 | 110950546 |
| LRIF1 | chr1 | 111489811 | 111506566 |
| DRAM2 | chr1 | 111659953 | 111682838 |
| CEPT1 | chr1 | 111682832 | 111727724 |
| WDR77 | chr1 | 111982511 | 111991830 |
| ATP5F1 | chr1 | 111991742 | 112004525 |
| RAP1A | chr1 | 112162404 | 112256101 |
| FAM212B | chr1 | 112264685 | 112298419 |
| DDX20 | chr1 | 112298189 | 112310199 |
| ST7L | chr1 | 113066140 | 113162040 |
| MOV10 | chr1 | 113217047 | 113243368 |
| LRIG2 | chr1 | 113615830 | 113667342 |
| PHTF1 | chr1 | 114239823 | 114257834 |
| RSBN1 | chr1 | 114304453 | 114355070 |
| AP4B1 | chr1 | 114437370 | 114447741 |
| DCLRE1B | chr1 | 114447914 | 114456708 |
| BCAS2 | chr1 | 115110180 | 115124265 |
| NRAS | chr1 | 115247084 | 115259515 |
| VANGL1 | chr1 | 116184573 | 116240845 |
| ATP1A1 | chr1 | 116925991 | 116947396 |
| MIR942 | chr1 | 117637264 | 117637350 |
| MAN1A2 | chr1 | 117910084 | 118068320 |
| GDAP2 | chr1 | 118406106 | 118472302 |
| WDR3 | chr1 | 118472371 | 118503049 |
| WARS2 | chr1 | 119573838 | 119683295 |
| NOTCH2 | chr1 | 120454175 | 120612317 |
| FAM72B | chr1 | 120839004 | 120855681 |
| FAM72D | chr1 | 143896451 | 143913143 |
| RBM8A | chr1 | 145507556 | 145513535 |
| POLR3C | chr1 | 145592604 | 145610884 |
| RNF115 | chr1 | 145611035 | 145688776 |
| PRKAB2 | chr1 | 146626684 | 146644129 |
| CHD1L | chr1 | 146714290 | 146767447 |
| GPR89B | chr1 | 147400505 | 147465755 |
| NBPF15 | chr1 | 148558187 | 148596267 |
| LOC645166 | chr1 | 148930404 | 148953054 |
| HIST2H4A | chr1 | 149804220 | 149804616 |
| HIST2H4B | chr1 | 149804220 | 149804616 |
| HIST2H3C | chr1 | 149812258 | 149812765 |
| HIST2H3A | chr1 | 149812258 | 149812765 |
| HIST2H2AA4 | chr1 | 149813784 | 149814318 |
| HIST2H2BC | chr1 | 149821758 | 149822340 |
| HIST2H2BE | chr1 | 149856009 | 149858232 |
| HIST2H2AC | chr1 | 149858524 | 149858961 |
| HIST2H2AB | chr1 | 149859018 | 149859466 |
| SF3B4 | chr1 | 149895208 | 149900144 |
| OTUD7B | chr1 | 149912231 | 149982686 |
| VPS45 | chr1 | 150039341 | 150117505 |
| PLEKHO1 | chr1 | 150127086 | 150131825 |
| ANP32E | chr1 | 150190716 | 150208504 |
| APH1A | chr1 | 150237798 | 150241609 |
| MRPS21 | chr1 | 150266268 | 150280819 |
| PRPF3 | chr1 | 150293927 | 150325704 |
| ENSA | chr1 | 150595753 | 150602098 |
| GOLPH3L | chr1 | 150618700 | 150669672 |
| ARNT | chr1 | 150782180 | 150849244 |
| SETDB1 | chr1 | 150898814 | 150937220 |
| FAM63A | chr1 | 150969300 | 150980225 |
| PRUNE | chr1 | 150980972 | 151008189 |
| GABPB2 | chr1 | 151043079 | 151091007 |
| LYSMD1 | chr1 | 151132223 | 151138370 |
| SCNM1 | chr1 | 151138497 | 151142773 |
| VPS72 | chr1 | 151148775 | 151162689 |
| PSMD4 | chr1 | 151227196 | 151239954 |
| ZNF687 | chr1 | 151254790 | 151264381 |
| PI4KB | chr1 | 151264272 | 151300191 |
| RFX5 | chr1 | 151313115 | 151319769 |
| PSMB4 | chr1 | 151372040 | 151374412 |
| POGZ | chr1 | 151375199 | 151431941 |
| MRPL9 | chr1 | 151732122 | 151736040 |
| OAZ3 | chr1 | 151735444 | 151743806 |
| S100A11 | chr1 | 152004981 | 152009511 |
| S100A6 | chr1 | 153507075 | 153508717 |
| S100A2 | chr1 | 153533584 | 153538306 |
| S100A13 | chr1 | 153591275 | 153606568 |
| CHTOP | chr1 | 153609047 | 153618782 |
| INTS3 | chr1 | 153700566 | 153746555 |
| DENND4B | chr1 | 153901976 | 153919154 |
| CRTC2 | chr1 | 153920147 | 153931132 |
| SLC39A1 | chr1 | 153931574 | 153935968 |
| JTB | chr1 | 153946744 | 153950451 |
| RAB13 | chr1 | 153954092 | 153957842 |
| RPS27 | chr1 | 153963238 | 153964631 |
| HAX1 | chr1 | 154245038 | 154248355 |
| TDRD10 | chr1 | 154474694 | 154520623 |
| ADAR | chr1 | 154554533 | 154578776 |
| PMVK | chr1 | 154897207 | 154909484 |
| PBXIP1 | chr1 | 154916558 | 154928567 |
| PYGO2 | chr1 | 154929501 | 154934258 |
| MIR4258 | chr1 | 154948168 | 154948259 |
| FLAD1 | chr1 | 154955769 | 154963682 |
| ZBTB7B | chr1 | 154975105 | 154991001 |
| ADAM15 | chr1 | 155023747 | 155035252 |
| EFNA1 | chr1 | 155100348 | 155107386 |
| SLC50A1 | chr1 | 155108287 | 155111334 |
| DPM3 | chr1 | 155112366 | 155112883 |
| KRTCAP2 | chr1 | 155143876 | 155145804 |
| GBAP1 | chr1 | 155183615 | 155197325 |
| SCAMP3 | chr1 | 155225769 | 155232176 |
| CLK2 | chr1 | 155232658 | 155243281 |
| ASH1L-AS1 | chr1 | 155531771 | 155533735 |
| DAP3 | chr1 | 155658881 | 155708800 |
| GON4L | chr1 | 155734575 | 155827008 |
| SYT11 | chr1 | 155829259 | 155854990 |
| RIT1 | chr1 | 155867598 | 155881193 |
| SSR2 | chr1 | 155978838 | 155990758 |
| UBQLN4 | chr1 | 156005091 | 156023516 |
| LAMTOR2 | chr1 | 156024516 | 156028605 |
| MEX3A | chr1 | 156041803 | 156051789 |
| SLC25A44 | chr1 | 156163729 | 156182587 |
| PMF1 | chr1 | 156182778 | 156209868 |
| PMF1-BGLAP | chr1 | 156182778 | 156213123 |
| TMEM79 | chr1 | 156252703 | 156262234 |
| TSACC | chr1 | 156307104 | 156316785 |
| MEF2D | chr1 | 156433512 | 156470634 |
| IQGAP3 | chr1 | 156495196 | 156542396 |
| GPATCH4 | chr1 | 156564099 | 156571279 |
| NES | chr1 | 156638555 | 156647189 |
| RRNAD1 | chr1 | 156698262 | 156706752 |
| MRPL24 | chr1 | 156707093 | 156710923 |
| ARHGEF11 | chr1 | 156904631 | 157015162 |
| ETV3 | chr1 | 157094458 | 157108383 |
| KIRREL | chr1 | 157963062 | 158065844 |
| IFI16 | chr1 | 158979681 | 159024945 |
| DUSP23 | chr1 | 159750807 | 159752333 |
| DCAF8 | chr1 | 160185504 | 160254941 |
| PEX19 | chr1 | 160246598 | 160254941 |
| NCSTN | chr1 | 160313062 | 160328742 |
| F11R | chr1 | 160965000 | 161008774 |
| USF1 | chr1 | 161009040 | 161015769 |
| NIT1 | chr1 | 161087861 | 161095235 |
| DEDD | chr1 | 161090768 | 161102478 |
| UFC1 | chr1 | 161123533 | 161128646 |
| ADAMTS4 | chr1 | 161159537 | 161168845 |
| NDUFS2 | chr1 | 161171936 | 161184184 |
| SDHC | chr1 | 161284165 | 161334535 |
| DUSP12 | chr1 | 161719580 | 161726952 |
| UHMK1 | chr1 | 162467594 | 162499419 |
| DDR2 | chr1 | 162602227 | 162750247 |
| RGS4 | chr1 | 163038395 | 163046592 |
| RGS5 | chr1 | 163112088 | 163172963 |
| NUF2 | chr1 | 163291722 | 163325553 |
| MGST3 | chr1 | 165600109 | 165625372 |
| TMCO1 | chr1 | 165693527 | 165738159 |
| POGK | chr1 | 166808723 | 166823709 |
| POU2F1 | chr1 | 167190122 | 167396582 |
| MPZL1 | chr1 | 167734294 | 167761156 |
| MPC2 | chr1 | 167885912 | 167905439 |
| DCAF6 | chr1 | 167905796 | 168045083 |
| TIPRL | chr1 | 168148170 | 168171351 |
| SFT2D2 | chr1 | 168195254 | 168212088 |
| NME7 | chr1 | 169252459 | 169337186 |
| BLZF1 | chr1 | 169337193 | 169357539 |
| SLC19A2 | chr1 | 169433148 | 169455208 |
| METTL18 | chr1 | 169761669 | 169764061 |
| C1orf112 | chr1 | 169764549 | 169822229 |
| SCYL3 | chr1 | 169822214 | 169863100 |
| GORAB | chr1 | 170501262 | 170522974 |
| VAMP4 | chr1 | 171669295 | 171711379 |
| METTL13 | chr1 | 171750760 | 171766856 |
| DNM3OS | chr1 | 172106018 | 172113975 |
| PIGC | chr1 | 172410596 | 172413230 |
| SUCO | chr1 | 172502259 | 172580973 |
| TNFSF18 | chr1 | 173010359 | 173020103 |
| LOC100506023 | chr1 | 173204198 | 173446294 |
| PRDX6 | chr1 | 173446485 | 173457946 |
| KLHL20 | chr1 | 173684079 | 173755840 |
| CENPL | chr1 | 173768687 | 173793777 |
| DARS2 | chr1 | 173793796 | 173827682 |
| SNORD81 | chr1 | 173833312 | 173833355 |
| SNORD47 | chr1 | 173833506 | 173833583 |
| SNORD80 | chr1 | 173833970 | 173834041 |
| SNORD79 | chr1 | 173834487 | 173834568 |
| SNORD78 | chr1 | 173834770 | 173834824 |
| SNORD44 | chr1 | 173835105 | 173835166 |
| SNORD77 | chr1 | 173835448 | 173835509 |
| SNORD76 | chr1 | 173835772 | 173835853 |
| SNORD75 | chr1 | 173836016 | 173836076 |
| ZBTB37 | chr1 | 173837219 | 173855774 |
| MRPS14 | chr1 | 174982093 | 174992591 |
| RASAL2 | chr1 | 178062863 | 178448648 |
| FAM20B | chr1 | 178995073 | 179045702 |
| ABL2 | chr1 | 179068461 | 179198819 |
| TOR1AIP2 | chr1 | 179809101 | 179846941 |
| TOR1AIP1 | chr1 | 179851176 | 179889212 |
| CEP350 | chr1 | 179923907 | 180084015 |
| QSOX1 | chr1 | 180123967 | 180167169 |
| ACBD6 | chr1 | 180257351 | 180472022 |
| STX6 | chr1 | 180942175 | 180992046 |
| GLUL | chr1 | 182350838 | 182360924 |
| SMG7-AS1 | chr1 | 183430010 | 183441117 |
| SMG7 | chr1 | 183441505 | 183523328 |
| ARPC5 | chr1 | 183595327 | 183605076 |
| RGL1 | chr1 | 183605207 | 183897666 |
| EDEM3 | chr1 | 184659624 | 184724041 |
| RNF2 | chr1 | 185014550 | 185071740 |
| TRMT1L | chr1 | 185087217 | 185126116 |
| SWT1 | chr1 | 185126290 | 185260913 |
| MIR548F1 | chr1 | 186029866 | 186446655 |
| TPR | chr1 | 186280785 | 186344457 |
| C1orf27 | chr1 | 186344889 | 186390503 |
| PTGS2 | chr1 | 186640943 | 186649559 |
| RGS2 | chr1 | 192778168 | 192781407 |
| UCHL5 | chr1 | 192981495 | 193028523 |
| TROVE2 | chr1 | 193028551 | 193055115 |
| GLRX2 | chr1 | 193065594 | 193074608 |
| CDC73 | chr1 | 193091087 | 193223942 |
| ASPM | chr1 | 197053256 | 197115824 |
| ZBTB41 | chr1 | 197122813 | 197169672 |
| KIF14 | chr1 | 200520624 | 200589862 |
| DDX59 | chr1 | 200613164 | 200639126 |
| PHLDA3 | chr1 | 201434606 | 201438299 |
| CSRP1 | chr1 | 201452657 | 201465701 |
| NAV1 | chr1 | 201617449 | 201796102 |
| TIMM17A | chr1 | 201924618 | 201939789 |
| ARL8A | chr1 | 202102531 | 202113871 |
| UBE2T | chr1 | 202300784 | 202311094 |
| KDM5B | chr1 | 202696531 | 202777549 |
| ADIPOR1 | chr1 | 202909959 | 202927524 |
| TMEM183A | chr1 | 202976533 | 202993197 |
| ATP2B4 | chr1 | 203595914 | 203713209 |
| SNRPE | chr1 | 203830739 | 203840280 |
| SOX13 | chr1 | 204042245 | 204096871 |
| MDM4 | chr1 | 204485506 | 204527248 |
| RBBP5 | chr1 | 205055269 | 205085416 |
| FAM72A | chr1 | 206138910 | 206155074 |
| EIF2D | chr1 | 206764973 | 206785904 |
| MAPKAPK2 | chr1 | 206858364 | 206907630 |
| YOD1 | chr1 | 207217193 | 207224422 |
| CD55 | chr1 | 207494816 | 207534311 |
| LOC148696 | chr1 | 207991723 | 207995941 |
| DIEXF | chr1 | 210001311 | 210030910 |
| SLC30A1 | chr1 | 211748380 | 211752099 |
| INTS7 | chr1 | 212113740 | 212209002 |
| DTL | chr1 | 212208918 | 212278187 |
| PPP2R5A | chr1 | 212458878 | 212535205 |
| FLVCR1-AS1 | chr1 | 213029945 | 213031480 |
| FLVCR1 | chr1 | 213031596 | 213072705 |
| ANGEL2 | chr1 | 213165523 | 213189168 |
| CENPF | chr1 | 214776531 | 214837914 |
| KCTD3 | chr1 | 215740734 | 215795149 |
| GPATCH2 | chr1 | 217781534 | 217804409 |
| SPATA17 | chr1 | 217804694 | 218040484 |
| RRP15 | chr1 | 218458628 | 218511325 |
| LYPLAL1 | chr1 | 219347191 | 219386207 |
| EPRS | chr1 | 220141941 | 220220000 |
| RAB3GAP2 | chr1 | 220321609 | 220445843 |
| AIDA | chr1 | 222841354 | 222886526 |
| BROX | chr1 | 222885905 | 222906106 |
| TP53BP2 | chr1 | 223967594 | 224033674 |
| FBXO28 | chr1 | 224301788 | 224349749 |
| NVL | chr1 | 224415035 | 224517891 |
| CNIH4 | chr1 | 224544512 | 224566223 |
| ENAH | chr1 | 225674533 | 225840845 |
| LEFTY1 | chr1 | 226073981 | 226112040 |
| PYCR2 | chr1 | 226107576 | 226112040 |
| H3F3A | chr1 | 226250407 | 226254301 |
| H3F3AP4 | chr1 | 226251677 | 226259703 |
| ACBD3 | chr1 | 226332379 | 226374423 |
| LIN9 | chr1 | 226418849 | 226497204 |
| ITPKB | chr1 | 226819390 | 226926876 |
| ZNF678 | chr1 | 227751219 | 227850164 |
| JMJD4 | chr1 | 227918889 | 227923112 |
| SNAP47 | chr1 | 227922696 | 227964671 |
| MIR3620 | chr1 | 228284963 | 228285042 |
| GUK1 | chr1 | 228332403 | 228336655 |
| IBA57 | chr1 | 228353428 | 228369958 |
| RAB4A | chr1 | 229406808 | 229441640 |
| NUP133 | chr1 | 229577043 | 229644088 |
| ABCB10 | chr1 | 229652328 | 229694442 |
| TAF5L | chr1 | 229734940 | 229761794 |
| URB2 | chr1 | 229761980 | 229795946 |
| GALNT2 | chr1 | 230193535 | 230417875 |
| COG2 | chr1 | 230778201 | 230829731 |
| C1orf131 | chr1 | 231372441 | 231376924 |
| GNPAT | chr1 | 231376918 | 231413719 |
| SPRTN | chr1 | 231473681 | 231490769 |
| TSNAX-DISC1 | chr1 | 231664398 | 232177019 |
| TSNAX | chr1 | 231664398 | 231702269 |
| COA6 | chr1 | 234509213 | 234519795 |
| LOC100506810 | chr1 | 234859788 | 234867390 |
| RBM34 | chr1 | 235294497 | 235324772 |
| GGPS1 | chr1 | 235491868 | 235507844 |
| B3GALNT2 | chr1 | 235610504 | 235667781 |
| LGALS8 | chr1 | 236687026 | 236716279 |
| HEATR1 | chr1 | 236712304 | 236767841 |
| MTR | chr1 | 236958580 | 237067281 |
| FH | chr1 | 241660856 | 241683085 |
| OPN3 | chr1 | 241756451 | 241803701 |
| CHML | chr1 | 241792166 | 241799232 |
| EXO1 | chr1 | 242011492 | 242053241 |
| SDCCAG8 | chr1 | 243419306 | 243663393 |
| AKT3 | chr1 | 243663020 | 244006584 |
| ZBTB18 | chr1 | 244216506 | 244220780 |
| DESI2 | chr1 | 244816351 | 244872334 |
| COX20 | chr1 | 244998638 | 245008359 |
| EFCAB2 | chr1 | 245133283 | 245288530 |
| TFB2M | chr1 | 246703862 | 246729565 |
| CNST | chr1 | 246729638 | 246831884 |
| AHCTF1 | chr1 | 247002401 | 247094726 |
| ZNF670-ZNF695 | chr1 | 247108848 | 247242115 |
| ZNF670 | chr1 | 247197939 | 247242115 |
| ZNF124 | chr1 | 247285276 | 247335318 |
| ZNF496 | chr1 | 247463621 | 247495045 |
| SH3BP5L | chr1 | 249104650 | 249120154 |
| MIR3124 | chr1 | 249120575 | 249120642 |
| ZNF672 | chr1 | 249132529 | 249143714 |
| LARP4B | chr10 | 852853 | 977645 |
| GTPBP4 | chr10 | 1034654 | 1063708 |
| IDI1 | chr10 | 1085963 | 1091076 |
| PFKP | chr10 | 3155563 | 3178997 |
| KLF6 | chr10 | 3818187 | 3827473 |
| LINC00704 | chr10 | 4692376 | 4720262 |
| GDI2 | chr10 | 5807185 | 5855512 |
| RBM17 | chr10 | 6131308 | 6159422 |
| ATP5C1 | chr10 | 7830092 | 7849762 |
| GATA3 | chr10 | 8096666 | 8117164 |
| NMT2 | chr10 | 15147770 | 15210695 |
| FAM171A1 | chr10 | 15253643 | 15413058 |
| VIM | chr10 | 17270257 | 17279592 |
| STAM | chr10 | 17686123 | 17758821 |
| ARL5B | chr10 | 18948312 | 18966940 |
| ARHGAP21 | chr10 | 24872537 | 25012597 |
| YME1L1 | chr10 | 27399039 | 27443349 |
| RAB18 | chr10 | 27793102 | 27831166 |
| WAC | chr10 | 28821421 | 28912041 |
| MTPAP | chr10 | 30598729 | 30663377 |
| ZEB1 | chr10 | 31607423 | 31818742 |
| KIF5B | chr10 | 32297937 | 32345371 |
| EPC1 | chr10 | 32556643 | 32636146 |
| ITGB1 | chr10 | 33189245 | 33247293 |
| NRP1 | chr10 | 33466418 | 33623833 |
| CCNY | chr10 | 35625801 | 35860847 |
| HNRNPF | chr10 | 43881064 | 43892279 |
| ARHGAP22 | chr10 | 49654067 | 49813176 |
| ERCC6 | chr10 | 50662525 | 50714526 |
| TIMM23 | chr10 | 51371394 | 51734610 |
| NCOA4 | chr10 | 51565107 | 51590734 |
| DKK1 | chr10 | 54074040 | 54077417 |
| CDK1 | chr10 | 62538088 | 62547988 |
| HERC4 | chr10 | 69681655 | 69832943 |
| HNRNPH3 | chr10 | 70091767 | 70102953 |
| DDX21 | chr10 | 70716195 | 70744825 |
| SRGN | chr10 | 70847827 | 70864567 |
| HK1 | chr10 | 71078602 | 71161637 |
| SAR1A | chr10 | 71909960 | 71930285 |
| PSAP | chr10 | 73576054 | 73611082 |
| VCL | chr10 | 75757871 | 75879914 |
| VDAC2 | chr10 | 76970299 | 76991207 |
| RPS24 | chr10 | 79793517 | 79816571 |
| ANXA11 | chr10 | 81914879 | 81964247 |
| FAM213A | chr10 | 82168241 | 82192753 |
| TSPAN14 | chr10 | 82214037 | 82282391 |
| WAPAL | chr10 | 88195012 | 88281541 |
| MMRN2 | chr10 | 88695297 | 88717425 |
| PTEN | chr10 | 89623194 | 89728532 |
| KIF20B | chr10 | 91461366 | 91534700 |
| ANKRD1 | chr10 | 92671856 | 92681032 |
| KIF11 | chr10 | 94352824 | 94415152 |
| HHEX | chr10 | 94449680 | 94455408 |
| EXOC6 | chr10 | 94590934 | 94819251 |
| MYOF | chr10 | 95066185 | 95242074 |
| TM9SF3 | chr10 | 98277866 | 98346809 |
| PGAM1 | chr10 | 99187433 | 99193198 |
| MMS19 | chr10 | 99218080 | 99258366 |
| DNMBP | chr10 | 101635333 | 101769676 |
| DNMBP-AS1 | chr10 | 101686965 | 101718755 |
| SCD | chr10 | 102106771 | 102124588 |
| MGEA5 | chr10 | 103544199 | 103578222 |
| PPRC1 | chr10 | 103892786 | 103910090 |
| NOLC1 | chr10 | 103911932 | 103923627 |
| GBF1 | chr10 | 104005254 | 104142656 |
| ACTR1A | chr10 | 104238985 | 104262512 |
| TRIM8 | chr10 | 104404251 | 104418076 |
| NT5C2 | chr10 | 104847773 | 104913384 |
| ITPRIP | chr10 | 106069453 | 106093663 |
| SMNDC1 | chr10 | 112052797 | 112064707 |
| DUSP5 | chr10 | 112257624 | 112271302 |
| FAM160B1 | chr10 | 116581502 | 116624474 |
| EIF3A | chr10 | 120794540 | 120840334 |
| TIAL1 | chr10 | 121332977 | 121356541 |
| BAG3 | chr10 | 121410881 | 121437329 |
| MCMBP | chr10 | 121588915 | 121632395 |
| SEC23IP | chr10 | 121652084 | 121704170 |
| BUB3 | chr10 | 124913759 | 124924881 |
| CTBP2 | chr10 | 126676417 | 126849624 |
| MKI67 | chr10 | 129894924 | 129924468 |
| ZMYND11 | chr10 | 180404 | 300577 |
| WDR37 | chr10 | 1102775 | 1178237 |
| PITRM1 | chr10 | 3179918 | 3215033 |
| NET1 | chr10 | 5454513 | 5501019 |
| FAM208B | chr10 | 5726800 | 5805703 |
| ANKRD16 | chr10 | 5903688 | 5931860 |
| FBXO18 | chr10 | 5936348 | 5979558 |
| PFKFB3 | chr10 | 6188278 | 6277507 |
| KIN | chr10 | 7792924 | 7829990 |
| GATA3-AS1 | chr10 | 8092412 | 8095447 |
| USP6NL | chr10 | 11502508 | 11653679 |
| UPF2 | chr10 | 11962020 | 12077896 |
| NUDT5 | chr10 | 12209572 | 12238143 |
| CDC123 | chr10 | 12237960 | 12292589 |
| OPTN | chr10 | 13142081 | 13180276 |
| MCM10 | chr10 | 13203553 | 13253104 |
| SEPHS1 | chr10 | 13359437 | 13390298 |
| CDNF | chr10 | 14861250 | 14880574 |
| HSPA14 | chr10 | 14880158 | 14913740 |
| SUV39H2 | chr10 | 14920781 | 14946304 |
| DCLRE1C | chr10 | 14948870 | 14996094 |
| RPP38 | chr10 | 15139178 | 15146256 |
| PPIAP30 | chr10 | 15196720 | 15197346 |
| RSU1 | chr10 | 16632616 | 16859382 |
| TRDMT1 | chr10 | 17184981 | 17244070 |
| SKIDA1 | chr10 | 21802408 | 21814611 |
| MLLT10 | chr10 | 21823100 | 22032559 |
| PIP4K2A | chr10 | 22823765 | 23003503 |
| PDSS1 | chr10 | 26986594 | 27035726 |
| ABI1 | chr10 | 27035524 | 27150016 |
| MASTL | chr10 | 27443752 | 27475848 |
| ACBD5 | chr10 | 27484142 | 27529808 |
| WAC-AS1 | chr10 | 28808845 | 28821283 |
| MIR5586 | chr10 | 28835324 | 28835346 |
| PTCHD3P1 | chr10 | 29698500 | 29711299 |
| SVIL | chr10 | 29746276 | 29923901 |
| KIAA1462 | chr10 | 30301728 | 30348488 |
| ZNF438 | chr10 | 31133564 | 31288446 |
| ZEB1-AS1 | chr10 | 31605456 | 31608024 |
| ARHGAP12 | chr10 | 32094325 | 32217804 |
| CCDC7 | chr10 | 32735040 | 32863492 |
| ITGB1 | chr10 | 33189245 | 33224486 |
| CUL2 | chr10 | 35297478 | 35379570 |
| CREM | chr10 | 35415768 | 35501886 |
| BMS1 | chr10 | 43277953 | 43330385 |
| CSGALNACT2 | chr10 | 43633892 | 43680754 |
| C10orf10 | chr10 | 45471708 | 45474330 |
| C10orf25 | chr10 | 45493145 | 45496470 |
| ZNF22 | chr10 | 45496272 | 45500777 |
| PGBD3 | chr10 | 50723378 | 50725160 |
| PARG | chr10 | 51026324 | 51371331 |
| LOC728407 | chr10 | 51253907 | 51371316 |
| NCOA4 | chr10 | 51576284 | 51590734 |
| SGMS1 | chr10 | 52065344 | 52383737 |
| CSTF2T | chr10 | 53455245 | 53459355 |
| PRKG1-AS1 | chr10 | 54056607 | 54073888 |
| ZWINT | chr10 | 58117198 | 58121034 |
| IPMK | chr10 | 59951277 | 60027694 |
| CISD1 | chr10 | 60028861 | 60049019 |
| TFAM | chr10 | 60144902 | 60158990 |
| CCDC6 | chr10 | 61548505 | 61666414 |
| RHOBTB1 | chr10 | 62629197 | 62704033 |
| ARID5B | chr10 | 63661012 | 63856707 |
| ADO | chr10 | 64564515 | 64568239 |
| NRBF2 | chr10 | 64893006 | 64914786 |
| JMJD1C | chr10 | 64926987 | 65225722 |
| JMJD1C-AS1 | chr10 | 65224988 | 65226322 |
| REEP3 | chr10 | 65281122 | 65384883 |
| SIRT1 | chr10 | 69644426 | 69678147 |
| PBLD | chr10 | 70042416 | 70092684 |
| RUFY2 | chr10 | 70103274 | 70166946 |
| DNA2 | chr10 | 70173820 | 70231730 |
| CCAR1 | chr10 | 70480970 | 70551309 |
| DDX50 | chr10 | 70661033 | 70706603 |
| KIAA1279 | chr10 | 70748476 | 70776739 |
| VPS26A | chr10 | 70883907 | 70932616 |
| SUPV3L1 | chr10 | 70939992 | 70968849 |
| HK1 | chr10 | 71075609 | 71161637 |
| COL13A1 | chr10 | 71561643 | 71718904 |
| PPA1 | chr10 | 71962585 | 71993190 |
| EIF4EBP2 | chr10 | 72163860 | 72188374 |
| CHST3 | chr10 | 73724119 | 73773322 |
| ASCC1 | chr10 | 73855789 | 73976199 |
| ANAPC16 | chr10 | 73975757 | 73995618 |
| DDIT4 | chr10 | 74033676 | 74035797 |
| DNAJB12 | chr10 | 74092587 | 74114907 |
| MCU | chr10 | 74452376 | 74647452 |
| P4HA1 | chr10 | 74766979 | 74856732 |
| ECD | chr10 | 74894281 | 74927853 |
| FAM149B1 | chr10 | 74927876 | 75001939 |
| DNAJC9 | chr10 | 75002581 | 75008620 |
| DNAJC9-AS1 | chr10 | 75007124 | 75036742 |
| MRPS16 | chr10 | 75008600 | 75012451 |
| ANXA7 | chr10 | 75135188 | 75173841 |
| PPP3CB | chr10 | 75196562 | 75255782 |
| BMS1P4 | chr10 | 75458908 | 75490272 |
| GLUD1P3 | chr10 | 75491298 | 75495367 |
| SEC24C | chr10 | 75504130 | 75531933 |
| FUT11 | chr10 | 75532048 | 75535976 |
| CHCHD1 | chr10 | 75541807 | 75543406 |
| ZSWIM8 | chr10 | 75545381 | 75561556 |
| AP3M1 | chr10 | 75880014 | 75910826 |
| ADK | chr10 | 75936246 | 76469061 |
| SAMD8 | chr10 | 76871392 | 76941881 |
| POLR3A | chr10 | 79734906 | 79789298 |
| ZMIZ1 | chr10 | 80828791 | 81076285 |
| PPIF | chr10 | 81107219 | 81115089 |
| LOC642361 | chr10 | 81585657 | 81587358 |
| GHITM | chr10 | 85899184 | 85913311 |
| CCSER2 | chr10 | 86088409 | 86278276 |
| SNCG | chr10 | 88718287 | 88723017 |
| GLUD1 | chr10 | 88809958 | 88854776 |
| FAM35A | chr10 | 88854952 | 88951222 |
| NUTM2A-AS1 | chr10 | 88998423 | 89102315 |
| LOC439994 | chr10 | 89102167 | 89103331 |
| PAPSS2 | chr10 | 89419475 | 89507462 |
| ATAD1 | chr10 | 89512874 | 89577917 |
| KLLN | chr10 | 89618917 | 89623194 |
| IFIT3 | chr10 | 91087601 | 91100725 |
| RPP30 | chr10 | 92631473 | 92668312 |
| HECTD2 | chr10 | 93170095 | 93274520 |
| TNKS2 | chr10 | 93558150 | 93625232 |
| BTAF1 | chr10 | 93683735 | 93790080 |
| CPEB3 | chr10 | 93808396 | 94050875 |
| 5-Mar | chr10 | 94050923 | 94113721 |
| IDE | chr10 | 94211440 | 94333852 |
| EXOC6 | chr10 | 94608224 | 94819251 |
| CEP55 | chr10 | 95256388 | 95288849 |
| FRA10AC1 | chr10 | 95448836 | 95460095 |
| NOC3L | chr10 | 96092988 | 96122683 |
| HELLS | chr10 | 96305573 | 96361856 |
| ALDH18A1 | chr10 | 97365685 | 97416567 |
| CCNJ | chr10 | 97803158 | 97820625 |
| LCOR | chr10 | 98592711 | 98724198 |
| ARHGAP19-SLIT1 | chr10 | 98912798 | 99052430 |
| ARHGAP19 | chr10 | 98981929 | 99052430 |
| FRAT2 | chr10 | 99092253 | 99094458 |
| RRP12 | chr10 | 99116457 | 99161127 |
| EXOSC1 | chr10 | 99195665 | 99205768 |
| ZDHHC16 | chr10 | 99205929 | 99217127 |
| UBTD1 | chr10 | 99258767 | 99330960 |
| MARVELD1 | chr10 | 99473464 | 99477909 |
| ZFYVE27 | chr10 | 99496877 | 99520664 |
| R3HCC1L | chr10 | 99894380 | 100004654 |
| GOT1 | chr10 | 101156626 | 101190530 |
| SLC25A28 | chr10 | 101370274 | 101371868 |
| COX15 | chr10 | 101468504 | 101492423 |
| CUTC | chr10 | 101491957 | 101515894 |
| ERLIN1 | chr10 | 101909846 | 101945734 |
| CHUK | chr10 | 101948123 | 101989344 |
| CWF19L1 | chr10 | 101992054 | 102027437 |
| BLOC1S2 | chr10 | 102033034 | 102046469 |
| LINC00263 | chr10 | 102133332 | 102148111 |
| SEC31B | chr10 | 102267181 | 102289636 |
| NDUFB8 | chr10 | 102283496 | 102289636 |
| FAM178A | chr10 | 102672325 | 102724891 |
| MRPL43 | chr10 | 102737578 | 102747272 |
| C10orf2 | chr10 | 102747292 | 102754158 |
| LZTS2 | chr10 | 102759232 | 102767593 |
| KAZALD1 | chr10 | 102820998 | 102825351 |
| FBXW4 | chr10 | 103370420 | 103454743 |
| LOC100289509 | chr10 | 103578824 | 103588646 |
| HPS6 | chr10 | 103825123 | 103827795 |
| LDB1 | chr10 | 103867324 | 103874723 |
| SUFU | chr10 | 104263718 | 104393214 |
| ARL3 | chr10 | 104433483 | 104474190 |
| WBP1L | chr10 | 104535887 | 104576021 |
| C10orf32-ASMT | chr10 | 104613966 | 104661655 |
| C10orf32 | chr10 | 104613966 | 104624718 |
| USMG5 | chr10 | 105148808 | 105156270 |
| MIR1307 | chr10 | 105154009 | 105154158 |
| PDCD11 | chr10 | 105156411 | 105206019 |
| OBFC1 | chr10 | 105637317 | 105678045 |
| SLK | chr10 | 105727469 | 105787342 |
| SFR1 | chr10 | 105881946 | 105886143 |
| GSTO1 | chr10 | 106013951 | 106027222 |
| MIR4482-1 | chr10 | 106028093 | 106028163 |
| XPNPEP1 | chr10 | 111635285 | 111683311 |
| SMC3 | chr10 | 112327448 | 112364392 |
| BBIP1 | chr10 | 112658487 | 112679124 |
| SHOC2 | chr10 | 112679300 | 112773425 |
| ZDHHC6 | chr10 | 114190057 | 114206672 |
| VTI1A | chr10 | 114206755 | 114578504 |
| TCF7L2 | chr10 | 114710008 | 114927436 |
| CASP7 | chr10 | 115439427 | 115490668 |
| DCLRE1A | chr10 | 115594482 | 115613975 |
| NHLRC2 | chr10 | 115614390 | 115672265 |
| MIR2110 | chr10 | 115933863 | 115933938 |
| ABLIM1 | chr10 | 116190868 | 116444414 |
| TRUB1 | chr10 | 116697951 | 116737439 |
| KIAA1598 | chr10 | 118642887 | 118765088 |
| PDZD8 | chr10 | 119042605 | 119134937 |
| FAM204A | chr10 | 120068571 | 120101839 |
| CACUL1 | chr10 | 120440493 | 120514758 |
| SNORA19 | chr10 | 120819524 | 120819650 |
| PRDX3 | chr10 | 120927214 | 120938345 |
| GRK5 | chr10 | 120967196 | 121215131 |
| PPAPDC1A | chr10 | 122216465 | 122341296 |
| WDR11-AS1 | chr10 | 122521323 | 122610691 |
| WDR11 | chr10 | 122610686 | 122669038 |
| ATE1 | chr10 | 123502624 | 123687546 |
| NSMCE4A | chr10 | 123719533 | 123734273 |
| PLEKHA1 | chr10 | 124145584 | 124191871 |
| FAM24B-CUZD1 | chr10 | 124591670 | 124639157 |
| FAM24B | chr10 | 124608609 | 124639157 |
| LOC399815 | chr10 | 124639148 | 124658230 |
| IKZF5 | chr10 | 124750321 | 124768366 |
| ACADSB | chr10 | 124768428 | 124817806 |
| METTL10 | chr10 | 126447405 | 126480439 |
| FAM175B | chr10 | 126490353 | 126525239 |
| ZRANB1 | chr10 | 126630691 | 126676005 |
| CTBP2 | chr10 | 126676417 | 126716453 |
| MIR4296 | chr10 | 126721351 | 126721439 |
| FLJ37035 | chr10 | 127393858 | 127408062 |
| C10orf137 | chr10 | 127408083 | 127452712 |
| UROS | chr10 | 127477146 | 127511837 |
| BCCIP | chr10 | 127512103 | 127531376 |
| DOCK1 | chr10 | 128594022 | 129250780 |
| GLRX3 | chr10 | 131934638 | 131982785 |
| PPP2R2D | chr10 | 133753534 | 133770053 |
| BNIP3 | chr10 | 133783190 | 133795435 |
| LRRC27 | chr10 | 134145613 | 134195010 |
| TUBGCP2 | chr10 | 135092133 | 135122660 |
| ZNF511 | chr10 | 135122422 | 135126666 |
| PRAP1 | chr10 | 135122893 | 135166187 |
| MTG1 | chr10 | 135214832 | 135234174 |
| PHRF1 | chr11 | 576482 | 612222 |
| TALDO1 | chr11 | 747431 | 765024 |
| CD81 | chr11 | 2398546 | 2418649 |
| NAP1L4 | chr11 | 2965659 | 3013607 |
| NUP98 | chr11 | 3696239 | 3819022 |
| ILK | chr11 | 6625206 | 6632099 |
| EIF3F | chr11 | 8008444 | 8017719 |
| RPL27A | chr11 | 8703994 | 8711419 |
| DENND5A | chr11 | 9160374 | 9286873 |
| IPO7 | chr11 | 9406168 | 9469674 |
| WEE1 | chr11 | 9597087 | 9603017 |
| SWAP70 | chr11 | 9685627 | 9774507 |
| EIF4G2 | chr11 | 10818592 | 10829543 |
| USP47 | chr11 | 11862969 | 11980872 |
| MICAL2 | chr11 | 12115542 | 12285329 |
| BTBD10 | chr11 | 13409555 | 13461826 |
| FAR1 | chr11 | 13690205 | 13753893 |
| RRAS2 | chr11 | 14299465 | 14380730 |
| C11orf58 | chr11 | 16760147 | 16779901 |
| NAV2 | chr11 | 19734880 | 20143147 |
| RCN1 | chr11 | 32118079 | 32127272 |
| EIF3M | chr11 | 32605312 | 32624037 |
| CSTF3 | chr11 | 33106129 | 33183037 |
| HIPK3 | chr11 | 33278867 | 33378568 |
| CD59 | chr11 | 33724555 | 33744273 |
| CAPRIN1 | chr11 | 34073229 | 34124157 |
| API5 | chr11 | 43333504 | 43366080 |
| CKAP5 | chr11 | 46765083 | 46867859 |
| CELF1 | chr11 | 47487488 | 47574792 |
| FNBP4 | chr11 | 47738068 | 47788993 |
| ZDHHC5 | chr11 | 57435473 | 57468659 |
| TMX2-CTNND1 | chr11 | 57479994 | 57586652 |
| CTNND1 | chr11 | 57529233 | 57586652 |
| ZFP91 | chr11 | 58346586 | 58389023 |
| ZFP91-CNTF | chr11 | 58346586 | 58393205 |
| CCDC86 | chr11 | 60609428 | 60618561 |
| PRPF19 | chr11 | 60658019 | 60674061 |
| DDB1 | chr11 | 61066918 | 61100684 |
| FEN1 | chr11 | 61560108 | 61564714 |
| FADS2 | chr11 | 61595712 | 61634825 |
| FTH1 | chr11 | 61731756 | 61735132 |
| INCENP | chr11 | 61891444 | 61920635 |
| AHNAK | chr11 | 62283373 | 62314332 |
| EEF1G | chr11 | 62327072 | 62342401 |
| MTA2 | chr11 | 62360674 | 62369312 |
| HNRNPUL2-BSCL2 | chr11 | 62457733 | 62494857 |
| HNRNPUL2 | chr11 | 62480096 | 62494857 |
| SNHG1 | chr11 | 62619459 | 62623360 |
| SNORD26 | chr11 | 62622763 | 62622838 |
| SNORD25 | chr11 | 62623036 | 62623103 |
| SLC3A2 | chr11 | 62648343 | 62656355 |
| MARK2 | chr11 | 63606399 | 63678492 |
| STIP1 | chr11 | 63953586 | 63972020 |
| SF1 | chr11 | 64532075 | 64546316 |
| EHD1 | chr11 | 64620207 | 64647149 |
| CDC42EP2 | chr11 | 65082288 | 65089900 |
| NEAT1 | chr11 | 65190268 | 65194003 |
| MALAT1 | chr11 | 65265232 | 65273939 |
| MAP3K11 | chr11 | 65365225 | 65381720 |
| CFL1 | chr11 | 65622284 | 65626886 |
| FOSL1 | chr11 | 65659691 | 65667997 |
| C11orf68 | chr11 | 65684282 | 65686531 |
| RBM14 | chr11 | 66384052 | 66397397 |
| RBM14-RBM4 | chr11 | 66384052 | 66412103 |
| RBM4 | chr11 | 66406087 | 66435858 |
| KDM2A | chr11 | 66886739 | 67025550 |
| PPP6R3 | chr11 | 68228185 | 68382801 |
| CCND1 | chr11 | 69455872 | 69469242 |
| FADD | chr11 | 70049268 | 70053508 |
| CTTN | chr11 | 70244611 | 70282690 |
| RNF121 | chr11 | 71639767 | 71708643 |
| NUMA1 | chr11 | 71713910 | 71791573 |
| FCHSD2 | chr11 | 72547789 | 72853143 |
| RAB6A | chr11 | 73386682 | 73472201 |
| SPCS2 | chr11 | 74660291 | 74690076 |
| RPS3 | chr11 | 75110534 | 75117957 |
| SNORD15A | chr11 | 75111434 | 75111582 |
| SERPINH1 | chr11 | 75273414 | 75283849 |
| UVRAG | chr11 | 75526211 | 75855282 |
| RSF1 | chr11 | 77377273 | 77414578 |
| NDUFC2-KCTD14 | chr11 | 77726760 | 77791265 |
| NDUFC2 | chr11 | 77779392 | 77791265 |
| GAB2 | chr11 | 77926335 | 78128868 |
| PCF11 | chr11 | 82868136 | 82896835 |
| CREBZF | chr11 | 85368607 | 85376182 |
| PICALM | chr11 | 85668213 | 85780139 |
| EED | chr11 | 85955805 | 85989785 |
| PRSS23 | chr11 | 86511490 | 86522273 |
| FZD4 | chr11 | 86656716 | 86666440 |
| CHORDC1 | chr11 | 89933596 | 89956532 |
| TAF1D | chr11 | 93463446 | 93474703 |
| MED17 | chr11 | 93517404 | 93546496 |
| BIRC2 | chr11 | 102218104 | 102249401 |
| TMEM123 | chr11 | 102267055 | 102323775 |
| SLC35F2 | chr11 | 107661716 | 107729914 |
| CUL5 | chr11 | 107879407 | 107978488 |
| NPAT | chr11 | 108028118 | 108093365 |
| RDX | chr11 | 110100165 | 110167437 |
| NNMT | chr11 | 114166534 | 114183238 |
| RBM7 | chr11 | 114271383 | 114279635 |
| PAFAH1B2 | chr11 | 117014999 | 117041761 |
| ARCN1 | chr11 | 118443101 | 118473747 |
| DDX6 | chr11 | 118618472 | 118661972 |
| H2AFX | chr11 | 118964584 | 118966177 |
| MCAM | chr11 | 119179233 | 119187840 |
| ARHGEF12 | chr11 | 120207617 | 120360645 |
| HSPA8 | chr11 | 122928199 | 122932901 |
| ESAM | chr11 | 124623018 | 124627433 |
| ROBO4 | chr11 | 124754113 | 124767831 |
| ETS1 | chr11 | 128328655 | 128392205 |
| FLI1 | chr11 | 128563810 | 128683162 |
| APLP2 | chr11 | 129939715 | 130014706 |
| BET1L | chr11 | 202923 | 207422 |
| RIC8A | chr11 | 208529 | 215110 |
| SIRT3 | chr11 | 215029 | 236362 |
| PSMD13 | chr11 | 236807 | 252984 |
| IFITM2 | chr11 | 308106 | 309410 |
| IFITM3 | chr11 | 319672 | 320914 |
| PTDSS2 | chr11 | 450279 | 491387 |
| RNH1 | chr11 | 494511 | 503570 |
| LOC143666 | chr11 | 573807 | 575885 |
| DEAF1 | chr11 | 644224 | 695740 |
| TMEM80 | chr11 | 695590 | 704131 |
| PDDC1 | chr11 | 767222 | 777487 |
| RPLP2 | chr11 | 809935 | 812876 |
| SNORA52 | chr11 | 811680 | 811814 |
| CD151 | chr11 | 832951 | 838835 |
| POLR2L | chr11 | 839720 | 842529 |
| TSPAN4 | chr11 | 844445 | 867116 |
| AP2A2 | chr11 | 925808 | 1012245 |
| TOLLIP | chr11 | 1295597 | 1330892 |
| MRPL23 | chr11 | 1968501 | 1977839 |
| TSSC4 | chr11 | 2421717 | 2425106 |
| KCNQ1OT1 | chr11 | 2629557 | 2721228 |
| PHLDA2 | chr11 | 2949502 | 2950650 |
| CARS | chr11 | 3022151 | 3078681 |
| ZNF195 | chr11 | 3379156 | 3400452 |
| PGAP2 | chr11 | 3819188 | 3847601 |
| RHOG | chr11 | 3848207 | 3862213 |
| RRM1 | chr11 | 4115923 | 4160106 |
| TRIM5 | chr11 | 5684424 | 5706339 |
| TRIM22 | chr11 | 5710816 | 5732093 |
| FAM160A2 | chr11 | 6232563 | 6255941 |
| PRKCDBP | chr11 | 6340175 | 6341740 |
| ARFIP2 | chr11 | 6495912 | 6502709 |
| TIMM10B | chr11 | 6502676 | 6505911 |
| RRP8 | chr11 | 6621143 | 6624880 |
| TAF10 | chr11 | 6632047 | 6633475 |
| TPP1 | chr11 | 6633996 | 6640585 |
| MRPL17 | chr11 | 6701615 | 6704632 |
| PPFIBP2 | chr11 | 7626964 | 7674996 |
| SNORA3 | chr11 | 8705773 | 8705903 |
| SNORA45 | chr11 | 8706985 | 8707116 |
| TMEM9B | chr11 | 8968839 | 8985989 |
| TMEM41B | chr11 | 9302200 | 9336315 |
| LOC644656 | chr11 | 9481102 | 9482245 |
| ZNF143 | chr11 | 9482511 | 9550071 |
| LOC440028 | chr11 | 9776316 | 9781080 |
| SBF2 | chr11 | 9800213 | 10315754 |
| ADM | chr11 | 10326641 | 10328923 |
| CTR9 | chr11 | 10772810 | 10801290 |
| SNORD97 | chr11 | 10823013 | 10823155 |
| ZBED5 | chr11 | 10874294 | 10879592 |
| ZBED5-AS1 | chr11 | 10879763 | 10900823 |
| MICALCL | chr11 | 12308446 | 12380691 |
| PARVA | chr11 | 12399025 | 12556903 |
| TEAD1 | chr11 | 12695968 | 12966284 |
| COPB1 | chr11 | 14479048 | 14521404 |
| PSMA1 | chr11 | 14515187 | 14541991 |
| RPS13 | chr11 | 17095938 | 17099220 |
| PIK3C2A | chr11 | 17108123 | 17191353 |
| HPS5 | chr11 | 18300216 | 18343721 |
| GTF2H1 | chr11 | 18343815 | 18388590 |
| LDHA | chr11 | 18417812 | 18429765 |
| TSG101 | chr11 | 18501857 | 18548503 |
| UEVLD | chr11 | 18552949 | 18610293 |
| SPTY2D1 | chr11 | 18627947 | 18656020 |
| ZDHHC13 | chr11 | 19138691 | 19197967 |
| E2F8 | chr11 | 19245609 | 19262507 |
| LOC100126784 | chr11 | 19732479 | 19736218 |
| NAV2 | chr11 | 20044101 | 20143147 |
| PRMT3 | chr11 | 20409075 | 20530879 |
| SVIP | chr11 | 22843597 | 22851382 |
| LIN7C | chr11 | 27515964 | 27528326 |
| BDNF-AS | chr11 | 27528398 | 27699350 |
| KIF18A | chr11 | 28042162 | 28129746 |
| METTL15 | chr11 | 28129797 | 28355054 |
| ARL14EP | chr11 | 30344648 | 30359165 |
| IMMP1L | chr11 | 31473744 | 31531169 |
| ELP4 | chr11 | 31531296 | 31805329 |
| QSER1 | chr11 | 32914791 | 33001814 |
| CSTF3-AS1 | chr11 | 33183202 | 33213142 |
| C11orf91 | chr11 | 33719653 | 33722286 |
| NAT10 | chr11 | 34127110 | 34168458 |
| APIP | chr11 | 34903842 | 34937939 |
| PDHX | chr11 | 34937676 | 35017675 |
| CD44 | chr11 | 35160416 | 35253949 |
| FJX1 | chr11 | 35639734 | 35642421 |
| TRIM44 | chr11 | 35684352 | 35830930 |
| MIR3973 | chr11 | 36031647 | 36031754 |
| TRAF6 | chr11 | 36505316 | 36531863 |
| TTC17 | chr11 | 43380434 | 43516483 |
| HSD17B12 | chr11 | 43702142 | 43878169 |
| SLC35C1 | chr11 | 45826640 | 45834567 |
| PEX16 | chr11 | 45931219 | 45939674 |
| DGKZ | chr11 | 46383144 | 46402104 |
| AMBRA1 | chr11 | 46417961 | 46615619 |
| HARBI1 | chr11 | 46624855 | 46638777 |
| ATG13 | chr11 | 46638825 | 46697568 |
| ARHGAP1 | chr11 | 46698624 | 46722215 |
| ZNF408 | chr11 | 46722316 | 46727466 |
| LRP4-AS1 | chr11 | 46867961 | 46895967 |
| ARFGAP2 | chr11 | 47185848 | 47198676 |
| DDB2 | chr11 | 47236492 | 47260769 |
| MADD | chr11 | 47291198 | 47351582 |
| SLC39A13 | chr11 | 47430045 | 47438051 |
| PSMC3 | chr11 | 47440319 | 47448024 |
| CELF1 | chr11 | 47487488 | 47545540 |
| PTPMT1 | chr11 | 47586981 | 47595013 |
| KBTBD4 | chr11 | 47593748 | 47600341 |
| NDUFS3 | chr11 | 47600561 | 47603400 |
| MTCH2 | chr11 | 47638857 | 47664206 |
| NUP160 | chr11 | 47799669 | 47870057 |
| TNKS1BP1 | chr11 | 57067102 | 57089671 |
| SSRP1 | chr11 | 57093458 | 57103351 |
| SLC43A3 | chr11 | 57174426 | 57194560 |
| TIMM10 | chr11 | 57295935 | 57298232 |
| CLP1 | chr11 | 57425215 | 57429337 |
| MED19 | chr11 | 57471186 | 57479673 |
| TMX2-CTNND1 | chr11 | 57479994 | 57586652 |
| TMX2 | chr11 | 57479994 | 57508445 |
| C11orf31 | chr11 | 57508721 | 57510883 |
| LPXN | chr11 | 58294343 | 58343390 |
| FAM111A | chr11 | 58910317 | 58922511 |
| OSBP | chr11 | 59341870 | 59383617 |
| PATL1 | chr11 | 59404191 | 59436511 |
| STX3 | chr11 | 59522531 | 59573355 |
| TMEM109 | chr11 | 60681370 | 60690915 |
| VPS37C | chr11 | 60897727 | 60928916 |
| DAK | chr11 | 61100653 | 61116231 |
| CYB561A3 | chr11 | 61116219 | 61129755 |
| TMEM138 | chr11 | 61129472 | 61136975 |
| CPSF7 | chr11 | 61170119 | 61197383 |
| SDHAF2 | chr11 | 61197596 | 61214239 |
| TMEM258 | chr11 | 61556601 | 61560085 |
| MIR611 | chr11 | 61559966 | 61560033 |
| FADS1 | chr11 | 61567096 | 61584529 |
| MIR1908 | chr11 | 61582632 | 61582712 |
| FADS3 | chr11 | 61640997 | 61659006 |
| MIR3654 | chr11 | 62327634 | 62327778 |
| TUT1 | chr11 | 62342516 | 62359109 |
| EML3 | chr11 | 62369690 | 62380237 |
| GANAB | chr11 | 62392297 | 62414198 |
| INTS5 | chr11 | 62414319 | 62420774 |
| C11orf48 | chr11 | 62430288 | 62439241 |
| METTL12 | chr11 | 62432778 | 62434923 |
| SNORA57 | chr11 | 62432893 | 62433042 |
| C11orf83 | chr11 | 62439125 | 62441162 |
| UBXN1 | chr11 | 62443971 | 62446527 |
| TTC9C | chr11 | 62495583 | 62506108 |
| TMEM179B | chr11 | 62554873 | 62557872 |
| TMEM223 | chr11 | 62557786 | 62559486 |
| NXF1 | chr11 | 62559597 | 62572964 |
| STX5 | chr11 | 62574331 | 62599563 |
| WDR74 | chr11 | 62600382 | 62609281 |
| SNORD22 | chr11 | 62620381 | 62620507 |
| SNORD31 | chr11 | 62620797 | 62620865 |
| SNORD30 | chr11 | 62621134 | 62621204 |
| SNORD29 | chr11 | 62621375 | 62621440 |
| SNORD28 | chr11 | 62622092 | 62622167 |
| SNORD27 | chr11 | 62622483 | 62622555 |
| SLC3A2 | chr11 | 62623483 | 62656355 |
| ATL3 | chr11 | 63396436 | 63439444 |
| RTN3 | chr11 | 63448921 | 63527363 |
| C11orf95 | chr11 | 63527363 | 63536113 |
| C11orf84 | chr11 | 63580922 | 63595190 |
| MARK2 | chr11 | 63655986 | 63678492 |
| COX8A | chr11 | 63742078 | 63744015 |
| OTUB1 | chr11 | 63753324 | 63765892 |
| FERMT3 | chr11 | 63974151 | 63991363 |
| FKBP2 | chr11 | 64008412 | 64011607 |
| PPP1R14B | chr11 | 64011950 | 64014413 |
| PLCB3 | chr11 | 64018994 | 64036924 |
| TRMT112 | chr11 | 64084164 | 64085033 |
| PRDX5 | chr11 | 64085559 | 64089295 |
| CDCA5 | chr11 | 64844926 | 64851615 |
| ZFPL1 | chr11 | 64851693 | 64855874 |
| ZNHIT2 | chr11 | 64883874 | 64885170 |
| FAU | chr11 | 64888098 | 64889672 |
| MRPL49 | chr11 | 64889715 | 64894841 |
| SYVN1 | chr11 | 64894750 | 64902003 |
| CAPN1 | chr11 | 64948685 | 64979477 |
| POLA2 | chr11 | 65029431 | 65065088 |
| DPF2 | chr11 | 65101224 | 65120451 |
| SLC25A45 | chr11 | 65142662 | 65150142 |
| FRMD8 | chr11 | 65154040 | 65180995 |
| SCYL1 | chr11 | 65292547 | 65306182 |
| LTBP3 | chr11 | 65306029 | 65325699 |
| SSSCA1-AS1 | chr11 | 65336690 | 65337884 |
| SSSCA1 | chr11 | 65337942 | 65339239 |
| FAM89B | chr11 | 65339819 | 65341669 |
| PCNXL3 | chr11 | 65383782 | 65404910 |
| SIPA1 | chr11 | 65407591 | 65418391 |
| RELA | chr11 | 65421071 | 65430443 |
| KAT5 | chr11 | 65479472 | 65487077 |
| RNASEH2C | chr11 | 65485143 | 65488409 |
| MUS81 | chr11 | 65627871 | 65633914 |
| FIBP | chr11 | 65651210 | 65656010 |
| CCDC85B | chr11 | 65657874 | 65659106 |
| DRAP1 | chr11 | 65686727 | 65689048 |
| SART1 | chr11 | 65729159 | 65747607 |
| EIF1AD | chr11 | 65764015 | 65769637 |
| BANF1 | chr11 | 65769549 | 65771617 |
| SF3B2 | chr11 | 65819815 | 65836382 |
| PACS1 | chr11 | 65837823 | 66012218 |
| RAB1B | chr11 | 66036055 | 66044963 |
| YIF1A | chr11 | 66052050 | 66056638 |
| RIN1 | chr11 | 66099541 | 66104000 |
| BRMS1 | chr11 | 66104803 | 66112582 |
| DPP3 | chr11 | 66247483 | 66277130 |
| RBM4B | chr11 | 66432469 | 66445219 |
| RCE1 | chr11 | 66610882 | 66614003 |
| ADRBK1 | chr11 | 67033904 | 67054029 |
| ANKRD13D | chr11 | 67065542 | 67069955 |
| POLD4 | chr11 | 67118235 | 67121067 |
| PPP1CA | chr11 | 67165651 | 67188654 |
| RPS6KB2 | chr11 | 67195934 | 67202879 |
| CDK2AP2 | chr11 | 67273960 | 67275658 |
| GSTP1 | chr11 | 67351065 | 67354124 |
| NDUFS8 | chr11 | 67798083 | 67801197 |
| TCIRG1 | chr11 | 67806461 | 67818366 |
| SUV420H1 | chr11 | 67923506 | 67980784 |
| LRP5 | chr11 | 68080107 | 68216743 |
| MRPL21 | chr11 | 68659671 | 68671303 |
| IGHMBP2 | chr11 | 68671318 | 68708069 |
| MYEOV | chr11 | 69061621 | 69064754 |
| ORAOV1 | chr11 | 69480331 | 69490165 |
| PPFIA1 | chr11 | 70116805 | 70230607 |
| DHCR7 | chr11 | 71145456 | 71159477 |
| NADSYN1 | chr11 | 71164216 | 71212581 |
| LOC100133315 | chr11 | 71576554 | 71639493 |
| MIR3165 | chr11 | 71783273 | 71783348 |
| LRTOMT | chr11 | 71791376 | 71821828 |
| LAMTOR1 | chr11 | 71808337 | 71814433 |
| ANAPC15 | chr11 | 71820628 | 71823822 |
| INPPL1 | chr11 | 71935881 | 71950188 |
| ARAP1 | chr11 | 72396113 | 72433403 |
| ATG16L2 | chr11 | 72525450 | 72540680 |
| ARHGEF17 | chr11 | 73019662 | 73080425 |
| RELT | chr11 | 73087712 | 73108519 |
| COA4 | chr11 | 73583712 | 73587890 |
| PAAF1 | chr11 | 73588013 | 73638781 |
| C2CD3 | chr11 | 73745479 | 73882064 |
| PPME1 | chr11 | 73882107 | 73965748 |
| PGM2L1 | chr11 | 74041360 | 74109502 |
| POLD3 | chr11 | 74303649 | 74354105 |
| RNF169 | chr11 | 74459912 | 74553458 |
| XRRA1 | chr11 | 74551954 | 74660232 |
| ARRB1 | chr11 | 74971165 | 75062875 |
| MIR326 | chr11 | 75046135 | 75046230 |
| SNORD15B | chr11 | 75115464 | 75115610 |
| PRKRIR | chr11 | 76061003 | 76091880 |
| C11orf30 | chr11 | 76156068 | 76262589 |
| LRRC32 | chr11 | 76368567 | 76381044 |
| TSKU | chr11 | 76494284 | 76509198 |
| CLNS1A | chr11 | 77327195 | 77348851 |
| AAMDC | chr11 | 77532196 | 77629464 |
| INTS4 | chr11 | 77589765 | 77705717 |
| GAB2 | chr11 | 77926335 | 78052926 |
| PRCP | chr11 | 82535408 | 82611557 |
| C11orf82 | chr11 | 82612736 | 82645699 |
| RAB30 | chr11 | 82692477 | 82745606 |
| LOC100506233 | chr11 | 82783107 | 82784754 |
| ANKRD42 | chr11 | 82905290 | 82926242 |
| CCDC90B | chr11 | 82972501 | 82997377 |
| TMEM126B | chr11 | 85339616 | 85346128 |
| SYTL2 | chr11 | 85405264 | 85437511 |
| C11orf73 | chr11 | 86013252 | 86056985 |
| ME3 | chr11 | 86152149 | 86383678 |
| LOC100506368 | chr11 | 86666662 | 86711989 |
| TMEM135 | chr11 | 86748885 | 87039876 |
| CTSC | chr11 | 88053980 | 88070941 |
| NOX4 | chr11 | 89057521 | 89224653 |
| SLC36A4 | chr11 | 92880849 | 92931095 |
| KIAA1731 | chr11 | 93394815 | 93463522 |
| SNORA8 | chr11 | 93463680 | 93465665 |
| SNORD5 | chr11 | 93466393 | 93466466 |
| SNORA18 | chr11 | 93466631 | 93466763 |
| MIR1304 | chr11 | 93466839 | 93466930 |
| SNORA40 | chr11 | 93468275 | 93468402 |
| C11orf54 | chr11 | 93482176 | 93496247 |
| PANX1 | chr11 | 93862093 | 93915137 |
| MRE11A | chr11 | 94150468 | 94227040 |
| ANKRD49 | chr11 | 94227152 | 94232744 |
| AMOTL1 | chr11 | 94501507 | 94609918 |
| CWC15 | chr11 | 94695786 | 94706776 |
| SRSF8 | chr11 | 94800055 | 94804388 |
| FAM76B | chr11 | 95502105 | 95522954 |
| CEP57 | chr11 | 95523624 | 95565857 |
| MTMR2 | chr11 | 95566043 | 95657371 |
| MAML2 | chr11 | 95711439 | 96076344 |
| MIR1260B | chr11 | 96074601 | 96074690 |
| CCDC82 | chr11 | 96085928 | 96123083 |
| JRKL | chr11 | 96123157 | 96126727 |
| ANGPTL5 | chr11 | 101761404 | 101787253 |
| KIAA1377 | chr11 | 101826522 | 101871793 |
| DCUN1D5 | chr11 | 102921412 | 102962944 |
| LOC643733 | chr11 | 104772275 | 104788902 |
| CASP4 | chr11 | 104819546 | 104839325 |
| CARD16 | chr11 | 104912052 | 104916051 |
| MSANTD4 | chr11 | 105878628 | 105892954 |
| KBTBD3 | chr11 | 105921824 | 105948465 |
| AASDHPPT | chr11 | 105948291 | 105969419 |
| ALKBH8 | chr11 | 107373452 | 107436461 |
| ACAT1 | chr11 | 107992257 | 108018891 |
| ATM | chr11 | 108093558 | 108239826 |
| C11orf65 | chr11 | 108201925 | 108338258 |
| KDELC2 | chr11 | 108342832 | 108362231 |
| DDX10 | chr11 | 108535815 | 108792970 |
| ZC3H12C | chr11 | 110006578 | 110042566 |
| FDX1 | chr11 | 110300660 | 110335608 |
| PPP2R1B | chr11 | 111608604 | 111637169 |
| FDXACB1 | chr11 | 111744779 | 111750181 |
| C11orf1 | chr11 | 111751499 | 111754797 |
| DIXDC1 | chr11 | 111807926 | 111893307 |
| DLAT | chr11 | 111895537 | 111935002 |
| C11orf57 | chr11 | 111944967 | 111955874 |
| TIMM8B | chr11 | 111955538 | 111957522 |
| SDHD | chr11 | 111957547 | 111966525 |
| PTS | chr11 | 112097087 | 112104695 |
| ZW10 | chr11 | 113603904 | 113644485 |
| ZBTB16 | chr11 | 113930430 | 114060486 |
| C11orf71 | chr11 | 114269255 | 114271272 |
| REXO2 | chr11 | 114310107 | 114321000 |
| BUD13 | chr11 | 116618885 | 116643714 |
| ZNF259 | chr11 | 116649275 | 116658739 |
| SIK3 | chr11 | 116714117 | 116968993 |
| PCSK7 | chr11 | 117075787 | 117102811 |
| RNF214 | chr11 | 117103340 | 117156404 |
| UBE4A | chr11 | 118230295 | 118269926 |
| ATP5L | chr11 | 118272103 | 118280562 |
| KMT2A | chr11 | 118307204 | 118397539 |
| PHLDB1 | chr11 | 118478305 | 118528748 |
| BCL9L | chr11 | 118766850 | 118781613 |
| MIR4492 | chr11 | 118781416 | 118781496 |
| RPS25 | chr11 | 118886421 | 118889057 |
| TRAPPC4 | chr11 | 118889240 | 118891459 |
| MIR3656 | chr11 | 118889653 | 118889722 |
| HYOU1 | chr11 | 118914895 | 118927916 |
| VPS11 | chr11 | 118938492 | 118952688 |
| DPAGT1 | chr11 | 118967212 | 118972785 |
| HINFP | chr11 | 119002148 | 119005765 |
| CBL | chr11 | 119076985 | 119178859 |
| RNF26 | chr11 | 119205209 | 119208024 |
| TBCEL | chr11 | 120894812 | 120960354 |
| SC5D | chr11 | 121163576 | 121184119 |
| MIR125B1 | chr11 | 121970464 | 121970552 |
| MIRLET7A2 | chr11 | 122017229 | 122017301 |
| MIR100 | chr11 | 122022936 | 122023016 |
| UBASH3B | chr11 | 122526397 | 122685187 |
| ZNF202 | chr11 | 123594996 | 123612363 |
| TBRG1 | chr11 | 124492741 | 124505822 |
| MSANTD2 | chr11 | 124636393 | 124646138 |
| EI24 | chr11 | 125439297 | 125454575 |
| STT3A | chr11 | 125462738 | 125491280 |
| CHEK1 | chr11 | 125495030 | 125527042 |
| HYLS1 | chr11 | 125756954 | 125770541 |
| RPUSD4 | chr11 | 126071988 | 126081587 |
| FAM118B | chr11 | 126081618 | 126132879 |
| SRPR | chr11 | 126132813 | 126138877 |
| FOXRED1 | chr11 | 126139039 | 126148027 |
| ST3GAL4-AS1 | chr11 | 126211611 | 126225482 |
| ST3GAL4 | chr11 | 126225788 | 126284536 |
| FLI1-AS1 | chr11 | 128561566 | 128565918 |
| PRDM10 | chr11 | 129769600 | 129872730 |
| LINC00167 | chr11 | 129872518 | 129875381 |
| SNX19 | chr11 | 130745765 | 130786382 |
| NCAPD3 | chr11 | 134022336 | 134094426 |
| VPS26B | chr11 | 134094560 | 134117686 |
| KDM5A | chr12 | 389222 | 498620 |
| WNK1 | chr12 | 862088 | 1020618 |
| VWF | chr12 | 6058039 | 6233836 |
| CD9 | chr12 | 6309481 | 6347437 |
| TNFRSF1A | chr12 | 6437922 | 6451283 |
| GAPDH | chr12 | 6643584 | 6647537 |
| CHD4 | chr12 | 6679247 | 6716551 |
| ZNF384 | chr12 | 6775642 | 6798738 |
| PTMS | chr12 | 6875540 | 6880118 |
| TPI1 | chr12 | 6976583 | 6980110 |
| ATN1 | chr12 | 7037479 | 7053815 |
| YBX3 | chr12 | 10851675 | 10875953 |
| ETV6 | chr12 | 11802787 | 12048325 |
| CDKN1B | chr12 | 12870301 | 12875305 |
| DDX47 | chr12 | 12878850 | 12982915 |
| EMP1 | chr12 | 13349601 | 13369708 |
| ARHGDIB | chr12 | 15094949 | 15114562 |
| STRAP | chr12 | 16035287 | 16056410 |
| AEBP2 | chr12 | 19592607 | 19675173 |
| LDHB | chr12 | 21788274 | 21810728 |
| ETNK1 | chr12 | 22778075 | 22843608 |
| BCAT1 | chr12 | 24962957 | 25102393 |
| RASSF8 | chr12 | 26111963 | 26225807 |
| ITPR2 | chr12 | 26488284 | 26986131 |
| PPFIBP1 | chr12 | 27677044 | 27848497 |
| MRPS35 | chr12 | 27863705 | 27909237 |
| FAM60A | chr12 | 31433519 | 31479159 |
| PRICKLE1 | chr12 | 42852139 | 42983572 |
| ANO6 | chr12 | 45609769 | 45826134 |
| SCAF11 | chr12 | 46312913 | 46384401 |
| SLC38A1 | chr12 | 46576840 | 46663208 |
| SLC38A2 | chr12 | 46751970 | 46766645 |
| HDAC7 | chr12 | 48176506 | 48213763 |
| KANSL2 | chr12 | 49046994 | 49076008 |
| CCNT1 | chr12 | 49082240 | 49110781 |
| TUBA1B | chr12 | 49521566 | 49525178 |
| TUBA1A | chr12 | 49578577 | 49583107 |
| TUBA1C | chr12 | 49621708 | 49667113 |
| SPATS2 | chr12 | 49760687 | 49921207 |
| FMNL3 | chr12 | 50031723 | 50101197 |
| TMBIM6 | chr12 | 50135593 | 50158717 |
| LIMA1 | chr12 | 50569562 | 50616488 |
| LARP4 | chr12 | 50794591 | 50873788 |
| ACVRL1 | chr12 | 52301201 | 52317145 |
| ACVR1B | chr12 | 52345450 | 52390863 |
| EIF4B | chr12 | 53400061 | 53435993 |
| PCBP2 | chr12 | 53845885 | 53874946 |
| ATF7 | chr12 | 53905842 | 54020199 |
| CBX5 | chr12 | 54624730 | 54673915 |
| HNRNPA1 | chr12 | 54674487 | 54679030 |
| HNRNPA1P10 | chr12 | 54674532 | 54678663 |
| ITGA5 | chr12 | 54789044 | 54813050 |
| SARNP | chr12 | 56186338 | 56211540 |
| PA2G4 | chr12 | 56498102 | 56507694 |
| MYL6 | chr12 | 56552145 | 56554579 |
| CS | chr12 | 56665482 | 56694175 |
| RBMS2 | chr12 | 56915608 | 56989980 |
| ATP5B | chr12 | 57031958 | 57039852 |
| PTGES3 | chr12 | 57057124 | 57082078 |
| NACA | chr12 | 57106210 | 57119326 |
| CTDSP2 | chr12 | 58213709 | 58240747 |
| LRIG3 | chr12 | 59265936 | 59314319 |
| MIRLET7I | chr12 | 62997465 | 62997549 |
| TBK1 | chr12 | 64845839 | 64895899 |
| RASSF3 | chr12 | 65004292 | 65091347 |
| RPSAP52 | chr12 | 66151799 | 66220754 |
| HMGA2 | chr12 | 66218239 | 66352634 |
| CAND1 | chr12 | 67663060 | 67708388 |
| RAP1B | chr12 | 69004618 | 69054385 |
| CPSF6 | chr12 | 69633316 | 69668138 |
| CNOT2 | chr12 | 70637493 | 70748773 |
| PTPRB | chr12 | 70910631 | 71031220 |
| ZFC3H1 | chr12 | 72003378 | 72057749 |
| ATXN7L3B | chr12 | 74931550 | 74935232 |
| KRR1 | chr12 | 75891418 | 75905418 |
| PHLDA1 | chr12 | 76419226 | 76425556 |
| NAP1L1 | chr12 | 76438671 | 76478738 |
| PAWR | chr12 | 79985744 | 80084790 |
| PPP1R12A | chr12 | 80167342 | 80328978 |
| DUSP6 | chr12 | 89741836 | 89746296 |
| LOC256021 | chr12 | 92378751 | 92536447 |
| BTG1 | chr12 | 92534053 | 92539673 |
| EEA1 | chr12 | 93166284 | 93323107 |
| NUDT4 | chr12 | 93771700 | 93797024 |
| NUDT4P1 | chr12 | 93771745 | 93796052 |
| UBE2N | chr12 | 93802087 | 93836026 |
| VEZT | chr12 | 95611521 | 95696566 |
| METAP2 | chr12 | 95867821 | 95909613 |
| ELK3 | chr12 | 96588206 | 96661606 |
| CDK17 | chr12 | 96672038 | 96794366 |
| TMPO | chr12 | 98909350 | 98944157 |
| SLC25A3 | chr12 | 98987402 | 98995778 |
| HSP90B1 | chr12 | 104324111 | 104341708 |
| MIR3652 | chr12 | 104324202 | 104324333 |
| TDG | chr12 | 104359592 | 104382656 |
| TXNRD1 | chr12 | 104680459 | 104744085 |
| EID3 | chr12 | 104697509 | 104698982 |
| KIAA1033 | chr12 | 105501491 | 105562906 |
| C12orf23 | chr12 | 107349543 | 107367813 |
| PRDM4 | chr12 | 108126642 | 108154914 |
| SART3 | chr12 | 108915990 | 108955165 |
| CORO1C | chr12 | 109038884 | 109124619 |
| SSH1 | chr12 | 109176465 | 109251359 |
| KCTD10 | chr12 | 109886459 | 109915155 |
| ATP2A2 | chr12 | 110719031 | 110788897 |
| PPP1CC | chr12 | 111157612 | 111180783 |
| SH2B3 | chr12 | 111843751 | 111889427 |
| ATXN2 | chr12 | 111890017 | 112037480 |
| NAA25 | chr12 | 112464492 | 112546635 |
| PTPN11 | chr12 | 112856535 | 112947717 |
| RAB35 | chr12 | 120532898 | 120554643 |
| RPLP0 | chr12 | 120634502 | 120639014 |
| PXN | chr12 | 120648241 | 120664647 |
| SRSF9 | chr12 | 120899470 | 120907558 |
| RNF10 | chr12 | 120972131 | 121015397 |
| MLEC | chr12 | 121124948 | 121139667 |
| LOC338799 | chr12 | 122233172 | 122241390 |
| SETD1B | chr12 | 122242637 | 122270562 |
| MLXIP | chr12 | 122516651 | 122628967 |
| CLIP1 | chr12 | 122755980 | 122907116 |
| RSRC2 | chr12 | 122989189 | 123011560 |
| HIP1R | chr12 | 123320038 | 123347507 |
| VPS37B | chr12 | 123349874 | 123380712 |
| CDK2AP1 | chr12 | 123745516 | 123756687 |
| TMED2 | chr12 | 124069075 | 124082688 |
| NCOR2 | chr12 | 124808956 | 125052010 |
| UBC | chr12 | 125396191 | 125399587 |
| ANKLE2 | chr12 | 133302253 | 133338451 |
| GOLGA3 | chr12 | 133345494 | 133405288 |
| CCDC77 | chr12 | 498515 | 551806 |
| RAD52 | chr12 | 1021254 | 1099219 |
| ERC1 | chr12 | 1100403 | 1605099 |
| FBXL14 | chr12 | 1675158 | 1703331 |
| ADIPOR2 | chr12 | 1800260 | 1897845 |
| FKBP4 | chr12 | 2904107 | 2914587 |
| ITFG2 | chr12 | 2921786 | 2934237 |
| FOXM1 | chr12 | 2966846 | 2986321 |
| RHNO1 | chr12 | 2986364 | 2998691 |
| TULP3 | chr12 | 3000032 | 3050306 |
| TEAD4 | chr12 | 3068477 | 3149842 |
| C12orf5 | chr12 | 4430358 | 4469190 |
| C12orf4 | chr12 | 4596900 | 4647637 |
| RAD51AP1 | chr12 | 4647949 | 4669213 |
| NDUFA9 | chr12 | 4758263 | 4796720 |
| LTBR | chr12 | 6493198 | 6500737 |
| MRPL51 | chr12 | 6601315 | 6602471 |
| NCAPD2 | chr12 | 6603297 | 6641132 |
| GAPDH | chr12 | 6644408 | 6647537 |
| IFFO1 | chr12 | 6648126 | 6665249 |
| NOP2 | chr12 | 6666035 | 6677498 |
| ING4 | chr12 | 6759703 | 6772308 |
| MLF2 | chr12 | 6857157 | 6862636 |
| LEPREL2 | chr12 | 6938187 | 6949018 |
| CDCA3 | chr12 | 6953962 | 6960456 |
| USP5 | chr12 | 6961284 | 6975795 |
| TPI1 | chr12 | 6977277 | 6980110 |
| SPSB2 | chr12 | 6980099 | 6982521 |
| ATN1 | chr12 | 7033625 | 7053815 |
| C12orf57 | chr12 | 7053202 | 7055165 |
| PHB2 | chr12 | 7074514 | 7079916 |
| SCARNA12 | chr12 | 7076499 | 7076769 |
| EMG1 | chr12 | 7079943 | 7085165 |
| PEX5 | chr12 | 7342281 | 7364079 |
| SLC2A3 | chr12 | 8071823 | 8088892 |
| FOXJ2 | chr12 | 8185358 | 8208118 |
| NECAP1 | chr12 | 8234806 | 8250373 |
| RIMKLB | chr12 | 8850517 | 8929787 |
| M6PR | chr12 | 9092956 | 9102357 |
| MIR1244-1 | chr12 | 9392065 | 9392147 |
| LOC642846 | chr12 | 9436252 | 9466684 |
| DDX12P | chr12 | 9570286 | 9600768 |
| CLEC2B | chr12 | 10004967 | 10022458 |
| CLEC9A | chr12 | 10183275 | 10218565 |
| GABARAPL1 | chr12 | 10365488 | 10375724 |
| MAGOHB | chr12 | 10756363 | 10766208 |
| STYK1 | chr12 | 10771537 | 10826891 |
| PRH1-PRR4 | chr12 | 10998447 | 11324224 |
| LOC100129361 | chr12 | 11323779 | 11328619 |
| LRP6 | chr12 | 12268960 | 12419811 |
| DUSP16 | chr12 | 12626215 | 12715448 |
| GPR19 | chr12 | 12813994 | 12849121 |
| APOLD1 | chr12 | 12878850 | 12944399 |
| GPRC5A | chr12 | 13043955 | 13066600 |
| MIR614 | chr12 | 13068762 | 13068852 |
| LOC100506314 | chr12 | 13132770 | 13137576 |
| GSG1 | chr12 | 13236470 | 13256630 |
| ATF7IP | chr12 | 14518610 | 14651697 |
| HIST4H4 | chr12 | 14923653 | 14924065 |
| H2AFJ | chr12 | 14927269 | 14930936 |
| WBP11 | chr12 | 14939411 | 14956401 |
| C12orf60 | chr12 | 14956505 | 14976791 |
| ART4 | chr12 | 14982244 | 14996413 |
| DERA | chr12 | 16064185 | 16190315 |
| MGST1 | chr12 | 16500576 | 16530123 |
| PDE3A | chr12 | 20522178 | 20837041 |
| PYROXD1 | chr12 | 21590537 | 21624182 |
| RECQL | chr12 | 21621843 | 21654603 |
| GOLT1B | chr12 | 21654698 | 21671337 |
| BCAT1 | chr12 | 24962957 | 25055322 |
| KRAS | chr12 | 25358179 | 25403854 |
| RASSF8-AS1 | chr12 | 26107587 | 26110518 |
| RASSF8 | chr12 | 26126687 | 26225807 |
| ASUN | chr12 | 27058111 | 27091254 |
| FGFR1OP2 | chr12 | 27091304 | 27119581 |
| TM7SF3 | chr12 | 27124505 | 27167339 |
| MED21 | chr12 | 27175454 | 27183606 |
| STK38L | chr12 | 27397077 | 27478890 |
| ARNTL2 | chr12 | 27485786 | 27578746 |
| KLHL42 | chr12 | 27933186 | 27955973 |
| FAR2 | chr12 | 29301935 | 29488549 |
| ERGIC2 | chr12 | 29493578 | 29534143 |
| IPO8 | chr12 | 30781914 | 30848929 |
| CAPRIN2 | chr12 | 30862485 | 30907448 |
| LINC00941 | chr12 | 30948614 | 30955645 |
| DDX11-AS1 | chr12 | 31173696 | 31226781 |
| DDX11 | chr12 | 31226778 | 31257725 |
| FLJ13224 | chr12 | 31477249 | 31478879 |
| DENND5B | chr12 | 31535156 | 31743376 |
| DENND5B-AS1 | chr12 | 31742856 | 31768285 |
| AMN1 | chr12 | 31824070 | 31882108 |
| KIAA1551 | chr12 | 32112352 | 32146043 |
| BICD1 | chr12 | 32260184 | 32531141 |
| FGD4 | chr12 | 32655040 | 32798984 |
| DNM1L | chr12 | 32832136 | 32898584 |
| YARS2 | chr12 | 32899477 | 32908887 |
| GXYLT1 | chr12 | 42475647 | 42538673 |
| ZCRB1 | chr12 | 42705887 | 42719932 |
| PPHLN1 | chr12 | 42719946 | 42842422 |
| TWF1 | chr12 | 44187525 | 44200178 |
| PLEKHA8P1 | chr12 | 45566816 | 45609789 |
| LINC00938 | chr12 | 46119502 | 46121704 |
| ARID2 | chr12 | 46123619 | 46301819 |
| RPAP3 | chr12 | 48055714 | 48099844 |
| RAPGEF3 | chr12 | 48128452 | 48152889 |
| SENP1 | chr12 | 48436680 | 48499775 |
| PFKM | chr12 | 48499655 | 48540187 |
| DDX23 | chr12 | 49223538 | 49245957 |
| ARF3 | chr12 | 49329991 | 49351252 |
| PRKAG1 | chr12 | 49396054 | 49412531 |
| KMT2D | chr12 | 49412757 | 49449107 |
| LMBR1L | chr12 | 49490922 | 49504680 |
| TROAP | chr12 | 49716970 | 49725514 |
| LOC100335030 | chr12 | 49782956 | 49786116 |
| MCRS1 | chr12 | 49952085 | 49961533 |
| NCKAP5L | chr12 | 50184928 | 50222208 |
| RACGAP1 | chr12 | 50382944 | 50419307 |
| SMARCD1 | chr12 | 50478982 | 50494494 |
| CERS5 | chr12 | 50523580 | 50561097 |
| DIP2B | chr12 | 50898767 | 51142450 |
| ATF1 | chr12 | 51157788 | 51214943 |
| SLC11A2 | chr12 | 51379774 | 51419924 |
| LETMD1 | chr12 | 51442081 | 51454207 |
| CSRNP2 | chr12 | 51454987 | 51477437 |
| TFCP2 | chr12 | 51487538 | 51566926 |
| DAZAP2 | chr12 | 51632507 | 51637717 |
| SMAGP | chr12 | 51639132 | 51664202 |
| SLC4A8 | chr12 | 51785100 | 51909547 |
| ACVRL1 | chr12 | 52306112 | 52317145 |
| GRASP | chr12 | 52400728 | 52409673 |
| C12orf44 | chr12 | 52463757 | 52471279 |
| KRT80 | chr12 | 52562779 | 52579533 |
| KRT7 | chr12 | 52626953 | 52642709 |
| KRT8 | chr12 | 53290970 | 53343650 |
| KRT18 | chr12 | 53342842 | 53346685 |
| TENC1 | chr12 | 53442732 | 53458162 |
| ZNF740 | chr12 | 53574534 | 53584654 |
| ESPL1 | chr12 | 53662082 | 53687427 |
| PFDN5 | chr12 | 53689234 | 53693234 |
| C12orf10 | chr12 | 53693469 | 53700965 |
| SP1 | chr12 | 53773978 | 53810226 |
| PRR13 | chr12 | 53835432 | 53840427 |
| TARBP2 | chr12 | 53894704 | 53900215 |
| ATP5G2 | chr12 | 54058943 | 54070512 |
| CBX5 | chr12 | 54624730 | 54653329 |
| COPZ1 | chr12 | 54718873 | 54745635 |
| CD63 | chr12 | 56119226 | 56123457 |
| ORMDL2 | chr12 | 56211805 | 56214959 |
| DNAJC14 | chr12 | 56214743 | 56224342 |
| TMEM198B | chr12 | 56223391 | 56230030 |
| WIBG | chr12 | 56295196 | 56321051 |
| CDK2 | chr12 | 56360555 | 56366568 |
| RAB5B | chr12 | 56367794 | 56390467 |
| RPS26 | chr12 | 56435685 | 56438007 |
| RPL41 | chr12 | 56510373 | 56511616 |
| ZC3H10 | chr12 | 56512029 | 56516278 |
| ESYT1 | chr12 | 56521985 | 56538460 |
| SMARCC2 | chr12 | 56555635 | 56583351 |
| RNF41 | chr12 | 56596287 | 56615715 |
| ANKRD52 | chr12 | 56631590 | 56652143 |
| CNPY2 | chr12 | 56704212 | 56710128 |
| PAN2 | chr12 | 56710006 | 56727533 |
| SPRYD4 | chr12 | 56862300 | 56864767 |
| BAZ2A | chr12 | 56989379 | 57030163 |
| SNORD59B | chr12 | 57037463 | 57037538 |
| SNORD59A | chr12 | 57038810 | 57038885 |
| NAB2 | chr12 | 57482676 | 57489259 |
| STAT6 | chr12 | 57489186 | 57505196 |
| SHMT2 | chr12 | 57624142 | 57628718 |
| MARS | chr12 | 57881719 | 57910438 |
| DDIT3 | chr12 | 57910370 | 57914300 |
| MBD6 | chr12 | 57916658 | 57923931 |
| DCTN2 | chr12 | 57923832 | 57941114 |
| CDK4 | chr12 | 58141509 | 58146230 |
| TSFM | chr12 | 58176527 | 58191370 |
| USP15 | chr12 | 62654120 | 62803501 |
| MON2 | chr12 | 62860596 | 62991363 |
| C12orf61 | chr12 | 62995530 | 62997214 |
| SRGAP1 | chr12 | 64238540 | 64541613 |
| XPOT | chr12 | 64798152 | 64842463 |
| MIR548C | chr12 | 65016288 | 65016385 |
| MIR548Z | chr12 | 65016288 | 65016385 |
| LEMD3 | chr12 | 65563350 | 65642141 |
| MSRB3 | chr12 | 65672422 | 65860687 |
| LLPH | chr12 | 66516848 | 66524533 |
| TMBIM4 | chr12 | 66530716 | 66563807 |
| SNORA70G | chr12 | 69021013 | 69021155 |
| LOC100507250 | chr12 | 69068150 | 69080639 |
| NUP107 | chr12 | 69080730 | 69136473 |
| MDM2 | chr12 | 69201970 | 69239320 |
| YEATS4 | chr12 | 69753531 | 69784576 |
| FRS2 | chr12 | 69864128 | 69973562 |
| MIR3913-1 | chr12 | 69978501 | 69978603 |
| MIR3913-2 | chr12 | 69978502 | 69978602 |
| CCT2 | chr12 | 69979461 | 69995357 |
| BEST3 | chr12 | 70047388 | 70083056 |
| PTPRB | chr12 | 70910631 | 71003624 |
| THAP2 | chr12 | 72057676 | 72074428 |
| RAB21 | chr12 | 72148657 | 72181150 |
| TBC1D15 | chr12 | 72233486 | 72320629 |
| GLIPR1 | chr12 | 75874512 | 75895716 |
| BBS10 | chr12 | 76738265 | 76742222 |
| OSBPL8 | chr12 | 76745577 | 76953589 |
| ZDHHC17 | chr12 | 77157853 | 77247474 |
| E2F7 | chr12 | 77415025 | 77459360 |
| PPP1R12A | chr12 | 80167342 | 80307691 |
| CCDC59 | chr12 | 82746082 | 82752199 |
| METTL25 | chr12 | 82752275 | 82873016 |
| C12orf29 | chr12 | 88429267 | 88443937 |
| CEP290 | chr12 | 88442789 | 88535993 |
| TMTC3 | chr12 | 88536072 | 88593664 |
| KITLG | chr12 | 88886569 | 88974250 |
| POC1B | chr12 | 89813497 | 89920039 |
| POC1B-GALNT4 | chr12 | 89913189 | 89920039 |
| GALNT4 | chr12 | 89913189 | 89918583 |
| LOC643339 | chr12 | 93397533 | 93771512 |
| MRPL42 | chr12 | 93861265 | 93897548 |
| TMCC3 | chr12 | 94960899 | 95044324 |
| NDUFA12 | chr12 | 95365103 | 95397489 |
| NR2C1 | chr12 | 95414004 | 95467404 |
| FGD6 | chr12 | 95470524 | 95611240 |
| NTN4 | chr12 | 96051582 | 96184536 |
| SNRPF | chr12 | 96252708 | 96260238 |
| NEDD1 | chr12 | 97301000 | 97347469 |
| LOC643770 | chr12 | 98879321 | 98897633 |
| TMPO-AS1 | chr12 | 98906750 | 98910004 |
| SNORA53 | chr12 | 98993412 | 98993662 |
| IKBIP | chr12 | 99018029 | 99038829 |
| APAF1 | chr12 | 99039077 | 99129211 |
| UHRF1BP1L | chr12 | 100430862 | 100536642 |
| DEPDC4 | chr12 | 100631745 | 100660857 |
| SCYL2 | chr12 | 100661548 | 100733914 |
| GAS2L3 | chr12 | 100967488 | 101018685 |
| ARL1 | chr12 | 101786902 | 101801572 |
| DRAM1 | chr12 | 102271104 | 102317401 |
| PARPBP | chr12 | 102513955 | 102591298 |
| GNN | chr12 | 104237526 | 104323989 |
| C12orf73 | chr12 | 104343980 | 104350993 |
| NFYB | chr12 | 104510857 | 104532040 |
| TXNRD1 | chr12 | 104609556 | 104744085 |
| CHST11 | chr12 | 104850691 | 105155792 |
| APPL2 | chr12 | 105567074 | 105627183 |
| C12orf75 | chr12 | 105724413 | 105765296 |
| NUAK1 | chr12 | 106457124 | 106533811 |
| CKAP4 | chr12 | 106631658 | 106641713 |
| POLR3B | chr12 | 106751435 | 106903976 |
| LOC100287944 | chr12 | 106890187 | 107168609 |
| RIC8B | chr12 | 107168398 | 107283094 |
| CRY1 | chr12 | 107385142 | 107487635 |
| PWP1 | chr12 | 108079589 | 108106257 |
| ISCU | chr12 | 108956293 | 108963117 |
| CORO1C | chr12 | 109038884 | 109096774 |
| UBE3B | chr12 | 109915427 | 109974510 |
| GLTP | chr12 | 110288747 | 110318293 |
| TCHP | chr12 | 110338322 | 110355874 |
| GIT2 | chr12 | 110367606 | 110434194 |
| ANAPC7 | chr12 | 110810704 | 110841535 |
| GPN3 | chr12 | 110890290 | 110906526 |
| FAM216A | chr12 | 110906231 | 110924979 |
| VPS29 | chr12 | 110929329 | 110939916 |
| RAD9B | chr12 | 110940004 | 110969891 |
| PPTC7 | chr12 | 110972236 | 111021064 |
| BRAP | chr12 | 112079949 | 112123790 |
| ACAD10 | chr12 | 112123856 | 112194911 |
| MAPKAPK5-AS1 | chr12 | 112277572 | 112280706 |
| MAPKAPK5 | chr12 | 112280031 | 112331228 |
| TMEM116 | chr12 | 112369086 | 112451023 |
| ERP29 | chr12 | 112451151 | 112461024 |
| HECTD4 | chr12 | 112597991 | 112819896 |
| RPL6 | chr12 | 112845297 | 112847443 |
| DDX54 | chr12 | 113594977 | 113623284 |
| C12orf52 | chr12 | 113623554 | 113630119 |
| MED13L | chr12 | 116396380 | 116714991 |
| MIR620 | chr12 | 116586364 | 116586459 |
| C12orf49 | chr12 | 117153595 | 117175843 |
| WSB2 | chr12 | 118470491 | 118498951 |
| PEBP1 | chr12 | 118573869 | 118583390 |
| TAOK3 | chr12 | 118587605 | 118810750 |
| SUDS3 | chr12 | 118814357 | 118855840 |
| GCN1L1 | chr12 | 120565013 | 120632513 |
| PXN-AS1 | chr12 | 120639093 | 120650631 |
| COX6A1 | chr12 | 120875892 | 120878545 |
| TRIAP1 | chr12 | 120881763 | 120884215 |
| GATC | chr12 | 120884240 | 120901556 |
| DYNLL1 | chr12 | 120907659 | 120936298 |
| DYNLL1-AS1 | chr12 | 120928140 | 120933749 |
| COQ5 | chr12 | 120941081 | 120966964 |
| SPPL3 | chr12 | 121200312 | 121207941 |
| CAMKK2 | chr12 | 121675494 | 121734556 |
| ANAPC5 | chr12 | 121746047 | 121790265 |
| RNF34 | chr12 | 121837885 | 121862155 |
| ORAI1 | chr12 | 122064454 | 122079946 |
| PSMD9 | chr12 | 122326636 | 122355771 |
| LOC100507066 | chr12 | 122880088 | 122884491 |
| ZCCHC8 | chr12 | 122956145 | 122985543 |
| KNTC1 | chr12 | 123011808 | 123110947 |
| DENR | chr12 | 123237370 | 123255953 |
| OGFOD2 | chr12 | 123459353 | 123464588 |
| ARL6IP4 | chr12 | 123464879 | 123467460 |
| C12orf65 | chr12 | 123718027 | 123742506 |
| CDK2AP1 | chr12 | 123745516 | 123752801 |
| SNRNP35 | chr12 | 123942650 | 123950941 |
| RILPL1 | chr12 | 123955908 | 124018265 |
| DDX55 | chr12 | 124086671 | 124105482 |
| EIF2B1 | chr12 | 124105569 | 124118323 |
| GTF2H3 | chr12 | 124118285 | 124147151 |
| ATP6V0A2 | chr12 | 124196864 | 124246301 |
| SCARB1 | chr12 | 125262173 | 125348519 |
| UBC | chr12 | 125396249 | 125399587 |
| MIR5188 | chr12 | 125400092 | 125400205 |
| SLC15A4 | chr12 | 129277738 | 129308541 |
| STX2 | chr12 | 131274144 | 131323819 |
| RAN | chr12 | 131356782 | 131360826 |
| SFSWAP | chr12 | 132195631 | 132284283 |
| PUS1 | chr12 | 132413812 | 132428406 |
| EP400 | chr12 | 132434464 | 132565011 |
| DDX51 | chr12 | 132621139 | 132628880 |
| NOC4L | chr12 | 132628992 | 132636986 |
| POLE | chr12 | 133200347 | 133263945 |
| PGAM5 | chr12 | 133287392 | 133299323 |
| ZNF140 | chr12 | 133657036 | 133684258 |
| LATS2 | chr13 | 21547175 | 21635722 |
| USP12 | chr13 | 27640286 | 27746033 |
| RPL21 | chr13 | 27825691 | 27830702 |
| RPL21P28 | chr13 | 27825692 | 27830699 |
| SLC7A1 | chr13 | 30083550 | 30169825 |
| USPL1 | chr13 | 31191829 | 31226094 |
| HSPH1 | chr13 | 31710762 | 31736117 |
| STARD13 | chr13 | 33677271 | 33859901 |
| STARD13-AS | chr13 | 33851690 | 33855471 |
| PROSER1 | chr13 | 39584001 | 39612213 |
| FOXO1 | chr13 | 41129800 | 41240734 |
| ELF1 | chr13 | 41506054 | 41556418 |
| NAA16 | chr13 | 41885340 | 41951166 |
| TPT1 | chr13 | 45911303 | 45915297 |
| MED4 | chr13 | 48649863 | 48669277 |
| KPNA3 | chr13 | 50273442 | 50367057 |
| SUGT1 | chr13 | 53226830 | 53262433 |
| DIAPH3 | chr13 | 60239720 | 60738119 |
| KLF12 | chr13 | 74260148 | 74708394 |
| KCTD12 | chr13 | 77454303 | 77460540 |
| RBM26 | chr13 | 79894099 | 79979923 |
| SPRY2 | chr13 | 80910111 | 80913794 |
| MIR17HG | chr13 | 92000073 | 92006829 |
| MBNL2 | chr13 | 97874549 | 98046374 |
| STK24 | chr13 | 99102454 | 99229396 |
| DOCK9 | chr13 | 99508151 | 99630338 |
| ARGLU1 | chr13 | 107195661 | 107220514 |
| COL4A2 | chr13 | 110959630 | 111165373 |
| CARKD | chr13 | 111267930 | 111292342 |
| ANKRD10 | chr13 | 111530886 | 111567416 |
| CUL4A | chr13 | 113863930 | 113919392 |
| TMCO3 | chr13 | 114145307 | 114204544 |
| RASA3 | chr13 | 114747193 | 114898095 |
| MPHOSPH8 | chr13 | 20207787 | 20247599 |
| PSPC1 | chr13 | 20248891 | 20357159 |
| ZMYM5 | chr13 | 20397623 | 20437776 |
| ZMYM2 | chr13 | 20598533 | 20665968 |
| N6AMT2 | chr13 | 21303072 | 21348057 |
| XPO4 | chr13 | 21351467 | 21476913 |
| SAP18 | chr13 | 21714652 | 21723224 |
| SKA3 | chr13 | 21727733 | 21750741 |
| MRP63 | chr13 | 21750371 | 21753220 |
| ZDHHC20 | chr13 | 21946709 | 22033508 |
| SPATA13 | chr13 | 24553838 | 24881212 |
| PARP4 | chr13 | 24995068 | 25086948 |
| CENPJ | chr13 | 25456411 | 25497027 |
| MTMR6 | chr13 | 25820340 | 25861704 |
| NUPL1 | chr13 | 25875665 | 25910478 |
| RNF6 | chr13 | 26786904 | 26796508 |
| WASF3 | chr13 | 27131839 | 27263082 |
| SNORD102 | chr13 | 27829200 | 27829272 |
| SNORA27 | chr13 | 27829537 | 27829663 |
| GTF3A | chr13 | 27998680 | 28009846 |
| MTIF3 | chr13 | 28009775 | 28015809 |
| LNX2 | chr13 | 28120049 | 28194720 |
| POLR1D | chr13 | 28194879 | 28241559 |
| PAN3-AS1 | chr13 | 28710979 | 28713311 |
| PAN3 | chr13 | 28712642 | 28869475 |
| FLT1 | chr13 | 28874482 | 29069265 |
| POMP | chr13 | 29233140 | 29253093 |
| UBL3 | chr13 | 30338544 | 30424820 |
| KATNAL1 | chr13 | 30776766 | 30881624 |
| HMGB1 | chr13 | 31032878 | 31191510 |
| MEDAG | chr13 | 31480311 | 31499709 |
| BRCA2 | chr13 | 32889616 | 32973809 |
| N4BP2L2 | chr13 | 33091029 | 33112932 |
| PDS5B | chr13 | 33160563 | 33352158 |
| STARD13 | chr13 | 33677271 | 33780187 |
| RFC3 | chr13 | 34392205 | 34411644 |
| SPG20 | chr13 | 36875774 | 36920646 |
| SPG20OS | chr13 | 36920567 | 36943872 |
| RFXAP | chr13 | 37393338 | 37403740 |
| ALG5 | chr13 | 37523907 | 37573504 |
| EXOSC8 | chr13 | 37574677 | 37583751 |
| SUPT20H | chr13 | 37583450 | 37633850 |
| UFM1 | chr13 | 38923941 | 38937143 |
| NHLRC3 | chr13 | 39612447 | 39624244 |
| MRPS31 | chr13 | 41303431 | 41345347 |
| ELF1 | chr13 | 41506054 | 41593508 |
| WBP4 | chr13 | 41635696 | 41658139 |
| KBTBD6 | chr13 | 41701708 | 41706936 |
| DGKH | chr13 | 42622842 | 42803891 |
| AKAP11 | chr13 | 42846288 | 42897403 |
| DNAJC15 | chr13 | 43597361 | 43683306 |
| TSC22D1 | chr13 | 45006278 | 45150701 |
| TSC22D1-AS1 | chr13 | 45150031 | 45154568 |
| NUFIP1 | chr13 | 45513383 | 45563613 |
| KIAA1704 | chr13 | 45563686 | 45604664 |
| GTF2F2 | chr13 | 45694630 | 45858239 |
| SNORA31 | chr13 | 45911614 | 45911744 |
| TPT1-AS1 | chr13 | 45915479 | 45965618 |
| COG3 | chr13 | 46039029 | 46110833 |
| ZC3H13 | chr13 | 46529804 | 46619648 |
| CPB2-AS1 | chr13 | 46626982 | 46675482 |
| LRCH1 | chr13 | 47127295 | 47319036 |
| SUCLA2 | chr13 | 48516790 | 48612125 |
| NUDT15 | chr13 | 48611702 | 48621282 |
| ITM2B | chr13 | 48807273 | 48836232 |
| LINC00441 | chr13 | 48870648 | 48877797 |
| RB1 | chr13 | 48877882 | 49056026 |
| LPAR6 | chr13 | 48985181 | 48987653 |
| FNDC3A | chr13 | 49550047 | 49783915 |
| EBPL | chr13 | 50234809 | 50265623 |
| MIR3613 | chr13 | 50570550 | 50570637 |
| TRIM13 | chr13 | 50571142 | 50592603 |
| DLEU1 | chr13 | 50656413 | 50679433 |
| INTS6 | chr13 | 51935700 | 52027275 |
| UTP14C | chr13 | 52586522 | 52607736 |
| ALG11 | chr13 | 52586522 | 52603780 |
| VPS36 | chr13 | 52986736 | 53024333 |
| CKAP2 | chr13 | 53029494 | 53050763 |
| HNRNPA1L2 | chr13 | 53191604 | 53217919 |
| DIAPH3-AS2 | chr13 | 60718831 | 60727639 |
| TDRD3 | chr13 | 60970590 | 61148013 |
| MZT1 | chr13 | 73282494 | 73301938 |
| BORA | chr13 | 73302041 | 73330328 |
| DIS3 | chr13 | 73329539 | 73356344 |
| PIBF1 | chr13 | 73356229 | 73590591 |
| UCHL3 | chr13 | 76123615 | 76180156 |
| FBXL3 | chr13 | 77579388 | 77601331 |
| MYCBP2 | chr13 | 77618791 | 77901177 |
| RNF219 | chr13 | 79188420 | 79233314 |
| RBM26-AS1 | chr13 | 79980443 | 79998468 |
| MIR19B1 | chr13 | 92002858 | 92003645 |
| MIR17 | chr13 | 92002858 | 92002942 |
| MIR18A | chr13 | 92003004 | 92003075 |
| MIR19A | chr13 | 92003144 | 92003226 |
| MIR20A | chr13 | 92003318 | 92003389 |
| TGDS | chr13 | 95226307 | 95248511 |
| ABCC4 | chr13 | 95672082 | 95953687 |
| DNAJC3 | chr13 | 96329392 | 96447243 |
| RAP2A | chr13 | 98086474 | 98120252 |
| STK24 | chr13 | 99102454 | 99174341 |
| UBAC2-AS1 | chr13 | 99848627 | 99853011 |
| UBAC2 | chr13 | 99853162 | 100038753 |
| MIR623 | chr13 | 100008384 | 100008482 |
| TM9SF2 | chr13 | 100153627 | 100216302 |
| TMTC4 | chr13 | 101256089 | 101327103 |
| ITGBL1 | chr13 | 102142302 | 102368796 |
| TPP2 | chr13 | 103249285 | 103331523 |
| ERCC5 | chr13 | 103498190 | 103528351 |
| EFNB2 | chr13 | 107142078 | 107187388 |
| LIG4 | chr13 | 108859791 | 108870716 |
| ABHD13 | chr13 | 108870762 | 108886603 |
| COL4A1 | chr13 | 110801309 | 110959496 |
| ING1 | chr13 | 111365609 | 111373421 |
| ARHGEF7 | chr13 | 111806060 | 111958081 |
| TUBGCP3 | chr13 | 113139327 | 113242481 |
| ATP11A | chr13 | 113344642 | 113541482 |
| PCID2 | chr13 | 113831852 | 113862962 |
| CUL4A | chr13 | 113863085 | 113919392 |
| LAMP1 | chr13 | 113951468 | 113977741 |
| DCUN1D2 | chr13 | 114110133 | 114145023 |
| TFDP1 | chr13 | 114239055 | 114295788 |
| TMEM255B | chr13 | 114462215 | 114514899 |
| CDC16 | chr13 | 115000361 | 115038150 |
| UPF3A | chr13 | 115047058 | 115071291 |
| CHAMP1 | chr13 | 115079964 | 115092803 |
| PNP | chr14 | 20937537 | 20946165 |
| HNRNPC | chr14 | 21677295 | 21737638 |
| SUPT16H | chr14 | 21819630 | 21852425 |
| DAD1 | chr14 | 23033806 | 23058143 |
| MMP14 | chr14 | 23305792 | 23316803 |
| AJUBA | chr14 | 23440409 | 23451848 |
| ACIN1 | chr14 | 23527773 | 23564823 |
| G2E3 | chr14 | 31028328 | 31089046 |
| STRN3 | chr14 | 31363004 | 31495607 |
| HECTD1 | chr14 | 31569323 | 31676689 |
| PSMA6 | chr14 | 35761573 | 35786682 |
| NFKBIA | chr14 | 35870715 | 35873960 |
| CLEC14A | chr14 | 38723204 | 38725575 |
| PNN | chr14 | 39644386 | 39652422 |
| PRPF39 | chr14 | 45553301 | 45584804 |
| MIS18BP1 | chr14 | 45672392 | 45722605 |
| RPS29 | chr14 | 50043389 | 50053134 |
| MGAT2 | chr14 | 50087488 | 50090199 |
| ARF6 | chr14 | 50359735 | 50363772 |
| MAP4K5 | chr14 | 50885210 | 50999376 |
| FRMD6 | chr14 | 52118575 | 52197444 |
| FERMT2 | chr14 | 53323988 | 53417815 |
| BMP4 | chr14 | 54416454 | 54423554 |
| CNIH | chr14 | 54893646 | 54908148 |
| SAMD4A | chr14 | 55034329 | 55260033 |
| SOCS4 | chr14 | 55493843 | 55516206 |
| MAPK1IP1L | chr14 | 55518361 | 55536912 |
| DLGAP5 | chr14 | 55614833 | 55658396 |
| PSMA3 | chr14 | 58711522 | 58738727 |
| FLJ31306 | chr14 | 58732082 | 58764855 |
| PCNXL4 | chr14 | 60558628 | 60601532 |
| PRKCH | chr14 | 61788514 | 62017698 |
| HIF1A | chr14 | 62162118 | 62214977 |
| RHOJ | chr14 | 63671101 | 63760230 |
| ZBTB1 | chr14 | 64971291 | 65000408 |
| ATP6V1D | chr14 | 67804580 | 67826720 |
| EIF2S1 | chr14 | 67827033 | 67853233 |
| ZFP36L1 | chr14 | 69254371 | 69260631 |
| ACTN1 | chr14 | 69340839 | 69354980 |
| SRSF5 | chr14 | 70193618 | 70238722 |
| RBM25 | chr14 | 73525220 | 73588076 |
| NUMB | chr14 | 73741917 | 73925286 |
| PNMA1 | chr14 | 74178485 | 74181128 |
| ELMSAN1 | chr14 | 74181824 | 74227001 |
| LTBP2 | chr14 | 74964885 | 75079034 |
| AREL1 | chr14 | 75127954 | 75179807 |
| YLPM1 | chr14 | 75230068 | 75304013 |
| DLST | chr14 | 75348593 | 75370450 |
| PGF | chr14 | 75408532 | 75422467 |
| IRF2BPL | chr14 | 77490885 | 77495042 |
| FLRT2 | chr14 | 85996487 | 86094270 |
| CALM1 | chr14 | 90863326 | 90874619 |
| TRIP11 | chr14 | 92434242 | 92506403 |
| BTBD7 | chr14 | 93703895 | 93799385 |
| DDX24 | chr14 | 94517267 | 94547558 |
| SNHG10 | chr14 | 95999248 | 96001209 |
| PAPOLA | chr14 | 96968712 | 97001935 |
| CCNK | chr14 | 99947738 | 99977852 |
| YY1 | chr14 | 100705101 | 100745371 |
| DYNC1H1 | chr14 | 102430864 | 102517135 |
| HSP90AA1 | chr14 | 102547074 | 102553512 |
| CDC42BPB | chr14 | 103398715 | 103523742 |
| EIF5 | chr14 | 103801285 | 103811361 |
| MARK3 | chr14 | 103851700 | 103970166 |
| BAG5 | chr14 | 104022880 | 104028655 |
| KLC1 | chr14 | 104029298 | 104152568 |
| INF2 | chr14 | 105155942 | 105185947 |
| CDCA4 | chr14 | 105475909 | 105487425 |
| MTA1 | chr14 | 105886185 | 105937057 |
| CCNB1IP1 | chr14 | 20779526 | 20801471 |
| RPPH1 | chr14 | 20811229 | 20811570 |
| PARP2 | chr14 | 20811772 | 20826062 |
| OSGEP | chr14 | 20915206 | 20923267 |
| APEX1 | chr14 | 20923289 | 20925931 |
| TMEM55B | chr14 | 20926011 | 20929637 |
| RNASE4 | chr14 | 21152335 | 21168758 |
| ANG | chr14 | 21152335 | 21162345 |
| CHD8 | chr14 | 21853352 | 21899867 |
| RAB2B | chr14 | 21927178 | 21945132 |
| TOX4 | chr14 | 21945334 | 21967319 |
| METTL3 | chr14 | 21966281 | 21979457 |
| OXA1L | chr14 | 23235730 | 23240998 |
| MRPL52 | chr14 | 23299091 | 23304246 |
| LRP10 | chr14 | 23340959 | 23347291 |
| RBM23 | chr14 | 23369853 | 23388396 |
| PRMT5 | chr14 | 23389732 | 23398661 |
| AJUBA | chr14 | 23440409 | 23446432 |
| PSMB5 | chr14 | 23495059 | 23504354 |
| C14orf119 | chr14 | 23564682 | 23569665 |
| BCL2L2 | chr14 | 23775970 | 23780968 |
| BCL2L2-PABPN1 | chr14 | 23775970 | 23795394 |
| PABPN1 | chr14 | 23789396 | 23794545 |
| NGDN | chr14 | 23938897 | 23947402 |
| DCAF11 | chr14 | 24583985 | 24594451 |
| EMC9 | chr14 | 24608173 | 24610797 |
| PSME2 | chr14 | 24612573 | 24615855 |
| RNF31 | chr14 | 24616658 | 24630547 |
| TM9SF1 | chr14 | 24657924 | 24683357 |
| CHMP4A | chr14 | 24678786 | 24685276 |
| NEDD8-MDP1 | chr14 | 24682614 | 24701576 |
| NEDD8 | chr14 | 24686056 | 24701576 |
| GMPR2 | chr14 | 24701647 | 24708445 |
| TINF2 | chr14 | 24708850 | 24711880 |
| NOP9 | chr14 | 24769097 | 24774374 |
| NFATC4 | chr14 | 24836144 | 24848810 |
| CBLN3 | chr14 | 24895739 | 24898731 |
| KHNYN | chr14 | 24899140 | 24910547 |
| SDR39U1 | chr14 | 24908971 | 24912007 |
| SCFD1 | chr14 | 31091459 | 31205033 |
| MIR624 | chr14 | 31483851 | 31483948 |
| AP4S1 | chr14 | 31495848 | 31565656 |
| ARHGAP5 | chr14 | 32546494 | 32628934 |
| SPTSSA | chr14 | 34902143 | 34931468 |
| SNX6 | chr14 | 35030617 | 35099315 |
| CFL2 | chr14 | 35179587 | 35184029 |
| BAZ1A | chr14 | 35221936 | 35344853 |
| SRP54 | chr14 | 35452103 | 35498773 |
| FAM177A1 | chr14 | 35515609 | 35552589 |
| PPP2R3C | chr14 | 35554677 | 35591519 |
| KIAA0391 | chr14 | 35591526 | 35786682 |
| MBIP | chr14 | 36767763 | 36789882 |
| MIPOL1 | chr14 | 37667117 | 38020464 |
| SEC23A | chr14 | 39501122 | 39572437 |
| TRAPPC6B | chr14 | 39617014 | 39639634 |
| LOC100288846 | chr14 | 39734906 | 39736265 |
| CTAGE5 | chr14 | 39745429 | 39820397 |
| FBXO33 | chr14 | 39865576 | 39901704 |
| KLHL28 | chr14 | 45393526 | 45430335 |
| FAM179B | chr14 | 45431415 | 45543634 |
| FKBP3 | chr14 | 45584801 | 45604009 |
| FANCM | chr14 | 45605135 | 45670093 |
| LRR1 | chr14 | 50065414 | 50081390 |
| RPL36AL | chr14 | 50085405 | 50087403 |
| DNAAF2 | chr14 | 50091891 | 50101948 |
| KLHDC2 | chr14 | 50234786 | 50249856 |
| NEMF | chr14 | 50250531 | 50319539 |
| VCPKMT | chr14 | 50575349 | 50583297 |
| METTL21D | chr14 | 50575349 | 50583297 |
| SOS2 | chr14 | 50583845 | 50698099 |
| ATL1 | chr14 | 50999799 | 51099784 |
| SAV1 | chr14 | 51100297 | 51135071 |
| NIN | chr14 | 51186480 | 51297839 |
| TMX1 | chr14 | 51706885 | 51724372 |
| FRMD6-AS1 | chr14 | 52116235 | 52118462 |
| C14orf166 | chr14 | 52456227 | 52471420 |
| ERO1L | chr14 | 53108604 | 53162419 |
| PSMC6 | chr14 | 53173895 | 53194716 |
| STYX | chr14 | 53196882 | 53241705 |
| GNPNAT1 | chr14 | 53241910 | 53258386 |
| MIR5580 | chr14 | 54415144 | 54415202 |
| GMFB | chr14 | 54941208 | 54955744 |
| CGRRF1 | chr14 | 54976586 | 55005334 |
| WDHD1 | chr14 | 55405655 | 55493819 |
| FBXO34 | chr14 | 55817506 | 55893433 |
| ATG14 | chr14 | 55833108 | 55878576 |
| KTN1-AS1 | chr14 | 56042874 | 56046810 |
| KTN1 | chr14 | 56046924 | 56151302 |
| TMEM260 | chr14 | 57046510 | 57116232 |
| EXOC5 | chr14 | 57669193 | 57735617 |
| AP5M1 | chr14 | 57735605 | 57756797 |
| NAA30 | chr14 | 57857270 | 57879466 |
| ACTR10 | chr14 | 58666832 | 58702353 |
| ARID4A | chr14 | 58765221 | 58840451 |
| TIMM9 | chr14 | 58875369 | 58894232 |
| KIAA0586 | chr14 | 58894102 | 59015549 |
| DAAM1 | chr14 | 59655380 | 59838123 |
| L3HYPDH | chr14 | 59939405 | 59951073 |
| JKAMP | chr14 | 59951286 | 59972081 |
| PPM1A | chr14 | 60715965 | 60765805 |
| MNAT1 | chr14 | 61201458 | 61435398 |
| TRMT5 | chr14 | 61438166 | 61447782 |
| SLC38A6 | chr14 | 61447831 | 61519703 |
| HIF1A-AS2 | chr14 | 62213756 | 62215807 |
| SNAPC1 | chr14 | 62229074 | 62263146 |
| PPP2R5E | chr14 | 63841354 | 64010079 |
| WDR89 | chr14 | 64063756 | 64108641 |
| MTHFD1 | chr14 | 64854758 | 64926725 |
| ZBTB25 | chr14 | 64953554 | 64971931 |
| CHURC1 | chr14 | 65381078 | 65402084 |
| CHURC1-FNTB | chr14 | 65381078 | 65529373 |
| MAX | chr14 | 65541841 | 65569262 |
| MPP5 | chr14 | 67708011 | 67802778 |
| VTI1B | chr14 | 68117866 | 68141602 |
| RDH11 | chr14 | 68143518 | 68162510 |
| ZFYVE26 | chr14 | 68213236 | 68283306 |
| ACTN1-AS1 | chr14 | 69446398 | 69454180 |
| DCAF5 | chr14 | 69517636 | 69619914 |
| ERH | chr14 | 69846839 | 69865021 |
| SLC39A9 | chr14 | 69865384 | 69929107 |
| KIAA0247 | chr14 | 70078309 | 70181861 |
| LOC100289511 | chr14 | 70232999 | 70234430 |
| COX16 | chr14 | 70791797 | 70826448 |
| MED6 | chr14 | 71050956 | 71067384 |
| PCNX | chr14 | 71374121 | 71582099 |
| DPF3 | chr14 | 73128567 | 73360809 |
| PSEN1 | chr14 | 73603524 | 73690399 |
| C14orf169 | chr14 | 73957643 | 73960105 |
| ACOT2 | chr14 | 74035762 | 74042362 |
| MIR4505 | chr14 | 74225449 | 74225522 |
| ZNF410 | chr14 | 74353317 | 74398991 |
| ABCD4 | chr14 | 74751979 | 74769767 |
| NPC2 | chr14 | 74946642 | 74960084 |
| FCF1 | chr14 | 75179849 | 75203390 |
| EIF2B2 | chr14 | 75469611 | 75476294 |
| ACYP1 | chr14 | 75519927 | 75530736 |
| NEK9 | chr14 | 75548817 | 75593778 |
| TMED10 | chr14 | 75598170 | 75643349 |
| LOC731223 | chr14 | 75761106 | 75763111 |
| C14orf1 | chr14 | 76117232 | 76127538 |
| TTLL5 | chr14 | 76127550 | 76421425 |
| IFT43 | chr14 | 76452095 | 76550416 |
| GPATCH2L | chr14 | 76619988 | 76643033 |
| KIAA1737 | chr14 | 77564577 | 77583630 |
| POMT2 | chr14 | 77741298 | 77787225 |
| GSTZ1 | chr14 | 77787229 | 77797940 |
| VIPAS39 | chr14 | 77893017 | 77923983 |
| AHSA1 | chr14 | 77924372 | 77935815 |
| SPTLC2 | chr14 | 77972339 | 78083110 |
| ALKBH1 | chr14 | 78138748 | 78174356 |
| SLIRP | chr14 | 78174413 | 78183941 |
| SNW1 | chr14 | 78183943 | 78227497 |
| C14orf178 | chr14 | 78227172 | 78236085 |
| GTF2A1 | chr14 | 81641795 | 81687296 |
| SEL1L | chr14 | 81937890 | 82000205 |
| PTPN21 | chr14 | 88932121 | 89021123 |
| ZC3H14 | chr14 | 89029252 | 89079853 |
| FOXN3 | chr14 | 89622515 | 90085494 |
| EFCAB11 | chr14 | 90389669 | 90420867 |
| TDP1 | chr14 | 90422245 | 90511108 |
| PSMC1 | chr14 | 90722893 | 90738966 |
| NRDE2 | chr14 | 90744397 | 90798481 |
| TTC7B | chr14 | 91006931 | 91282761 |
| C14orf159 | chr14 | 91580356 | 91691703 |
| ATXN3 | chr14 | 92524895 | 92572965 |
| NDUFB1 | chr14 | 92582467 | 92588153 |
| CPSF2 | chr14 | 92588297 | 92630543 |
| GOLGA5 | chr14 | 93260649 | 93306304 |
| MOAP1 | chr14 | 93648540 | 93651249 |
| TMEM251 | chr14 | 93651374 | 93653431 |
| C14orf142 | chr14 | 93669236 | 93673459 |
| UBR7 | chr14 | 93673400 | 93695561 |
| UNC79 | chr14 | 93799564 | 94173689 |
| IFI27L1 | chr14 | 94547638 | 94569060 |
| DICER1 | chr14 | 95552564 | 95623759 |
| MIR3173 | chr14 | 95604255 | 95604323 |
| DICER1-AS1 | chr14 | 95624024 | 95646270 |
| SNHG10 | chr14 | 95999248 | 96001209 |
| GLRX5 | chr14 | 96001322 | 96011055 |
| ATG2B | chr14 | 96747594 | 96829678 |
| GSKIP | chr14 | 96829788 | 96853627 |
| VRK1 | chr14 | 97263683 | 97347951 |
| SETD3 | chr14 | 99864082 | 99946581 |
| SLC25A29 | chr14 | 100757452 | 100772860 |
| WARS | chr14 | 100800124 | 100841927 |
| WDR25 | chr14 | 100842832 | 100996640 |
| PPP2R5C | chr14 | 102276139 | 102394328 |
| HSP90AA1 | chr14 | 102547074 | 102606086 |
| WDR20 | chr14 | 102606188 | 102681341 |
| CINP | chr14 | 102814615 | 102829253 |
| TECPR2 | chr14 | 102829299 | 102968818 |
| RCOR1 | chr14 | 103058995 | 103196913 |
| TRAF3 | chr14 | 103243815 | 103377837 |
| SNORA28 | chr14 | 103804185 | 103804311 |
| TRMT61A | chr14 | 103995508 | 104003410 |
| APOPT1 | chr14 | 104029298 | 104057236 |
| XRCC3 | chr14 | 104163953 | 104181823 |
| ZFYVE21 | chr14 | 104182080 | 104200005 |
| C14orf2 | chr14 | 104378624 | 104387903 |
| SIVA1 | chr14 | 105219469 | 105223822 |
| AKT1 | chr14 | 105235686 | 105259938 |
| CEP170B | chr14 | 105331649 | 105363107 |
| JAG2 | chr14 | 105607317 | 105635161 |
| BRF1 | chr14 | 105675622 | 105767329 |
| BTBD6 | chr14 | 105714878 | 105717430 |
| CRIP2 | chr14 | 105942195 | 105946507 |
| NIPA2 | chr15 | 23004683 | 23034427 |
| UBE3A | chr15 | 25582395 | 25684175 |
| TJP1 | chr15 | 29992356 | 30114706 |
| ARHGAP11B | chr15 | 30918878 | 30977810 |
| MTMR10 | chr15 | 31231143 | 31242539 |
| ARHGAP11A | chr15 | 32907690 | 32931868 |
| ZNF770 | chr15 | 35270541 | 35280497 |
| THBS1 | chr15 | 39873279 | 39889668 |
| BUB1B | chr15 | 40453209 | 40513337 |
| NUSAP1 | chr15 | 41624891 | 41673248 |
| MAPKBP1 | chr15 | 42066631 | 42120053 |
| EHD4 | chr15 | 42191638 | 42264755 |
| SNAP23 | chr15 | 42787503 | 42825259 |
| PDIA3 | chr15 | 44038589 | 44064804 |
| CTDSPL2 | chr15 | 44719578 | 44819429 |
| EIF3J | chr15 | 44829265 | 44855001 |
| B2M | chr15 | 45003684 | 45008550 |
| COPS2 | chr15 | 49417470 | 49447854 |
| GABPB1 | chr15 | 50569388 | 50647605 |
| FLJ10038 | chr15 | 50641134 | 50647076 |
| GABPB1-AS1 | chr15 | 50646370 | 50650503 |
| TMOD3 | chr15 | 52121824 | 52204331 |
| MAPK6 | chr15 | 52311410 | 52358462 |
| ARPP19 | chr15 | 52839431 | 52861213 |
| RSL24D1 | chr15 | 55473511 | 55489231 |
| RNF111 | chr15 | 59279864 | 59389618 |
| MYO1E | chr15 | 59428167 | 59665071 |
| BNIP2 | chr15 | 59955061 | 59981642 |
| ANXA2 | chr15 | 60639349 | 60690185 |
| TPM1 | chr15 | 63334837 | 63364113 |
| USP3 | chr15 | 63796709 | 63886839 |
| RAB11A | chr15 | 66161796 | 66184329 |
| MAP2K1 | chr15 | 66679210 | 66783882 |
| RPL4 | chr15 | 66791652 | 66816870 |
| SMAD3 | chr15 | 67358194 | 67487533 |
| FEM1B | chr15 | 68580749 | 68588021 |
| ANP32A | chr15 | 69070874 | 69113261 |
| GLCE | chr15 | 69452972 | 69564544 |
| RPLP1 | chr15 | 69745158 | 69747884 |
| UACA | chr15 | 70946892 | 70994620 |
| PKM | chr15 | 72491369 | 72523727 |
| ARIH1 | chr15 | 72766666 | 72878896 |
| PML | chr15 | 74287013 | 74340155 |
| CLK3 | chr15 | 74907334 | 74922542 |
| CSK | chr15 | 75074424 | 75095539 |
| MIR4513 | chr15 | 75081012 | 75081098 |
| C15orf39 | chr15 | 75491232 | 75504510 |
| PEAK1 | chr15 | 77400497 | 77712446 |
| MORF4L1 | chr15 | 79165122 | 79190081 |
| MESDC1 | chr15 | 81293294 | 81296345 |
| BTBD1 | chr15 | 83685180 | 83736106 |
| HDGFRP3 | chr15 | 83806803 | 83876770 |
| SEC11A | chr15 | 85212767 | 85259691 |
| ZNF592 | chr15 | 85291817 | 85349663 |
| AEN | chr15 | 89164526 | 89175512 |
| POLG | chr15 | 89859535 | 89878026 |
| ANPEP | chr15 | 90328125 | 90358072 |
| NGRN | chr15 | 90808894 | 90815443 |
| IQGAP1 | chr15 | 90931472 | 91045475 |
| PRC1 | chr15 | 91509267 | 91537881 |
| LOC100507217 | chr15 | 93426072 | 93441977 |
| CHD2 | chr15 | 93443550 | 93571237 |
| MIR3175 | chr15 | 93447628 | 93447705 |
| NR2F2 | chr15 | 96874110 | 96883492 |
| MIR1469 | chr15 | 96876489 | 96876536 |
| CHSY1 | chr15 | 101715927 | 101792137 |
| SNRPA1 | chr15 | 101821714 | 101835460 |
| CYFIP1 | chr15 | 22892683 | 23003603 |
| SNRPN | chr15 | 25200134 | 25223729 |
| SNURF | chr15 | 25200134 | 25223729 |
| HERC2 | chr15 | 28356182 | 28567298 |
| EMC7 | chr15 | 34376223 | 34394053 |
| PGBD4 | chr15 | 34394273 | 34396591 |
| KATNBL1 | chr15 | 34432874 | 34502297 |
| EMC4 | chr15 | 34517244 | 34522352 |
| SLC12A6 | chr15 | 34522196 | 34629961 |
| NOP10 | chr15 | 34633916 | 34635362 |
| AQR | chr15 | 35148551 | 35261995 |
| DPH6 | chr15 | 35812473 | 35838404 |
| DPH6-AS1 | chr15 | 35838395 | 36151202 |
| MEIS2 | chr15 | 37183221 | 37392341 |
| SPRED1 | chr15 | 38545051 | 38649450 |
| FAM98B | chr15 | 38746327 | 38777063 |
| C15orf54 | chr15 | 39542884 | 39547048 |
| GPR176 | chr15 | 40091222 | 40213093 |
| EIF2AK4 | chr15 | 40226346 | 40327797 |
| SRP14 | chr15 | 40327890 | 40331389 |
| SRP14-AS1 | chr15 | 40331511 | 40359710 |
| C15orf52 | chr15 | 40623652 | 40633168 |
| KNSTRN | chr15 | 40674921 | 40686489 |
| RPUSD2 | chr15 | 40861536 | 40866661 |
| CASC5 | chr15 | 40886446 | 40954881 |
| RAD51-AS1 | chr15 | 40985952 | 40987303 |
| RAD51 | chr15 | 40987326 | 41024356 |
| VPS18 | chr15 | 41186627 | 41196173 |
| INO80 | chr15 | 41271078 | 41408340 |
| CHP1 | chr15 | 41523436 | 41574083 |
| OIP5-AS1 | chr15 | 41576200 | 41591795 |
| OIP5 | chr15 | 41601465 | 41624819 |
| RTF1 | chr15 | 41709301 | 41775761 |
| MGA | chr15 | 41952609 | 42062141 |
| VPS39 | chr15 | 42450898 | 42500502 |
| TMEM87A | chr15 | 42502725 | 42565755 |
| GANC | chr15 | 42566365 | 42645864 |
| LRRC57 | chr15 | 42834719 | 42841000 |
| HAUS2 | chr15 | 42841010 | 42862190 |
| UBR1 | chr15 | 43235097 | 43398286 |
| CATSPER2P1 | chr15 | 44028145 | 44038496 |
| SERF2 | chr15 | 44084173 | 44088287 |
| SERF2-C15ORF63 | chr15 | 44084173 | 44094769 |
| MIR1282 | chr15 | 44085856 | 44085957 |
| MFAP1 | chr15 | 44096732 | 44116951 |
| WDR76 | chr15 | 44119111 | 44160617 |
| FRMD5 | chr15 | 44165729 | 44487429 |
| CASC4 | chr15 | 44580908 | 44707959 |
| EIF3J-AS1 | chr15 | 44826702 | 44829121 |
| SPATA5L1 | chr15 | 45694518 | 45713616 |
| BLOC1S6 | chr15 | 45879416 | 45901909 |
| MYEF2 | chr15 | 48431628 | 48470558 |
| DUT | chr15 | 48623620 | 48635570 |
| FBN1 | chr15 | 48700502 | 48937985 |
| EID1 | chr15 | 49170289 | 49172380 |
| SECISBP2L | chr15 | 49280834 | 49338760 |
| NDUFAF4P1 | chr15 | 49448494 | 49450822 |
| GALK2 | chr15 | 49462421 | 49622002 |
| FAM227B | chr15 | 49620591 | 49913118 |
| DTWD1 | chr15 | 49913225 | 49937333 |
| USP8 | chr15 | 50716578 | 50793277 |
| TRPM7 | chr15 | 50849351 | 50979012 |
| SPPL2A | chr15 | 50999736 | 51057910 |
| LEO1 | chr15 | 52230221 | 52263958 |
| MYO5A | chr15 | 52599479 | 52821247 |
| FAM214A | chr15 | 52873517 | 52944237 |
| PIGB | chr15 | 55611132 | 55647846 |
| NEDD4 | chr15 | 56119121 | 56285835 |
| ZNF280D | chr15 | 56945018 | 57210697 |
| LOC145783 | chr15 | 57178367 | 57210697 |
| TCF12 | chr15 | 57210832 | 57580714 |
| POLR2M | chr15 | 57998900 | 58009755 |
| ADAM10 | chr15 | 58888509 | 59042177 |
| SLTM | chr15 | 59171243 | 59225852 |
| RNF111 | chr15 | 59280344 | 59389618 |
| CCNB2 | chr15 | 59397283 | 59417244 |
| GTF2A2 | chr15 | 59930260 | 59949737 |
| NARG2 | chr15 | 60711807 | 60771359 |
| VPS13C | chr15 | 62156504 | 62352664 |
| C2CD4B | chr15 | 62455736 | 62457482 |
| TPM1 | chr15 | 63334837 | 63364113 |
| RPS27L | chr15 | 63445538 | 63449741 |
| RAB8B | chr15 | 63481727 | 63559973 |
| HERC1 | chr15 | 63900816 | 64126147 |
| FAM96A | chr15 | 64366516 | 64386207 |
| SNX1 | chr15 | 64388082 | 64436433 |
| PPIB | chr15 | 64451472 | 64455354 |
| CSNK1G1 | chr15 | 64457715 | 64648442 |
| KIAA0101 | chr15 | 64657210 | 64673702 |
| TRIP4 | chr15 | 64680002 | 64747502 |
| ZNF609 | chr15 | 64791618 | 64978266 |
| OAZ2 | chr15 | 64979772 | 64995462 |
| PLEKHO2 | chr15 | 65134081 | 65160201 |
| SPG21 | chr15 | 65255362 | 65282251 |
| PDCD7 | chr15 | 65409716 | 65426174 |
| CLPX | chr15 | 65442783 | 65477563 |
| DPP8 | chr15 | 65737997 | 65809608 |
| PTPLAD1 | chr15 | 65822826 | 65870693 |
| VWA9 | chr15 | 65871095 | 65903486 |
| DENND4A | chr15 | 65952956 | 66084631 |
| TIPIN | chr15 | 66629007 | 66649054 |
| SNAPC5 | chr15 | 66782665 | 66790146 |
| SNORD18C | chr15 | 66793589 | 66793656 |
| SNORD18B | chr15 | 66794359 | 66794429 |
| SNORD16 | chr15 | 66795148 | 66795248 |
| SNORD18A | chr15 | 66795582 | 66795652 |
| ZWILCH | chr15 | 66797430 | 66841822 |
| SMAD6 | chr15 | 66994673 | 67074337 |
| SMAD3 | chr15 | 67458492 | 67487533 |
| AAGAB | chr15 | 67493012 | 67547074 |
| C15orf61 | chr15 | 67813521 | 67819641 |
| PIAS1 | chr15 | 68346571 | 68480404 |
| CLN6 | chr15 | 68499329 | 68549444 |
| MIR4312 | chr15 | 69094188 | 69094264 |
| ANP32A-IT1 | chr15 | 69096159 | 69099440 |
| LINC00277 | chr15 | 69373189 | 69388163 |
| KIF23 | chr15 | 69706626 | 69740764 |
| TLE3 | chr15 | 70340542 | 70390256 |
| MIR629 | chr15 | 70371710 | 70371807 |
| UACA | chr15 | 70946892 | 71055850 |
| LRRC49 | chr15 | 71145617 | 71342436 |
| THAP10 | chr15 | 71173680 | 71184772 |
| MYO9A | chr15 | 72181812 | 72338975 |
| ADPGK | chr15 | 73043707 | 73076126 |
| NPTN | chr15 | 73852343 | 73925753 |
| CD276 | chr15 | 73976621 | 74006859 |
| STOML1 | chr15 | 74275558 | 74286963 |
| EDC3 | chr15 | 74922898 | 74988386 |
| SCAMP2 | chr15 | 75136072 | 75146460 |
| FAM219B | chr15 | 75192327 | 75199462 |
| COX5A | chr15 | 75212616 | 75230495 |
| RPP25 | chr15 | 75247442 | 75249775 |
| PPCDC | chr15 | 75315926 | 75343067 |
| MAN2C1 | chr15 | 75648132 | 75660968 |
| SIN3A | chr15 | 75661719 | 75744087 |
| PTPN9 | chr15 | 75759461 | 75871625 |
| IMP3 | chr15 | 75931428 | 75941047 |
| UBE2Q2 | chr15 | 76135621 | 76193388 |
| FBXO22 | chr15 | 76196199 | 76227608 |
| ETFA | chr15 | 76508628 | 76603810 |
| TSPAN3 | chr15 | 77336359 | 77363570 |
| HMG20A | chr15 | 77713242 | 77777945 |
| TBC1D2B | chr15 | 78287326 | 78369994 |
| WDR61 | chr15 | 78575577 | 78591940 |
| IREB2 | chr15 | 78730517 | 78793798 |
| PSMA4 | chr15 | 78832785 | 78841563 |
| TMED3 | chr15 | 79603490 | 79615189 |
| ZFAND6 | chr15 | 80352271 | 80430735 |
| ABHD17C | chr15 | 80987651 | 81047962 |
| MESDC2 | chr15 | 81268094 | 81282205 |
| MEX3B | chr15 | 82334118 | 82338484 |
| RPS17L | chr15 | 82821160 | 82824865 |
| RPS17 | chr15 | 82821160 | 82824865 |
| WHAMM | chr15 | 83477972 | 83503613 |
| MIR4515 | chr15 | 83736086 | 83736167 |
| TM6SF1 | chr15 | 83776323 | 83806111 |
| WDR73 | chr15 | 85186011 | 85197521 |
| ALPK3 | chr15 | 85359910 | 85416713 |
| PDE8A | chr15 | 85525204 | 85682376 |
| AKAP13 | chr15 | 85923846 | 86292589 |
| MRPL46 | chr15 | 89002707 | 89009004 |
| MRPS11 | chr15 | 89010683 | 89021861 |
| ISG20 | chr15 | 89182038 | 89198879 |
| ABHD2 | chr15 | 89631380 | 89745591 |
| FANCI | chr15 | 89787193 | 89860362 |
| TICRR | chr15 | 90118817 | 90171253 |
| AP3S2 | chr15 | 90373830 | 90437617 |
| ZNF710 | chr15 | 90544751 | 90625432 |
| BLM | chr15 | 91260578 | 91358686 |
| FURIN | chr15 | 91411884 | 91426687 |
| FES | chr15 | 91427664 | 91439006 |
| UNC45A | chr15 | 91478214 | 91497323 |
| FAM174B | chr15 | 93160678 | 93199031 |
| LOC400456 | chr15 | 95822518 | 95870329 |
| IGF1R | chr15 | 99192760 | 99507759 |
| LRRC28 | chr15 | 99791651 | 99927280 |
| MEF2A | chr15 | 100106132 | 100256629 |
| LINS | chr15 | 101109427 | 101113767 |
| ASB7 | chr15 | 101142754 | 101191904 |
| VIMP | chr15 | 101811213 | 101817700 |
| TM2D3 | chr15 | 102182048 | 102190895 |
| LUC7L | chr16 | 238973 | 259772 |
| SOLH | chr16 | 577855 | 604636 |
| UBE2I | chr16 | 1359627 | 1377019 |
| TMEM204 | chr16 | 1583657 | 1605581 |
| HN1L | chr16 | 1728277 | 1752073 |
| NDUFB10 | chr16 | 2009516 | 2011976 |
| RPS2 | chr16 | 2012061 | 2014814 |
| ZNF598 | chr16 | 2047652 | 2059822 |
| SNORD60 | chr16 | 2205023 | 2205106 |
| TRAF7 | chr16 | 2205798 | 2228130 |
| RNPS1 | chr16 | 2303099 | 2317604 |
| ATP6V0C | chr16 | 2563870 | 2570224 |
| SRRM2 | chr16 | 2802329 | 2821413 |
| NAA60 | chr16 | 3507991 | 3536963 |
| CREBBP | chr16 | 3775055 | 3930121 |
| PMM2 | chr16 | 8891669 | 8943194 |
| USP7 | chr16 | 8985950 | 9057341 |
| C16orf72 | chr16 | 9185536 | 9213555 |
| ZC3H7A | chr16 | 11844441 | 11891114 |
| GSPT1 | chr16 | 11961984 | 12010519 |
| BFAR | chr16 | 14726667 | 14763093 |
| ABCC1 | chr16 | 16043433 | 16236930 |
| RPS15A | chr16 | 18794276 | 18801656 |
| ARL6IP1 | chr16 | 18802990 | 18812692 |
| SMG1 | chr16 | 18816174 | 18937726 |
| ITPRIPL2 | chr16 | 19125253 | 19132952 |
| DCUN1D3 | chr16 | 20869395 | 20911561 |
| LYRM1 | chr16 | 20912425 | 20936328 |
| LOC100271836 | chr16 | 21458003 | 21513602 |
| SLC7A5P2 | chr16 | 21529229 | 21531765 |
| CDR2 | chr16 | 22357256 | 22385938 |
| SMG1P1 | chr16 | 22448328 | 22503541 |
| PLK1 | chr16 | 23690200 | 23701688 |
| RBBP6 | chr16 | 24550907 | 24584183 |
| TNRC6A | chr16 | 24741048 | 24837547 |
| ARHGAP17 | chr16 | 24930711 | 24968857 |
| IL4R | chr16 | 27325229 | 27376099 |
| XPO6 | chr16 | 28109297 | 28223239 |
| ATXN2L | chr16 | 28834368 | 28848558 |
| SLC7A5P1 | chr16 | 29624423 | 29625038 |
| KIF22 | chr16 | 29802288 | 29816706 |
| TAOK2 | chr16 | 29985187 | 29999726 |
| ALDOA | chr16 | 30064471 | 30081741 |
| PPP4C | chr16 | 30087349 | 30096695 |
| TBC1D10B | chr16 | 30368421 | 30381522 |
| FUS | chr16 | 31191430 | 31206192 |
| ZNF267 | chr16 | 31885078 | 31928629 |
| MMP2 | chr16 | 55513080 | 55540586 |
| NUDT21 | chr16 | 56463047 | 56485261 |
| MT2A | chr16 | 56642477 | 56643409 |
| HERPUD1 | chr16 | 56969862 | 56977793 |
| RSPRY1 | chr16 | 57220244 | 57272947 |
| CNOT1 | chr16 | 58553849 | 58663790 |
| GOT2 | chr16 | 58741034 | 58768246 |
| CDH5 | chr16 | 66400524 | 66438689 |
| DYNC1LI2 | chr16 | 66754798 | 66785525 |
| CBFB | chr16 | 67063049 | 67134958 |
| E2F4 | chr16 | 67226067 | 67232821 |
| CTCF | chr16 | 67596309 | 67673088 |
| SLC12A4 | chr16 | 67977376 | 68002597 |
| SNTB2 | chr16 | 69221049 | 69342955 |
| SF3B3 | chr16 | 70557690 | 70611571 |
| AP1G1 | chr16 | 71762904 | 71842976 |
| IST1 | chr16 | 71928310 | 71964540 |
| PSMD7 | chr16 | 74330672 | 74340186 |
| BCAR1 | chr16 | 75262927 | 75299905 |
| TERF2IP | chr16 | 75681634 | 75691341 |
| CMC2 | chr16 | 81009698 | 81040502 |
| CMIP | chr16 | 81478774 | 81745367 |
| USP10 | chr16 | 84733554 | 84813527 |
| EMC8 | chr16 | 85812230 | 85833148 |
| FOXC2 | chr16 | 86600856 | 86602537 |
| MAP1LC3B | chr16 | 87425800 | 87438380 |
| SLC7A5 | chr16 | 87863628 | 87903100 |
| BANP | chr16 | 87985037 | 88110924 |
| PIEZO1 | chr16 | 88781745 | 88851372 |
| ANKRD11 | chr16 | 89334028 | 89556969 |
| LOC100287036 | chr16 | 89387540 | 89391518 |
| RPL13 | chr16 | 89627118 | 89633237 |
| RHBDF1 | chr16 | 108057 | 122629 |
| RAB11FIP3 | chr16 | 475667 | 572481 |
| LINC00235 | chr16 | 576846 | 577407 |
| MIR5587 | chr16 | 585315 | 585368 |
| MIR3176 | chr16 | 593276 | 593366 |
| RAB40C | chr16 | 639356 | 679273 |
| STUB1 | chr16 | 730114 | 732768 |
| JMJD8 | chr16 | 731666 | 734439 |
| RPUSD1 | chr16 | 834973 | 837971 |
| CHTF18 | chr16 | 838621 | 848074 |
| TSR3 | chr16 | 1399240 | 1401873 |
| GNPTG | chr16 | 1401899 | 1413352 |
| UNKL | chr16 | 1413205 | 1429684 |
| C16orf91 | chr16 | 1469744 | 1470801 |
| TELO2 | chr16 | 1543351 | 1560460 |
| IFT140 | chr16 | 1560427 | 1662109 |
| CRAMP1L | chr16 | 1664640 | 1727909 |
| MAPK8IP3 | chr16 | 1756220 | 1820318 |
| MRPS34 | chr16 | 1821895 | 1823140 |
| EME2 | chr16 | 1823228 | 1826340 |
| NUBP2 | chr16 | 1832932 | 1839192 |
| HAGH | chr16 | 1859103 | 1877195 |
| FAHD1 | chr16 | 1877224 | 1890203 |
| SNORA10 | chr16 | 2012334 | 2012467 |
| SNORA64 | chr16 | 2012973 | 2013107 |
| SNHG9 | chr16 | 2014996 | 2015505 |
| SNORA78 | chr16 | 2015184 | 2015311 |
| RNF151 | chr16 | 2016874 | 2018976 |
| TBL3 | chr16 | 2022063 | 2028751 |
| SLC9A3R2 | chr16 | 2083264 | 2089027 |
| PKD1 | chr16 | 2138710 | 2185899 |
| MIR4516 | chr16 | 2183119 | 2183205 |
| MIR3180-5 | chr16 | 2185977 | 2186130 |
| PGP | chr16 | 2261602 | 2264822 |
| E4F1 | chr16 | 2273566 | 2285743 |
| MIR3677 | chr16 | 2320713 | 2320773 |
| CCNF | chr16 | 2479394 | 2508859 |
| AMDHD2 | chr16 | 2570362 | 2580955 |
| PDPK1 | chr16 | 2588164 | 2653191 |
| LOC652276 | chr16 | 2653384 | 2680495 |
| KCTD5 | chr16 | 2732494 | 2759031 |
| SRRM2-AS1 | chr16 | 2787076 | 2802601 |
| TCEB2 | chr16 | 2821414 | 2827297 |
| TNFRSF12A | chr16 | 3070390 | 3072383 |
| HCFC1R1 | chr16 | 3072625 | 3074287 |
| IL32 | chr16 | 3115312 | 3119668 |
| ZNF213 | chr16 | 3185056 | 3192805 |
| ZNF263 | chr16 | 3339392 | 3351400 |
| ZNF597 | chr16 | 3486109 | 3493490 |
| NAA60 | chr16 | 3493667 | 3536963 |
| TRAP1 | chr16 | 3708037 | 3728305 |
| CORO7-PAM16 | chr16 | 4390251 | 4466962 |
| PAM16 | chr16 | 4390251 | 4401373 |
| CORO7 | chr16 | 4404542 | 4466962 |
| DNAJA3 | chr16 | 4475805 | 4506775 |
| HMOX2 | chr16 | 4545858 | 4560348 |
| UBALD1 | chr16 | 4658883 | 4664927 |
| MGRN1 | chr16 | 4674824 | 4740975 |
| GLYR1 | chr16 | 4853203 | 4897303 |
| UBN1 | chr16 | 4897911 | 4932363 |
| METTL22 | chr16 | 8715526 | 8740079 |
| TMEM186 | chr16 | 8889036 | 8891505 |
| CARHSP1 | chr16 | 8946798 | 8962258 |
| DEXI | chr16 | 11022747 | 11036257 |
| CLEC16A | chr16 | 11038344 | 11276046 |
| TXNDC11 | chr16 | 11772942 | 11836648 |
| RSL1D1 | chr16 | 11928054 | 11945442 |
| CPPED1 | chr16 | 12753655 | 12897744 |
| PARN | chr16 | 14529556 | 14724128 |
| NOMO1 | chr16 | 14927642 | 14990014 |
| MIR3180-3 | chr16 | 15005076 | 15005170 |
| MIR3180-1 | chr16 | 15005076 | 15005170 |
| PDXDC1 | chr16 | 15068832 | 15131552 |
| NTAN1 | chr16 | 15131709 | 15149936 |
| RRN3 | chr16 | 15153878 | 15188158 |
| MIR3180-4 | chr16 | 15248706 | 15248859 |
| KIAA0430 | chr16 | 15688225 | 15737023 |
| NDE1 | chr16 | 15737123 | 15820208 |
| MIR484 | chr16 | 15737150 | 15737229 |
| FOPNL | chr16 | 15959576 | 15982447 |
| NOMO3 | chr16 | 16326388 | 16388668 |
| NOMO2 | chr16 | 18511181 | 18573434 |
| COQ7 | chr16 | 19079310 | 19091417 |
| GDE1 | chr16 | 19513014 | 19533450 |
| CCP110 | chr16 | 19535178 | 19564728 |
| C16orf62 | chr16 | 19566736 | 19712485 |
| KNOP1 | chr16 | 19717673 | 19729492 |
| IQCK | chr16 | 19727777 | 19868859 |
| THUMPD1 | chr16 | 20744988 | 20753199 |
| LOC81691 | chr16 | 20817766 | 20860990 |
| LYRM1 | chr16 | 20912074 | 20936328 |
| METTL9 | chr16 | 21610855 | 21668792 |
| RRN3P1 | chr16 | 21807950 | 21830495 |
| UQCRC2 | chr16 | 21964608 | 21994668 |
| C16orf52 | chr16 | 22019455 | 22095972 |
| EEF2K | chr16 | 22217591 | 22300066 |
| POLR3E | chr16 | 22308695 | 22346424 |
| RRN3P3 | chr16 | 22430866 | 22449036 |
| USP31 | chr16 | 23072727 | 23160591 |
| GGA2 | chr16 | 23474862 | 23521815 |
| EARS2 | chr16 | 23533333 | 23568696 |
| UBFD1 | chr16 | 23568861 | 23585710 |
| NDUFAB1 | chr16 | 23592334 | 23607639 |
| PALB2 | chr16 | 23614482 | 23652678 |
| DCTN5 | chr16 | 23652686 | 23685068 |
| LCMT1 | chr16 | 25123046 | 25189551 |
| KDM8 | chr16 | 27215295 | 27233089 |
| GTF3C1 | chr16 | 27471933 | 27561251 |
| KIAA0556 | chr16 | 27561467 | 27791692 |
| EIF3CL | chr16 | 28390899 | 28405372 |
| EIF3C | chr16 | 28390899 | 28415165 |
| TUFM | chr16 | 28853731 | 28857729 |
| SH2B1 | chr16 | 28875313 | 28885534 |
| NFATC2IP | chr16 | 28962317 | 28977767 |
| SPNS1 | chr16 | 28986095 | 28996838 |
| RRN3P2 | chr16 | 29086162 | 29128038 |
| BOLA2 | chr16 | 29464913 | 29466285 |
| SLX1A | chr16 | 29465821 | 29469545 |
| MAZ | chr16 | 29817857 | 29822504 |
| PAGR1 | chr16 | 29827527 | 29833816 |
| MVP | chr16 | 29831714 | 29859360 |
| CDIPT | chr16 | 29869677 | 29874578 |
| INO80E | chr16 | 30007530 | 30017112 |
| SLX1B-SULT1A4 | chr16 | 30205753 | 30215650 |
| SLX1A-SULT1A3 | chr16 | 30205753 | 30215650 |
| LOC595101 | chr16 | 30278913 | 30346695 |
| CD2BP2 | chr16 | 30362086 | 30366255 |
| ZNF48 | chr16 | 30406432 | 30411429 |
| SEPHS2 | chr16 | 30454951 | 30457224 |
| ZNF764 | chr16 | 30565084 | 30569642 |
| ZNF785 | chr16 | 30591993 | 30597092 |
| PRR14 | chr16 | 30662703 | 30667734 |
| FBRS | chr16 | 30671242 | 30682131 |
| LOC730183 | chr16 | 30709024 | 30709810 |
| SRCAP | chr16 | 30715384 | 30756517 |
| C16orf93 | chr16 | 30768743 | 30773519 |
| RNF40 | chr16 | 30772932 | 30787628 |
| FBXL19-AS1 | chr16 | 30930639 | 30934590 |
| SETD1A | chr16 | 30968614 | 30995981 |
| ZNF668 | chr16 | 31072163 | 31084823 |
| ZNF646 | chr16 | 31087222 | 31094826 |
| KAT8 | chr16 | 31128984 | 31142714 |
| ARMC5 | chr16 | 31470316 | 31478488 |
| TGFB1I1 | chr16 | 31483475 | 31489281 |
| C16orf58 | chr16 | 31500795 | 31519740 |
| ZNF720 | chr16 | 31724549 | 31772886 |
| SHCBP1 | chr16 | 46614467 | 46655311 |
| VPS35 | chr16 | 46693588 | 46723025 |
| ORC6 | chr16 | 46723557 | 46728576 |
| C16orf87 | chr16 | 46835958 | 46865074 |
| DNAJA2 | chr16 | 46989273 | 47007625 |
| ITFG1 | chr16 | 47189297 | 47495015 |
| PHKB | chr16 | 47495209 | 47735434 |
| LONP2 | chr16 | 48278210 | 48387407 |
| SIAH1 | chr16 | 48394445 | 48399784 |
| N4BP1 | chr16 | 48572636 | 48644120 |
| CNEP1R1 | chr16 | 50059188 | 50070999 |
| HEATR3 | chr16 | 50099880 | 50139375 |
| PAPD5 | chr16 | 50186828 | 50269219 |
| BRD7 | chr16 | 50352928 | 50402845 |
| CHD9 | chr16 | 53189837 | 53361414 |
| RBL2 | chr16 | 53468350 | 53525560 |
| AKTIP | chr16 | 53525191 | 53537170 |
| FTO | chr16 | 53737874 | 54148379 |
| MMP2 | chr16 | 55515473 | 55540586 |
| LPCAT2 | chr16 | 55542912 | 55620582 |
| AMFR | chr16 | 56395363 | 56459450 |
| OGFOD1 | chr16 | 56485423 | 56511407 |
| MT1E | chr16 | 56659584 | 56661024 |
| NUP93 | chr16 | 56764016 | 56878861 |
| FAM192A | chr16 | 57186377 | 57219976 |
| CIAPIN1 | chr16 | 57462086 | 57481369 |
| COQ9 | chr16 | 57481336 | 57488216 |
| POLR2C | chr16 | 57496550 | 57505921 |
| GPR56 | chr16 | 57662418 | 57698944 |
| KATNB1 | chr16 | 57769659 | 57791162 |
| KIFC3 | chr16 | 57792128 | 57836439 |
| C16orf80 | chr16 | 58147496 | 58163296 |
| CSNK2A2 | chr16 | 58191811 | 58231782 |
| GINS3 | chr16 | 58426297 | 58440048 |
| LINC00920 | chr16 | 66442426 | 66444803 |
| CKLF | chr16 | 66586465 | 66600190 |
| CKLF-CMTM1 | chr16 | 66586465 | 66613038 |
| CMTM1 | chr16 | 66603838 | 66613038 |
| CMTM3 | chr16 | 66637934 | 66647795 |
| FAM96B | chr16 | 66965957 | 66968326 |
| CES2 | chr16 | 66968373 | 66978994 |
| C16orf70 | chr16 | 67143914 | 67182442 |
| KIAA0895L | chr16 | 67209504 | 67217943 |
| EXOC3L1 | chr16 | 67213332 | 67224107 |
| TMEM208 | chr16 | 67261015 | 67263182 |
| FHOD1 | chr16 | 67263291 | 67281425 |
| PLEKHG4 | chr16 | 67311412 | 67323403 |
| ATP6V0D1 | chr16 | 67471916 | 67515089 |
| FAM65A | chr16 | 67563539 | 67580691 |
| GFOD2 | chr16 | 67714618 | 67753273 |
| RANBP10 | chr16 | 67757004 | 67840555 |
| CENPT | chr16 | 67862059 | 67881361 |
| THAP11 | chr16 | 67876212 | 67878098 |
| NUTF2 | chr16 | 67880818 | 67905219 |
| EDC4 | chr16 | 67906925 | 67918417 |
| PSKH1 | chr16 | 67927174 | 67963581 |
| SLC12A4 | chr16 | 67977376 | 67997968 |
| DDX28 | chr16 | 68055176 | 68057770 |
| DUS2L | chr16 | 68056846 | 68113226 |
| NFATC3 | chr16 | 68122603 | 68263162 |
| SLC7A6 | chr16 | 68298418 | 68335726 |
| SLC7A6OS | chr16 | 68334517 | 68344868 |
| PRMT7 | chr16 | 68344876 | 68391169 |
| CHTF8 | chr16 | 69151911 | 69166493 |
| CIRH1A | chr16 | 69166498 | 69201745 |
| VPS4A | chr16 | 69345286 | 69358946 |
| COG8 | chr16 | 69362523 | 69373526 |
| PDF | chr16 | 69362523 | 69364498 |
| NIP7 | chr16 | 69373414 | 69377013 |
| TERF2 | chr16 | 69389463 | 69419891 |
| CYB5B | chr16 | 69458497 | 69500167 |
| MIR1538 | chr16 | 69599710 | 69599771 |
| NFAT5 | chr16 | 69599868 | 69738569 |
| NQO1 | chr16 | 69743303 | 69760533 |
| NOB1 | chr16 | 69775756 | 69788871 |
| PDXDC2P | chr16 | 70010201 | 70099851 |
| PDPR | chr16 | 70147528 | 70195184 |
| EXOSC6 | chr16 | 70284133 | 70285833 |
| AARS | chr16 | 70286296 | 70323412 |
| DDX19B | chr16 | 70333061 | 70367735 |
| LOC100506083 | chr16 | 70349542 | 70380650 |
| DDX19A | chr16 | 70380823 | 70407281 |
| ST3GAL2 | chr16 | 70413337 | 70434541 |
| COG4 | chr16 | 70514471 | 70557457 |
| SNORD111B | chr16 | 70563401 | 70563502 |
| SNORD111 | chr16 | 70571907 | 70572001 |
| VAC14 | chr16 | 70721341 | 70835061 |
| ATXN1L | chr16 | 71879893 | 71891236 |
| TXNL4B | chr16 | 72118755 | 72127539 |
| DHX38 | chr16 | 72127614 | 72146811 |
| ZFHX3 | chr16 | 72816785 | 73082274 |
| LOC283922 | chr16 | 74366303 | 74402153 |
| GLG1 | chr16 | 74481325 | 74641042 |
| RFWD3 | chr16 | 74655296 | 74700779 |
| MLKL | chr16 | 74705752 | 74734789 |
| ZNRF1 | chr16 | 75032914 | 75144892 |
| BCAR1 | chr16 | 75262927 | 75272980 |
| CFDP1 | chr16 | 75327607 | 75467387 |
| TMEM170A | chr16 | 75477141 | 75498314 |
| ADAT1 | chr16 | 75632246 | 75657221 |
| KARS | chr16 | 75661621 | 75681585 |
| MON1B | chr16 | 77224835 | 77233543 |
| CDYL2 | chr16 | 80637675 | 80838175 |
| CENPN | chr16 | 81040102 | 81065090 |
| GAN | chr16 | 81348570 | 81413803 |
| MIR4720 | chr16 | 81418622 | 81418698 |
| CMIP | chr16 | 81528953 | 81745367 |
| MPHOSPH6 | chr16 | 82181766 | 82203829 |
| CDH13 | chr16 | 82660398 | 83830215 |
| HSBP1 | chr16 | 83841507 | 83846607 |
| OSGIN1 | chr16 | 83982671 | 83999937 |
| MBTPS1 | chr16 | 84087368 | 84150517 |
| TAF1C | chr16 | 84211452 | 84220676 |
| COTL1 | chr16 | 84599203 | 84651669 |
| ZDHHC7 | chr16 | 85008066 | 85045141 |
| C16orf74 | chr16 | 85741123 | 85784689 |
| COX4I1 | chr16 | 85833172 | 85840607 |
| MTHFSD | chr16 | 86563781 | 86588841 |
| FOXL1 | chr16 | 86612114 | 86615304 |
| C16orf95 | chr16 | 87336403 | 87351026 |
| FBXO31 | chr16 | 87362941 | 87394561 |
| ZCCHC14 | chr16 | 87439851 | 87525460 |
| KLHDC4 | chr16 | 87742870 | 87795646 |
| BANP | chr16 | 88003623 | 88110924 |
| ZC3H18 | chr16 | 88636788 | 88698372 |
| MVD | chr16 | 88718347 | 88723674 |
| SNAI3-AS1 | chr16 | 88729780 | 88741914 |
| RNF166 | chr16 | 88762902 | 88770025 |
| CTU2 | chr16 | 88772890 | 88781786 |
| CDT1 | chr16 | 88870185 | 88875666 |
| APRT | chr16 | 88875876 | 88878342 |
| ACSF3 | chr16 | 89160216 | 89222171 |
| SPG7 | chr16 | 89574804 | 89624174 |
| SNORD68 | chr16 | 89627837 | 89627909 |
| CHMP1A | chr16 | 89710838 | 89724193 |
| C16orf55 | chr16 | 89724211 | 89727916 |
| SPATA33 | chr16 | 89724454 | 89727916 |
| VPS9D1 | chr16 | 89773540 | 89786115 |
| ZNF276 | chr16 | 89787951 | 89807332 |
| FANCA | chr16 | 89803958 | 89883065 |
| TCF25 | chr16 | 89939993 | 89977792 |
| TUBB3 | chr16 | 89996842 | 90002505 |
| CENPBD1 | chr16 | 90036182 | 90039240 |
| AFG3L1P | chr16 | 90038987 | 90063028 |
| YWHAE | chr17 | 1247833 | 1303556 |
| CRK | chr17 | 1324646 | 1359561 |
| MIR22HG | chr17 | 1614797 | 1619566 |
| PAFAH1B1 | chr17 | 2496922 | 2588909 |
| CLUH | chr17 | 2592679 | 2614927 |
| PFN1 | chr17 | 4848944 | 4852381 |
| DHX33 | chr17 | 5344231 | 5372380 |
| BCL6B | chr17 | 6926368 | 6932961 |
| GABARAP | chr17 | 7143737 | 7145753 |
| CTDNEP1 | chr17 | 7146905 | 7155259 |
| EIF5A | chr17 | 7210855 | 7215782 |
| POLR2A | chr17 | 7387697 | 7417935 |
| SENP3-EIF4A1 | chr17 | 7465308 | 7482324 |
| TP53 | chr17 | 7571719 | 7590868 |
| RPL26 | chr17 | 8280833 | 8286565 |
| FAM211A-AS1 | chr17 | 16342300 | 16345340 |
| C17orf76-AS1 | chr17 | 16342300 | 16345340 |
| SNORD49B | chr17 | 16342822 | 16342870 |
| MPRIP | chr17 | 16946073 | 17095962 |
| RAI1 | chr17 | 17584786 | 17714765 |
| SREBF1 | chr17 | 17714662 | 17740325 |
| ALKBH5 | chr17 | 18086866 | 18113267 |
| GRAP | chr17 | 18923989 | 18950336 |
| GRAPL | chr17 | 19030781 | 19062148 |
| USP22 | chr17 | 20902905 | 20947073 |
| TMEM11 | chr17 | 21101262 | 21117908 |
| WSB1 | chr17 | 25621105 | 25640645 |
| RPL23A | chr17 | 27047308 | 27051374 |
| TRAF4 | chr17 | 27071022 | 27077976 |
| PHF12 | chr17 | 27232270 | 27278508 |
| NUFIP2 | chr17 | 27582853 | 27621166 |
| MIR4523 | chr17 | 27717679 | 27717748 |
| TAOK1 | chr17 | 27717942 | 27878921 |
| GIT1 | chr17 | 27900486 | 27916610 |
| MIR193A | chr17 | 29887014 | 29887102 |
| SUZ12 | chr17 | 30264043 | 30328057 |
| MIR632 | chr17 | 30677127 | 30677221 |
| ZNF207 | chr17 | 30677156 | 30707975 |
| PSMD11 | chr17 | 30771480 | 30810337 |
| AP2B1 | chr17 | 33914281 | 34053436 |
| TAF15 | chr17 | 34136458 | 34174246 |
| GGNBP2 | chr17 | 34900736 | 34946276 |
| PCGF2 | chr17 | 36890149 | 36904561 |
| RPL23 | chr17 | 37006320 | 37010053 |
| SNORA21 | chr17 | 37009115 | 37009248 |
| LASP1 | chr17 | 37026111 | 37078023 |
| CDK12 | chr17 | 37617738 | 37690800 |
| MSL1 | chr17 | 38278789 | 38293045 |
| RARA | chr17 | 38498270 | 38513895 |
| IGFBP4 | chr17 | 38599675 | 38613982 |
| EIF1 | chr17 | 39845126 | 39847898 |
| ACLY | chr17 | 40023178 | 40075272 |
| DNAJC7 | chr17 | 40128438 | 40169715 |
| RAB5C | chr17 | 40276993 | 40307062 |
| PTRF | chr17 | 40554466 | 40575338 |
| RPL27 | chr17 | 41150445 | 41154971 |
| LSM12 | chr17 | 42112002 | 42144987 |
| MAP3K14 | chr17 | 43340487 | 43394414 |
| KANSL1 | chr17 | 44107281 | 44270166 |
| GOSR2 | chr17 | 45000485 | 45018733 |
| CDC27 | chr17 | 45195310 | 45266665 |
| KPNB1 | chr17 | 45727203 | 45761004 |
| NFE2L1 | chr17 | 46125685 | 46138907 |
| HOXB2 | chr17 | 46620018 | 46622393 |
| HOXB3 | chr17 | 46626231 | 46651810 |
| HOXB4 | chr17 | 46652868 | 46655743 |
| MIR10A | chr17 | 46657199 | 46657309 |
| HOXB6 | chr17 | 46673098 | 46682354 |
| HOXB-AS3 | chr17 | 46673319 | 46679703 |
| UBE2Z | chr17 | 46985730 | 47006422 |
| IGF2BP1 | chr17 | 47074773 | 47133507 |
| KAT7 | chr17 | 47866808 | 47906458 |
| PPP1R9B | chr17 | 48211100 | 48227877 |
| LRRC59 | chr17 | 48458593 | 48474914 |
| LUC7L3 | chr17 | 48796925 | 48830072 |
| TRIM25 | chr17 | 54965269 | 54991409 |
| VEZF1 | chr17 | 56048909 | 56065615 |
| SRSF1 | chr17 | 56078279 | 56084707 |
| DYNLL2 | chr17 | 56160779 | 56167618 |
| SKA2 | chr17 | 57187307 | 57232800 |
| PRR11 | chr17 | 57232859 | 57284070 |
| YPEL2 | chr17 | 57409052 | 57479095 |
| CLTC | chr17 | 57697049 | 57774317 |
| PTRH2 | chr17 | 57774666 | 57784856 |
| VMP1 | chr17 | 57784862 | 57917952 |
| APPBP2 | chr17 | 58520519 | 58603580 |
| PPM1D | chr17 | 58677543 | 58743640 |
| MED13 | chr17 | 60019965 | 60142643 |
| TLK2 | chr17 | 60556385 | 60692841 |
| DCAF7 | chr17 | 61627795 | 61671642 |
| MAP3K3 | chr17 | 61699800 | 61773670 |
| DDX42 | chr17 | 61851566 | 61896677 |
| ICAM2 | chr17 | 62079954 | 62084283 |
| DDX5 | chr17 | 62494373 | 62502484 |
| SMURF2 | chr17 | 62540734 | 62658386 |
| GNA13 | chr17 | 63005406 | 63052920 |
| PSMD12 | chr17 | 65336618 | 65362721 |
| KPNA2 | chr17 | 66032334 | 66042970 |
| PRKAR1A | chr17 | 66510961 | 66529570 |
| RPL38 | chr17 | 72199794 | 72206019 |
| HN1 | chr17 | 73131343 | 73150775 |
| SUMO2 | chr17 | 73163824 | 73179098 |
| GRB2 | chr17 | 73314156 | 73401790 |
| SAP30BP | chr17 | 73663398 | 73704139 |
| UBALD2 | chr17 | 74261285 | 74267379 |
| SNHG16 | chr17 | 74553845 | 74561430 |
| SRSF2 | chr17 | 74730196 | 74733493 |
| MIR636 | chr17 | 74732531 | 74732630 |
| LINC00338 | chr17 | 75084724 | 75091068 |
| SEC14L1 | chr17 | 75085234 | 75213181 |
| SCARNA16 | chr17 | 75085388 | 75085575 |
| PGS1 | chr17 | 76396815 | 76417992 |
| CANT1 | chr17 | 76987797 | 77005899 |
| ACTG1 | chr17 | 79476996 | 79479892 |
| NPLOC4 | chr17 | 79523912 | 79604138 |
| HGS | chr17 | 79650961 | 79669151 |
| P4HB | chr17 | 79801033 | 79818544 |
| ALYREF | chr17 | 79845710 | 79849462 |
| MAFG | chr17 | 79876144 | 79885587 |
| FASN | chr17 | 80036213 | 80056106 |
| SLC16A3 | chr17 | 80191931 | 80197375 |
| CSNK1D | chr17 | 80200536 | 80212834 |
| FOXK2 | chr17 | 80477593 | 80562483 |
| VPS53 | chr17 | 411907 | 618096 |
| GEMIN4 | chr17 | 647660 | 655501 |
| DBIL5P | chr17 | 655572 | 658576 |
| GLOD4 | chr17 | 662548 | 680932 |
| RNMTL1 | chr17 | 685512 | 695741 |
| TIMM22 | chr17 | 900356 | 905390 |
| ABR | chr17 | 906757 | 1012340 |
| MYO1C | chr17 | 1367479 | 1396001 |
| INPP5K | chr17 | 1397870 | 1420182 |
| PITPNA | chr17 | 1421282 | 1466110 |
| SLC43A2 | chr17 | 1477695 | 1531635 |
| SCARF1 | chr17 | 1537151 | 1549083 |
| PRPF8 | chr17 | 1553922 | 1588176 |
| MIR22 | chr17 | 1617196 | 1617281 |
| WDR81 | chr17 | 1619816 | 1641893 |
| SMYD4 | chr17 | 1682828 | 1733175 |
| RPA1 | chr17 | 1733272 | 1802848 |
| OVCA2 | chr17 | 1945276 | 1946725 |
| SMG6 | chr17 | 1963132 | 2207069 |
| SRR | chr17 | 2207247 | 2228553 |
| TSR1 | chr17 | 2225981 | 2240678 |
| SGSM2 | chr17 | 2240805 | 2284348 |
| MNT | chr17 | 2287353 | 2304258 |
| METTL16 | chr17 | 2319347 | 2415200 |
| TAX1BP3 | chr17 | 3566186 | 3571973 |
| EMC6 | chr17 | 3572089 | 3572962 |
| GSG2 | chr17 | 3627196 | 3629992 |
| C17orf85 | chr17 | 3710044 | 3732662 |
| CAMKK1 | chr17 | 3763616 | 3794037 |
| ZZEF1 | chr17 | 3907738 | 4046253 |
| CYB5D2 | chr17 | 4046461 | 4060995 |
| ANKFY1 | chr17 | 4066664 | 4167142 |
| UBE2G1 | chr17 | 4172511 | 4269969 |
| MYBBP1A | chr17 | 4442190 | 4458681 |
| PELP1 | chr17 | 4574678 | 4607632 |
| MINK1 | chr17 | 4736634 | 4801356 |
| SLC25A11 | chr17 | 4840425 | 4843325 |
| RNF167 | chr17 | 4843629 | 4848517 |
| ENO3 | chr17 | 4854383 | 4860426 |
| SPAG7 | chr17 | 4862520 | 4871132 |
| CAMTA2 | chr17 | 4871286 | 4890665 |
| KIF1C | chr17 | 4901242 | 4931694 |
| RABEP1 | chr17 | 5185557 | 5289132 |
| NUP88 | chr17 | 5289345 | 5323059 |
| RPAIN | chr17 | 5322960 | 5332985 |
| C1QBP | chr17 | 5336098 | 5342471 |
| DERL2 | chr17 | 5377612 | 5389494 |
| MIS12 | chr17 | 5389693 | 5394130 |
| NLRP1 | chr17 | 5417437 | 5487832 |
| KIAA0753 | chr17 | 6481644 | 6544247 |
| TXNDC17 | chr17 | 6544221 | 6547861 |
| MED31 | chr17 | 6546632 | 6554954 |
| C17orf100 | chr17 | 6555058 | 6556617 |
| LOC100506713 | chr17 | 6888441 | 6915653 |
| RNASEK-C17orf49 | chr17 | 6915735 | 6920843 |
| RNASEK | chr17 | 6915735 | 6917852 |
| MIR497HG | chr17 | 6919136 | 6922973 |
| MIR497 | chr17 | 6920933 | 6921341 |
| MIR195 | chr17 | 6920933 | 6921020 |
| SLC16A13 | chr17 | 6939393 | 6943440 |
| DVL2 | chr17 | 7128660 | 7137863 |
| PHF23 | chr17 | 7138346 | 7142825 |
| ELP5 | chr17 | 7155550 | 7163259 |
| GPS2 | chr17 | 7215977 | 7222527 |
| ZBTB4 | chr17 | 7362684 | 7387568 |
| SLC35G6 | chr17 | 7384720 | 7386383 |
| SENP3-EIF4A1 | chr17 | 7465308 | 7482324 |
| SENP3 | chr17 | 7465308 | 7475287 |
| SNORA48 | chr17 | 7478030 | 7478165 |
| SNORD10 | chr17 | 7480128 | 7480276 |
| SNORA67 | chr17 | 7481272 | 7481409 |
| CD68 | chr17 | 7482804 | 7485429 |
| MPDU1 | chr17 | 7486964 | 7491527 |
| FXR2 | chr17 | 7494547 | 7518215 |
| WRAP53 | chr17 | 7591666 | 7606820 |
| KDM6B | chr17 | 7743234 | 7758118 |
| LSMD1 | chr17 | 7760002 | 7761172 |
| CYB5D1 | chr17 | 7761063 | 7765600 |
| CHD3 | chr17 | 7788122 | 7816075 |
| TRAPPC1 | chr17 | 7833662 | 7835267 |
| CNTROB | chr17 | 7835441 | 7853237 |
| PER1 | chr17 | 8043787 | 8055753 |
| VAMP2 | chr17 | 8062464 | 8066293 |
| AURKB | chr17 | 8108048 | 8113944 |
| CTC1 | chr17 | 8128138 | 8151413 |
| PFAS | chr17 | 8152595 | 8173809 |
| ARHGEF15 | chr17 | 8213555 | 8225834 |
| LOC100128288 | chr17 | 8261730 | 8263859 |
| NDEL1 | chr17 | 8339169 | 8371495 |
| MYH10 | chr17 | 8377522 | 8534079 |
| SCO1 | chr17 | 10583648 | 10600885 |
| ADPRM | chr17 | 10600926 | 10614875 |
| COX10-AS1 | chr17 | 13932608 | 13972775 |
| COX10 | chr17 | 13972718 | 14111996 |
| PMP22 | chr17 | 15133095 | 15165889 |
| TVP23C-CDRT4 | chr17 | 15339331 | 15466945 |
| TVP23C | chr17 | 15440293 | 15466945 |
| ADORA2B | chr17 | 15848230 | 15879210 |
| ZSWIM7 | chr17 | 15879874 | 15903006 |
| TTC19 | chr17 | 15902693 | 15932723 |
| NCOR1 | chr17 | 15933407 | 16118874 |
| PIGL | chr17 | 16120508 | 16220938 |
| UBB | chr17 | 16284366 | 16286054 |
| SNORD49A | chr17 | 16343349 | 16343420 |
| SNORD65 | chr17 | 16344539 | 16344612 |
| FLCN | chr17 | 17115526 | 17140502 |
| COPS3 | chr17 | 17149937 | 17184617 |
| MED9 | chr17 | 17380299 | 17396534 |
| DRG2 | chr17 | 17991180 | 18011299 |
| FLII | chr17 | 18148130 | 18162230 |
| TOP3A | chr17 | 18177234 | 18218321 |
| SMCR8 | chr17 | 18218593 | 18231370 |
| TVP23B | chr17 | 18684581 | 18710026 |
| FAM83G | chr17 | 18874380 | 18908060 |
| EPN2 | chr17 | 19140689 | 19240028 |
| B9D1 | chr17 | 19240866 | 19281495 |
| MAPK7 | chr17 | 19281033 | 19286857 |
| AKAP10 | chr17 | 19807764 | 19881169 |
| MAP2K3 | chr17 | 21187967 | 21218551 |
| MIR4522 | chr17 | 25620935 | 25621022 |
| KSR1 | chr17 | 25799035 | 25950718 |
| NLK | chr17 | 26369687 | 26523404 |
| TMEM97 | chr17 | 26646120 | 26655711 |
| IFT20 | chr17 | 26655350 | 26662515 |
| TNFAIP1 | chr17 | 26662547 | 26674035 |
| POLDIP2 | chr17 | 26673658 | 26684603 |
| TMEM199 | chr17 | 26684686 | 26689089 |
| SARM1 | chr17 | 26684686 | 26708840 |
| SPAG5 | chr17 | 26904582 | 26926056 |
| SPAG5-AS1 | chr17 | 26926150 | 26944395 |
| KIAA0100 | chr17 | 26941457 | 26972177 |
| SDF2 | chr17 | 26975373 | 26989190 |
| SUPT6H | chr17 | 26989301 | 27029249 |
| RAB34 | chr17 | 27041298 | 27044908 |
| NARR | chr17 | 27043010 | 27044908 |
| SNORD42B | chr17 | 27047567 | 27047634 |
| SNORD4A | chr17 | 27049599 | 27049671 |
| SNORD42A | chr17 | 27050446 | 27050509 |
| SNORD4B | chr17 | 27050698 | 27050772 |
| TLCD1 | chr17 | 27051365 | 27053230 |
| FAM222B | chr17 | 27082995 | 27139453 |
| FLOT2 | chr17 | 27206356 | 27224715 |
| SSH2 | chr17 | 27952964 | 28257018 |
| EFCAB5 | chr17 | 28295808 | 28435470 |
| MIR423 | chr17 | 28444096 | 28444190 |
| MIR3184 | chr17 | 28444103 | 28444178 |
| NSRP1 | chr17 | 28483266 | 28513493 |
| BLMH | chr17 | 28575212 | 28619184 |
| GOSR1 | chr17 | 28804425 | 28853832 |
| CRLF3 | chr17 | 29109701 | 29151778 |
| ATAD5 | chr17 | 29159022 | 29222295 |
| TEFM | chr17 | 29226000 | 29233286 |
| NF1 | chr17 | 29421944 | 29704695 |
| MIR4725 | chr17 | 29902287 | 29902377 |
| MIR365B | chr17 | 29902429 | 29902540 |
| UTP6 | chr17 | 30190189 | 30215240 |
| RHOT1 | chr17 | 30469472 | 30536757 |
| MYO1D | chr17 | 30819627 | 31203902 |
| CCL2 | chr17 | 32582295 | 32584220 |
| CCT6B | chr17 | 33254877 | 33288528 |
| ZNF830 | chr17 | 33288548 | 33290205 |
| LIG3 | chr17 | 33307516 | 33332088 |
| RFFL | chr17 | 33336130 | 33396546 |
| NLE1 | chr17 | 33458367 | 33469322 |
| SLFN5 | chr17 | 33570085 | 33594761 |
| SLFN11 | chr17 | 33677328 | 33700720 |
| SLFN12 | chr17 | 33738080 | 33760173 |
| MYO19 | chr17 | 34851598 | 34891305 |
| PIGW | chr17 | 34891402 | 34895150 |
| MRM1 | chr17 | 34958024 | 34965407 |
| ACACA | chr17 | 35441926 | 35766902 |
| TADA2A | chr17 | 35767311 | 35837226 |
| DUSP14 | chr17 | 35869980 | 35873588 |
| SYNRG | chr17 | 35874899 | 35969486 |
| DDX52 | chr17 | 35972362 | 36003493 |
| LOC440434 | chr17 | 36351795 | 36413256 |
| ARHGAP23 | chr17 | 36584719 | 36668628 |
| PSMB3 | chr17 | 36909001 | 36920478 |
| PIP4K2B | chr17 | 36921943 | 36956158 |
| CWC25 | chr17 | 36956686 | 36981603 |
| MIR4727 | chr17 | 36982090 | 36982145 |
| CACNB1 | chr17 | 37329708 | 37350136 |
| RPL19 | chr17 | 37356535 | 37360980 |
| MED1 | chr17 | 37560537 | 37607527 |
| ORMDL3 | chr17 | 38077295 | 38083884 |
| PSMD3 | chr17 | 38137020 | 38154213 |
| CSF3 | chr17 | 38171613 | 38174066 |
| NR1D1 | chr17 | 38249036 | 38256978 |
| CASC3 | chr17 | 38296506 | 38328431 |
| WIPF2 | chr17 | 38375573 | 38438439 |
| CDC6 | chr17 | 38444145 | 38459413 |
| TOP2A | chr17 | 38544772 | 38574202 |
| SMARCE1 | chr17 | 38783975 | 38804103 |
| LEPREL4 | chr17 | 39958204 | 39968855 |
| FKBP10 | chr17 | 39968961 | 39979469 |
| KLHL11 | chr17 | 40009798 | 40021629 |
| CNP | chr17 | 40118758 | 40129754 |
| NKIRAS2 | chr17 | 40172124 | 40177656 |
| STAT5B | chr17 | 40351194 | 40428424 |
| STAT3 | chr17 | 40465342 | 40540513 |
| COASY | chr17 | 40714091 | 40718299 |
| MLX | chr17 | 40719077 | 40725221 |
| PSMC3IP | chr17 | 40724327 | 40729849 |
| FAM134C | chr17 | 40731525 | 40761445 |
| TUBG1 | chr17 | 40761357 | 40767256 |
| BECN1 | chr17 | 40962149 | 40976310 |
| PSME3 | chr17 | 40985158 | 40995777 |
| VAT1 | chr17 | 41166621 | 41174459 |
| BRCA1 | chr17 | 41196311 | 41277500 |
| NBR2 | chr17 | 41277599 | 41297125 |
| NBR1 | chr17 | 41322510 | 41363707 |
| LINC00910 | chr17 | 41453295 | 41466266 |
| DHX8 | chr17 | 41561333 | 41601680 |
| G6PC3 | chr17 | 42148097 | 42153712 |
| ASB16-AS1 | chr17 | 42253341 | 42264085 |
| TMUB2 | chr17 | 42264573 | 42269099 |
| ATXN7L3 | chr17 | 42269172 | 42275529 |
| UBTF | chr17 | 42282400 | 42298994 |
| GPATCH8 | chr17 | 42472644 | 42542461 |
| CCDC43 | chr17 | 42754804 | 42767165 |
| DBF4B | chr17 | 42785975 | 42827917 |
| GJC1 | chr17 | 42875815 | 42885252 |
| EFTUD2 | chr17 | 42927654 | 42976736 |
| CCDC103 | chr17 | 42977079 | 42981047 |
| KIF18B | chr17 | 43002078 | 43025079 |
| DCAKD | chr17 | 43100705 | 43138473 |
| NMT1 | chr17 | 43138679 | 43186382 |
| HEXIM1 | chr17 | 43224683 | 43229468 |
| HEXIM2 | chr17 | 43238263 | 43247406 |
| PLEKHM1 | chr17 | 43513265 | 43568146 |
| KANSL1-AS1 | chr17 | 44270938 | 44274089 |
| NPEPPS | chr17 | 45608443 | 45700642 |
| MRPL10 | chr17 | 45900637 | 45908907 |
| SP2 | chr17 | 45974973 | 46006323 |
| CBX1 | chr17 | 46147413 | 46178560 |
| SNX11 | chr17 | 46184919 | 46200105 |
| HOXB5 | chr17 | 46668618 | 46671103 |
| HOXB-AS3 | chr17 | 46669653 | 46683774 |
| HOXB7 | chr17 | 46684594 | 46688383 |
| ATP5G1 | chr17 | 46970147 | 46973232 |
| SNF8 | chr17 | 47007458 | 47022154 |
| GNGT2 | chr17 | 47283595 | 47286743 |
| ABI3 | chr17 | 47287588 | 47300587 |
| ZNF652 | chr17 | 47366567 | 47439476 |
| PHB | chr17 | 47481419 | 47492242 |
| SPOP | chr17 | 47676245 | 47723992 |
| SLC35B1 | chr17 | 47778689 | 47785282 |
| ITGA3 | chr17 | 48133339 | 48167849 |
| MRPL27 | chr17 | 48445227 | 48450562 |
| EME1 | chr17 | 48450580 | 48458820 |
| ANKRD40 | chr17 | 48770550 | 48785270 |
| SPAG9 | chr17 | 49039534 | 49198226 |
| NME1-NME2 | chr17 | 49230896 | 49249105 |
| NME1 | chr17 | 49230896 | 49239450 |
| NME2 | chr17 | 49243632 | 49249105 |
| MBTD1 | chr17 | 49254785 | 49337427 |
| UTP18 | chr17 | 49337896 | 49375292 |
| COX11 | chr17 | 53029258 | 53046064 |
| STXBP4 | chr17 | 53046125 | 53241449 |
| C17orf67 | chr17 | 54869273 | 54911256 |
| DGKE | chr17 | 54911459 | 54946036 |
| COIL | chr17 | 55015560 | 55038411 |
| AKAP1 | chr17 | 55162552 | 55198710 |
| MRPS23 | chr17 | 55916286 | 55927433 |
| RAD51C | chr17 | 56769962 | 56772241 |
| TRIM37 | chr17 | 57075560 | 57184266 |
| MIR454 | chr17 | 57215118 | 57215233 |
| MIR301A | chr17 | 57228496 | 57228582 |
| SMG8 | chr17 | 57287370 | 57292611 |
| MIR4729 | chr17 | 57443443 | 57443515 |
| MIR21 | chr17 | 57918626 | 57918698 |
| TUBD1 | chr17 | 57936840 | 57970306 |
| RPS6KB1 | chr17 | 57970406 | 58027786 |
| USP32 | chr17 | 58254690 | 58469586 |
| BRIP1 | chr17 | 59756546 | 59940920 |
| METTL2A | chr17 | 60501245 | 60527454 |
| CYB561 | chr17 | 61509664 | 61515310 |
| STRADA | chr17 | 61780191 | 61819330 |
| CCDC47 | chr17 | 61822609 | 61851088 |
| FTSJ3 | chr17 | 61896792 | 61905031 |
| PSMC5 | chr17 | 61904769 | 61909387 |
| SMARCD2 | chr17 | 61909440 | 61920351 |
| ICAM2 | chr17 | 62079954 | 62097994 |
| ERN1 | chr17 | 62120389 | 62207502 |
| SNORD104 | chr17 | 62223437 | 62223517 |
| SNORA76 | chr17 | 62223698 | 62223831 |
| PECAM1 | chr17 | 62396776 | 62407083 |
| POLG2 | chr17 | 62473901 | 62493184 |
| MIR3064 | chr17 | 62496891 | 62496957 |
| MIR5047 | chr17 | 62497331 | 62497431 |
| CEP95 | chr17 | 62503157 | 62534062 |
| LOC146880 | chr17 | 62774256 | 62777622 |
| PLEKHM1P | chr17 | 62780958 | 62833302 |
| PRKCA | chr17 | 64298925 | 64806862 |
| HELZ | chr17 | 65066553 | 65235605 |
| NOL11 | chr17 | 65714060 | 65740266 |
| BPTF | chr17 | 65821779 | 65980494 |
| PRKAR1A | chr17 | 66511534 | 66547457 |
| KCNJ2-AS1 | chr17 | 68163101 | 68165543 |
| KCNJ2 | chr17 | 68164813 | 68176183 |
| COG1 | chr17 | 71189172 | 71204645 |
| FAM104A | chr17 | 71203491 | 71228533 |
| C17orf80 | chr17 | 71228371 | 71245095 |
| CDC42EP4 | chr17 | 71279762 | 71308143 |
| NAT9 | chr17 | 72766685 | 72772470 |
| TMEM104 | chr17 | 72772621 | 72835922 |
| CDR2L | chr17 | 72983726 | 73001892 |
| ICT1 | chr17 | 73008779 | 73017356 |
| SLC16A5 | chr17 | 73085032 | 73102255 |
| NUP85 | chr17 | 73201596 | 73231854 |
| GGA3 | chr17 | 73232693 | 73257707 |
| MRPS7 | chr17 | 73257748 | 73262457 |
| SLC25A19 | chr17 | 73269060 | 73283755 |
| MIR3678 | chr17 | 73402149 | 73402243 |
| CASKIN2 | chr17 | 73496340 | 73511664 |
| RECQL5 | chr17 | 73622924 | 73663269 |
| H3F3B | chr17 | 73772514 | 73775860 |
| MIR4738 | chr17 | 73780601 | 73780688 |
| UNK | chr17 | 73780919 | 73821886 |
| WBP2 | chr17 | 73841779 | 73851501 |
| TRIM47 | chr17 | 73870244 | 73874656 |
| TRIM65 | chr17 | 73885040 | 73893084 |
| ACOX1 | chr17 | 73937588 | 73975515 |
| TEN1-CDK3 | chr17 | 73975297 | 74002080 |
| TEN1 | chr17 | 73975297 | 73996667 |
| SRP68 | chr17 | 74034855 | 74068607 |
| PRPSAP1 | chr17 | 74306867 | 74350230 |
| SPHK1 | chr17 | 74379057 | 74383941 |
| UBE2O | chr17 | 74385612 | 74449288 |
| RHBDF2 | chr17 | 74466974 | 74497509 |
| SNORD1C | chr17 | 74554873 | 74554951 |
| SNORD1B | chr17 | 74557189 | 74557275 |
| SNORD1A | chr17 | 74557714 | 74557788 |
| MXRA7 | chr17 | 74671808 | 74707056 |
| JMJD6 | chr17 | 74708913 | 74722881 |
| METTL23 | chr17 | 74722911 | 74729962 |
| MFSD11 | chr17 | 74732271 | 74775336 |
| BIRC5 | chr17 | 76210276 | 76221716 |
| SOCS3 | chr17 | 76352858 | 76356158 |
| USP36 | chr17 | 76792398 | 76836969 |
| TIMP2 | chr17 | 76849058 | 76921472 |
| TBC1D16 | chr17 | 77906141 | 78009657 |
| EIF4A3 | chr17 | 78109012 | 78120982 |
| RNF213 | chr17 | 78234659 | 78372581 |
| RPTOR | chr17 | 78518624 | 78940173 |
| ENTHD2 | chr17 | 79202076 | 79212203 |
| C17orf89 | chr17 | 79213110 | 79215098 |
| SLC38A10 | chr17 | 79218798 | 79259514 |
| BAHCC1 | chr17 | 79373539 | 79433358 |
| MIR4740 | chr17 | 79374515 | 79374578 |
| C17orf70 | chr17 | 79506910 | 79519429 |
| OXLD1 | chr17 | 79632065 | 79633625 |
| CCDC137 | chr17 | 79633760 | 79640936 |
| ARL16 | chr17 | 79648223 | 79650954 |
| MRPL12 | chr17 | 79670399 | 79674556 |
| ARHGDIA | chr17 | 79825596 | 79828790 |
| ANAPC11 | chr17 | 79849598 | 79858363 |
| SIRT7 | chr17 | 79869814 | 79876058 |
| MAFG | chr17 | 79876144 | 79881444 |
| MAFG-AS1 | chr17 | 79885704 | 79888629 |
| SLC16A3 | chr17 | 80190107 | 80197375 |
| WDR45B | chr17 | 80572437 | 80606411 |
| USP14 | chr18 | 158482 | 213739 |
| MYL12A | chr18 | 3247527 | 3256234 |
| MYL12B | chr18 | 3262610 | 3278282 |
| TGIF1 | chr18 | 3451590 | 3458406 |
| RAB12 | chr18 | 8609442 | 8639380 |
| NDUFV2 | chr18 | 9102627 | 9134343 |
| PPP4R1 | chr18 | 9546788 | 9614605 |
| VAPA | chr18 | 9913954 | 9960018 |
| TUBB6 | chr18 | 12308256 | 12326568 |
| SPIRE1 | chr18 | 12446510 | 12657912 |
| CEP192 | chr18 | 12991360 | 13125051 |
| ROCK1 | chr18 | 18529702 | 18691812 |
| SNRPD1 | chr18 | 19192259 | 19210208 |
| GATA6 | chr18 | 19749403 | 19782491 |
| ZNF521 | chr18 | 22641887 | 22932214 |
| RNF138 | chr18 | 29672568 | 29711524 |
| MAPRE2 | chr18 | 32621323 | 32723432 |
| ZNF24 | chr18 | 32912177 | 32924426 |
| MIR3975 | chr18 | 33171700 | 33171770 |
| ATP5A1 | chr18 | 43664109 | 43678319 |
| SMAD2 | chr18 | 45359465 | 45456970 |
| RPL17-C18orf32 | chr18 | 47007547 | 47018935 |
| RPL17 | chr18 | 47014850 | 47018935 |
| LIPG | chr18 | 47088426 | 47119278 |
| MEX3C | chr18 | 48700919 | 48724051 |
| MBD2 | chr18 | 51677970 | 51751158 |
| SNORA37 | chr18 | 51748653 | 51748782 |
| TCF4 | chr18 | 52889561 | 53071226 |
| TXNL1 | chr18 | 54275509 | 54305736 |
| ATP8B1 | chr18 | 55313658 | 55470327 |
| SOCS6 | chr18 | 67956136 | 67997434 |
| CTDP1 | chr18 | 77439800 | 77514510 |
| TXNL4A | chr18 | 77732866 | 77748532 |
| ROCK1P1 | chr18 | 109064 | 122222 |
| THOC1 | chr18 | 214519 | 268059 |
| C18orf56 | chr18 | 649619 | 658340 |
| TYMS | chr18 | 657603 | 673499 |
| ENOSF1 | chr18 | 670323 | 712662 |
| YES1 | chr18 | 721591 | 812327 |
| METTL4 | chr18 | 2537523 | 2571489 |
| NDC80 | chr18 | 2571509 | 2616634 |
| CBX3P2 | chr18 | 2652168 | 2655394 |
| SMCHD1 | chr18 | 2655885 | 2805015 |
| LPIN2 | chr18 | 2916991 | 3011945 |
| DLGAP1-AS1 | chr18 | 3594111 | 3597377 |
| EPB41L3 | chr18 | 5392387 | 5543986 |
| SOGA2 | chr18 | 8717368 | 8832775 |
| ANKRD12 | chr18 | 9137560 | 9285983 |
| TWSG1 | chr18 | 9334764 | 9402418 |
| RALBP1 | chr18 | 9475006 | 9538106 |
| NAPG | chr18 | 10539120 | 10552766 |
| CHMP1B | chr18 | 11851388 | 11854448 |
| AFG3L2 | chr18 | 12328942 | 12377275 |
| SLMO1 | chr18 | 12420187 | 12432236 |
| SPIRE1 | chr18 | 12446510 | 12636339 |
| CEP76 | chr18 | 12672625 | 12702776 |
| PSMG2 | chr18 | 12702986 | 12725658 |
| PTPN2 | chr18 | 12792300 | 12884334 |
| SEH1L | chr18 | 12947982 | 12987536 |
| FAM210A | chr18 | 13663345 | 13726591 |
| RNMT | chr18 | 13726703 | 13764554 |
| ESCO1 | chr18 | 19109261 | 19180693 |
| MIB1 | chr18 | 19321544 | 19450912 |
| CABLES1 | chr18 | 20715726 | 20840434 |
| RIOK3 | chr18 | 21032786 | 21063099 |
| NPC1 | chr18 | 21111462 | 21166581 |
| SS18 | chr18 | 23596216 | 23670611 |
| CDH2 | chr18 | 25530929 | 25757445 |
| TRAPPC8 | chr18 | 29409135 | 29523091 |
| RNF138 | chr18 | 29671817 | 29711524 |
| ZNF397 | chr18 | 32820993 | 32827153 |
| ZSCAN30 | chr18 | 32831022 | 32870196 |
| ZNF271 | chr18 | 32870235 | 32890730 |
| INO80C | chr18 | 33033289 | 33077955 |
| GALNT1 | chr18 | 33234532 | 33291798 |
| C18orf21 | chr18 | 33552587 | 33559250 |
| RPRD1A | chr18 | 33569791 | 33647373 |
| SLC39A6 | chr18 | 33688493 | 33709357 |
| ELP2 | chr18 | 33709836 | 33754688 |
| TPGS2 | chr18 | 34374068 | 34409179 |
| KIAA1328 | chr18 | 34409079 | 34805288 |
| PIK3C3 | chr18 | 39605294 | 39661446 |
| C18orf25 | chr18 | 43753987 | 43846955 |
| IER3IP1 | chr18 | 44681389 | 44702745 |
| SMAD2 | chr18 | 45359465 | 45457517 |
| CTIF | chr18 | 46065426 | 46389586 |
| RPL17-C18orf32 | chr18 | 47007547 | 47017954 |
| C18orf32 | chr18 | 47007547 | 47013644 |
| MIR1539 | chr18 | 47013742 | 47013792 |
| SNORD58C | chr18 | 47015604 | 47015694 |
| SNORD58A | chr18 | 47017652 | 47017717 |
| SNORD58B | chr18 | 47018033 | 47018099 |
| ACAA2 | chr18 | 47309873 | 47340251 |
| MBD1 | chr18 | 47795215 | 47808144 |
| SKA1 | chr18 | 47901391 | 47920538 |
| ME2 | chr18 | 48405431 | 48476162 |
| SMAD4 | chr18 | 48578695 | 48611411 |
| C18orf54 | chr18 | 51884287 | 51908404 |
| TCF4 | chr18 | 52889561 | 53255860 |
| MIR4529 | chr18 | 53146451 | 53146529 |
| NARS | chr18 | 55267893 | 55289177 |
| NEDD4L | chr18 | 55888753 | 56068772 |
| LMAN1 | chr18 | 56995055 | 57026508 |
| PMAIP1 | chr18 | 57567191 | 57571538 |
| PIGN | chr18 | 59711457 | 59854289 |
| KIAA1468 | chr18 | 59854523 | 59974355 |
| ZCCHC2 | chr18 | 60190657 | 60253962 |
| KDSR | chr18 | 60994970 | 61034506 |
| VPS4B | chr18 | 61056424 | 61089752 |
| SERPINB8 | chr18 | 61637262 | 61656608 |
| TMX3 | chr18 | 66340924 | 66382353 |
| TIMM21 | chr18 | 71815745 | 71826204 |
| CNDP2 | chr18 | 72163499 | 72190689 |
| LINC00909 | chr18 | 72259009 | 72265071 |
| ZADH2 | chr18 | 72909277 | 72921281 |
| TSHZ1 | chr18 | 72922709 | 73001905 |
| LOC100131655 | chr18 | 74506687 | 74534251 |
| ZNF236 | chr18 | 74536115 | 74682682 |
| NFATC1 | chr18 | 77160325 | 77289323 |
| CTDP1 | chr18 | 77441429 | 77514510 |
| ADNP2 | chr18 | 77866914 | 77898228 |
| MIER2 | chr19 | 305574 | 344791 |
| CDC34 | chr19 | 531732 | 542087 |
| BSG | chr19 | 578849 | 583493 |
| RNF126 | chr19 | 647525 | 663233 |
| PTBP1 | chr19 | 797391 | 812327 |
| TMEM259 | chr19 | 1009649 | 1021141 |
| CNN2 | chr19 | 1026297 | 1039064 |
| CIRBP | chr19 | 1269266 | 1274809 |
| DAZAP1 | chr19 | 1407583 | 1435682 |
| MEX3D | chr19 | 1554667 | 1568057 |
| REXO1 | chr19 | 1815244 | 1848452 |
| KLF16 | chr19 | 1852397 | 1863564 |
| CSNK1G2 | chr19 | 1941160 | 1981336 |
| AP3D1 | chr19 | 2100986 | 2151556 |
| SF3A2 | chr19 | 2236815 | 2248678 |
| OAZ1 | chr19 | 2269519 | 2273487 |
| LMNB2 | chr19 | 2428162 | 2456966 |
| GADD45B | chr19 | 2476122 | 2478257 |
| SGTA | chr19 | 2754711 | 2783354 |
| GNA11 | chr19 | 3094407 | 3121454 |
| NFIC | chr19 | 3366564 | 3469215 |
| FZR1 | chr19 | 3506294 | 3536755 |
| PIP5K1C | chr19 | 3630178 | 3700477 |
| EEF2 | chr19 | 3976053 | 3985461 |
| SNORD37 | chr19 | 3982504 | 3982570 |
| ZBTB7A | chr19 | 4045215 | 4066816 |
| SH3GL1 | chr19 | 4360363 | 4400565 |
| SAFB2 | chr19 | 5587009 | 5622938 |
| SAFB | chr19 | 5623045 | 5668489 |
| RPL36 | chr19 | 5690345 | 5691678 |
| LONP1 | chr19 | 5691844 | 5720269 |
| RANBP3 | chr19 | 5916151 | 5934473 |
| MLLT1 | chr19 | 6210391 | 6279959 |
| KHSRP | chr19 | 6413118 | 6424822 |
| ELAVL1 | chr19 | 8023456 | 8070529 |
| HNRNPM | chr19 | 8509802 | 8554002 |
| DNMT1 | chr19 | 10244021 | 10305755 |
| CDC37 | chr19 | 10501808 | 10514271 |
| MIR1181 | chr19 | 10514133 | 10514214 |
| ILF3 | chr19 | 10764936 | 10803095 |
| MIR638 | chr19 | 10829079 | 10829179 |
| DNM2 | chr19 | 10919883 | 10942586 |
| SMARCA4 | chr19 | 11071597 | 11172958 |
| LDLR | chr19 | 11201274 | 11244505 |
| TNPO2 | chr19 | 12810007 | 12832529 |
| C19orf43 | chr19 | 12841453 | 12845529 |
| CALR | chr19 | 13049413 | 13055304 |
| LYL1 | chr19 | 13209841 | 13213974 |
| NACC1 | chr19 | 13229101 | 13251961 |
| IER2 | chr19 | 13261281 | 13265718 |
| DDX39A | chr19 | 14519609 | 14529906 |
| BRD4 | chr19 | 15348300 | 15391262 |
| AKAP8 | chr19 | 15464331 | 15490612 |
| AKAP8L | chr19 | 15490858 | 15529833 |
| TPM4 | chr19 | 16187134 | 16213813 |
| CHERP | chr19 | 16628699 | 16653263 |
| MED26 | chr19 | 16685717 | 16739015 |
| MYO9B | chr19 | 17186590 | 17324104 |
| MAP1S | chr19 | 17831093 | 17845324 |
| RPL18A | chr19 | 17970686 | 17974133 |
| JUND | chr19 | 18390562 | 18392432 |
| SSBP4 | chr19 | 18530145 | 18545372 |
| ELL | chr19 | 18553472 | 18632937 |
| UPF1 | chr19 | 18942743 | 18979039 |
| RPSAP58 | chr19 | 23945815 | 24010919 |
| UQCRFS1 | chr19 | 29698166 | 29704136 |
| CEBPG | chr19 | 33864574 | 33873592 |
| LSM14A | chr19 | 34699100 | 34720420 |
| UBA2 | chr19 | 34919780 | 34960798 |
| ZNF792 | chr19 | 35447257 | 35454953 |
| GRAMD1A | chr19 | 35491245 | 35517373 |
| ZNF146 | chr19 | 36705503 | 36729675 |
| ACTN4 | chr19 | 39138266 | 39221171 |
| HNRNPL | chr19 | 39327027 | 39340617 |
| RPS16 | chr19 | 39923846 | 39926618 |
| SUPT5H | chr19 | 39958093 | 39965865 |
| FBL | chr19 | 40328900 | 40337054 |
| AKT2 | chr19 | 40736223 | 40749209 |
| ITPKC | chr19 | 41223007 | 41246765 |
| SNRPA | chr19 | 41256758 | 41271297 |
| RAB4B-EGLN2 | chr19 | 41284123 | 41314346 |
| EGLN2 | chr19 | 41305449 | 41314346 |
| AXL | chr19 | 41725107 | 41767671 |
| HNRNPUL1 | chr19 | 41770119 | 41813811 |
| TGFB1 | chr19 | 41836811 | 41859831 |
| RPS19 | chr19 | 42363987 | 42375484 |
| ZNF574 | chr19 | 42572628 | 42585720 |
| ERF | chr19 | 42751716 | 42759309 |
| PVR | chr19 | 45147097 | 45169428 |
| PVRL2 | chr19 | 45349392 | 45392485 |
| CLPTM1 | chr19 | 45458598 | 45496604 |
| PPP1R37 | chr19 | 45596430 | 45650543 |
| VASP | chr19 | 46010687 | 46030240 |
| CALM3 | chr19 | 47104511 | 47114039 |
| STRN4 | chr19 | 47222767 | 47249720 |
| SLC1A5 | chr19 | 47278139 | 47291842 |
| ZC3H4 | chr19 | 47567446 | 47617009 |
| SAE1 | chr19 | 47634079 | 47713893 |
| GLTSCR1 | chr19 | 48182218 | 48206534 |
| RPL18 | chr19 | 49118583 | 49122675 |
| PPP1R15A | chr19 | 49375648 | 49379319 |
| SNRNP70 | chr19 | 49588464 | 49611863 |
| RPL13A | chr19 | 49990810 | 49995564 |
| RPL13AP5 | chr19 | 49990864 | 49995096 |
| SCAF1 | chr19 | 50145381 | 50161906 |
| PRMT1 | chr19 | 50180408 | 50191707 |
| IL4I1 | chr19 | 50390638 | 50432988 |
| NUP62 | chr19 | 50410083 | 50432988 |
| C19orf48 | chr19 | 51300960 | 51307974 |
| PPP2R1A | chr19 | 52704345 | 52729678 |
| RPS9 | chr19 | 54704725 | 54711515 |
| LENG8 | chr19 | 54960064 | 54973196 |
| PPP1R12C | chr19 | 55602280 | 55628968 |
| PPP6R1 | chr19 | 55741146 | 55758740 |
| RPL28 | chr19 | 55897299 | 55903451 |
| UBE2S | chr19 | 55912649 | 55919325 |
| U2AF2 | chr19 | 56165415 | 56186082 |
| ZNF787 | chr19 | 56598731 | 56632649 |
| ZNF460 | chr19 | 57791852 | 57805436 |
| TRIM28 | chr19 | 59055835 | 59062082 |
| UBE2M | chr19 | 59067078 | 59070343 |
| TPGS1 | chr19 | 507496 | 519654 |
| MIR4745 | chr19 | 804939 | 805001 |
| POLR2E | chr19 | 1086577 | 1095391 |
| GPX4 | chr19 | 1104648 | 1106787 |
| SBNO2 | chr19 | 1107632 | 1174282 |
| STK11 | chr19 | 1205797 | 1228434 |
| ATP5D | chr19 | 1241748 | 1244824 |
| MIDN | chr19 | 1248551 | 1259142 |
| CIRBP-AS1 | chr19 | 1267469 | 1270259 |
| C19orf24 | chr19 | 1275519 | 1279243 |
| RPS15 | chr19 | 1438362 | 1440492 |
| UQCR11 | chr19 | 1597153 | 1605483 |
| TCF3 | chr19 | 1609288 | 1652328 |
| ABHD17A | chr19 | 1876974 | 1885518 |
| SCAMP4 | chr19 | 1905370 | 1926012 |
| ADAT3 | chr19 | 1905370 | 1913446 |
| CSNK1G2-AS1 | chr19 | 1952525 | 1954548 |
| MKNK2 | chr19 | 2037469 | 2051243 |
| MOB3A | chr19 | 2071034 | 2096269 |
| DOT1L | chr19 | 2164147 | 2232577 |
| PLEKHJ1 | chr19 | 2233154 | 2236951 |
| MIR1227 | chr19 | 2234060 | 2234148 |
| LSM7 | chr19 | 2321519 | 2328614 |
| SPPL2B | chr19 | 2328628 | 2355100 |
| TIMM13 | chr19 | 2425621 | 2427875 |
| THOP1 | chr19 | 2785505 | 2813599 |
| AES | chr19 | 3052907 | 3062964 |
| NCLN | chr19 | 3185874 | 3209573 |
| DOHH | chr19 | 3490818 | 3500938 |
| MFSD12 | chr19 | 3544196 | 3557571 |
| CACTIN | chr19 | 3610626 | 3626813 |
| APBA3 | chr19 | 3750770 | 3761673 |
| MRPL54 | chr19 | 3762664 | 3767563 |
| DAPK3 | chr19 | 3958451 | 3971038 |
| PIAS4 | chr19 | 4007748 | 4038067 |
| MAP2K2 | chr19 | 4090319 | 4103202 |
| CHAF1A | chr19 | 4402659 | 4443394 |
| HDGFRP2 | chr19 | 4472254 | 4502222 |
| SEMA6B | chr19 | 4542599 | 4559771 |
| TNFAIP8L1 | chr19 | 4640028 | 4655580 |
| C19orf10 | chr19 | 4657556 | 4670415 |
| DPP9 | chr19 | 4675243 | 4723855 |
| FEM1A | chr19 | 4791727 | 4795571 |
| TICAM1 | chr19 | 4815935 | 4831754 |
| PLIN3 | chr19 | 4838345 | 4867780 |
| UHRF1 | chr19 | 4910565 | 4962165 |
| KDM4B | chr19 | 4969123 | 5153608 |
| CATSPERD | chr19 | 5720687 | 5778742 |
| NDUFA11 | chr19 | 5891286 | 5904024 |
| VMAC | chr19 | 5904851 | 5910263 |
| LOC100128568 | chr19 | 5978413 | 6020374 |
| RFX2 | chr19 | 5993174 | 6110664 |
| GTF2F1 | chr19 | 6379579 | 6393291 |
| GPR108 | chr19 | 6729924 | 6737633 |
| TRIP10 | chr19 | 6739706 | 6751529 |
| XAB2 | chr19 | 7684410 | 7694439 |
| PET100 | chr19 | 7694670 | 7696510 |
| PCP2 | chr19 | 7696500 | 7697898 |
| MAP2K7 | chr19 | 7968764 | 7979363 |
| TIMM44 | chr19 | 7991602 | 8008708 |
| CD320 | chr19 | 8367010 | 8373240 |
| NDUFA7 | chr19 | 8376183 | 8386280 |
| RPS28 | chr19 | 8386383 | 8387280 |
| RAB11B-AS1 | chr19 | 8439259 | 8455575 |
| RAB11B | chr19 | 8455204 | 8469317 |
| ZNF317 | chr19 | 9266600 | 9274091 |
| ZNF121 | chr19 | 9676291 | 9695209 |
| ZNF561 | chr19 | 9718001 | 9731916 |
| LOC284385 | chr19 | 9732157 | 9745538 |
| ZNF562 | chr19 | 9759337 | 9785776 |
| FBXL12 | chr19 | 9920942 | 9929731 |
| UBL5 | chr19 | 9938567 | 9940797 |
| PIN1 | chr19 | 9945882 | 9960365 |
| PPAN-P2RY11 | chr19 | 10216898 | 10226064 |
| PPAN | chr19 | 10216898 | 10221975 |
| SNORD105 | chr19 | 10218326 | 10218411 |
| EIF3G | chr19 | 10225689 | 10230599 |
| MRPL4 | chr19 | 10362639 | 10370736 |
| RAVER1 | chr19 | 10426888 | 10444314 |
| KEAP1 | chr19 | 10596795 | 10614054 |
| SLC44A2 | chr19 | 10713120 | 10755235 |
| ILF3-AS1 | chr19 | 10762537 | 10764548 |
| MIR4748 | chr19 | 10890929 | 10891011 |
| CARM1 | chr19 | 10982252 | 11033448 |
| C19orf52 | chr19 | 11039423 | 11040916 |
| SMARCA4 | chr19 | 11094799 | 11172958 |
| SPC24 | chr19 | 11257830 | 11266484 |
| KANK2 | chr19 | 11274942 | 11308243 |
| DOCK6 | chr19 | 11309968 | 11373168 |
| PRKCSH | chr19 | 11546565 | 11561782 |
| ZNF627 | chr19 | 11708234 | 11729974 |
| ZNF823 | chr19 | 11832079 | 11849760 |
| ZNF564 | chr19 | 12636183 | 12662356 |
| ZNF490 | chr19 | 12686919 | 12721623 |
| ZNF791 | chr19 | 12721731 | 12740676 |
| WDR83OS | chr19 | 12778880 | 12780465 |
| WDR83 | chr19 | 12780516 | 12786646 |
| ASNA1 | chr19 | 12848305 | 12859137 |
| JUNB | chr19 | 12902309 | 12904125 |
| FARSA | chr19 | 13033283 | 13044558 |
| RAD23A | chr19 | 13056627 | 13064457 |
| GADD45GIP1 | chr19 | 13064969 | 13068068 |
| TRMT1 | chr19 | 13215713 | 13227463 |
| STX10 | chr19 | 13254871 | 13261188 |
| CCDC130 | chr19 | 13858752 | 13874106 |
| C19orf53 | chr19 | 13885256 | 13889586 |
| ZSWIM4 | chr19 | 13906273 | 13943044 |
| LOC284454 | chr19 | 13945329 | 13947473 |
| MIR24-2 | chr19 | 13947100 | 13947173 |
| MIR27A | chr19 | 13947253 | 13947331 |
| DCAF15 | chr19 | 14063318 | 14072256 |
| RFX1 | chr19 | 14072341 | 14117134 |
| PRKACA | chr19 | 14202506 | 14228559 |
| ASF1B | chr19 | 14230320 | 14247440 |
| PKN1 | chr19 | 14544165 | 14582679 |
| GIPC1 | chr19 | 14588570 | 14606961 |
| DNAJB1 | chr19 | 14625581 | 14629201 |
| NDUFB7 | chr19 | 14676889 | 14682889 |
| SYDE1 | chr19 | 15218213 | 15225789 |
| ILVBL | chr19 | 15225784 | 15236577 |
| TPM4 | chr19 | 16178316 | 16213813 |
| RAB8A | chr19 | 16222489 | 16244445 |
| FAM32A | chr19 | 16296234 | 16302857 |
| AP1M1 | chr19 | 16308664 | 16346156 |
| KLF2 | chr19 | 16435650 | 16438339 |
| SLC35E1 | chr19 | 16660647 | 16683193 |
| SMIM7 | chr19 | 16756958 | 16770968 |
| SIN3B | chr19 | 16940208 | 16991164 |
| HAUS8 | chr19 | 17160570 | 17186343 |
| NR2F6 | chr19 | 17342693 | 17356151 |
| BABAM1 | chr19 | 17378231 | 17388332 |
| DDA1 | chr19 | 17420336 | 17434106 |
| PGLS | chr19 | 17622431 | 17632097 |
| COLGALT1 | chr19 | 17666510 | 17693965 |
| SNORA68 | chr19 | 17973396 | 17973529 |
| PIK3R2 | chr19 | 18263987 | 18288927 |
| MIR3188 | chr19 | 18392886 | 18392971 |
| LSM4 | chr19 | 18417039 | 18434001 |
| GDF15 | chr19 | 18496967 | 18499986 |
| MIR3189 | chr19 | 18497371 | 18497444 |
| KXD1 | chr19 | 18672331 | 18680197 |
| UBA52 | chr19 | 18682613 | 18688270 |
| COPE | chr19 | 19010322 | 19030199 |
| DDX49 | chr19 | 19030483 | 19039442 |
| HOMER3 | chr19 | 19040009 | 19050444 |
| SUGP2 | chr19 | 19103175 | 19144832 |
| ARMC6 | chr19 | 19144386 | 19168987 |
| MEF2BNB-MEF2B | chr19 | 19256375 | 19303400 |
| MEF2BNB | chr19 | 19292684 | 19303400 |
| RFXANK | chr19 | 19303007 | 19312678 |
| SUGP1 | chr19 | 19387321 | 19431321 |
| MAU2 | chr19 | 19431629 | 19469563 |
| GATAD2A | chr19 | 19538335 | 19619741 |
| ATP13A1 | chr19 | 19756009 | 19774503 |
| ZNF430 | chr19 | 21203425 | 21242852 |
| ZNF708 | chr19 | 21473962 | 21512212 |
| ZNF43 | chr19 | 21987750 | 22034870 |
| ZNF91 | chr19 | 23540497 | 23578269 |
| ZNF675 | chr19 | 23835707 | 23870017 |
| ZNF254 | chr19 | 24269975 | 24312654 |
| POP4 | chr19 | 30097169 | 30108162 |
| ZNF507 | chr19 | 32836513 | 32878573 |
| DPY19L3 | chr19 | 32896654 | 32976799 |
| PDCD5 | chr19 | 33072093 | 33077449 |
| ANKRD27 | chr19 | 33087906 | 33166102 |
| NUDT19 | chr19 | 33182866 | 33204702 |
| CEP89 | chr19 | 33369903 | 33462869 |
| GPI | chr19 | 34855644 | 34893318 |
| FXYD5 | chr19 | 35645671 | 35660788 |
| KMT2B | chr19 | 36208920 | 36229779 |
| PSENEN | chr19 | 36236493 | 36237903 |
| C19orf55 | chr19 | 36249043 | 36260077 |
| CAPNS1 | chr19 | 36630917 | 36641251 |
| ZNF565 | chr19 | 36673186 | 36705986 |
| ZNF260 | chr19 | 37001588 | 37019248 |
| ZNF790 | chr19 | 37309223 | 37341169 |
| ZNF345 | chr19 | 37341259 | 37370477 |
| SIPA1L3 | chr19 | 38397867 | 38699008 |
| PSMD8 | chr19 | 38865189 | 38874464 |
| EIF3K | chr19 | 39109721 | 39126125 |
| HNRNPL | chr19 | 39327027 | 39332968 |
| SIRT2 | chr19 | 39369194 | 39385050 |
| NFKBIB | chr19 | 39390569 | 39399534 |
| SARS2 | chr19 | 39405903 | 39440495 |
| MRPS12 | chr19 | 39421593 | 39423659 |
| PAK4 | chr19 | 39616419 | 39670046 |
| SAMD4B | chr19 | 39833107 | 39875537 |
| PAF1 | chr19 | 39876269 | 39881835 |
| MED29 | chr19 | 39881962 | 39891203 |
| ZFP36 | chr19 | 39897486 | 39900052 |
| MIR4530 | chr19 | 39900262 | 39900318 |
| PLEKHG2 | chr19 | 39903749 | 39919055 |
| TIMM50 | chr19 | 39971051 | 39981528 |
| EID2 | chr19 | 40029446 | 40030838 |
| PSMC4 | chr19 | 40476911 | 40487671 |
| MIR641 | chr19 | 40788449 | 40788548 |
| C19orf47 | chr19 | 40826966 | 40854316 |
| PLD3 | chr19 | 40854331 | 40884390 |
| SERTAD1 | chr19 | 40928408 | 40931932 |
| SERTAD3 | chr19 | 40946747 | 40948505 |
| NUMBL | chr19 | 41171811 | 41196556 |
| ADCK4 | chr19 | 41197433 | 41222790 |
| C19orf54 | chr19 | 41246760 | 41255828 |
| CCDC97 | chr19 | 41816093 | 41830788 |
| B9D2 | chr19 | 41860321 | 41870078 |
| TMEM91 | chr19 | 41869870 | 41889987 |
| BCKDHA | chr19 | 41882437 | 41930910 |
| EXOSC5 | chr19 | 41892275 | 41903256 |
| LOC100505495 | chr19 | 41960073 | 42006554 |
| ARHGEF1 | chr19 | 42401092 | 42411604 |
| DEDD2 | chr19 | 42702744 | 42721944 |
| ZNF526 | chr19 | 42724491 | 42732353 |
| GSK3A | chr19 | 42734337 | 42746045 |
| CIC | chr19 | 42788816 | 42799949 |
| XRCC1 | chr19 | 44047463 | 44079730 |
| IRGQ | chr19 | 44088518 | 44100287 |
| ZNF576 | chr19 | 44100543 | 44104587 |
| ZNF428 | chr19 | 44111375 | 44124014 |
| SMG9 | chr19 | 44236421 | 44259142 |
| ZNF180 | chr19 | 44979858 | 45004574 |
| MIR4531 | chr19 | 45156955 | 45157002 |
| BCL3 | chr19 | 45251977 | 45263301 |
| TOMM40 | chr19 | 45394476 | 45406937 |
| CLASRP | chr19 | 45542781 | 45574214 |
| GEMIN7 | chr19 | 45582529 | 45594782 |
| BLOC1S3 | chr19 | 45682002 | 45685058 |
| MARK4 | chr19 | 45754515 | 45808541 |
| PPP1R13L | chr19 | 45882891 | 45909607 |
| CD3EAP | chr19 | 45909466 | 45914024 |
| ERCC1 | chr19 | 45910590 | 45927177 |
| OPA3 | chr19 | 46049538 | 46088122 |
| GPR4 | chr19 | 46093022 | 46105466 |
| SNRPD2 | chr19 | 46190711 | 46195443 |
| QPCTL | chr19 | 46195740 | 46207248 |
| FBXO46 | chr19 | 46213886 | 46226777 |
| DMWD | chr19 | 46286263 | 46296060 |
| SYMPK | chr19 | 46322170 | 46359060 |
| PPP5C | chr19 | 46850250 | 46894232 |
| PPP5D1 | chr19 | 46984044 | 47104457 |
| PRKD2 | chr19 | 47177572 | 47220384 |
| FKRP | chr19 | 47249302 | 47261832 |
| SLC1A5 | chr19 | 47278139 | 47288134 |
| AP2S1 | chr19 | 47341422 | 47354203 |
| BBC3 | chr19 | 47724078 | 47734451 |
| MIR3190 | chr19 | 47730198 | 47730278 |
| MIR3191 | chr19 | 47730200 | 47730276 |
| NAPA | chr19 | 47990890 | 48018515 |
| EHD2 | chr19 | 48216600 | 48246391 |
| GLTSCR2 | chr19 | 48248792 | 48260435 |
| SEPW1 | chr19 | 48281841 | 48287943 |
| LIG1 | chr19 | 48618702 | 48673560 |
| CARD8 | chr19 | 48711342 | 48759203 |
| LOC100505812 | chr19 | 48758931 | 48761450 |
| EMP3 | chr19 | 48828628 | 48833810 |
| KDELR1 | chr19 | 48885826 | 48894112 |
| GRWD1 | chr19 | 48949029 | 48960278 |
| CYTH2 | chr19 | 48972464 | 48985571 |
| SPHK2 | chr19 | 49122547 | 49133663 |
| RASIP1 | chr19 | 49223841 | 49243970 |
| BAX | chr19 | 49458116 | 49465055 |
| FTL | chr19 | 49468565 | 49470136 |
| GYS1 | chr19 | 49471381 | 49496610 |
| RUVBL2 | chr19 | 49497155 | 49519182 |
| TEAD2 | chr19 | 49843852 | 49863695 |
| PIH1D1 | chr19 | 49949549 | 49955115 |
| ALDH16A1 | chr19 | 49956472 | 49974305 |
| SNORD32A | chr19 | 49993222 | 49993304 |
| SNORD33 | chr19 | 49993873 | 49993956 |
| SNORD34 | chr19 | 49994163 | 49994229 |
| SNORD35A | chr19 | 49994431 | 49994517 |
| RPS11 | chr19 | 49999621 | 50002969 |
| SNORD35B | chr19 | 50000975 | 50001062 |
| MIR150 | chr19 | 50004041 | 50004125 |
| NOSIP | chr19 | 50058724 | 50083829 |
| PRR12 | chr19 | 50094911 | 50129696 |
| RRAS | chr19 | 50138551 | 50143400 |
| IRF3 | chr19 | 50162825 | 50169114 |
| BCL2L12 | chr19 | 50168398 | 50177173 |
| MIR5088 | chr19 | 50185327 | 50185411 |
| AP2A1 | chr19 | 50270179 | 50310369 |
| FUZ | chr19 | 50310123 | 50316567 |
| MED25 | chr19 | 50321535 | 50340237 |
| PTOV1 | chr19 | 50354415 | 50363999 |
| PNKP | chr19 | 50364459 | 50370258 |
| AKT1S1 | chr19 | 50372289 | 50380644 |
| TBC1D17 | chr19 | 50380681 | 50392007 |
| ATF5 | chr19 | 50431958 | 50437193 |
| VRK3 | chr19 | 50479723 | 50528805 |
| ZNF473 | chr19 | 50529211 | 50552031 |
| POLD1 | chr19 | 50902107 | 50921275 |
| SNORD88A | chr19 | 51302695 | 51302792 |
| SNORD88C | chr19 | 51305581 | 51305678 |
| CTU1 | chr19 | 51600862 | 51611647 |
| ZNF175 | chr19 | 52074530 | 52092991 |
| MIR99B | chr19 | 52195864 | 52195934 |
| MIRLET7E | chr19 | 52196038 | 52196117 |
| MIR125A | chr19 | 52196506 | 52196592 |
| LINC00085 | chr19 | 52196592 | 52208443 |
| ZNF614 | chr19 | 52516576 | 52531680 |
| ZNF432 | chr19 | 52536676 | 52552073 |
| ZNF841 | chr19 | 52567718 | 52599018 |
| ZNF766 | chr19 | 52772823 | 52795976 |
| MYADM | chr19 | 54372780 | 54379689 |
| TFPT | chr19 | 54610319 | 54619055 |
| PRPF31 | chr19 | 54618789 | 54635150 |
| CNOT3 | chr19 | 54645111 | 54659446 |
| LENG1 | chr19 | 54659378 | 54663446 |
| MBOAT7 | chr19 | 54677105 | 54693733 |
| TSEN34 | chr19 | 54694581 | 54698394 |
| TMEM238 | chr19 | 55890611 | 55895627 |
| FIZ1 | chr19 | 56102736 | 56113336 |
| ZNF524 | chr19 | 56111729 | 56114504 |
| ZNF581 | chr19 | 56146824 | 56156989 |
| ZNF580 | chr19 | 56153417 | 56154836 |
| EPN1 | chr19 | 56187990 | 56207133 |
| ZNF264 | chr19 | 57702867 | 57734214 |
| ZNF805 | chr19 | 57752052 | 57774106 |
| ZNF543 | chr19 | 57831864 | 57842144 |
| ZNF551 | chr19 | 58193336 | 58201169 |
| ZNF587B | chr19 | 58341661 | 58357606 |
| ZNF587 | chr19 | 58361180 | 58376491 |
| ZNF417 | chr19 | 58417141 | 58427978 |
| ZNF274 | chr19 | 58694395 | 58724927 |
| ZNF544 | chr19 | 58740069 | 58775008 |
| ZNF8 | chr19 | 58790317 | 58807254 |
| ZSCAN22 | chr19 | 58838384 | 58853712 |
| MIR4754 | chr19 | 58898136 | 58898225 |
| RPS5 | chr19 | 58898635 | 58906171 |
| ZBTB45 | chr19 | 59024896 | 59030921 |
| CHMP2A | chr19 | 59062932 | 59066005 |
| LOC100131691 | chr19 | 59070552 | 59086164 |
| MZF1 | chr19 | 59073283 | 59084942 |
| MGC2752 | chr19 | 59086765 | 59095762 |
| RPS7 | chr2 | 3622852 | 3628509 |
| ASAP2 | chr2 | 9346893 | 9545812 |
| YWHAQ | chr2 | 9724105 | 9771106 |
| PDIA6 | chr2 | 10923516 | 10978103 |
| ROCK2 | chr2 | 11321777 | 11484711 |
| LPIN1 | chr2 | 11886721 | 11967533 |
| LAPTM4A | chr2 | 20232410 | 20251789 |
| RHOB | chr2 | 20646834 | 20649201 |
| CENPO | chr2 | 25016174 | 25045245 |
| RAB10 | chr2 | 26256728 | 26360323 |
| CENPA | chr2 | 27008881 | 27017455 |
| PPM1G | chr2 | 27604065 | 27632550 |
| ZNF512 | chr2 | 27805835 | 27846082 |
| FOSL2 | chr2 | 28615668 | 28639445 |
| PPP1CB | chr2 | 28974625 | 29025806 |
| WDR43 | chr2 | 29117532 | 29171080 |
| MEMO1 | chr2 | 32092893 | 32235698 |
| BIRC6 | chr2 | 32582095 | 32843965 |
| CRIM1 | chr2 | 36583369 | 36778278 |
| FEZ2 | chr2 | 36779403 | 36825332 |
| CDC42EP3 | chr2 | 37869024 | 37899342 |
| HNRPLL | chr2 | 38790327 | 38830178 |
| SRSF7 | chr2 | 38970740 | 38978636 |
| COX7A2L | chr2 | 42577644 | 42588988 |
| ZFP36L2 | chr2 | 43449540 | 43453745 |
| LRPPRC | chr2 | 44113362 | 44223144 |
| EPAS1 | chr2 | 46524540 | 46613842 |
| MCFD2 | chr2 | 47129008 | 47143007 |
| CALM2 | chr2 | 47387220 | 47403740 |
| FBXO11 | chr2 | 48034058 | 48132932 |
| FOXN2 | chr2 | 48541794 | 48606434 |
| PSME4 | chr2 | 54091203 | 54197977 |
| SPTBN1 | chr2 | 54785530 | 54889445 |
| RTN4 | chr2 | 55199326 | 55277734 |
| RPS27A | chr2 | 55459540 | 55462989 |
| EFEMP1 | chr2 | 56093096 | 56151298 |
| XPO1 | chr2 | 61705068 | 61765418 |
| CCT4 | chr2 | 62095261 | 62115806 |
| PELI1 | chr2 | 64319785 | 64339483 |
| RAB1A | chr2 | 65313987 | 65357435 |
| PPP3R1 | chr2 | 68405988 | 68479651 |
| CNRIP1 | chr2 | 68520141 | 68547183 |
| AAK1 | chr2 | 69685126 | 69870977 |
| PCBP1 | chr2 | 70314584 | 70316334 |
| TIA1 | chr2 | 70436575 | 70475779 |
| RAB11FIP5 | chr2 | 73300509 | 73340146 |
| CCT7 | chr2 | 73461363 | 73480150 |
| KCMF1 | chr2 | 85198230 | 85286595 |
| TGOLN2 | chr2 | 85545140 | 85555419 |
| MAT2A | chr2 | 85766100 | 85772403 |
| IMMT | chr2 | 86371054 | 86422893 |
| LINC00152 | chr2 | 87754973 | 87821030 |
| STARD7 | chr2 | 96850602 | 96874573 |
| EIF5B | chr2 | 99953833 | 100016728 |
| REV1 | chr2 | 100016937 | 100106480 |
| RPL31 | chr2 | 101618690 | 101635061 |
| TBC1D8 | chr2 | 101623689 | 101767846 |
| RNF149 | chr2 | 101892062 | 101925178 |
| MAP4K4 | chr2 | 102314164 | 102511152 |
| FHL2 | chr2 | 105977282 | 106015681 |
| LIMS1 | chr2 | 109271267 | 109303702 |
| 10-Sep | chr2 | 110300373 | 110332344 |
| LOC541471 | chr2 | 112124590 | 112252692 |
| SLC20A1 | chr2 | 113403433 | 113421400 |
| ACTR3 | chr2 | 114647510 | 114719129 |
| WDR33 | chr2 | 128461807 | 128568761 |
| CCNT2 | chr2 | 135676391 | 135716915 |
| UBXN4 | chr2 | 136499188 | 136542633 |
| RND3 | chr2 | 151324706 | 151344209 |
| ARL5A | chr2 | 152657479 | 152685009 |
| PRPF40A | chr2 | 153508106 | 153573975 |
| TANC1 | chr2 | 159825145 | 160089170 |
| 7-Mar | chr2 | 160569009 | 160625084 |
| MIR4785 | chr2 | 161264320 | 161264393 |
| PSMD14 | chr2 | 162164785 | 162268228 |
| PPIG | chr2 | 170440849 | 170494254 |
| GORASP2 | chr2 | 171785035 | 171823643 |
| DYNC1I2 | chr2 | 172543918 | 172606668 |
| SP3 | chr2 | 174771186 | 174828947 |
| ATF2 | chr2 | 175936977 | 176032934 |
| MIR933 | chr2 | 176032360 | 176032437 |
| HNRNPA3 | chr2 | 178077421 | 178088685 |
| MIR4444-1 | chr2 | 178077453 | 178077526 |
| NFE2L2 | chr2 | 178095030 | 178129859 |
| MIR3128 | chr2 | 178120672 | 178120738 |
| AGPS | chr2 | 178257470 | 178408564 |
| SSFA2 | chr2 | 182756471 | 182795464 |
| NCKAP1 | chr2 | 183789578 | 183903586 |
| ZC3H15 | chr2 | 187350884 | 187374087 |
| ITGAV | chr2 | 187454789 | 187545629 |
| CALCRL | chr2 | 188206689 | 188313021 |
| TFPI | chr2 | 188328957 | 188419219 |
| WDR75 | chr2 | 190306158 | 190340264 |
| GLS | chr2 | 191783988 | 191830270 |
| SDPR | chr2 | 192699031 | 192712006 |
| SF3B1 | chr2 | 198256697 | 198299771 |
| HSPD1 | chr2 | 198351307 | 198364640 |
| BZW1 | chr2 | 201676268 | 201688569 |
| CLK1 | chr2 | 201717731 | 201729467 |
| CFLAR | chr2 | 201994451 | 202037411 |
| NOP58 | chr2 | 203130514 | 203168384 |
| BMPR2 | chr2 | 203241049 | 203432474 |
| RAPH1 | chr2 | 204298538 | 204400058 |
| NDUFS1 | chr2 | 206987802 | 207024243 |
| KLF7 | chr2 | 207938861 | 208030740 |
| RPE | chr2 | 210867288 | 210886984 |
| FN1 | chr2 | 216225178 | 216300791 |
| XRCC5 | chr2 | 216981607 | 217071016 |
| RPL37A | chr2 | 217363519 | 217366188 |
| ARPC2 | chr2 | 219100063 | 219119071 |
| TMBIM1 | chr2 | 219138916 | 219151572 |
| RQCD1 | chr2 | 219433302 | 219461158 |
| ACSL3 | chr2 | 223725731 | 223808119 |
| FAM124B | chr2 | 225243414 | 225266711 |
| CUL3 | chr2 | 225334866 | 225450114 |
| MIR5703 | chr2 | 228336847 | 228336903 |
| AGFG1 | chr2 | 228336887 | 228425938 |
| TRIP12 | chr2 | 230631929 | 230786655 |
| SP100 | chr2 | 231280870 | 231410317 |
| CAB39 | chr2 | 231578262 | 231685790 |
| PSMD1 | chr2 | 231921577 | 232037540 |
| NCL | chr2 | 232319458 | 232329205 |
| PTMA | chr2 | 232574490 | 232578250 |
| GIGYF2 | chr2 | 233562014 | 233725289 |
| HJURP | chr2 | 234745485 | 234763212 |
| SH3BP4 | chr2 | 235887328 | 235964358 |
| LRRFIP1 | chr2 | 238600806 | 238674558 |
| ILKAP | chr2 | 239079042 | 239112324 |
| HDLBP | chr2 | 242166681 | 242255115 |
| 2-Sep | chr2 | 242255280 | 242293441 |
| STK25 | chr2 | 242434121 | 242448110 |
| SH3YL1 | chr2 | 218135 | 264068 |
| ACP1 | chr2 | 264868 | 278282 |
| TMEM18 | chr2 | 667972 | 677439 |
| PXDN | chr2 | 1635658 | 1748291 |
| TSSC1 | chr2 | 3192740 | 3381653 |
| TRAPPC12 | chr2 | 3383445 | 3483342 |
| RNASEH1 | chr2 | 3592690 | 3605940 |
| LOC100506054 | chr2 | 3605975 | 3609340 |
| ITGB1BP1 | chr2 | 9546789 | 9563379 |
| CPSF3 | chr2 | 9564012 | 9613239 |
| ADAM17 | chr2 | 9629410 | 9695917 |
| TAF1B | chr2 | 9983570 | 10074545 |
| RRM2 | chr2 | 10262694 | 10271546 |
| HPCAL1 | chr2 | 10443825 | 10567743 |
| ODC1 | chr2 | 10580507 | 10587906 |
| SNORA80B | chr2 | 10586839 | 10586975 |
| NOL10 | chr2 | 10710891 | 10830113 |
| E2F6 | chr2 | 11584500 | 11606303 |
| TRIB2 | chr2 | 12856997 | 12882858 |
| NBAS | chr2 | 15307031 | 15701472 |
| DDX1 | chr2 | 15731744 | 15771235 |
| FAM49A | chr2 | 16730729 | 16847134 |
| SMC6 | chr2 | 17845078 | 17981462 |
| GEN1 | chr2 | 17935413 | 17966632 |
| TTC32 | chr2 | 20096513 | 20101744 |
| PUM2 | chr2 | 20448452 | 20550463 |
| ATAD2B | chr2 | 23971533 | 24149936 |
| UBXN2A | chr2 | 24150154 | 24223693 |
| SF3B14 | chr2 | 24290453 | 24299314 |
| TP53I3 | chr2 | 24300302 | 24307201 |
| ITSN2 | chr2 | 24425734 | 24583397 |
| PTRHD1 | chr2 | 25013135 | 25016251 |
| DNAJC27 | chr2 | 25166504 | 25194963 |
| DNAJC27-AS1 | chr2 | 25194980 | 25262563 |
| ASXL2 | chr2 | 25962252 | 26101312 |
| HADHA | chr2 | 26413503 | 26467594 |
| HADHB | chr2 | 26467615 | 26513333 |
| GPR113 | chr2 | 26531040 | 26569685 |
| EPT1 | chr2 | 26568953 | 26618759 |
| SLC35F6 | chr2 | 26987141 | 27004099 |
| TMEM214 | chr2 | 27255773 | 27264565 |
| OST4 | chr2 | 27293341 | 27294567 |
| PREB | chr2 | 27353624 | 27357542 |
| SLC5A6 | chr2 | 27422454 | 27435175 |
| ATRAID | chr2 | 27434898 | 27440046 |
| CAD | chr2 | 27440257 | 27466654 |
| MPV17 | chr2 | 27532359 | 27545969 |
| GTF3C2 | chr2 | 27548715 | 27579359 |
| EIF2B4 | chr2 | 27587218 | 27593196 |
| SNX17 | chr2 | 27593362 | 27600400 |
| ZNF513 | chr2 | 27600097 | 27603611 |
| FTH1P3 | chr2 | 27615489 | 27616443 |
| NRBP1 | chr2 | 27650656 | 27665124 |
| CCDC121 | chr2 | 27848505 | 27851745 |
| GPN1 | chr2 | 27851862 | 27873713 |
| SUPT7L | chr2 | 27873678 | 27886449 |
| SLC4A1AP | chr2 | 27886337 | 27917847 |
| MRPL33 | chr2 | 27994583 | 28002608 |
| RBKS | chr2 | 28032165 | 28113223 |
| BRE-AS1 | chr2 | 28112322 | 28113981 |
| BRE | chr2 | 28113481 | 28561767 |
| TRMT61B | chr2 | 29072687 | 29093175 |
| CLIP4 | chr2 | 29338307 | 29406679 |
| SLC30A6 | chr2 | 32390909 | 32449181 |
| YIPF4 | chr2 | 32502957 | 32531658 |
| FAM98A | chr2 | 33808728 | 33814915 |
| LOC100288911 | chr2 | 36581891 | 36582713 |
| STRN | chr2 | 37064840 | 37193615 |
| HEATR5B | chr2 | 37208152 | 37311485 |
| GPATCH11 | chr2 | 37311593 | 37323738 |
| EIF2AK2 | chr2 | 37332283 | 37384190 |
| LOC100505876 | chr2 | 37423634 | 37431886 |
| CEBPZ | chr2 | 37428774 | 37458740 |
| NDUFAF7 | chr2 | 37458773 | 37476303 |
| PRKD3 | chr2 | 37477645 | 37544222 |
| ATL2 | chr2 | 38522028 | 38603672 |
| DHX57 | chr2 | 39024875 | 39103021 |
| SOS1 | chr2 | 39208689 | 39347604 |
| MAP4K3 | chr2 | 39476406 | 39664453 |
| LOC728730 | chr2 | 39664556 | 39828484 |
| THUMPD2 | chr2 | 39963199 | 40006416 |
| LOC100129726 | chr2 | 43454349 | 43455994 |
| THADA | chr2 | 43789357 | 43823185 |
| PPM1B | chr2 | 44395999 | 44461742 |
| PREPL | chr2 | 44544747 | 44587813 |
| CAMKMT | chr2 | 44589042 | 44999731 |
| SRBD1 | chr2 | 45615818 | 45838433 |
| PIGF | chr2 | 46808412 | 46844251 |
| CRIPT | chr2 | 46844324 | 46852881 |
| SOCS5 | chr2 | 46926348 | 46989927 |
| TTC7A | chr2 | 47168312 | 47303275 |
| MSH2 | chr2 | 47630205 | 47906510 |
| MSH6 | chr2 | 48010220 | 48034092 |
| FBXO11 | chr2 | 48034058 | 48115858 |
| ASB3 | chr2 | 53897116 | 54013985 |
| CHAC2 | chr2 | 53994928 | 54002287 |
| ERLEC1 | chr2 | 54014067 | 54045956 |
| SPTBN1 | chr2 | 54683453 | 54898583 |
| CLHC1 | chr2 | 55399686 | 55459449 |
| RPS27A | chr2 | 55459813 | 55462989 |
| MIR4426 | chr2 | 55461299 | 55461985 |
| CCDC88A | chr2 | 55514977 | 55647057 |
| SMEK2 | chr2 | 55775513 | 55845113 |
| PNPT1 | chr2 | 55861197 | 55921045 |
| MIR217 | chr2 | 56210101 | 56210211 |
| MIR216A | chr2 | 56216084 | 56216194 |
| CCDC85A | chr2 | 56411257 | 56613309 |
| VRK2 | chr2 | 58134785 | 58387055 |
| FANCL | chr2 | 58386377 | 58468515 |
| PAPOLG | chr2 | 60983364 | 61029221 |
| FLJ16341 | chr2 | 61074894 | 61108449 |
| REL | chr2 | 61108751 | 61150178 |
| PUS10 | chr2 | 61167547 | 61245365 |
| PEX13 | chr2 | 61244811 | 61279125 |
| AHSA2 | chr2 | 61404554 | 61414686 |
| USP34 | chr2 | 61414589 | 61697849 |
| WDPCP | chr2 | 63348534 | 63670364 |
| MDH1 | chr2 | 63815742 | 63834330 |
| UGP2 | chr2 | 64069013 | 64118696 |
| VPS54 | chr2 | 64119666 | 64163855 |
| AFTPH | chr2 | 64751438 | 64820138 |
| MIR4434 | chr2 | 64752646 | 64752699 |
| SERTAD2 | chr2 | 64858754 | 64881046 |
| CEP68 | chr2 | 65283494 | 65314142 |
| ACTR2 | chr2 | 65454828 | 65498390 |
| MEIS1 | chr2 | 66662531 | 66799891 |
| ETAA1 | chr2 | 67624441 | 67637533 |
| C1D | chr2 | 68269331 | 68290159 |
| WDR92 | chr2 | 68357280 | 68384692 |
| PNO1 | chr2 | 68385004 | 68403094 |
| GFPT1 | chr2 | 69546900 | 69614386 |
| NFU1 | chr2 | 69623244 | 69664760 |
| GMCL1 | chr2 | 70056817 | 70106727 |
| SNRNP27 | chr2 | 70121074 | 70132368 |
| MXD1 | chr2 | 70142172 | 70170076 |
| PCBP1-AS1 | chr2 | 70189394 | 70314147 |
| SNRPG | chr2 | 70508505 | 70520869 |
| TEX261 | chr2 | 71213067 | 71222001 |
| NAGK | chr2 | 71295407 | 71305998 |
| MCEE | chr2 | 71336805 | 71357394 |
| MPHOSPH10 | chr2 | 71357443 | 71377232 |
| ZNF638 | chr2 | 71558884 | 71662191 |
| DYSF | chr2 | 71693831 | 71913893 |
| SFXN5 | chr2 | 73169164 | 73298965 |
| PRADC1 | chr2 | 73455133 | 73460356 |
| TPRKB | chr2 | 73956956 | 73961715 |
| DUSP11 | chr2 | 73994545 | 74007284 |
| DGUOK | chr2 | 74153952 | 74186088 |
| MOB1A | chr2 | 74391148 | 74405995 |
| MTHFD2 | chr2 | 74425689 | 74442424 |
| DCTN1 | chr2 | 74588280 | 74607482 |
| DCTN1-AS1 | chr2 | 74612844 | 74621008 |
| CCDC142 | chr2 | 74699958 | 74710357 |
| AUP1 | chr2 | 74753774 | 74756974 |
| HTRA2 | chr2 | 74756531 | 74760683 |
| EVA1A | chr2 | 75719443 | 75788092 |
| GCFC2 | chr2 | 75889831 | 75937732 |
| SUCLG1 | chr2 | 84650646 | 84686586 |
| TMSB10 | chr2 | 85132762 | 85133799 |
| RETSAT | chr2 | 85569077 | 85581821 |
| ELMOD3 | chr2 | 85581842 | 85618875 |
| LOC100630918 | chr2 | 85764589 | 85766009 |
| GGCX | chr2 | 85771977 | 85788657 |
| VAMP5 | chr2 | 85811530 | 85820511 |
| RNF181 | chr2 | 85822836 | 85824831 |
| USP39 | chr2 | 85843214 | 85876407 |
| ATOH8 | chr2 | 85980908 | 86018506 |
| POLR1A | chr2 | 86253450 | 86333278 |
| PTCD3 | chr2 | 86333304 | 86369280 |
| MIR4779 | chr2 | 86420148 | 86420231 |
| MRPL35 | chr2 | 86426555 | 86440477 |
| KDM3A | chr2 | 86668270 | 86719839 |
| RNF103-CHMP3 | chr2 | 86730552 | 86948245 |
| CHMP3 | chr2 | 86730552 | 86790620 |
| RNF103 | chr2 | 86830515 | 86841821 |
| RMND5A | chr2 | 86947413 | 87005164 |
| LOC285074 | chr2 | 87257797 | 87303536 |
| KRCC1 | chr2 | 88326723 | 88355248 |
| EIF2AK3 | chr2 | 88856258 | 88927094 |
| RPIA | chr2 | 88991175 | 89050452 |
| LOC654342 | chr2 | 91824708 | 91847975 |
| MRPS5 | chr2 | 95769497 | 95787754 |
| ZNF2 | chr2 | 95831182 | 95850064 |
| LOC285033 | chr2 | 96874153 | 96908362 |
| TMEM127 | chr2 | 96915945 | 96931751 |
| CIAO1 | chr2 | 96931883 | 96939900 |
| SNRNP200 | chr2 | 96940073 | 96971307 |
| NCAPH | chr2 | 97001483 | 97041274 |
| ARID5A | chr2 | 97203615 | 97218371 |
| KANSL3 | chr2 | 97258891 | 97304116 |
| ANKRD36 | chr2 | 97779232 | 97930257 |
| ANKRD36B | chr2 | 98121260 | 98206428 |
| ACTR1B | chr2 | 98272401 | 98280561 |
| TMEM131 | chr2 | 98372800 | 98612354 |
| INPP4A | chr2 | 99061320 | 99207496 |
| COA5 | chr2 | 99215785 | 99224981 |
| UNC50 | chr2 | 99225041 | 99234977 |
| MGAT4A | chr2 | 99235568 | 99347589 |
| MITD1 | chr2 | 99785725 | 99797492 |
| MRPL30 | chr2 | 99797541 | 99816020 |
| TXNDC9 | chr2 | 99935486 | 99952860 |
| NPAS2 | chr2 | 101436612 | 101613287 |
| CNOT11 | chr2 | 101869344 | 101886778 |
| MIR5696 | chr2 | 101925911 | 101925996 |
| IL1RL1 | chr2 | 102953716 | 102961182 |
| TGFBRAP1 | chr2 | 105883539 | 105946148 |
| C2orf49 | chr2 | 105954012 | 105961984 |
| NCK2 | chr2 | 106361519 | 106510730 |
| GCC2 | chr2 | 109065576 | 109125854 |
| LIMS1 | chr2 | 109150810 | 109303702 |
| RANBP2 | chr2 | 109335936 | 109402267 |
| SH3RF3-AS1 | chr2 | 109743783 | 109746575 |
| SH3RF3 | chr2 | 109745996 | 110262207 |
| SOWAHC | chr2 | 110371910 | 110376564 |
| LIMS3-LOC440895 | chr2 | 110656008 | 110726149 |
| LIMS3L | chr2 | 110656008 | 110683202 |
| LIMS3 | chr2 | 110656008 | 110683202 |
| MALL | chr2 | 110841446 | 110874143 |
| BUB1 | chr2 | 111395408 | 111435684 |
| ANAPC1 | chr2 | 112525213 | 112641741 |
| MERTK | chr2 | 112656190 | 112786945 |
| TMEM87B | chr2 | 112812799 | 112876895 |
| ZC3H6 | chr2 | 113033177 | 113097640 |
| RGPD8 | chr2 | 113125945 | 113191222 |
| TTL | chr2 | 113239742 | 113290222 |
| POLR1B | chr2 | 113299491 | 113334727 |
| CHCHD5 | chr2 | 113342035 | 113346617 |
| FLJ42351 | chr2 | 113399406 | 113401757 |
| CKAP2L | chr2 | 113495443 | 113522254 |
| SLC35F5 | chr2 | 114471932 | 114514400 |
| DDX18 | chr2 | 118572254 | 118589953 |
| CCDC93 | chr2 | 118673053 | 118771739 |
| INSIG2 | chr2 | 118846049 | 118867597 |
| C2orf76 | chr2 | 120060019 | 120124258 |
| DBI | chr2 | 120124828 | 120130122 |
| RALB | chr2 | 121010413 | 121052286 |
| CLASP1 | chr2 | 122095351 | 122407052 |
| RNU4ATAC | chr2 | 122288455 | 122288585 |
| LOC254128 | chr2 | 122407229 | 122486136 |
| MKI67IP | chr2 | 122484520 | 122494503 |
| TSN | chr2 | 122513120 | 122525428 |
| ERCC3 | chr2 | 128014865 | 128051752 |
| IWS1 | chr2 | 128238382 | 128284087 |
| POLR2D | chr2 | 128603839 | 128615729 |
| AMMECR1L | chr2 | 128619206 | 128643514 |
| UGGT1 | chr2 | 128848781 | 128953249 |
| SMPD4 | chr2 | 130908964 | 130940323 |
| MZT2B | chr2 | 130939500 | 130948300 |
| IMP4 | chr2 | 131100488 | 131104197 |
| FAM168B | chr2 | 131805448 | 131851004 |
| PLEKHB2 | chr2 | 131877807 | 131907425 |
| MZT2A | chr2 | 132241532 | 132250064 |
| MIR4784 | chr2 | 132248732 | 132248809 |
| LOC150776 | chr2 | 132250385 | 132279149 |
| LYPD1 | chr2 | 133402336 | 133428481 |
| MIR3679 | chr2 | 134884695 | 134884763 |
| LOC100129961 | chr2 | 135624202 | 135676176 |
| RAB3GAP1 | chr2 | 135809834 | 135928279 |
| ZRANB3 | chr2 | 135957573 | 136288300 |
| R3HDM1 | chr2 | 136343921 | 136482839 |
| MCM6 | chr2 | 136597195 | 136634047 |
| HNMT | chr2 | 138721807 | 138773934 |
| SPOPL | chr2 | 139259349 | 139330805 |
| ZEB2 | chr2 | 145141941 | 145277958 |
| ZEB2-AS1 | chr2 | 145277180 | 145278465 |
| ORC4 | chr2 | 148687965 | 148778463 |
| MBD5 | chr2 | 148778579 | 149271044 |
| EPC2 | chr2 | 149402559 | 149545136 |
| MMADHC | chr2 | 150426146 | 150444330 |
| RBM43 | chr2 | 152104727 | 152118389 |
| NMI | chr2 | 152126981 | 152146430 |
| RIF1 | chr2 | 152266603 | 152333860 |
| STAM2 | chr2 | 152973314 | 153032506 |
| ARL6IP6 | chr2 | 153574406 | 153617767 |
| ACVR1 | chr2 | 158592957 | 158731623 |
| PKP4 | chr2 | 159313475 | 159537940 |
| BAZ2B | chr2 | 160175489 | 160473112 |
| RBMS1 | chr2 | 161128661 | 161350318 |
| TANK | chr2 | 161993465 | 162092683 |
| FIGN | chr2 | 164464117 | 164592513 |
| TTC21B | chr2 | 166729871 | 166810348 |
| SSB | chr2 | 170655788 | 170668571 |
| METTL5 | chr2 | 170668267 | 170681353 |
| UBR3 | chr2 | 170684017 | 170940639 |
| TLK1 | chr2 | 171847332 | 172017410 |
| METTL8 | chr2 | 172173912 | 172291312 |
| HAT1 | chr2 | 172778934 | 172848600 |
| ITGA6 | chr2 | 173292313 | 173371181 |
| PDK1 | chr2 | 173420778 | 173463862 |
| ZAK | chr2 | 173940564 | 174091873 |
| CDCA7 | chr2 | 174219560 | 174233718 |
| SP3 | chr2 | 174771186 | 174830430 |
| OLA1 | chr2 | 174937174 | 175113365 |
| CIR1 | chr2 | 175212877 | 175260443 |
| ATP5G3 | chr2 | 176040985 | 176046490 |
| HOXD8 | chr2 | 176994467 | 176997423 |
| HOXD-AS2 | chr2 | 176999568 | 177001826 |
| MTX2 | chr2 | 177134122 | 177202753 |
| AC074286.1 | chr2 | 178148235 | 178257419 |
| TTC30B | chr2 | 178414880 | 178417524 |
| PRKRA | chr2 | 179296140 | 179315484 |
| FKBP7 | chr2 | 179328390 | 179343355 |
| PLEKHA3 | chr2 | 179345198 | 179369782 |
| SESTD1 | chr2 | 179966418 | 180129350 |
| CWC22 | chr2 | 180809603 | 180871780 |
| UBE2E3 | chr2 | 181845341 | 181928150 |
| DNAJC10 | chr2 | 183580767 | 183644750 |
| NUP35 | chr2 | 183989082 | 184026408 |
| ITGAV | chr2 | 187464931 | 187545629 |
| MIR561 | chr2 | 189162218 | 189162315 |
| GULP1 | chr2 | 189434754 | 189459786 |
| COL5A2 | chr2 | 189896640 | 190044605 |
| ASNSD1 | chr2 | 190526124 | 190535557 |
| ORMDL1 | chr2 | 190634992 | 190648108 |
| PMS1 | chr2 | 190648810 | 190742355 |
| INPP1 | chr2 | 191208195 | 191236391 |
| NAB1 | chr2 | 191513847 | 191557492 |
| STAT1 | chr2 | 191833761 | 191878681 |
| NABP1 | chr2 | 192542860 | 192553248 |
| SLC39A10 | chr2 | 196521531 | 196602426 |
| STK17B | chr2 | 196998306 | 197036336 |
| GTF3C3 | chr2 | 197627755 | 197664492 |
| PGAP1 | chr2 | 197697727 | 197791454 |
| COQ10B | chr2 | 198318230 | 198339851 |
| HSPE1-MOB4 | chr2 | 198364720 | 198418423 |
| HSPE1 | chr2 | 198364720 | 198368187 |
| MOB4 | chr2 | 198380294 | 198418423 |
| MARS2 | chr2 | 198570027 | 198573114 |
| C2orf69 | chr2 | 200775978 | 200792996 |
| TYW5 | chr2 | 200793633 | 200820459 |
| C2orf47 | chr2 | 200820039 | 200828847 |
| SPATS2L | chr2 | 201170615 | 201346986 |
| SGOL2 | chr2 | 201390864 | 201448818 |
| PPIL3 | chr2 | 201735678 | 201753849 |
| NIF3L1 | chr2 | 201755928 | 201768655 |
| ORC2 | chr2 | 201774893 | 201828424 |
| FAM126B | chr2 | 201838440 | 201936392 |
| NDUFB3 | chr2 | 201936461 | 201950473 |
| CFLAR | chr2 | 202004997 | 202037411 |
| CFLAR-AS1 | chr2 | 202005011 | 202022515 |
| CASP10 | chr2 | 202047620 | 202086383 |
| CASP8 | chr2 | 202122753 | 202152434 |
| TMEM237 | chr2 | 202484906 | 202507667 |
| MPP4 | chr2 | 202509596 | 202563417 |
| ALS2 | chr2 | 202564985 | 202645895 |
| SUMO1 | chr2 | 203070902 | 203103322 |
| SNORD70 | chr2 | 203141153 | 203141241 |
| WDR12 | chr2 | 203745322 | 203776949 |
| ALS2CR8 | chr2 | 203777116 | 203851060 |
| NBEAL1 | chr2 | 203879601 | 204082717 |
| CYP20A1 | chr2 | 204103163 | 204170563 |
| ABI2 | chr2 | 204193002 | 204296892 |
| NRP2 | chr2 | 206547223 | 206662857 |
| INO80D | chr2 | 206858444 | 206950906 |
| NDUFS1 | chr2 | 206987802 | 207023918 |
| EEF1B2 | chr2 | 207024317 | 207027653 |
| SNORD51 | chr2 | 207026604 | 207026674 |
| SNORA41 | chr2 | 207026951 | 207027083 |
| MDH1B | chr2 | 207602488 | 207630050 |
| FASTKD2 | chr2 | 207630111 | 207660911 |
| MIR2355 | chr2 | 207974710 | 207974797 |
| CREB1 | chr2 | 208394615 | 208470284 |
| CCNYL1 | chr2 | 208576263 | 208620896 |
| PIKFYVE | chr2 | 209130990 | 209223475 |
| KANSL1L | chr2 | 210885434 | 211036051 |
| BARD1 | chr2 | 215593274 | 215674428 |
| LINC00607 | chr2 | 216476285 | 216708259 |
| 4-Mar | chr2 | 217122584 | 217236750 |
| CTDSP1 | chr2 | 219264477 | 219270664 |
| USP37 | chr2 | 219314973 | 219433084 |
| ZNF142 | chr2 | 219502639 | 219524355 |
| BCS1L | chr2 | 219524494 | 219528166 |
| RNF25 | chr2 | 219528586 | 219536781 |
| TTLL4 | chr2 | 219575567 | 219620138 |
| CNPPD1 | chr2 | 220036618 | 220042732 |
| FAM134A | chr2 | 220042938 | 220050197 |
| ATG9A | chr2 | 220082846 | 220094361 |
| ANKZF1 | chr2 | 220095346 | 220100034 |
| GLB1L | chr2 | 220101502 | 220110131 |
| STK16 | chr2 | 220110200 | 220115059 |
| FARSB | chr2 | 223436161 | 223520827 |
| WDFY1 | chr2 | 224740064 | 224810052 |
| MRPL44 | chr2 | 224822120 | 224832431 |
| CUL3 | chr2 | 225334866 | 225434542 |
| MFF | chr2 | 228192227 | 228222552 |
| FBXO36 | chr2 | 230787206 | 230877825 |
| SP140L | chr2 | 231191893 | 231268445 |
| SP100 | chr2 | 231281470 | 231335250 |
| SNORD82 | chr2 | 232325078 | 232325153 |
| MIR1244-2 | chr2 | 232578023 | 232578105 |
| COPS7B | chr2 | 232658225 | 232673434 |
| TIGD1 | chr2 | 233412778 | 233415283 |
| MIR5001 | chr2 | 233415183 | 233415283 |
| EIF4E2 | chr2 | 233415356 | 233448349 |
| INPP5D | chr2 | 233925035 | 234116549 |
| ATG16L1 | chr2 | 234160216 | 234204320 |
| CXCR7 | chr2 | 237469423 | 237490994 |
| COPS8 | chr2 | 237994083 | 238007489 |
| ASB1 | chr2 | 239335625 | 239360891 |
| NDUFA10 | chr2 | 240896788 | 240964819 |
| MYEOV2 | chr2 | 241065979 | 241075764 |
| RNPEPL1 | chr2 | 241508003 | 241518149 |
| CAPN10 | chr2 | 241526132 | 241538526 |
| MTERFD2 | chr2 | 242026508 | 242041747 |
| FARP2 | chr2 | 242295663 | 242434256 |
| BOK | chr2 | 242498145 | 242513553 |
| THAP4 | chr2 | 242523819 | 242576725 |
| ATG4B | chr2 | 242577026 | 242613271 |
| DTYMK | chr2 | 242615156 | 242626383 |
| CSNK2A1 | chr20 | 463337 | 524482 |
| FKBP1A-SDCBP2 | chr20 | 1290554 | 1373816 |
| SDCBP2-AS1 | chr20 | 1305986 | 1359379 |
| FKBP1A | chr20 | 1349620 | 1373816 |
| SNRPB | chr20 | 2442287 | 2451499 |
| NOP56 | chr20 | 2633177 | 2639039 |
| MIR1292 | chr20 | 2633422 | 2633488 |
| UBOX5 | chr20 | 3088218 | 3140556 |
| FASTKD5 | chr20 | 3127164 | 3140556 |
| CENPB | chr20 | 3764497 | 3767337 |
| PANK2 | chr20 | 3869741 | 3904502 |
| PCNA | chr20 | 5095598 | 5100647 |
| DSTN | chr20 | 17550598 | 17588652 |
| SNX5 | chr20 | 17922243 | 17949490 |
| SNORD17 | chr20 | 17943352 | 17943589 |
| RIN2 | chr20 | 19867164 | 19983103 |
| NAA20 | chr20 | 19998438 | 20014273 |
| CD93 | chr20 | 23059992 | 23066977 |
| NXT1 | chr20 | 23331372 | 23335408 |
| HM13 | chr20 | 30102240 | 30127596 |
| BCL2L1 | chr20 | 30252260 | 30310656 |
| TPX2 | chr20 | 30326903 | 30389603 |
| ASXL1 | chr20 | 30946146 | 31027122 |
| C20orf112 | chr20 | 31030861 | 31071385 |
| MAPRE1 | chr20 | 31407698 | 31438211 |
| CHMP4B | chr20 | 32399109 | 32442173 |
| RALY | chr20 | 32581457 | 32670991 |
| EIF2S2 | chr20 | 32676114 | 32700085 |
| ITCH | chr20 | 32951040 | 33099198 |
| TRPC4AP | chr20 | 33590206 | 33680618 |
| PROCR | chr20 | 33759773 | 33765165 |
| CPNE1 | chr20 | 34213952 | 34252878 |
| RBM12 | chr20 | 34236846 | 34252878 |
| RBM39 | chr20 | 34291530 | 34330158 |
| LINC00657 | chr20 | 34633539 | 34638882 |
| DLGAP4 | chr20 | 35126219 | 35157040 |
| RPN2 | chr20 | 35807455 | 35870025 |
| TGM2 | chr20 | 36756863 | 36793700 |
| SNHG17 | chr20 | 37049238 | 37064018 |
| SNORA71D | chr20 | 37062504 | 37062642 |
| FAM83D | chr20 | 37554954 | 37574842 |
| TOP1 | chr20 | 39657461 | 39753126 |
| PLCG1 | chr20 | 39766160 | 39804357 |
| OSER1 | chr20 | 42824580 | 42839546 |
| YWHAB | chr20 | 43514343 | 43537161 |
| ZMYND8 | chr20 | 45838380 | 45984401 |
| LOC100131496 | chr20 | 45947245 | 45949498 |
| PREX1 | chr20 | 47240792 | 47444420 |
| CSE1L | chr20 | 47662782 | 47713497 |
| STAU1 | chr20 | 47729875 | 47804904 |
| ZFAS1 | chr20 | 47894714 | 47905795 |
| SNORD12C | chr20 | 47895481 | 47895560 |
| SNORD12B | chr20 | 47896849 | 47896952 |
| SNORD12 | chr20 | 47897219 | 47897309 |
| B4GALT5 | chr20 | 48249482 | 48330421 |
| SNAI1 | chr20 | 48599512 | 48605420 |
| TMEM189-UBE2V1 | chr20 | 48697660 | 48770335 |
| UBE2V1 | chr20 | 48697660 | 48732496 |
| TMEM189 | chr20 | 48740273 | 48770335 |
| PTPN1 | chr20 | 49126890 | 49201086 |
| ADNP | chr20 | 49506882 | 49547527 |
| DPM1 | chr20 | 49551404 | 49575060 |
| VAPB | chr20 | 56964174 | 57026156 |
| STX16 | chr20 | 57226308 | 57254582 |
| STX16-NPEPL1 | chr20 | 57226308 | 57290900 |
| GNAS | chr20 | 57472494 | 57486250 |
| ATP5E | chr20 | 57603732 | 57607422 |
| PSMA7 | chr20 | 60711782 | 60718514 |
| SS18L1 | chr20 | 60718821 | 60757566 |
| ADRM1 | chr20 | 60878026 | 60883918 |
| DIDO1 | chr20 | 61509089 | 61569304 |
| ARFGAP1 | chr20 | 61904164 | 61921142 |
| DNAJC5 | chr20 | 62526454 | 62567384 |
| C20orf96 | chr20 | 251503 | 271419 |
| ZCCHC3 | chr20 | 278203 | 280963 |
| SOX12 | chr20 | 306214 | 310872 |
| TRIB3 | chr20 | 361307 | 378203 |
| RBCK1 | chr20 | 388708 | 411610 |
| TBC1D20 | chr20 | 416123 | 443187 |
| SRXN1 | chr20 | 627267 | 634014 |
| FAM110A | chr20 | 814355 | 826922 |
| PSMF1 | chr20 | 1099239 | 1148426 |
| SDCBP2 | chr20 | 1290554 | 1309879 |
| NSFL1C | chr20 | 1422806 | 1448337 |
| SIRPB2 | chr20 | 1455235 | 1472233 |
| STK35 | chr20 | 2082527 | 2129198 |
| SNORD110 | chr20 | 2634857 | 2634932 |
| SNORA51 | chr20 | 2635712 | 2635844 |
| SNORD86 | chr20 | 2636742 | 2636828 |
| SNORD56 | chr20 | 2637269 | 2637340 |
| SNORD57 | chr20 | 2637584 | 2637656 |
| IDH3B | chr20 | 2639040 | 2644865 |
| PTPRA | chr20 | 2854141 | 3019315 |
| DDRGK1 | chr20 | 3174652 | 3185295 |
| ITPA | chr20 | 3190005 | 3204516 |
| C20orf194 | chr20 | 3229947 | 3388309 |
| ATRN | chr20 | 3451664 | 3631769 |
| CDC25B | chr20 | 3776400 | 3786761 |
| AP5S1 | chr20 | 3801170 | 3805954 |
| MAVS | chr20 | 3827445 | 3856770 |
| MIR103A2 | chr20 | 3898140 | 3898218 |
| MIR103B2 | chr20 | 3898148 | 3898210 |
| PRNP | chr20 | 4666796 | 4682234 |
| TMEM230 | chr20 | 5080483 | 5093733 |
| PCNA-AS1 | chr20 | 5100231 | 5100615 |
| CDS2 | chr20 | 5107406 | 5178533 |
| GPCPD1 | chr20 | 5525079 | 5566683 |
| TRMT6 | chr20 | 5918485 | 5931173 |
| MCM8 | chr20 | 5931297 | 5975831 |
| CRLS1 | chr20 | 5986738 | 6020697 |
| MKKS | chr20 | 10385427 | 10412573 |
| SLX4IP | chr20 | 10415950 | 10604027 |
| JAG1 | chr20 | 10618331 | 10654694 |
| BTBD3 | chr20 | 11871476 | 11907243 |
| ESF1 | chr20 | 13694968 | 13765532 |
| NDUFAF5 | chr20 | 13765671 | 13799067 |
| SNRPB2 | chr20 | 16710608 | 16722417 |
| BFSP1 | chr20 | 17474549 | 17539605 |
| RRBP1 | chr20 | 17594322 | 17662928 |
| MGME1 | chr20 | 17949761 | 17971762 |
| ZNF133 | chr20 | 18269120 | 18297640 |
| DZANK1 | chr20 | 18364010 | 18447829 |
| POLR3F | chr20 | 18448032 | 18465286 |
| SEC23B | chr20 | 18488187 | 18542059 |
| RIN2 | chr20 | 19870209 | 19983103 |
| RALGAPA2 | chr20 | 20370271 | 20693266 |
| XRN2 | chr20 | 21283941 | 21370463 |
| GZF1 | chr20 | 23342818 | 23353683 |
| CST3 | chr20 | 23608533 | 23618574 |
| PYGB | chr20 | 25228705 | 25278648 |
| GINS1 | chr20 | 25388322 | 25429191 |
| NANP | chr20 | 25593572 | 25604648 |
| FRG1B | chr20 | 29611878 | 29634007 |
| PSIMCT-1 | chr20 | 30135184 | 30136019 |
| HM13-AS1 | chr20 | 30155509 | 30161066 |
| ID1 | chr20 | 30193085 | 30194317 |
| MIR3193 | chr20 | 30194988 | 30195043 |
| PDRG1 | chr20 | 30532757 | 30539883 |
| TM9SF4 | chr20 | 30697538 | 30755061 |
| PLAGL2 | chr20 | 30780306 | 30795546 |
| POFUT1 | chr20 | 30795695 | 30826467 |
| CDK5RAP1 | chr20 | 31946646 | 31989375 |
| MIR4755 | chr20 | 32636924 | 32636996 |
| AHCY | chr20 | 32868070 | 32891215 |
| DYNLRB1 | chr20 | 33104203 | 33123391 |
| NCOA6 | chr20 | 33302577 | 33409442 |
| EDEM2 | chr20 | 33705827 | 33865960 |
| EIF6 | chr20 | 33866708 | 33872520 |
| UQCC | chr20 | 33931919 | 33982103 |
| CEP250 | chr20 | 34043222 | 34099803 |
| CPNE1 | chr20 | 34213952 | 34241831 |
| NFS1 | chr20 | 34256609 | 34287111 |
| ROMO1 | chr20 | 34287231 | 34288902 |
| PHF20 | chr20 | 34359922 | 34538288 |
| SCAND1 | chr20 | 34541550 | 34547394 |
| AAR2 | chr20 | 34824338 | 34844863 |
| TGIF2 | chr20 | 35201875 | 35222355 |
| TGIF2-C20orf24 | chr20 | 35202956 | 35240960 |
| C20orf24 | chr20 | 35234136 | 35240960 |
| DSN1 | chr20 | 35380193 | 35402230 |
| SOGA1 | chr20 | 35412177 | 35492087 |
| RBL1 | chr20 | 35626177 | 35724410 |
| MROH8 | chr20 | 35729628 | 35807974 |
| MANBAL | chr20 | 35918050 | 35945663 |
| SRC | chr20 | 35974556 | 36033821 |
| BLCAP | chr20 | 36145818 | 36156333 |
| NNAT | chr20 | 36149606 | 36152090 |
| TTI1 | chr20 | 36611422 | 36661833 |
| RPRD1B | chr20 | 36661947 | 36720766 |
| SNORA71B | chr20 | 37053842 | 37053978 |
| SNORA71A | chr20 | 37055948 | 37056086 |
| SNORA71C | chr20 | 37058309 | 37058447 |
| SNORA39 | chr20 | 37076725 | 37078147 |
| RALGAPB | chr20 | 37101485 | 37207504 |
| PPP1R16B | chr20 | 37434347 | 37551667 |
| DHX35 | chr20 | 37590980 | 37668366 |
| SRSF6 | chr20 | 42086503 | 42092244 |
| OSER1-AS1 | chr20 | 42839725 | 42854667 |
| SERINC3 | chr20 | 43127900 | 43150726 |
| PKIG | chr20 | 43160435 | 43247678 |
| TOMM34 | chr20 | 43570770 | 43589114 |
| STK4 | chr20 | 43595119 | 43708593 |
| SDC4 | chr20 | 43953928 | 43977064 |
| SYS1 | chr20 | 43991739 | 44005442 |
| SYS1-DBNDD2 | chr20 | 43991808 | 44039250 |
| PIGT | chr20 | 44046812 | 44054885 |
| UBE2C | chr20 | 44441671 | 44445596 |
| ZSWIM1 | chr20 | 44509847 | 44513905 |
| PCIF1 | chr20 | 44563316 | 44576662 |
| ZNF335 | chr20 | 44577291 | 44600833 |
| NCOA5 | chr20 | 44689625 | 44708591 |
| SLC35C2 | chr20 | 44978176 | 44991813 |
| TP53RK | chr20 | 45313003 | 45318276 |
| NCOA3 | chr20 | 46130600 | 46285621 |
| ARFGEF2 | chr20 | 47538274 | 47653230 |
| DDX27 | chr20 | 47835831 | 47860614 |
| ZNFX1 | chr20 | 47862438 | 47894756 |
| SLC9A8 | chr20 | 48429249 | 48508779 |
| SPATA2 | chr20 | 48519928 | 48530276 |
| RNF114 | chr20 | 48552913 | 48570422 |
| MOCS3 | chr20 | 49575350 | 49578399 |
| ZNF217 | chr20 | 52183609 | 52199707 |
| PFDN4 | chr20 | 52824501 | 52836492 |
| AURKA | chr20 | 54944444 | 54967351 |
| CSTF1 | chr20 | 54967573 | 54979582 |
| RTFDC1 | chr20 | 55043646 | 55093942 |
| RAE1 | chr20 | 55926144 | 55950408 |
| PMEPA1 | chr20 | 56223447 | 56285031 |
| PPP4R1L | chr20 | 56807743 | 56884495 |
| RAB22A | chr20 | 56884770 | 56942563 |
| GNAS | chr20 | 57464179 | 57486247 |
| NELFCD | chr20 | 57556262 | 57570188 |
| CTSZ | chr20 | 57570241 | 57582309 |
| SLMO2-ATP5E | chr20 | 57603732 | 57617901 |
| SLMO2 | chr20 | 57608199 | 57617901 |
| PPP1R3D | chr20 | 58511886 | 58515352 |
| FAM217B | chr20 | 58515443 | 58523702 |
| MTG2 | chr20 | 60758080 | 60777810 |
| LAMA5 | chr20 | 60884120 | 60942368 |
| RPS21 | chr20 | 60962120 | 60963576 |
| OGFR | chr20 | 61436176 | 61445352 |
| GID8 | chr20 | 61569440 | 61579827 |
| SLC17A9 | chr20 | 61583998 | 61599949 |
| YTHDF1 | chr20 | 61826781 | 61847538 |
| GMEB2 | chr20 | 62218954 | 62251229 |
| ARFRP1 | chr20 | 62329994 | 62339010 |
| ZGPAT | chr20 | 62339904 | 62370460 |
| TPD52L2 | chr20 | 62496580 | 62522898 |
| UCKL1 | chr20 | 62571181 | 62582527 |
| UCKL1-AS1 | chr20 | 62584736 | 62588338 |
| SOX18 | chr20 | 62679078 | 62680979 |
| NRIP1 | chr21 | 16333555 | 16438231 |
| USP25 | chr21 | 17102495 | 17252377 |
| BTG3 | chr21 | 18965967 | 18985268 |
| MIR155HG | chr21 | 26934456 | 26947480 |
| APP | chr21 | 27252860 | 27543138 |
| CCT8 | chr21 | 30428647 | 30446010 |
| BACH1 | chr21 | 30671219 | 30718469 |
| SCAF4 | chr21 | 33043312 | 33104431 |
| PAXBP1 | chr21 | 34106209 | 34144169 |
| SON | chr21 | 34915349 | 34949812 |
| ITSN1 | chr21 | 35014783 | 35261609 |
| RUNX1 | chr21 | 36160097 | 36260987 |
| MORC3 | chr21 | 37692486 | 37748944 |
| DYRK1A | chr21 | 38739858 | 38887679 |
| ERG | chr21 | 39751949 | 39870428 |
| ETS2 | chr21 | 40177230 | 40196878 |
| HMGN1 | chr21 | 40714240 | 40721047 |
| U2AF1 | chr21 | 44513072 | 44527688 |
| PTTG1IP | chr21 | 46269499 | 46293818 |
| COL18A1 | chr21 | 46825096 | 46933634 |
| C21orf58 | chr21 | 47721046 | 47743785 |
| MIR3648 | chr21 | 9825831 | 9826011 |
| MIR3687 | chr21 | 9826202 | 9826263 |
| HSPA13 | chr21 | 15743436 | 15755509 |
| SAMSN1 | chr21 | 15857548 | 15918681 |
| LINC00478 | chr21 | 17566698 | 17982094 |
| MIR155 | chr21 | 26946291 | 26946356 |
| ATP5J | chr21 | 27096790 | 27107965 |
| GABPA | chr21 | 27107328 | 27144771 |
| LINC00161 | chr21 | 29911639 | 29912677 |
| LTN1 | chr21 | 30300465 | 30365277 |
| USP16 | chr21 | 30396937 | 30426807 |
| BACH1 | chr21 | 30671736 | 30718469 |
| SOD1 | chr21 | 33031934 | 33041243 |
| URB1 | chr21 | 33683329 | 33765312 |
| EVA1C | chr21 | 33824492 | 33887697 |
| C21orf59 | chr21 | 33973983 | 33984918 |
| C21orf49 | chr21 | 34144410 | 34170016 |
| IFNAR2 | chr21 | 34602230 | 34636820 |
| IFNAR1 | chr21 | 34697213 | 34732128 |
| IFNGR2 | chr21 | 34775201 | 34809828 |
| GART | chr21 | 34876237 | 34915195 |
| DONSON | chr21 | 34949858 | 34967920 |
| CRYZL1 | chr21 | 34961647 | 35014160 |
| ATP5O | chr21 | 35275756 | 35288158 |
| RCAN1 | chr21 | 35888783 | 35899261 |
| CLDN14 | chr21 | 37832919 | 37852388 |
| PIGP | chr21 | 38437663 | 38445458 |
| TTC3 | chr21 | 38458089 | 38575408 |
| DSCR3 | chr21 | 38595725 | 38639833 |
| DYRK1A | chr21 | 38791206 | 38887679 |
| PSMG1 | chr21 | 40547371 | 40555440 |
| WRB | chr21 | 40752212 | 40769815 |
| C2CD2 | chr21 | 43305218 | 43373999 |
| WDR4 | chr21 | 44269335 | 44299385 |
| NDUFV3 | chr21 | 44299753 | 44329773 |
| PKNOX1 | chr21 | 44394642 | 44453688 |
| LINC00313 | chr21 | 44881973 | 44898103 |
| HSF2BP | chr21 | 44949071 | 45079374 |
| RRP1B | chr21 | 45079431 | 45115960 |
| RRP1 | chr21 | 45212811 | 45223983 |
| AGPAT3 | chr21 | 45366810 | 45407475 |
| TRAPPC10 | chr21 | 45432205 | 45526432 |
| PWP2 | chr21 | 45527207 | 45551063 |
| C21orf33 | chr21 | 45553493 | 45565605 |
| PFKL | chr21 | 45719924 | 45747261 |
| UBE2G2 | chr21 | 46188494 | 46221751 |
| SUMO3 | chr21 | 46225531 | 46238044 |
| C21orf67 | chr21 | 46353198 | 46359828 |
| FAM207A | chr21 | 46359954 | 46396888 |
| ADARB1 | chr21 | 46494492 | 46646478 |
| COL18A1-AS2 | chr21 | 46827300 | 46829980 |
| COL18A1-AS1 | chr21 | 46839630 | 46844955 |
| SLC19A1 | chr21 | 46934628 | 46962385 |
| LSS | chr21 | 47609037 | 47648738 |
| MCM3AP-AS1 | chr21 | 47649157 | 47671604 |
| MCM3AP | chr21 | 47655047 | 47705236 |
| YBEY | chr21 | 47706266 | 47712111 |
| PCNT | chr21 | 47744035 | 47865682 |
| DIP2A | chr21 | 47878861 | 47989926 |
| DIP2A-IT1 | chr21 | 47882383 | 47889218 |
| PRMT2 | chr21 | 48055506 | 48075276 |
| HIRA | chr22 | 19318223 | 19435755 |
| UFD1L | chr22 | 19438143 | 19466738 |
| GNB1L | chr22 | 19775933 | 19842462 |
| C22orf29 | chr22 | 19833660 | 19842339 |
| COMT | chr22 | 19929262 | 19957498 |
| DGCR8 | chr22 | 20067754 | 20099400 |
| RANBP1 | chr22 | 20105023 | 20113711 |
| MED15 | chr22 | 20908525 | 20941919 |
| CRKL | chr22 | 21271713 | 21308037 |
| UBE2L3 | chr22 | 21922018 | 21978323 |
| MAPK1 | chr22 | 22113946 | 22221970 |
| PPM1F | chr22 | 22273791 | 22307250 |
| BCR | chr22 | 23522551 | 23660224 |
| SPECC1L | chr22 | 24666789 | 24813708 |
| SNRPD3 | chr22 | 24951617 | 24978854 |
| PITPNB | chr22 | 28247656 | 28315187 |
| MIR3199-1 | chr22 | 28316512 | 28316600 |
| XBP1 | chr22 | 29190547 | 29196560 |
| EWSR1 | chr22 | 29685501 | 29696515 |
| NF2 | chr22 | 29999544 | 30094589 |
| TUG1 | chr22 | 31365633 | 31375380 |
| SMTN | chr22 | 31482486 | 31500610 |
| LIMK2 | chr22 | 31644347 | 31676066 |
| PISD | chr22 | 32014476 | 32026810 |
| YWHAH | chr22 | 32340478 | 32353590 |
| FBXO7 | chr22 | 32887607 | 32894818 |
| MYH9 | chr22 | 36677322 | 36784063 |
| MFNG | chr22 | 37865100 | 37882478 |
| CDC42EP1 | chr22 | 37956470 | 37965410 |
| TRIOBP | chr22 | 38142240 | 38172563 |
| MAFF | chr22 | 38597938 | 38612517 |
| TMEM184B | chr22 | 38615297 | 38669040 |
| CSNK1E | chr22 | 38686696 | 38794527 |
| DDX17 | chr22 | 38879442 | 38902345 |
| JOSD1 | chr22 | 39081547 | 39096459 |
| GTPBP1 | chr22 | 39101806 | 39129592 |
| PDGFB | chr22 | 39619684 | 39640957 |
| RPL3 | chr22 | 39708886 | 39716391 |
| RNU86 | chr22 | 39712846 | 39712901 |
| SNORD43 | chr22 | 39715056 | 39715118 |
| ATF4 | chr22 | 39916568 | 39918691 |
| TNRC6B | chr22 | 40573928 | 40731812 |
| ST13 | chr22 | 41220600 | 41252687 |
| MIR1281 | chr22 | 41488516 | 41488570 |
| EP300 | chr22 | 41488613 | 41576081 |
| RANGAP1 | chr22 | 41641614 | 41678518 |
| ZC3H7B | chr22 | 41697506 | 41756151 |
| TOB2 | chr22 | 41829491 | 41843027 |
| DESI1 | chr22 | 41994031 | 42017061 |
| SREBF2 | chr22 | 42229105 | 42302375 |
| RRP7A | chr22 | 42904340 | 42912921 |
| POLDIP3 | chr22 | 42979726 | 43010962 |
| PARVB | chr22 | 44420156 | 44565112 |
| NUP50 | chr22 | 45559725 | 45583890 |
| MIRLET7BHG | chr22 | 46481876 | 46509808 |
| MIR3619 | chr22 | 46486923 | 46487006 |
| CERK | chr22 | 47080306 | 47134152 |
| BRD1 | chr22 | 50166936 | 50217979 |
| ZBED4 | chr22 | 50247496 | 50283726 |
| PIM3 | chr22 | 50354142 | 50357720 |
| PLXNB2 | chr22 | 50713407 | 50746001 |
| SHANK3 | chr22 | 51113069 | 51171640 |
| ATP6V1E1 | chr22 | 18074902 | 18111588 |
| BCL2L13 | chr22 | 18121349 | 18213621 |
| MIR648 | chr22 | 18463633 | 18463727 |
| PEX26 | chr22 | 18560759 | 18573797 |
| DGCR2 | chr22 | 19023794 | 19109967 |
| MRPL40 | chr22 | 19420035 | 19423596 |
| C22orf39 | chr22 | 19430672 | 19435755 |
| CDC45 | chr22 | 19467413 | 19508135 |
| CLDN5 | chr22 | 19510546 | 19512860 |
| TXNRD2 | chr22 | 19863046 | 19929359 |
| COMT | chr22 | 19938459 | 19957498 |
| TANGO2 | chr22 | 20008632 | 20043764 |
| MIR3618 | chr22 | 20073268 | 20073356 |
| MIR1306 | chr22 | 20073580 | 20073665 |
| TRMT2A | chr22 | 20099388 | 20104818 |
| ZDHHC8 | chr22 | 20119363 | 20135530 |
| PI4KA | chr22 | 21061978 | 21213100 |
| SNAP29 | chr22 | 21213291 | 21245501 |
| LZTR1 | chr22 | 21333750 | 21353326 |
| THAP7 | chr22 | 21354060 | 21356404 |
| THAP7-AS1 | chr22 | 21356210 | 21364663 |
| HIC2 | chr22 | 21796748 | 21805750 |
| YDJC | chr22 | 21982377 | 21984340 |
| SDF2L1 | chr22 | 21996541 | 21998588 |
| TOP3B | chr22 | 22311402 | 22337147 |
| SMARCB1 | chr22 | 24129149 | 24176705 |
| MIF | chr22 | 24236564 | 24237409 |
| DDTL | chr22 | 24309025 | 24314748 |
| DDT | chr22 | 24313553 | 24316680 |
| GUCD1 | chr22 | 24936405 | 24951275 |
| LOC100128531 | chr22 | 25498383 | 25508659 |
| CRYBB2P1 | chr22 | 25844053 | 25857645 |
| HPS4 | chr22 | 26846848 | 26879829 |
| SRRD | chr22 | 26879849 | 26887904 |
| TFIP11 | chr22 | 26887893 | 26908343 |
| TPST2 | chr22 | 26921713 | 26986089 |
| TTC28-AS1 | chr22 | 28315363 | 28398667 |
| MIR3199-2 | chr22 | 28316513 | 28316599 |
| CHEK2 | chr22 | 29083730 | 29137822 |
| HSCB | chr22 | 29138042 | 29153496 |
| CCDC117 | chr22 | 29168661 | 29185283 |
| ZNRF3-AS1 | chr22 | 29420986 | 29427464 |
| RHBDD3 | chr22 | 29655843 | 29663914 |
| GAS2L1 | chr22 | 29702996 | 29708774 |
| AP1B1 | chr22 | 29723668 | 29784572 |
| THOC5 | chr22 | 29904155 | 29949644 |
| ZMAT5 | chr22 | 30126944 | 30162969 |
| UQCR10 | chr22 | 30163357 | 30166402 |
| MTMR3 | chr22 | 30279157 | 30426857 |
| SF3A1 | chr22 | 30727976 | 30752913 |
| CCDC157 | chr22 | 30752626 | 30772818 |
| SEC14L2 | chr22 | 30792929 | 30821291 |
| MTFP1 | chr22 | 30805175 | 30825041 |
| PES1 | chr22 | 30972611 | 31003000 |
| TCN2 | chr22 | 31003069 | 31023047 |
| SLC35E4 | chr22 | 31031792 | 31065003 |
| MORC2 | chr22 | 31322599 | 31364187 |
| SMTN | chr22 | 31480981 | 31500610 |
| RNF185 | chr22 | 31556137 | 31603005 |
| LIMK2 | chr22 | 31644347 | 31676066 |
| PATZ1 | chr22 | 31721789 | 31742249 |
| DRG1 | chr22 | 31795538 | 31830172 |
| EIF4ENIF1 | chr22 | 31835344 | 31885874 |
| PRR14L | chr22 | 32077333 | 32146120 |
| DEPDC5 | chr22 | 32150008 | 32303020 |
| C22orf24 | chr22 | 32329506 | 32341336 |
| RTCB | chr22 | 32783561 | 32808274 |
| HMGXB4 | chr22 | 35653444 | 35691800 |
| TOM1 | chr22 | 35695796 | 35743987 |
| MCM5 | chr22 | 35796115 | 35820495 |
| RBFOX2 | chr22 | 36134782 | 36236630 |
| APOL2 | chr22 | 36622254 | 36636000 |
| TXN2 | chr22 | 36863092 | 36877687 |
| EIF3D | chr22 | 36906896 | 36925277 |
| MPST | chr22 | 37415682 | 37425863 |
| C1QTNF6 | chr22 | 37576205 | 37581994 |
| RAC2 | chr22 | 37621309 | 37640305 |
| CARD10 | chr22 | 37875382 | 37915378 |
| GGA1 | chr22 | 38005046 | 38029571 |
| PDXP | chr22 | 38054736 | 38062939 |
| LGALS1 | chr22 | 38071612 | 38075809 |
| NOL12 | chr22 | 38082343 | 38089485 |
| H1F0 | chr22 | 38201113 | 38203443 |
| ANKRD54 | chr22 | 38226861 | 38240353 |
| MIR658 | chr22 | 38240278 | 38240378 |
| MIR659 | chr22 | 38243684 | 38243781 |
| EIF3L | chr22 | 38245378 | 38284789 |
| MICALL1 | chr22 | 38302154 | 38338465 |
| PLA2G6 | chr22 | 38507501 | 38577761 |
| LOC400927 | chr22 | 38740669 | 38794931 |
| CBY1 | chr22 | 39052657 | 39069855 |
| SUN2 | chr22 | 39130718 | 39152024 |
| SNORD83B | chr22 | 39709823 | 39709916 |
| SNORD83A | chr22 | 39711217 | 39711312 |
| SMCR7L | chr22 | 39898283 | 39914137 |
| RPS19BP1 | chr22 | 39925097 | 39928860 |
| ADSL | chr22 | 40742503 | 40762575 |
| MKL1 | chr22 | 40806291 | 41032690 |
| SLC25A17 | chr22 | 41165638 | 41215392 |
| XPNPEP3 | chr22 | 41253084 | 41328823 |
| DNAJB7 | chr22 | 41255553 | 41258130 |
| RBX1 | chr22 | 41347350 | 41369019 |
| L3MBTL2 | chr22 | 41601312 | 41627275 |
| PHF5A | chr22 | 41855720 | 41864708 |
| ACO2 | chr22 | 41865128 | 41924993 |
| POLR3H | chr22 | 41921802 | 41940479 |
| XRCC6 | chr22 | 42017353 | 42060052 |
| NHP2L1 | chr22 | 42069936 | 42084913 |
| CENPM | chr22 | 42340180 | 42343148 |
| FAM109B | chr22 | 42470254 | 42475442 |
| SMDT1 | chr22 | 42475694 | 42480288 |
| NDUFA6 | chr22 | 42481529 | 42486888 |
| NDUFA6-AS1 | chr22 | 42486936 | 42521412 |
| RRP7B | chr22 | 42969265 | 42978017 |
| RNU12 | chr22 | 43011250 | 43011399 |
| CYB5R3 | chr22 | 43013845 | 43042995 |
| PACSIN2 | chr22 | 43265771 | 43411184 |
| TSPO | chr22 | 43547940 | 43559248 |
| TTLL12 | chr22 | 43562627 | 43583137 |
| SAMM50 | chr22 | 44370474 | 44392412 |
| PARVB | chr22 | 44464996 | 44565112 |
| LOC100506714 | chr22 | 45529638 | 45559662 |
| NUP50 | chr22 | 45559725 | 45583890 |
| FAM118A | chr22 | 45705784 | 45737836 |
| ATXN10 | chr22 | 46067677 | 46241187 |
| C22orf26 | chr22 | 46446338 | 46450024 |
| LOC150381 | chr22 | 46449725 | 46454402 |
| GTSE1-AS1 | chr22 | 46691039 | 46692557 |
| GTSE1 | chr22 | 46692637 | 46726707 |
| TRMU | chr22 | 46731297 | 46753237 |
| TBC1D22A | chr22 | 47159638 | 47571342 |
| C22orf34 | chr22 | 50013289 | 50051190 |
| LOC90834 | chr22 | 50171537 | 50173958 |
| TRABD | chr22 | 50624359 | 50638027 |
| SELO | chr22 | 50639407 | 50656045 |
| TUBGCP6 | chr22 | 50656117 | 50683400 |
| SBF1 | chr22 | 50883430 | 50913464 |
| LMF2 | chr22 | 50941375 | 50946135 |
| NCAPH2 | chr22 | 50946644 | 50963209 |
| BHLHE40 | chr3 | 5021096 | 5026865 |
| ARL8B | chr3 | 5163929 | 5222601 |
| EDEM1 | chr3 | 5229358 | 5261650 |
| SETD5-AS1 | chr3 | 9430536 | 9439174 |
| SETD5 | chr3 | 9439402 | 9519838 |
| ARPC4-TTLL3 | chr3 | 9834231 | 9878040 |
| ARPC4 | chr3 | 9834231 | 9848789 |
| TTLL3 | chr3 | 9851643 | 9878040 |
| VGLL4 | chr3 | 11597543 | 11762220 |
| MKRN2 | chr3 | 12598512 | 12625210 |
| RAF1 | chr3 | 12625099 | 12705700 |
| RPL32 | chr3 | 12876443 | 12881949 |
| SNORA7A | chr3 | 12881810 | 12881949 |
| ANKRD28 | chr3 | 15708743 | 15839691 |
| RAB5A | chr3 | 19988571 | 20026667 |
| RPL15 | chr3 | 23958294 | 23965187 |
| RARB | chr3 | 25469833 | 25639422 |
| SLC4A7 | chr3 | 27414211 | 27525838 |
| RBMS3 | chr3 | 29322802 | 30051886 |
| TGFBR2 | chr3 | 30647993 | 30735633 |
| UBP1 | chr3 | 33429828 | 33481870 |
| EPM2AIP1 | chr3 | 37027356 | 37034795 |
| CSRNP1 | chr3 | 39183341 | 39195102 |
| RPSA | chr3 | 39448203 | 39454032 |
| CTNNB1 | chr3 | 41240941 | 41281939 |
| SNRK | chr3 | 43328003 | 43392634 |
| LIMD1 | chr3 | 45636322 | 45722755 |
| SACM1L | chr3 | 45730753 | 45786900 |
| SETD2 | chr3 | 47057897 | 47205467 |
| SMARCC1 | chr3 | 47627377 | 47823405 |
| MAP4 | chr3 | 47892179 | 48130769 |
| IP6K2 | chr3 | 48730883 | 48754920 |
| QRICH1 | chr3 | 49067141 | 49130902 |
| RHOA | chr3 | 49396578 | 49449526 |
| RBM5 | chr3 | 50126340 | 50156397 |
| GNAI2 | chr3 | 50263723 | 50296786 |
| HYAL2 | chr3 | 50355220 | 50360213 |
| RASSF1 | chr3 | 50367216 | 50374895 |
| TMEM115 | chr3 | 50392179 | 50396939 |
| MANF | chr3 | 51422691 | 51426828 |
| RBM15B | chr3 | 51428698 | 51435336 |
| WDR82 | chr3 | 52288437 | 52302154 |
| STAB1 | chr3 | 52529355 | 52558511 |
| NT5DC2 | chr3 | 52558384 | 52569093 |
| GNL3 | chr3 | 52719935 | 52728510 |
| TKT | chr3 | 53258722 | 53290130 |
| ARF4 | chr3 | 57557089 | 57583215 |
| FLNB | chr3 | 57994126 | 58157982 |
| RPP14 | chr3 | 58291971 | 58305920 |
| ARL6IP5 | chr3 | 69134089 | 69155239 |
| FOXP1 | chr3 | 71003864 | 71114074 |
| PPP4R2 | chr3 | 73046118 | 73115011 |
| GBE1 | chr3 | 81538849 | 81810950 |
| COL8A1 | chr3 | 99357439 | 99515577 |
| FILIP1L | chr3 | 99566771 | 99595046 |
| TFG | chr3 | 100428174 | 100467811 |
| PCNP | chr3 | 101293041 | 101313281 |
| RPL24 | chr3 | 101399933 | 101405563 |
| NFKBIZ | chr3 | 101568357 | 101579869 |
| ALCAM | chr3 | 105085556 | 105295757 |
| PHLDB2 | chr3 | 111578026 | 111695364 |
| NAA50 | chr3 | 113435306 | 113465120 |
| ARHGAP31 | chr3 | 119013219 | 119138323 |
| GSK3B | chr3 | 119540801 | 119813264 |
| FSTL1 | chr3 | 120113060 | 120169918 |
| KPNA1 | chr3 | 122140747 | 122179504 |
| PDIA5 | chr3 | 122785855 | 122880953 |
| HEG1 | chr3 | 124684553 | 124774802 |
| ZNF148 | chr3 | 124944512 | 125094198 |
| SEC61A1 | chr3 | 127771211 | 127790526 |
| GATA2 | chr3 | 128198264 | 128207373 |
| RPN1 | chr3 | 128338812 | 128369719 |
| RAB7A | chr3 | 128444978 | 128533641 |
| CNBP | chr3 | 128886657 | 128902810 |
| RPL32P3 | chr3 | 129101676 | 129118282 |
| PLXND1 | chr3 | 129274055 | 129325582 |
| CDV3 | chr3 | 133292433 | 133309118 |
| AMOTL2 | chr3 | 134074189 | 134094259 |
| ANAPC13 | chr3 | 134196545 | 134204865 |
| MSL2 | chr3 | 135867759 | 135914688 |
| STAG1 | chr3 | 136055998 | 136471245 |
| RASA2 | chr3 | 141205925 | 141331197 |
| RNF7 | chr3 | 141457050 | 141465645 |
| ATP1B3 | chr3 | 141595469 | 141645382 |
| U2SURP | chr3 | 142720371 | 142779567 |
| C3orf58 | chr3 | 143690639 | 143711210 |
| PLOD2 | chr3 | 145787227 | 145879282 |
| TM4SF1 | chr3 | 149086804 | 149095568 |
| WWTR1 | chr3 | 149235021 | 149375888 |
| PFN2 | chr3 | 149682690 | 149688741 |
| TSC22D2 | chr3 | 150126787 | 150177615 |
| SERP1 | chr3 | 150259779 | 150264964 |
| MBNL1 | chr3 | 151985828 | 152183569 |
| RAP2B | chr3 | 152880028 | 152886263 |
| SLC33A1 | chr3 | 155544300 | 155572248 |
| SSR3 | chr3 | 156257928 | 156272973 |
| TIPARP | chr3 | 156392714 | 156424557 |
| CCNL1 | chr3 | 156865585 | 156878482 |
| PTX3 | chr3 | 157154579 | 157161417 |
| SMC4 | chr3 | 160118346 | 160152741 |
| TRIM59 | chr3 | 160153290 | 160167626 |
| KPNA4 | chr3 | 160212782 | 160283376 |
| NMD3 | chr3 | 160939098 | 160969795 |
| MECOM | chr3 | 168801286 | 168865522 |
| SKIL | chr3 | 170075472 | 170114637 |
| CLDN11 | chr3 | 170136652 | 170152479 |
| FNDC3B | chr3 | 171757417 | 172118492 |
| ECT2 | chr3 | 172468474 | 172539264 |
| TBL1XR1 | chr3 | 176738541 | 176915048 |
| GNB4 | chr3 | 179113875 | 179169371 |
| FXR1 | chr3 | 180630233 | 180700539 |
| YEATS2 | chr3 | 183415605 | 183530413 |
| DVL3 | chr3 | 183873283 | 183891314 |
| AP2M1 | chr3 | 183892633 | 183901879 |
| PSMD2 | chr3 | 184017021 | 184026840 |
| EIF4G1 | chr3 | 184032951 | 184053146 |
| IGF2BP2 | chr3 | 185361526 | 185542827 |
| TRA2B | chr3 | 185632357 | 185655924 |
| EIF4A2 | chr3 | 186501360 | 186507685 |
| SNORD2 | chr3 | 186502584 | 186502654 |
| CCDC50 | chr3 | 191046873 | 191116459 |
| FAM43A | chr3 | 194406621 | 194409766 |
| SDHAP2 | chr3 | 195384909 | 195415735 |
| SDHAP1 | chr3 | 195686791 | 195717150 |
| RNF168 | chr3 | 196195656 | 196230639 |
| PAK2 | chr3 | 196466727 | 196559518 |
| LOC220729 | chr3 | 197340897 | 197354752 |
| LRCH3 | chr3 | 197518144 | 197611043 |
| RPL35A | chr3 | 197677051 | 197682721 |
| TRNT1 | chr3 | 3168599 | 3190706 |
| CRBN | chr3 | 3191316 | 3221401 |
| RAD18 | chr3 | 8918879 | 9005159 |
| THUMPD3 | chr3 | 9404716 | 9428475 |
| BRPF1 | chr3 | 9773433 | 9789699 |
| TADA3 | chr3 | 9824811 | 9834695 |
| JAGN1 | chr3 | 9932270 | 9936031 |
| EMC3 | chr3 | 10010344 | 10028522 |
| CIDECP | chr3 | 10059236 | 10067820 |
| FANCD2 | chr3 | 10068112 | 10141344 |
| BRK1 | chr3 | 10157332 | 10168874 |
| VHL | chr3 | 10183318 | 10195354 |
| TATDN2 | chr3 | 10290176 | 10322906 |
| SEC13 | chr3 | 10342614 | 10355656 |
| HRH1 | chr3 | 11267668 | 11304939 |
| ATG7 | chr3 | 11314009 | 11599139 |
| VGLL4 | chr3 | 11597543 | 11623836 |
| TAMM41 | chr3 | 11848767 | 11888352 |
| CHCHD4 | chr3 | 14153576 | 14166371 |
| TMEM43 | chr3 | 14166439 | 14185180 |
| XPC | chr3 | 14186647 | 14220172 |
| LSM3 | chr3 | 14220227 | 14239869 |
| SLC6A6 | chr3 | 14444075 | 14530857 |
| CCDC174 | chr3 | 14693252 | 14714166 |
| FGD5 | chr3 | 14860468 | 14976072 |
| FGD5-AS1 | chr3 | 14984285 | 14989948 |
| NR2C2 | chr3 | 14989235 | 15090780 |
| MRPS25 | chr3 | 15090018 | 15106816 |
| ZFYVE20 | chr3 | 15111579 | 15140655 |
| CAPN7 | chr3 | 15247732 | 15294423 |
| SH3BP5 | chr3 | 15295862 | 15374136 |
| METTL6 | chr3 | 15451376 | 15469042 |
| EAF1 | chr3 | 15469063 | 15484120 |
| HACL1 | chr3 | 15602239 | 15643130 |
| BTD | chr3 | 15643254 | 15687325 |
| DPH3 | chr3 | 16298567 | 16306496 |
| OXNAD1 | chr3 | 16306666 | 16347594 |
| RFTN1 | chr3 | 16357351 | 16555222 |
| TBC1D5 | chr3 | 17198653 | 17782399 |
| SGOL1 | chr3 | 20209935 | 20227724 |
| UBE2E1 | chr3 | 23851933 | 23933131 |
| NKIRAS1 | chr3 | 23933571 | 23958537 |
| RPL15 | chr3 | 23959195 | 23962347 |
| NR1D2 | chr3 | 23987611 | 24022109 |
| TOP2B | chr3 | 25639395 | 25705863 |
| MIR4442 | chr3 | 25706366 | 25706430 |
| SLC4A7 | chr3 | 27414211 | 27525911 |
| AZI2 | chr3 | 28363843 | 28390618 |
| STT3B | chr3 | 31574490 | 31677556 |
| CMTM7 | chr3 | 32433162 | 32496333 |
| CMTM6 | chr3 | 32522803 | 32544403 |
| DYNC1LI1 | chr3 | 32567462 | 32612366 |
| CNOT10 | chr3 | 32726636 | 32815367 |
| GLB1 | chr3 | 33038099 | 33138694 |
| TMPPE | chr3 | 33131907 | 33138314 |
| CLASP2 | chr3 | 33537738 | 33759705 |
| PDCD6IP | chr3 | 33840062 | 33911199 |
| MLH1 | chr3 | 37034840 | 37092337 |
| LRRFIP2 | chr3 | 37094116 | 37217851 |
| GOLGA4 | chr3 | 37284681 | 37408370 |
| OXSR1 | chr3 | 38207025 | 38296979 |
| GORASP1 | chr3 | 39138090 | 39149130 |
| TTC21A | chr3 | 39149151 | 39180394 |
| SLC25A38 | chr3 | 39424814 | 39438819 |
| RPSA | chr3 | 39449110 | 39454032 |
| SNORA6 | chr3 | 39449881 | 39450030 |
| SNORA62 | chr3 | 39452544 | 39452698 |
| EIF1B-AS1 | chr3 | 40214637 | 40351189 |
| EIF1B | chr3 | 40351172 | 40353915 |
| RPL14 | chr3 | 40498829 | 40503863 |
| ZNF620 | chr3 | 40547529 | 40559712 |
| ULK4 | chr3 | 41288089 | 42003660 |
| TRAK1 | chr3 | 42201660 | 42254118 |
| SEC22C | chr3 | 42589458 | 42623520 |
| SS18L2 | chr3 | 42632297 | 42636490 |
| NKTR | chr3 | 42642146 | 42690233 |
| ZBTB47 | chr3 | 42695175 | 42709072 |
| HIGD1A | chr3 | 42824399 | 42845934 |
| TCAIM | chr3 | 44379943 | 44450940 |
| KIAA1143 | chr3 | 44790235 | 44803173 |
| KIF15 | chr3 | 44803208 | 44894748 |
| ZDHHC3 | chr3 | 44956752 | 45017674 |
| EXOSC7 | chr3 | 45016749 | 45054158 |
| LIMD1-AS1 | chr3 | 45719656 | 45730374 |
| CCRL2 | chr3 | 46448720 | 46451014 |
| CCDC12 | chr3 | 46963219 | 47023500 |
| NBEAL2 | chr3 | 47021172 | 47051194 |
| KIF9-AS1 | chr3 | 47205859 | 47285606 |
| KIF9 | chr3 | 47269515 | 47324337 |
| KLHL18 | chr3 | 47324329 | 47388306 |
| PTPN23 | chr3 | 47422490 | 47454931 |
| ELP6 | chr3 | 47537129 | 47555199 |
| DHX30 | chr3 | 47844398 | 47891686 |
| CDC25A | chr3 | 48198667 | 48229801 |
| NME6 | chr3 | 48335588 | 48342848 |
| CCDC51 | chr3 | 48473579 | 48481529 |
| TMA7 | chr3 | 48481685 | 48485537 |
| ATRIP | chr3 | 48488496 | 48507708 |
| TREX1 | chr3 | 48501185 | 48509044 |
| SHISA5 | chr3 | 48509196 | 48514742 |
| ARIH2OS | chr3 | 48955220 | 48956818 |
| ARIH2 | chr3 | 48956280 | 49022971 |
| P4HTM | chr3 | 49027340 | 49044581 |
| WDR6 | chr3 | 49044636 | 49053386 |
| DALRD3 | chr3 | 49052920 | 49058504 |
| MIR425 | chr3 | 49057580 | 49057667 |
| MIR191 | chr3 | 49058050 | 49058142 |
| NDUFAF3 | chr3 | 49059073 | 49060926 |
| IMPDH2 | chr3 | 49061761 | 49066875 |
| QARS | chr3 | 49133364 | 49142562 |
| LAMB2 | chr3 | 49158546 | 49170599 |
| USP4 | chr3 | 49314576 | 49350296 |
| GPX1 | chr3 | 49394608 | 49395791 |
| TCTA | chr3 | 49449638 | 49453909 |
| DAG1 | chr3 | 49507564 | 49573051 |
| RNF123 | chr3 | 49726949 | 49758962 |
| GMPPB | chr3 | 49758931 | 49761384 |
| IP6K1 | chr3 | 49761727 | 49823627 |
| MIR5193 | chr3 | 49843569 | 49843678 |
| RBM6 | chr3 | 49977476 | 50114685 |
| SEMA3F | chr3 | 50196451 | 50226508 |
| IFRD2 | chr3 | 50325162 | 50330349 |
| TUSC2 | chr3 | 50362340 | 50365668 |
| VPRBP | chr3 | 51433297 | 51534018 |
| RAD54L2 | chr3 | 51575595 | 51697612 |
| RRP9 | chr3 | 51967441 | 51975957 |
| RPL29 | chr3 | 52027643 | 52029958 |
| DUSP7 | chr3 | 52085647 | 52090461 |
| ALAS1 | chr3 | 52232098 | 52248343 |
| TWF2 | chr3 | 52262625 | 52273183 |
| MIRLET7G | chr3 | 52302293 | 52302377 |
| GLYCTK | chr3 | 52321835 | 52329272 |
| BAP1 | chr3 | 52435019 | 52444121 |
| PHF7 | chr3 | 52444526 | 52457657 |
| NISCH | chr3 | 52489523 | 52527088 |
| SMIM4 | chr3 | 52570620 | 52574586 |
| PBRM1 | chr3 | 52579367 | 52713739 |
| SNORD19 | chr3 | 52723255 | 52723331 |
| SNORD19B | chr3 | 52724753 | 52724846 |
| GLT8D1 | chr3 | 52728503 | 52740048 |
| SPCS1 | chr3 | 52739856 | 52742197 |
| RFT1 | chr3 | 53122500 | 53164470 |
| DCP1A | chr3 | 53317444 | 53381654 |
| ACTR8 | chr3 | 53901093 | 53916229 |
| SELK | chr3 | 53919225 | 53925989 |
| CCDC66 | chr3 | 56591183 | 56655848 |
| FAM208A | chr3 | 56654159 | 56717135 |
| APPL1 | chr3 | 57261764 | 57307498 |
| PDE12 | chr3 | 57541980 | 57547768 |
| DENND6A | chr3 | 57611180 | 57678816 |
| SLMAP | chr3 | 57743173 | 57914894 |
| KCTD6 | chr3 | 58477822 | 58488087 |
| PTPRG | chr3 | 61547242 | 62280573 |
| THOC7 | chr3 | 63819545 | 63849597 |
| ATXN7 | chr3 | 63850232 | 63989136 |
| PSMD6 | chr3 | 63996224 | 64009113 |
| PRICKLE2-AS3 | chr3 | 64173219 | 64187236 |
| ADAMTS9 | chr3 | 64501330 | 64673365 |
| ADAMTS9-AS2 | chr3 | 64670545 | 64997143 |
| MAGI1 | chr3 | 65339905 | 66024509 |
| LRIG1 | chr3 | 66429220 | 66550845 |
| EOGT | chr3 | 69024367 | 69062774 |
| TMF1 | chr3 | 69068977 | 69101484 |
| MIR3136 | chr3 | 69098108 | 69098186 |
| UBA3 | chr3 | 69103880 | 69129524 |
| FOXP1 | chr3 | 71003864 | 71632904 |
| RYBP | chr3 | 72423743 | 72495774 |
| CGGBP1 | chr3 | 88101099 | 88108146 |
| C3orf38 | chr3 | 88198892 | 88207115 |
| DHFRL1 | chr3 | 93776765 | 93782067 |
| NSUN3 | chr3 | 93781854 | 93845630 |
| MINA | chr3 | 97660660 | 97691295 |
| CLDND1 | chr3 | 98234316 | 98241358 |
| ST3GAL6-AS1 | chr3 | 98433176 | 98451495 |
| ST3GAL6 | chr3 | 98451547 | 98514689 |
| DCBLD2 | chr3 | 98514813 | 98620533 |
| CMSS1 | chr3 | 99536677 | 99897476 |
| TBC1D23 | chr3 | 99979660 | 100044096 |
| TOMM70A | chr3 | 100082302 | 100120242 |
| LNP1 | chr3 | 100120036 | 100175170 |
| SENP7 | chr3 | 101043117 | 101232085 |
| TRMT10C | chr3 | 101280679 | 101285290 |
| ZBTB11 | chr3 | 101368282 | 101395988 |
| ZBTB11-AS1 | chr3 | 101395273 | 101398057 |
| CEP97 | chr3 | 101443493 | 101486181 |
| LOC152225 | chr3 | 101659702 | 101716770 |
| CBLB | chr3 | 105377108 | 105587887 |
| KIAA1524 | chr3 | 108268717 | 108308491 |
| DZIP3 | chr3 | 108308336 | 108413693 |
| PVRL3 | chr3 | 110790605 | 110856407 |
| ABHD10 | chr3 | 111697722 | 111712215 |
| ATG3 | chr3 | 112251356 | 112280485 |
| SLC35A5 | chr3 | 112280856 | 112303284 |
| CCDC80 | chr3 | 112323408 | 112359977 |
| GTPBP8 | chr3 | 112709799 | 112720221 |
| C3orf17 | chr3 | 112721291 | 112738555 |
| ATP6V1A | chr3 | 113465865 | 113530905 |
| KIAA1407 | chr3 | 113682983 | 113775460 |
| QTRTD1 | chr3 | 113775581 | 113807268 |
| ZBTB20 | chr3 | 114056946 | 114343053 |
| TMEM39A | chr3 | 119147806 | 119182529 |
| TIMMDC1 | chr3 | 119217367 | 119243125 |
| LRRC58 | chr3 | 120043575 | 120068186 |
| NDUFB4 | chr3 | 120315127 | 120321258 |
| RABL3 | chr3 | 120405527 | 120461384 |
| GTF2E1 | chr3 | 120461557 | 120501916 |
| POLQ | chr3 | 121150272 | 121264853 |
| HCLS1 | chr3 | 121350245 | 121379791 |
| GOLGB1 | chr3 | 121382045 | 121468614 |
| CCDC58 | chr3 | 122078435 | 122102074 |
| WDR5B | chr3 | 122130699 | 122134882 |
| HSPBAP1 | chr3 | 122458843 | 122512666 |
| DIRC2 | chr3 | 122513900 | 122599986 |
| PTPLB | chr3 | 123213362 | 123303924 |
| MYLK-AS1 | chr3 | 123304402 | 123349666 |
| CCDC14 | chr3 | 123632273 | 123680255 |
| UMPS | chr3 | 124449212 | 124468119 |
| SNX4 | chr3 | 125165487 | 125239058 |
| OSBPL11 | chr3 | 125247701 | 125314381 |
| ZXDC | chr3 | 126169958 | 126194762 |
| TPRA1 | chr3 | 127291906 | 127309602 |
| MCM2 | chr3 | 127317199 | 127341278 |
| MGLL | chr3 | 127407904 | 127541725 |
| RUVBL1 | chr3 | 127783627 | 127872757 |
| ACAD9 | chr3 | 128598332 | 128631957 |
| ISY1-RAB43 | chr3 | 128806411 | 128880073 |
| RAB43 | chr3 | 128806411 | 128840647 |
| ISY1 | chr3 | 128846258 | 128880073 |
| COPG1 | chr3 | 128968452 | 128996616 |
| C3orf37 | chr3 | 128997816 | 129024135 |
| SNORA7B | chr3 | 129116052 | 129116191 |
| MBD4 | chr3 | 129149786 | 129159022 |
| IFT122 | chr3 | 129158967 | 129239191 |
| TMCC1 | chr3 | 129366634 | 129407575 |
| PIK3R4 | chr3 | 130397777 | 130465696 |
| ATP2C1 | chr3 | 130612834 | 130722046 |
| LOC339874 | chr3 | 131043935 | 131100319 |
| NUDT16 | chr3 | 131100624 | 131107674 |
| MRPL3 | chr3 | 131181044 | 131221860 |
| DNAJC13 | chr3 | 132136552 | 132257876 |
| ACAD11 | chr3 | 132276981 | 132378975 |
| UBA5 | chr3 | 132379139 | 132396944 |
| TOPBP1 | chr3 | 133319448 | 133380737 |
| RYK | chr3 | 133875977 | 133969586 |
| CEP63 | chr3 | 134205046 | 134283870 |
| SLC35G2 | chr3 | 136537860 | 136574734 |
| NCK1 | chr3 | 136581049 | 136667968 |
| ARMC8 | chr3 | 137906114 | 138017228 |
| MRPS22 | chr3 | 139062797 | 139075887 |
| COPB2 | chr3 | 139076432 | 139108522 |
| SLC25A36 | chr3 | 140660661 | 140698785 |
| XRN1 | chr3 | 142025448 | 142166853 |
| ATR | chr3 | 142168076 | 142297668 |
| LOC100289361 | chr3 | 142719686 | 142720309 |
| PLSCR4 | chr3 | 145910123 | 145968966 |
| PLSCR1 | chr3 | 146232966 | 146262628 |
| TM4SF18 | chr3 | 149036284 | 149051548 |
| WWTR1-AS1 | chr3 | 149375996 | 149379149 |
| RNF13 | chr3 | 149530474 | 149679925 |
| LOC646903 | chr3 | 149689065 | 149691029 |
| EIF2A | chr3 | 150264573 | 150303803 |
| SELT | chr3 | 150321065 | 150348234 |
| SIAH2 | chr3 | 150458909 | 150481263 |
| LOC201651 | chr3 | 151488243 | 151502682 |
| MBNL1-AS1 | chr3 | 151980404 | 151987415 |
| TMEM14E | chr3 | 152057486 | 152058779 |
| DHX36 | chr3 | 153993456 | 154042286 |
| GMPS | chr3 | 155588324 | 155655520 |
| TIPARP-AS1 | chr3 | 156390959 | 156393502 |
| LINC00886 | chr3 | 156465131 | 156534851 |
| LEKR1 | chr3 | 156544095 | 156763918 |
| VEPH1 | chr3 | 156977531 | 157217445 |
| GFM1 | chr3 | 158362316 | 158410360 |
| LXN | chr3 | 158384202 | 158390482 |
| SCHIP1 | chr3 | 159557649 | 159615155 |
| IFT80 | chr3 | 159974773 | 160117320 |
| MIR15B | chr3 | 160122375 | 160122473 |
| MIR16-2 | chr3 | 160122532 | 160122613 |
| PDCD10 | chr3 | 167401694 | 167452594 |
| GOLIM4 | chr3 | 167727653 | 167813417 |
| MYNN | chr3 | 169490852 | 169507504 |
| LOC100128164 | chr3 | 169661771 | 169684522 |
| SEC62 | chr3 | 169684579 | 169716161 |
| PHC3 | chr3 | 169805367 | 169899537 |
| PRKCI | chr3 | 169940219 | 170023770 |
| RPL22L1 | chr3 | 170582664 | 170588045 |
| TNIK | chr3 | 170780291 | 171178197 |
| PLD1 | chr3 | 171318194 | 171528504 |
| NCEH1 | chr3 | 172348434 | 172429008 |
| NLGN1 | chr3 | 173302476 | 174001116 |
| ZMAT3 | chr3 | 178735010 | 178789656 |
| PIK3CA | chr3 | 178866310 | 178952497 |
| ZNF639 | chr3 | 179041550 | 179053320 |
| ACTL6A | chr3 | 179280707 | 179306193 |
| MRPL47 | chr3 | 179306254 | 179322434 |
| NDUFB5 | chr3 | 179322574 | 179342288 |
| USP13 | chr3 | 179370932 | 179507189 |
| ATP11B | chr3 | 182511290 | 182639421 |
| DCUN1D1 | chr3 | 182660558 | 182698326 |
| B3GNT5 | chr3 | 182971605 | 182991179 |
| KLHL6 | chr3 | 183205318 | 183273499 |
| PARL | chr3 | 183547172 | 183602693 |
| EIF2B5 | chr3 | 183852809 | 183863099 |
| ABCF3 | chr3 | 183903862 | 183911795 |
| ALG3 | chr3 | 183960116 | 183967313 |
| EIF4G1 | chr3 | 184038100 | 184053146 |
| SNORD66 | chr3 | 184043483 | 184043559 |
| FAM131A | chr3 | 184055275 | 184064063 |
| POLR2H | chr3 | 184081193 | 184086363 |
| SENP2 | chr3 | 185304030 | 185348885 |
| C3orf65 | chr3 | 185431039 | 185435955 |
| ETV5 | chr3 | 185764105 | 185826901 |
| TBCCD1 | chr3 | 186263855 | 186288332 |
| DNAJB11 | chr3 | 186287951 | 186303589 |
| SNORA81 | chr3 | 186504460 | 186504641 |
| MIR1248 | chr3 | 186504460 | 186504566 |
| SNORA63 | chr3 | 186505087 | 186505222 |
| SNORA4 | chr3 | 186505401 | 186505538 |
| ST6GAL1 | chr3 | 186648314 | 186796341 |
| BCL6 | chr3 | 187439164 | 187454285 |
| LPP-AS2 | chr3 | 187868993 | 187871876 |
| LPP | chr3 | 187871662 | 188608460 |
| FLJ42393 | chr3 | 187896330 | 187898596 |
| UTS2B | chr3 | 190984943 | 191048325 |
| MB21D2 | chr3 | 192514604 | 192635950 |
| OPA1 | chr3 | 193310932 | 193415600 |
| LOC647323 | chr3 | 193675160 | 193721448 |
| HES1 | chr3 | 193853930 | 193856401 |
| TMEM44-AS1 | chr3 | 194304996 | 194311561 |
| TMEM44 | chr3 | 194308401 | 194354150 |
| LSG1 | chr3 | 194361516 | 194393206 |
| XXYLT1 | chr3 | 194789012 | 194991895 |
| ACAP2 | chr3 | 194995464 | 195163817 |
| PPP1R2 | chr3 | 195241220 | 195270224 |
| TNK2 | chr3 | 195590235 | 195622432 |
| TFRC | chr3 | 195776154 | 195808961 |
| PCYT1A | chr3 | 195965252 | 196014584 |
| UBXN7 | chr3 | 196080368 | 196159345 |
| WDR53 | chr3 | 196281058 | 196295413 |
| FBXO45 | chr3 | 196295724 | 196315930 |
| SENP5 | chr3 | 196594726 | 196661584 |
| NCBP2 | chr3 | 196662272 | 196669276 |
| NCBP2-AS2 | chr3 | 196669493 | 196670884 |
| DLG1 | chr3 | 196769430 | 197026143 |
| MIR4797 | chr3 | 197020748 | 197020819 |
| FYTTD1 | chr3 | 197477260 | 197511317 |
| IQCG | chr3 | 197615945 | 197676796 |
| PCGF3 | chr4 | 727672 | 764427 |
| LOC100129917 | chr4 | 773936 | 775636 |
| CTBP1 | chr4 | 1205227 | 1242908 |
| MAEA | chr4 | 1303598 | 1333925 |
| SLBP | chr4 | 1694526 | 1714030 |
| TACC3 | chr4 | 1723216 | 1746905 |
| WHSC1 | chr4 | 1873122 | 1983934 |
| RNF4 | chr4 | 2471179 | 2517586 |
| MRFAP1 | chr4 | 6641817 | 6644470 |
| AFAP1 | chr4 | 7760439 | 7941653 |
| SH3TC1 | chr4 | 8201059 | 8242830 |
| WDR1 | chr4 | 10075962 | 10118573 |
| MED28 | chr4 | 17616272 | 17626160 |
| DCAF16 | chr4 | 17802277 | 17812381 |
| NCAPG | chr4 | 17812435 | 17846487 |
| DHX15 | chr4 | 24529087 | 24586184 |
| KLF3 | chr4 | 38665789 | 38703129 |
| RPL9 | chr4 | 39457430 | 39460568 |
| UBE2K | chr4 | 39699663 | 39784410 |
| PDS5A | chr4 | 39824482 | 39979576 |
| APBB2 | chr4 | 40812043 | 41216635 |
| FRYL | chr4 | 48499379 | 48782316 |
| CHIC2 | chr4 | 54875957 | 54930815 |
| KDR | chr4 | 55944425 | 55991762 |
| TMEM165 | chr4 | 56262079 | 56292342 |
| POLR2B | chr4 | 57845108 | 57897334 |
| IGFBP7 | chr4 | 57897236 | 57976551 |
| YTHDC1 | chr4 | 69176104 | 69215824 |
| GRSF1 | chr4 | 71681498 | 71705627 |
| ANKRD17 | chr4 | 73940501 | 74124502 |
| RCHY1 | chr4 | 76404246 | 76439640 |
| G3BP2 | chr4 | 76567952 | 76598667 |
| SDAD1 | chr4 | 76871067 | 76912113 |
| 11-Sep | chr4 | 77870894 | 77959768 |
| CCNI | chr4 | 77969176 | 77997125 |
| CNOT6L | chr4 | 78634540 | 78740522 |
| HNRNPD | chr4 | 83274466 | 83295149 |
| HNRPDL | chr4 | 83343716 | 83351378 |
| HNRNPDL | chr4 | 83343716 | 83351378 |
| SEC31A | chr4 | 83739813 | 83822069 |
| THAP9-AS1 | chr4 | 83814604 | 83822069 |
| ARHGAP24 | chr4 | 86699850 | 86923823 |
| AFF1 | chr4 | 87928152 | 88062206 |
| MMRN1 | chr4 | 90816051 | 90875780 |
| PDLIM5 | chr4 | 95373007 | 95589378 |
| TSPAN5 | chr4 | 99391517 | 99579812 |
| EIF4E | chr4 | 99799606 | 99851786 |
| H2AFZ | chr4 | 100869243 | 100871512 |
| UBE2D3 | chr4 | 103717132 | 103748340 |
| AIMP1 | chr4 | 107236852 | 107270381 |
| RPL34 | chr4 | 109541748 | 109551639 |
| OSTC | chr4 | 109571740 | 109588978 |
| SEC24B | chr4 | 110354970 | 110461615 |
| ARSJ | chr4 | 114821439 | 114900878 |
| SEC24D | chr4 | 119643977 | 119757326 |
| ANXA5 | chr4 | 122589151 | 122618147 |
| CCNA2 | chr4 | 122737598 | 122745088 |
| ANKRD50 | chr4 | 125585203 | 125633887 |
| SLC7A11 | chr4 | 139085247 | 139163503 |
| CCRN4L | chr4 | 139936942 | 139967095 |
| NAA15 | chr4 | 140222675 | 140311935 |
| RAB33B | chr4 | 140374960 | 140397069 |
| USP38 | chr4 | 144106069 | 144143141 |
| SMARCA5 | chr4 | 144434615 | 144478642 |
| HHIP-AS1 | chr4 | 145564067 | 145567171 |
| HHIP | chr4 | 145567147 | 145659881 |
| ABCE1 | chr4 | 146019155 | 146050676 |
| OTUD4 | chr4 | 146054801 | 146100832 |
| SMAD1 | chr4 | 146403962 | 146480325 |
| ARHGAP10 | chr4 | 148653452 | 148993927 |
| RPS3A | chr4 | 152020724 | 152025357 |
| FAM198B | chr4 | 159045731 | 159093718 |
| MSMO1 | chr4 | 166248817 | 166264314 |
| SH3RF1 | chr4 | 170015406 | 170192249 |
| HMGB2 | chr4 | 174252526 | 174255595 |
| SPCS3 | chr4 | 177241089 | 177253396 |
| VEGFC | chr4 | 177604690 | 177713895 |
| CASP3 | chr4 | 185548849 | 185570629 |
| ZNF595 | chr4 | 53226 | 88099 |
| ZNF718 | chr4 | 53226 | 156490 |
| ABCA11P | chr4 | 419223 | 467998 |
| ZNF721 | chr4 | 433772 | 467998 |
| PIGG | chr4 | 492988 | 533320 |
| GAK | chr4 | 843064 | 926174 |
| TMEM175 | chr4 | 926261 | 952443 |
| CTBP1-AS1 | chr4 | 1243227 | 1246795 |
| TMEM129 | chr4 | 1717678 | 1723084 |
| LETM1 | chr4 | 1813205 | 1857974 |
| WHSC1 | chr4 | 1900947 | 1950545 |
| NELFA | chr4 | 1984440 | 1993971 |
| HAUS3 | chr4 | 2230095 | 2243860 |
| TNIP2 | chr4 | 2743386 | 2758103 |
| SH3BP2 | chr4 | 2828051 | 2842823 |
| ADD1 | chr4 | 2845877 | 2931802 |
| MFSD10 | chr4 | 2932287 | 2935964 |
| NOP14 | chr4 | 2939663 | 2965118 |
| GRK4 | chr4 | 2965342 | 3042474 |
| HTT | chr4 | 3076407 | 3245687 |
| RGS12 | chr4 | 3315873 | 3433904 |
| LRPAP1 | chr4 | 3505323 | 3534224 |
| TMEM128 | chr4 | 4237272 | 4249959 |
| LYAR | chr4 | 4269428 | 4291896 |
| ZBTB49 | chr4 | 4291923 | 4323513 |
| STX18 | chr4 | 4420695 | 4543775 |
| LOC100507266 | chr4 | 4543857 | 4712664 |
| LOC93622 | chr4 | 6675820 | 6677774 |
| MRFAP1L1 | chr4 | 6709428 | 6711606 |
| KIAA0232 | chr4 | 6784458 | 6885899 |
| TBC1D14 | chr4 | 6988888 | 7034845 |
| LOC100129931 | chr4 | 7032280 | 7047958 |
| TADA2B | chr4 | 7045155 | 7059677 |
| GRPEL1 | chr4 | 7062255 | 7069800 |
| ACOX3 | chr4 | 8368008 | 8430208 |
| TRMT44 | chr4 | 8442531 | 8481260 |
| ZNF518B | chr4 | 10441503 | 10459032 |
| RAB28 | chr4 | 13369346 | 13485989 |
| BOD1L1 | chr4 | 13570365 | 13629328 |
| MIR5091 | chr4 | 13629488 | 13629581 |
| CPEB2 | chr4 | 15004297 | 15071777 |
| FBXL5 | chr4 | 15606006 | 15657035 |
| FAM200B | chr4 | 15683351 | 15692070 |
| LDB2 | chr4 | 16503168 | 16900424 |
| LCORL | chr4 | 17882217 | 18023483 |
| SLIT2 | chr4 | 20255234 | 20620788 |
| PACRGL | chr4 | 20702035 | 20729980 |
| CCDC149 | chr4 | 24828580 | 24981826 |
| SEPSECS | chr4 | 25121626 | 25162204 |
| LOC285540 | chr4 | 25162293 | 25200127 |
| PI4K2B | chr4 | 25235652 | 25280831 |
| SEL1L3 | chr4 | 25749048 | 25864610 |
| RBPJ | chr4 | 26322428 | 26436752 |
| PCDH7 | chr4 | 30722036 | 31148423 |
| RELL1 | chr4 | 37612255 | 37687999 |
| PGM2 | chr4 | 37828281 | 37864559 |
| FLJ13197 | chr4 | 38614321 | 38666249 |
| FAM114A1 | chr4 | 38869353 | 38947365 |
| MIR574 | chr4 | 38869652 | 38869748 |
| KLHL5 | chr4 | 39046450 | 39127853 |
| RFC1 | chr4 | 39289068 | 39368001 |
| LIAS | chr4 | 39460664 | 39479271 |
| UGDH | chr4 | 39500374 | 39529218 |
| UGDH-AS1 | chr4 | 39529458 | 39596327 |
| SMIM14 | chr4 | 39552545 | 39640481 |
| LOC344967 | chr4 | 40044536 | 40058819 |
| N4BP2 | chr4 | 40058523 | 40159872 |
| TMEM33 | chr4 | 41937136 | 41962824 |
| SLC30A9 | chr4 | 41992522 | 42089551 |
| GUF1 | chr4 | 44680432 | 44702697 |
| ATP10D | chr4 | 47487409 | 47595503 |
| NFXL1 | chr4 | 47849257 | 47916680 |
| SLAIN2 | chr4 | 48343612 | 48428215 |
| OCIAD1 | chr4 | 48833243 | 48863834 |
| OCIAD2 | chr4 | 48887404 | 48908815 |
| SGCB | chr4 | 52886860 | 52904485 |
| DANCR | chr4 | 53578620 | 53580305 |
| MIR4449 | chr4 | 53578848 | 53578914 |
| SNORA26 | chr4 | 53579415 | 53579537 |
| FIP1L1 | chr4 | 54243819 | 54326103 |
| LNX1 | chr4 | 54326436 | 54424436 |
| CLOCK | chr4 | 56294067 | 56412099 |
| EXOC1 | chr4 | 56719815 | 56771244 |
| CEP135 | chr4 | 56814973 | 56899529 |
| AASDH | chr4 | 57204456 | 57253638 |
| PPAT | chr4 | 57259528 | 57301802 |
| PAICS | chr4 | 57301917 | 57327534 |
| SRP72 | chr4 | 57333761 | 57369847 |
| REST | chr4 | 57774041 | 57802010 |
| NOA1 | chr4 | 57829515 | 57843826 |
| LOC255130 | chr4 | 57975927 | 58071465 |
| CENPC1 | chr4 | 68337988 | 68411256 |
| UBA6 | chr4 | 68481478 | 68566889 |
| LOC550112 | chr4 | 68566995 | 68588222 |
| SULT1B1 | chr4 | 70592685 | 70626430 |
| UTP3 | chr4 | 71554195 | 71556268 |
| RUFY3 | chr4 | 71570653 | 71655614 |
| MOB1B | chr4 | 71768056 | 71853891 |
| DCK | chr4 | 71859264 | 71896629 |
| COX18 | chr4 | 73920415 | 73935472 |
| CXCL2 | chr4 | 74962753 | 74964997 |
| MTHFD2L | chr4 | 75023828 | 75168814 |
| THAP6 | chr4 | 76439729 | 76455236 |
| USO1 | chr4 | 76649828 | 76735366 |
| NUP54 | chr4 | 77035816 | 77069655 |
| SCARB2 | chr4 | 77079891 | 77135052 |
| MRPL1 | chr4 | 78783804 | 78873944 |
| ANXA3 | chr4 | 79472741 | 79531605 |
| LOC100505702 | chr4 | 79567147 | 79605655 |
| BMP2K | chr4 | 79697531 | 79833341 |
| ANTXR2 | chr4 | 80822770 | 80994477 |
| ENOPH1 | chr4 | 83351725 | 83382244 |
| THAP9 | chr4 | 83821836 | 83841284 |
| LIN54 | chr4 | 83845755 | 83931982 |
| COPS4 | chr4 | 83956238 | 83996971 |
| COQ2 | chr4 | 84184976 | 84206067 |
| HELQ | chr4 | 84328498 | 84377025 |
| MRPS18C | chr4 | 84377117 | 84382929 |
| WDFY3 | chr4 | 85590692 | 85887544 |
| WDFY3-AS2 | chr4 | 85887970 | 85928168 |
| ARHGAP24 | chr4 | 86851425 | 86923823 |
| LOC100506746 | chr4 | 87846045 | 87856002 |
| AFF1 | chr4 | 87856153 | 88062206 |
| NUDT9 | chr4 | 88343727 | 88380606 |
| PKD2 | chr4 | 88928798 | 88998931 |
| PYURF | chr4 | 89442128 | 89444952 |
| PIGY | chr4 | 89442724 | 89442940 |
| FAM13A | chr4 | 89647104 | 89978346 |
| SNCA | chr4 | 90645249 | 90758350 |
| LOC644248 | chr4 | 90757551 | 90763142 |
| SMARCAD1 | chr4 | 95129234 | 95212443 |
| RAP1GDS1 | chr4 | 99182526 | 99365012 |
| METAP1 | chr4 | 99916787 | 99983960 |
| MIR3684 | chr4 | 99918537 | 99918611 |
| ADH5 | chr4 | 99992129 | 100009931 |
| LOC100507053 | chr4 | 100010007 | 100222513 |
| LAMTOR3 | chr4 | 100799494 | 100815703 |
| DNAJB14 | chr4 | 100817406 | 100867883 |
| LOC256880 | chr4 | 100871635 | 100873620 |
| EMCN | chr4 | 101316497 | 101439250 |
| NFKB1 | chr4 | 103498870 | 103538459 |
| UBE2D3 | chr4 | 103717132 | 103790032 |
| CISD2 | chr4 | 103790134 | 103813963 |
| SLC9B2 | chr4 | 103946651 | 103998170 |
| CENPE | chr4 | 104026962 | 104119566 |
| TET2 | chr4 | 106067841 | 106200960 |
| PPA2 | chr4 | 106290233 | 106395227 |
| INTS12 | chr4 | 106603784 | 106629881 |
| GSTCD | chr4 | 106629940 | 106768882 |
| TBCK | chr4 | 106967232 | 107237423 |
| AIMP1 | chr4 | 107237663 | 107270381 |
| PAPSS1 | chr4 | 108534821 | 108641419 |
| SGMS2 | chr4 | 108745720 | 108836204 |
| RPL34-AS1 | chr4 | 109459345 | 109541613 |
| SEC24B-AS1 | chr4 | 110351118 | 110354973 |
| CCDC109B | chr4 | 110481354 | 110608872 |
| GAR1 | chr4 | 110736665 | 110745893 |
| C4orf32 | chr4 | 113066552 | 113110237 |
| AP1AR | chr4 | 113152894 | 113191211 |
| TIFA | chr4 | 113196781 | 113207059 |
| C4orf21 | chr4 | 113460488 | 113558151 |
| LARP7 | chr4 | 113558119 | 113578748 |
| CAMK2D | chr4 | 114372187 | 114683083 |
| SNHG8 | chr4 | 119199916 | 119200978 |
| SNORA24 | chr4 | 119200344 | 119200475 |
| METTL14 | chr4 | 119606573 | 119632077 |
| USP53 | chr4 | 120133781 | 120216673 |
| C4orf3 | chr4 | 120217573 | 120221968 |
| LOC645513 | chr4 | 120375937 | 120420747 |
| MAD2L1 | chr4 | 120980578 | 120988013 |
| EXOSC9 | chr4 | 122722747 | 122738176 |
| NUDT6 | chr4 | 123813798 | 123844159 |
| SPATA5 | chr4 | 123844224 | 124240604 |
| SPRY1 | chr4 | 124317949 | 124324915 |
| FAT4 | chr4 | 126237566 | 126414087 |
| PLK4 | chr4 | 128802015 | 128820377 |
| MFSD8 | chr4 | 128838959 | 128887139 |
| C4orf29 | chr4 | 128886460 | 128952455 |
| LARP1B | chr4 | 128982502 | 129132289 |
| PGRMC2 | chr4 | 129190391 | 129209984 |
| PHF17 | chr4 | 129731158 | 129796379 |
| SCLT1 | chr4 | 129805151 | 130014764 |
| C4orf33 | chr4 | 130014828 | 130033843 |
| PCDH10 | chr4 | 134070469 | 134112732 |
| ELF2 | chr4 | 139978870 | 140005568 |
| NDUFC1 | chr4 | 140211070 | 140222364 |
| SETD7 | chr4 | 140427191 | 140477577 |
| MGST2 | chr4 | 140586921 | 140661899 |
| MAML3 | chr4 | 140637545 | 141075233 |
| LOC100129858 | chr4 | 141204879 | 141294546 |
| SCOC | chr4 | 141294663 | 141303710 |
| TBC1D9 | chr4 | 141541935 | 141677471 |
| ZNF330 | chr4 | 142142048 | 142155850 |
| ANAPC10 | chr4 | 145915726 | 146019693 |
| OTUD4 | chr4 | 146080288 | 146095896 |
| SMAD1 | chr4 | 146403956 | 146480325 |
| MMAA | chr4 | 146540539 | 146581187 |
| ZNF827 | chr4 | 146681887 | 146859607 |
| LSM6 | chr4 | 147096834 | 147111213 |
| SLC10A7 | chr4 | 147175136 | 147443123 |
| TMEM184C | chr4 | 148538538 | 148556672 |
| PRMT10 | chr4 | 148559533 | 148605280 |
| NR3C2 | chr4 | 148999914 | 149363672 |
| LRBA | chr4 | 151185810 | 151936419 |
| SNORD73A | chr4 | 152024978 | 152025043 |
| SH3D19 | chr4 | 152041432 | 152147660 |
| FBXW7 | chr4 | 153242409 | 153456393 |
| DKFZP434I0714 | chr4 | 153457415 | 153460415 |
| TMEM154 | chr4 | 153547265 | 153601317 |
| ARFIP1 | chr4 | 153701111 | 153833063 |
| PLRG1 | chr4 | 155456148 | 155471585 |
| C4orf46 | chr4 | 159587826 | 159593202 |
| ETFDH | chr4 | 159593276 | 159629841 |
| PPID | chr4 | 159630278 | 159644552 |
| NAF1 | chr4 | 164049822 | 164088073 |
| TMA16 | chr4 | 164415672 | 164441691 |
| KLHL2 | chr4 | 166131170 | 166244308 |
| DDX60L | chr4 | 169277885 | 169401638 |
| CBR4 | chr4 | 169908741 | 169931468 |
| NEK1 | chr4 | 170314420 | 170533778 |
| CLCN3 | chr4 | 170541671 | 170644338 |
| GALNT7 | chr4 | 174089903 | 174245118 |
| HMGB2 | chr4 | 174252526 | 174254920 |
| SAP30 | chr4 | 174292092 | 174298683 |
| FBXO8 | chr4 | 175157809 | 175205402 |
| CEP44 | chr4 | 175205054 | 175254531 |
| WWC2-AS2 | chr4 | 184018173 | 184020352 |
| WWC2 | chr4 | 184020462 | 184241929 |
| CDKN2AIP | chr4 | 184365788 | 184369049 |
| LOC389247 | chr4 | 184415889 | 184425668 |
| ING2 | chr4 | 184426219 | 184432249 |
| RWDD4 | chr4 | 184560788 | 184580331 |
| TRAPPC11 | chr4 | 184580419 | 184634747 |
| IRF2 | chr4 | 185308875 | 185395726 |
| CCDC111 | chr4 | 185570766 | 185616112 |
| MLF1IP | chr4 | 185615218 | 185655286 |
| SNX25 | chr4 | 186131283 | 186285120 |
| UFSP2 | chr4 | 186320693 | 186347139 |
| SORBS2 | chr4 | 186506597 | 186697066 |
| FRG1 | chr4 | 190861973 | 190884359 |
| FGF2 | chr4 | 123747862 | 123819390 |
| SDHA | chr5 | 218355 | 256814 |
| KIAA0947 | chr5 | 5422785 | 5490347 |
| MED10 | chr5 | 6372038 | 6378639 |
| NSUN2 | chr5 | 6599351 | 6621602 |
| PAPD7 | chr5 | 6714717 | 6757161 |
| CCT5 | chr5 | 10250650 | 10266501 |
| 6-Mar | chr5 | 10353750 | 10440500 |
| DAP | chr5 | 10679341 | 10761387 |
| TRIO | chr5 | 14143828 | 14509458 |
| MYO10 | chr5 | 16662015 | 16936385 |
| BASP1 | chr5 | 17217669 | 17276954 |
| GOLPH3 | chr5 | 32124823 | 32174425 |
| ZFR | chr5 | 32354455 | 32444844 |
| TARS | chr5 | 33440881 | 33468196 |
| RAI14 | chr5 | 34684611 | 34832717 |
| BRIX1 | chr5 | 34915819 | 34925787 |
| NIPBL | chr5 | 36876860 | 37065921 |
| NUP155 | chr5 | 37291940 | 37371197 |
| DAB2 | chr5 | 39371775 | 39425335 |
| PTGER4 | chr5 | 40680031 | 40693837 |
| PRKAA1 | chr5 | 40759480 | 40798297 |
| RPL37 | chr5 | 40831429 | 40835387 |
| ANXA2R | chr5 | 43039181 | 43040447 |
| HMGCS1 | chr5 | 43287571 | 43313614 |
| ITGA2 | chr5 | 52285155 | 52390609 |
| ESM1 | chr5 | 54273694 | 54281414 |
| DHX29 | chr5 | 54552072 | 54603521 |
| SKIV2L2 | chr5 | 54603575 | 54721409 |
| IL6ST | chr5 | 55230924 | 55290821 |
| GPBP1 | chr5 | 56469774 | 56560506 |
| PLK2 | chr5 | 57749809 | 57755966 |
| PDE4D | chr5 | 58264865 | 59064438 |
| ZSWIM6 | chr5 | 60628099 | 60841999 |
| KIF2A | chr5 | 61601988 | 61683011 |
| LRRC70 | chr5 | 61874561 | 61877275 |
| IPO11-LRRC70 | chr5 | 61874561 | 61924416 |
| ADAMTS6 | chr5 | 64444562 | 64777704 |
| ERBB2IP | chr5 | 65222381 | 65376851 |
| SREK1 | chr5 | 65440662 | 65479444 |
| TAF9 | chr5 | 68660569 | 68665840 |
| MAP1B | chr5 | 71403117 | 71505397 |
| TNPO1 | chr5 | 72112417 | 72210215 |
| BTF3 | chr5 | 72794249 | 72801448 |
| ARHGEF28 | chr5 | 72921982 | 73237818 |
| ENC1 | chr5 | 73923230 | 73937249 |
| HMGCR | chr5 | 74632153 | 74657926 |
| F2R | chr5 | 76011867 | 76031595 |
| ARRDC3 | chr5 | 90664540 | 90679149 |
| ARRDC3-AS1 | chr5 | 90676163 | 90716532 |
| MCTP1 | chr5 | 94042288 | 94417545 |
| STARD4 | chr5 | 110834021 | 110848157 |
| DCP2 | chr5 | 112312406 | 112357892 |
| FEM1C | chr5 | 114856607 | 114880591 |
| TMED7-TICAM2 | chr5 | 114914338 | 114961876 |
| TMED7 | chr5 | 114948904 | 114961876 |
| LMNB1 | chr5 | 126112314 | 126172712 |
| PRRC1 | chr5 | 126853308 | 126890780 |
| AFF4 | chr5 | 132211070 | 132299354 |
| HSPA4 | chr5 | 132387661 | 132440709 |
| VDAC1 | chr5 | 133307565 | 133442104 |
| SKP1 | chr5 | 133492081 | 133512724 |
| PPP2CA | chr5 | 133532147 | 133561950 |
| UBE2B | chr5 | 133706869 | 133727799 |
| SEC24A | chr5 | 133984474 | 134063601 |
| DDX46 | chr5 | 134094460 | 134166812 |
| H2AFY | chr5 | 134670070 | 134734928 |
| SMAD5 | chr5 | 135468535 | 135518422 |
| HNRNPA0 | chr5 | 137087072 | 137090039 |
| ETF1 | chr5 | 137841781 | 137878989 |
| HSPA9 | chr5 | 137890570 | 137911318 |
| CTNNA1 | chr5 | 138089106 | 138270723 |
| MATR3 | chr5 | 138647450 | 138667366 |
| PAIP2 | chr5 | 138678130 | 138705409 |
| ECSCR | chr5 | 138784244 | 138842320 |
| UBE2D2 | chr5 | 138940750 | 139008018 |
| HBEGF | chr5 | 139712427 | 139726188 |
| ANKHD1-EIF4EBP3 | chr5 | 139781398 | 139929163 |
| ANKHD1 | chr5 | 139781398 | 139919441 |
| ARAP3 | chr5 | 141032967 | 141061800 |
| PCDH1 | chr5 | 141232672 | 141257944 |
| SPRY4 | chr5 | 141689991 | 141704620 |
| YIPF5 | chr5 | 143537722 | 143550278 |
| TCERG1 | chr5 | 145826872 | 145891069 |
| CSNK1A1 | chr5 | 148875456 | 148931115 |
| RPS14 | chr5 | 149823791 | 149829319 |
| SYNPO | chr5 | 150020206 | 150038792 |
| SPARC | chr5 | 151040656 | 151066615 |
| G3BP1 | chr5 | 151151475 | 151184915 |
| CNOT8 | chr5 | 154237808 | 154256352 |
| RNF145 | chr5 | 158584416 | 158636563 |
| MAT2B | chr5 | 162930069 | 162946359 |
| PANK3 | chr5 | 167982627 | 168006614 |
| SPDL1 | chr5 | 169010637 | 169031781 |
| FAM196B | chr5 | 169290718 | 169407744 |
| NPM1 | chr5 | 170814707 | 170837888 |
| SH3PXD2B | chr5 | 171760502 | 171881527 |
| DUSP1 | chr5 | 172195092 | 172198203 |
| BOD1 | chr5 | 173034147 | 173043666 |
| NOP16 | chr5 | 175813024 | 175815763 |
| UIMC1 | chr5 | 176332005 | 176433780 |
| DBN1 | chr5 | 176883613 | 176899871 |
| HNRNPAB | chr5 | 177631507 | 177638184 |
| HNRNPH1 | chr5 | 179041178 | 179051670 |
| CANX | chr5 | 179125427 | 179158639 |
| SQSTM1 | chr5 | 179247841 | 179265077 |
| MGAT1 | chr5 | 180217540 | 180242541 |
| GNB2L1 | chr5 | 180663927 | 180670906 |
| SNORD95 | chr5 | 180670313 | 180670376 |
| CCDC127 | chr5 | 204874 | 218297 |
| PDCD6 | chr5 | 271735 | 277055 |
| BRD9 | chr5 | 863849 | 892939 |
| TRIP13 | chr5 | 892968 | 918164 |
| CLPTM1L | chr5 | 1317999 | 1345002 |
| LPCAT1 | chr5 | 1461541 | 1524076 |
| MRPL36 | chr5 | 1798498 | 1799956 |
| NDUFS6 | chr5 | 1801495 | 1816167 |
| SRD5A1 | chr5 | 6633499 | 6669675 |
| PAPD7 | chr5 | 6746100 | 6757161 |
| FASTKD3 | chr5 | 7859271 | 7869150 |
| MTRR | chr5 | 7869216 | 7901235 |
| FAM173B | chr5 | 10225619 | 10250021 |
| FAM105B | chr5 | 14664782 | 14699842 |
| FBXL7 | chr5 | 15500304 | 15939900 |
| ZNF622 | chr5 | 16451627 | 16465894 |
| LOC285696 | chr5 | 17130136 | 17217531 |
| DROSHA | chr5 | 31400601 | 31532282 |
| C5orf22 | chr5 | 31532372 | 31555165 |
| MTMR12 | chr5 | 32227110 | 32313114 |
| SUB1 | chr5 | 32585604 | 32604185 |
| RAD1 | chr5 | 34905365 | 34918383 |
| DNAJC21 | chr5 | 34929697 | 34959069 |
| LMBRD2 | chr5 | 36103413 | 36152015 |
| SKP2 | chr5 | 36152144 | 36184142 |
| LOC646719 | chr5 | 36871462 | 36876796 |
| NUP155 | chr5 | 37291940 | 37370887 |
| RICTOR | chr5 | 38938021 | 39074501 |
| TTC33 | chr5 | 40711677 | 40756072 |
| LOC100506548 | chr5 | 40825364 | 40829244 |
| SNORD72 | chr5 | 40832757 | 40832837 |
| CARD6 | chr5 | 40841409 | 40855456 |
| C5orf51 | chr5 | 41904469 | 41921738 |
| FBXO4 | chr5 | 41925355 | 41935185 |
| LOC648987 | chr5 | 43016149 | 43018913 |
| LOC153684 | chr5 | 43042235 | 43045370 |
| LOC100132356 | chr5 | 43065288 | 43067073 |
| ZNF131 | chr5 | 43121609 | 43175823 |
| C5orf28 | chr5 | 43444353 | 43483992 |
| C5orf34 | chr5 | 43486802 | 43515273 |
| PAIP1 | chr5 | 43526369 | 43557195 |
| LOC100652772 | chr5 | 43573286 | 43603332 |
| NNT | chr5 | 43602790 | 43705668 |
| MRPS30 | chr5 | 44809026 | 44815618 |
| NDUFS4 | chr5 | 52856464 | 52979171 |
| SNX18 | chr5 | 53813588 | 53842416 |
| GPX8 | chr5 | 54455983 | 54463129 |
| MAP3K1 | chr5 | 56110899 | 56191978 |
| SETD9 | chr5 | 56205086 | 56213165 |
| GPBP1 | chr5 | 56509900 | 56560506 |
| PDE4D | chr5 | 58264865 | 58571945 |
| DEPDC1B | chr5 | 59892738 | 59995993 |
| ERCC8 | chr5 | 60169658 | 60240905 |
| NDUFAF2 | chr5 | 60240955 | 60448864 |
| SMIM15 | chr5 | 60453535 | 60458302 |
| DIMT1 | chr5 | 61684350 | 61699728 |
| IPO11 | chr5 | 61708572 | 61924416 |
| SREK1IP1 | chr5 | 64013977 | 64064496 |
| CWC27 | chr5 | 64064754 | 64142516 |
| CENPK | chr5 | 64813592 | 64858995 |
| PPWD1 | chr5 | 64859062 | 64883376 |
| TRIM23 | chr5 | 64885506 | 64920187 |
| TRAPPC13 | chr5 | 64920557 | 64961954 |
| SGTB | chr5 | 64961754 | 65017941 |
| NLN | chr5 | 65018022 | 65125111 |
| MAST4 | chr5 | 66124603 | 66465423 |
| PIK3R1 | chr5 | 67511583 | 67597649 |
| SLC30A5 | chr5 | 68389775 | 68426899 |
| CCNB1 | chr5 | 68462836 | 68474070 |
| CDK7 | chr5 | 68530621 | 68573257 |
| RAD17 | chr5 | 68666531 | 68710630 |
| SMN2 | chr5 | 69345349 | 69373422 |
| SMN1 | chr5 | 69345349 | 69373418 |
| BDP1 | chr5 | 70751441 | 70863649 |
| MIR4803 | chr5 | 71465293 | 71465367 |
| MRPS27 | chr5 | 71515235 | 71616084 |
| MIR4804 | chr5 | 72174417 | 72174490 |
| FCHO2 | chr5 | 72251807 | 72386349 |
| ANKRA2 | chr5 | 72848024 | 72861511 |
| UTP15 | chr5 | 72861597 | 72877794 |
| HEXB | chr5 | 73935847 | 74017113 |
| GFM2 | chr5 | 74017030 | 74063042 |
| NSA2 | chr5 | 74062815 | 74072737 |
| COL4A3BP | chr5 | 74666927 | 74807806 |
| POLK | chr5 | 74842834 | 74895646 |
| POC5 | chr5 | 74970023 | 75013313 |
| F2RL1 | chr5 | 76114832 | 76131140 |
| TBCA | chr5 | 76987073 | 77072185 |
| AP3B1 | chr5 | 77298149 | 77590579 |
| SCAMP1 | chr5 | 77656338 | 77776562 |
| LHFPL2 | chr5 | 77781037 | 77944648 |
| PAPD4 | chr5 | 78908242 | 78982471 |
| ZFYVE16 | chr5 | 79703837 | 79775498 |
| DHFR | chr5 | 79922044 | 79950800 |
| MSH3 | chr5 | 79950466 | 80172634 |
| LOC100131067 | chr5 | 80533383 | 80597388 |
| ZCCHC9 | chr5 | 80597401 | 80608965 |
| ATG10 | chr5 | 81267843 | 81551216 |
| RPS23 | chr5 | 81569138 | 81574235 |
| TMEM167A | chr5 | 82348664 | 82373272 |
| XRCC4 | chr5 | 82373316 | 82649579 |
| COX7C | chr5 | 85913783 | 85916583 |
| RASA1 | chr5 | 86564069 | 86687743 |
| CCNH | chr5 | 86690078 | 86708721 |
| TMEM161B | chr5 | 87491022 | 87564665 |
| TMEM161B-AS1 | chr5 | 87564698 | 87732491 |
| MEF2C | chr5 | 88014057 | 88179283 |
| CETN3 | chr5 | 89689528 | 89705603 |
| LYSMD3 | chr5 | 89811444 | 89825401 |
| NR2F1-AS1 | chr5 | 92877577 | 92917003 |
| NR2F1 | chr5 | 92919042 | 92929786 |
| MIR548AO | chr5 | 92922830 | 92957593 |
| ANKRD32 | chr5 | 93954390 | 94031573 |
| TTC37 | chr5 | 94799598 | 94890709 |
| ARSK | chr5 | 94890824 | 94940806 |
| RHOBTB3 | chr5 | 95066849 | 95132071 |
| ELL2 | chr5 | 95220801 | 95297775 |
| CAST | chr5 | 95997906 | 96110385 |
| ERAP2 | chr5 | 96211643 | 96255406 |
| LNPEP | chr5 | 96271345 | 96365115 |
| RIOK2 | chr5 | 96496570 | 96519005 |
| RGMB | chr5 | 98104998 | 98132198 |
| RGMB-AS1 | chr5 | 98105321 | 98108788 |
| CHD1 | chr5 | 98190907 | 98262238 |
| LOC100289230 | chr5 | 98264837 | 98266713 |
| FAM174A | chr5 | 99871123 | 99922440 |
| ST8SIA4 | chr5 | 100142638 | 100238987 |
| GIN1 | chr5 | 102421703 | 102455842 |
| PPIP5K2 | chr5 | 102455957 | 102539224 |
| C5orf30 | chr5 | 102595124 | 102614361 |
| EFNA5 | chr5 | 106712589 | 107006596 |
| FER | chr5 | 108083522 | 108523373 |
| PJA2 | chr5 | 108670409 | 108745675 |
| MAN2A1 | chr5 | 109025155 | 109203429 |
| WDR36 | chr5 | 110427869 | 110466200 |
| STARD4-AS1 | chr5 | 110847923 | 111075423 |
| NREP | chr5 | 111064999 | 111091948 |
| EPB41L4A-AS1 | chr5 | 111496222 | 111498198 |
| SNORA13 | chr5 | 111497181 | 111497314 |
| APC | chr5 | 112073555 | 112181936 |
| SRP19 | chr5 | 112196884 | 112228776 |
| REEP5 | chr5 | 112212080 | 112258031 |
| MCC | chr5 | 112357795 | 112630612 |
| YTHDC2 | chr5 | 112849390 | 112930984 |
| PGGT1B | chr5 | 114546526 | 114598569 |
| ATG12 | chr5 | 115163893 | 115177548 |
| AP3S1 | chr5 | 115177618 | 115249778 |
| COMMD10 | chr5 | 115420726 | 115628978 |
| DMXL1 | chr5 | 118407083 | 118584822 |
| SRFBP1 | chr5 | 121297655 | 121364295 |
| LOX | chr5 | 121398889 | 121414055 |
| SNX2 | chr5 | 122110690 | 122170234 |
| CEP120 | chr5 | 122680578 | 122759286 |
| CSNK1G3 | chr5 | 122847792 | 122952738 |
| ZNF608 | chr5 | 123972609 | 124080865 |
| PHAX | chr5 | 125936606 | 125962944 |
| 3-Mar | chr5 | 126203405 | 126366500 |
| FLJ33630 | chr5 | 127357243 | 127418766 |
| SLC12A2 | chr5 | 127419482 | 127525380 |
| HINT1 | chr5 | 130494975 | 130501041 |
| CDC42SE2 | chr5 | 130599701 | 130730360 |
| RAPGEF6 | chr5 | 130759613 | 131132756 |
| FNIP1 | chr5 | 130977406 | 131132756 |
| PDLIM4 | chr5 | 131593350 | 131609147 |
| SLC22A4 | chr5 | 131630144 | 131679899 |
| C5orf56 | chr5 | 131746464 | 131811736 |
| IRF1 | chr5 | 131817300 | 131826465 |
| 8-Sep | chr5 | 132091696 | 132113067 |
| UQCRQ | chr5 | 132202318 | 132204536 |
| ZCCHC10 | chr5 | 132332677 | 132362240 |
| C5orf15 | chr5 | 133291197 | 133304406 |
| MIR3661 | chr5 | 133561447 | 133561543 |
| CDKN2AIPNL | chr5 | 133737755 | 133747598 |
| CAMLG | chr5 | 134074169 | 134087850 |
| C5orf24 | chr5 | 134181657 | 134195425 |
| SMAD5-AS1 | chr5 | 135465202 | 135470579 |
| SPOCK1 | chr5 | 136310986 | 136834438 |
| FAM13B | chr5 | 137273641 | 137368802 |
| BRD8 | chr5 | 137475458 | 137514358 |
| KIF20A | chr5 | 137514416 | 137523404 |
| CDC23 | chr5 | 137523336 | 137549032 |
| FAM53C | chr5 | 137673223 | 137685418 |
| KDM3B | chr5 | 137688284 | 137772716 |
| LRRTM2 | chr5 | 138205078 | 138211057 |
| SIL1 | chr5 | 138282409 | 138534065 |
| MATR3 | chr5 | 138609440 | 138667366 |
| SNHG4 | chr5 | 138609440 | 138615317 |
| SNORA74A | chr5 | 138614468 | 138614668 |
| PAIP2 | chr5 | 138678133 | 138705409 |
| TMEM173 | chr5 | 138855112 | 138862343 |
| PFDN1 | chr5 | 139624634 | 139682689 |
| SRA1 | chr5 | 139929651 | 139937678 |
| APBB3 | chr5 | 139937852 | 139944189 |
| SLC35A4 | chr5 | 139944475 | 139948683 |
| NDUFA2 | chr5 | 140024947 | 140027370 |
| IK | chr5 | 140027383 | 140042065 |
| MIR3655 | chr5 | 140027428 | 140027511 |
| WDR55 | chr5 | 140044383 | 140050553 |
| HARS | chr5 | 140053489 | 140064923 |
| HARS2 | chr5 | 140071017 | 140078890 |
| ZMAT2 | chr5 | 140080031 | 140086239 |
| TAF7 | chr5 | 140698056 | 140700351 |
| DIAPH1 | chr5 | 140894587 | 140998622 |
| LOC100505658 | chr5 | 140937877 | 140944827 |
| HDAC3 | chr5 | 141000442 | 141014729 |
| KIAA0141 | chr5 | 141303384 | 141321612 |
| RNF14 | chr5 | 141348450 | 141369856 |
| NDFIP1 | chr5 | 141488323 | 141534008 |
| NR3C1 | chr5 | 142657495 | 142815077 |
| KCTD16 | chr5 | 143584839 | 143856944 |
| LARS | chr5 | 145492588 | 145562294 |
| RBM27 | chr5 | 145583162 | 145668784 |
| DPYSL3 | chr5 | 146770370 | 146889619 |
| FBXO38 | chr5 | 147774276 | 147822399 |
| ADRB2 | chr5 | 148206155 | 148208197 |
| SH3TC2 | chr5 | 148361712 | 148442737 |
| ABLIM3 | chr5 | 148521176 | 148639999 |
| GRPEL2 | chr5 | 148724976 | 148734146 |
| TIGD6 | chr5 | 149372685 | 149380730 |
| HMGXB3 | chr5 | 149380168 | 149432706 |
| TCOF1 | chr5 | 149737201 | 149779871 |
| RBM22 | chr5 | 150070351 | 150080669 |
| DCTN4 | chr5 | 150088308 | 150138082 |
| TNIP1 | chr5 | 150409503 | 150460649 |
| ANXA6 | chr5 | 150480266 | 150537443 |
| FAM114A2 | chr5 | 153371268 | 153418426 |
| MFAP3 | chr5 | 153418518 | 153437014 |
| GEMIN5 | chr5 | 154266975 | 154317776 |
| MRPL22 | chr5 | 154320632 | 154348971 |
| MED7 | chr5 | 156565450 | 156569786 |
| CLINT1 | chr5 | 157212750 | 157286183 |
| RNF145 | chr5 | 158584416 | 158634656 |
| TTC1 | chr5 | 159436179 | 159492550 |
| C5orf54 | chr5 | 159820154 | 159827060 |
| SLU7 | chr5 | 159828647 | 159846168 |
| PTTG1 | chr5 | 159849214 | 159855746 |
| MIR3142 | chr5 | 159901408 | 159901490 |
| CCNG1 | chr5 | 162864576 | 162872022 |
| NUDCD2 | chr5 | 162880585 | 162887143 |
| HMMR | chr5 | 162887516 | 162918953 |
| RARS | chr5 | 167913462 | 167946309 |
| MIR3912 | chr5 | 170813659 | 170813764 |
| FBXW11 | chr5 | 171288555 | 171433877 |
| UBTD2 | chr5 | 171636649 | 171710795 |
| ERGIC1 | chr5 | 172261222 | 172379688 |
| RPL26L1 | chr5 | 172386438 | 172396774 |
| ATP6V0E1 | chr5 | 172410762 | 172461900 |
| STC2 | chr5 | 172741725 | 172756506 |
| CPEB4 | chr5 | 173315330 | 173387313 |
| SFXN1 | chr5 | 174905513 | 174955621 |
| KIAA1191 | chr5 | 175773065 | 175788809 |
| HIGD2A | chr5 | 175815783 | 175816751 |
| CLTB | chr5 | 175819455 | 175843570 |
| FAF2 | chr5 | 175875355 | 175937075 |
| RNF44 | chr5 | 175953699 | 175964421 |
| ZNF346 | chr5 | 176449696 | 176493745 |
| NSD1 | chr5 | 176560832 | 176727214 |
| RAB24 | chr5 | 176728198 | 176730744 |
| PRELID1 | chr5 | 176730762 | 176733960 |
| MXD3 | chr5 | 176732500 | 176739292 |
| LMAN2 | chr5 | 176758562 | 176778885 |
| GRK6 | chr5 | 176853686 | 176869850 |
| PDLIM7 | chr5 | 176910394 | 176924602 |
| DDX41 | chr5 | 176938577 | 176943917 |
| FAM193B | chr5 | 176946789 | 176981548 |
| TMED9 | chr5 | 177019212 | 177023099 |
| NHP2 | chr5 | 177576464 | 177580961 |
| AGXT2L2 | chr5 | 177635539 | 177659803 |
| CLK4 | chr5 | 178029664 | 178054054 |
| RUFY1 | chr5 | 178977561 | 179037027 |
| MAML1 | chr5 | 179159850 | 179223512 |
| MGAT4B | chr5 | 179224597 | 179233952 |
| SQSTM1 | chr5 | 179233387 | 179265077 |
| TBC1D9B | chr5 | 179289070 | 179334856 |
| CNOT6 | chr5 | 179921432 | 180005353 |
| MGAT1 | chr5 | 180217540 | 180236817 |
| TRIM41 | chr5 | 180650262 | 180662808 |
| SNORD96A | chr5 | 180668817 | 180668889 |
| TRIM52 | chr5 | 180683385 | 180688119 |
| FOXC1 | chr6 | 1610680 | 1614129 |
| WRNIP1 | chr6 | 2765665 | 2785979 |
| PXDC1 | chr6 | 3722835 | 3752246 |
| PRPF4B | chr6 | 4021568 | 4065217 |
| SSR1 | chr6 | 7281287 | 7313541 |
| BMP6 | chr6 | 7727010 | 7881961 |
| BLOC1S5-TXNDC5 | chr6 | 7881482 | 8064647 |
| TXNDC5 | chr6 | 7881482 | 7911047 |
| NEDD9 | chr6 | 11183530 | 11382581 |
| EDN1 | chr6 | 12290528 | 12296773 |
| RANBP9 | chr6 | 13621729 | 13711796 |
| NUP153 | chr6 | 17615265 | 17707065 |
| DEK | chr6 | 18224399 | 18264799 |
| RNF144B | chr6 | 18387580 | 18469105 |
| SOX4 | chr6 | 21593971 | 21598849 |
| C6orf62 | chr6 | 24705089 | 24719403 |
| GMNN | chr6 | 24775158 | 24786325 |
| TRIM27 | chr6 | 28870778 | 28891768 |
| HLA-E | chr6 | 30457182 | 30461982 |
| PPP1R10 | chr6 | 30568176 | 30585084 |
| PPP1R18 | chr6 | 30644165 | 30655672 |
| IER3 | chr6 | 30710975 | 30712327 |
| MICA | chr6 | 31367560 | 31383090 |
| ATP6V1G2-DDX39B | chr6 | 31497995 | 31514625 |
| SNORD84 | chr6 | 31508877 | 31508955 |
| PRRC2A | chr6 | 31588449 | 31605554 |
| C6orf48 | chr6 | 31802692 | 31807541 |
| SNORD48 | chr6 | 31803039 | 31803103 |
| SLC39A7 | chr6 | 33168602 | 33172214 |
| RPS18 | chr6 | 33239851 | 33244281 |
| KIFC1 | chr6 | 33359312 | 33377699 |
| LEMD2 | chr6 | 33738989 | 33756906 |
| HMGA1 | chr6 | 34204649 | 34214008 |
| RPS10-NUDT3 | chr6 | 34254972 | 34393902 |
| RPS10 | chr6 | 34385230 | 34393818 |
| RPL10A | chr6 | 35436177 | 35438558 |
| MAPK14 | chr6 | 35995453 | 36079013 |
| SRSF3 | chr6 | 36562089 | 36572244 |
| CDKN1A | chr6 | 36646486 | 36655116 |
| MTCH1 | chr6 | 36935910 | 36954327 |
| ZFAND3 | chr6 | 37787306 | 38122399 |
| NFYA | chr6 | 41040960 | 41070146 |
| BYSL | chr6 | 41888964 | 41900784 |
| MRPL14 | chr6 | 44081372 | 44095191 |
| SLC29A1 | chr6 | 44191362 | 44201888 |
| HSP90AB1 | chr6 | 44214694 | 44221625 |
| TRAM2 | chr6 | 52362199 | 52441862 |
| ELOVL5 | chr6 | 53132195 | 53213977 |
| RPS16P5 | chr6 | 53199257 | 53202415 |
| PTP4A1 | chr6 | 64281919 | 64293489 |
| LINC00472 | chr6 | 72124148 | 72130448 |
| EEF1A1 | chr6 | 74225472 | 74230741 |
| TMEM30A | chr6 | 75962637 | 75994632 |
| SENP6 | chr6 | 76311621 | 76427994 |
| HTR1B | chr6 | 78171947 | 78173120 |
| TBX18 | chr6 | 85442215 | 85473954 |
| SYNCRIP | chr6 | 86317501 | 86353043 |
| SNHG5 | chr6 | 86386724 | 86388451 |
| SNORD50A | chr6 | 86387011 | 86387086 |
| SNORD50B | chr6 | 86387306 | 86387377 |
| AKIRIN2 | chr6 | 88384577 | 88411985 |
| UBE2J1 | chr6 | 90036343 | 90062619 |
| PNISR | chr6 | 99847840 | 99873207 |
| SEC63 | chr6 | 108188959 | 108279482 |
| CD164 | chr6 | 109687716 | 109703762 |
| AMD1 | chr6 | 111195986 | 111216913 |
| LAMA4 | chr6 | 112429133 | 112575917 |
| MARCKS | chr6 | 114178526 | 114184652 |
| HDAC2 | chr6 | 114257319 | 114292359 |
| TSPYL4 | chr6 | 116571130 | 116575261 |
| DCBLD1 | chr6 | 117860417 | 117870960 |
| GJA1 | chr6 | 121756744 | 121770873 |
| PTPRK | chr6 | 128289923 | 128841870 |
| ARHGAP18 | chr6 | 129898239 | 130031370 |
| CTGF | chr6 | 132269316 | 132272518 |
| LOC100507254 | chr6 | 132455117 | 132490514 |
| RPS12 | chr6 | 133135707 | 133138703 |
| SGK1 | chr6 | 134490383 | 134497070 |
| BCLAF1 | chr6 | 136578000 | 136610989 |
| CITED2 | chr6 | 139693391 | 139695787 |
| GPR126 | chr6 | 142623055 | 142767403 |
| PHACTR2 | chr6 | 143999101 | 144152322 |
| SASH1 | chr6 | 148663728 | 148873184 |
| PPIL4 | chr6 | 149836291 | 149867238 |
| MTHFD1L | chr6 | 151196378 | 151423023 |
| AKAP12 | chr6 | 151561133 | 151679694 |
| MYCT1 | chr6 | 153019029 | 153045715 |
| FBXO5 | chr6 | 153291657 | 153304740 |
| SCAF8 | chr6 | 155054511 | 155155194 |
| ARID1B | chr6 | 157099063 | 157531913 |
| SNX9 | chr6 | 158244293 | 158366109 |
| SYNJ2 | chr6 | 158402887 | 158520207 |
| WTAP | chr6 | 160172233 | 160177352 |
| TCP1 | chr6 | 160199529 | 160210735 |
| QKI | chr6 | 163835674 | 163999628 |
| PSMB1 | chr6 | 170780106 | 170862417 |
| DUSP22 | chr6 | 304627 | 351353 |
| EXOC2 | chr6 | 485137 | 693141 |
| MIR4645 | chr6 | 2854264 | 2854341 |
| MGC39372 | chr6 | 2854890 | 2876744 |
| SERPINB9 | chr6 | 2887499 | 2903546 |
| SERPINB6 | chr6 | 2948392 | 2962404 |
| LINC01011 | chr6 | 2988200 | 2991405 |
| DKFZP686I15217 | chr6 | 2988200 | 2991405 |
| PSMG4 | chr6 | 3259161 | 3268300 |
| ECI2 | chr6 | 4115926 | 4135831 |
| CDYL | chr6 | 4776679 | 4955778 |
| LYRM4 | chr6 | 5108652 | 5261172 |
| FARS2 | chr6 | 5261276 | 5771816 |
| RREB1 | chr6 | 7108085 | 7252213 |
| CAGE1 | chr6 | 7326886 | 7389942 |
| RIOK1 | chr6 | 7390061 | 7418270 |
| SNRNP48 | chr6 | 7590431 | 7612200 |
| BLOC1S5-TXNDC5 | chr6 | 7881482 | 8064647 |
| EEF1E1-MUTED | chr6 | 8013799 | 8102828 |
| EEF1E1-BLOC1S5 | chr6 | 8013799 | 8102828 |
| BLOC1S5 | chr6 | 8013799 | 8064647 |
| EEF1E1 | chr6 | 8079627 | 8102828 |
| SCARNA27 | chr6 | 8086640 | 8086766 |
| SLC35B3 | chr6 | 8413300 | 8435794 |
| LOC100506207 | chr6 | 8435855 | 8712526 |
| GCNT2 | chr6 | 10528588 | 10629601 |
| C6orf52 | chr6 | 10671650 | 10695030 |
| PAK1IP1 | chr6 | 10695187 | 10709970 |
| TMEM14B | chr6 | 10747994 | 10757214 |
| SMIM13 | chr6 | 11094265 | 11138969 |
| ERVFRD-1 | chr6 | 11102721 | 11112071 |
| HIVEP1 | chr6 | 12012723 | 12165232 |
| GFOD1 | chr6 | 13363586 | 13487869 |
| NOL7 | chr6 | 13615558 | 13621127 |
| MYLIP | chr6 | 16129316 | 16148478 |
| GMPR | chr6 | 16238810 | 16295780 |
| ATXN1 | chr6 | 16299342 | 16761721 |
| KIF13A | chr6 | 17759413 | 17987854 |
| E2F3 | chr6 | 20402136 | 20493945 |
| CDKAL1 | chr6 | 20534687 | 21232634 |
| TDP2 | chr6 | 24650204 | 24667115 |
| ACOT13 | chr6 | 24667262 | 24705295 |
| GMNN | chr6 | 24775640 | 24786325 |
| TRIM38 | chr6 | 25962916 | 25987557 |
| HIST1H3A | chr6 | 26020717 | 26021186 |
| HIST1H4A | chr6 | 26021906 | 26022278 |
| HIST1H4B | chr6 | 26027123 | 26027480 |
| HIST1H3B | chr6 | 26031816 | 26032288 |
| HIST1H2AB | chr6 | 26033319 | 26033796 |
| HIST1H2BB | chr6 | 26043454 | 26043885 |
| HIST1H3C | chr6 | 26045638 | 26046097 |
| HIST1H1C | chr6 | 26055967 | 26056699 |
| HIST1H2BC | chr6 | 26114625 | 26124154 |
| HIST1H2AC | chr6 | 26124372 | 26124918 |
| HIST1H1E | chr6 | 26156558 | 26157343 |
| HIST1H2BD | chr6 | 26158348 | 26171576 |
| HIST1H3D | chr6 | 26197011 | 26199521 |
| HIST1H2AD | chr6 | 26199011 | 26199521 |
| HIST1H2BF | chr6 | 26199786 | 26200216 |
| HIST1H4E | chr6 | 26204872 | 26205249 |
| HIST1H2BG | chr6 | 26216427 | 26216872 |
| HIST1H2AE | chr6 | 26217147 | 26217711 |
| HIST1H3F | chr6 | 26224426 | 26226589 |
| HIST1H3E | chr6 | 26225382 | 26225844 |
| HIST1H1D | chr6 | 26234439 | 26235216 |
| HIST1H2BH | chr6 | 26251878 | 26252303 |
| HIST1H3G | chr6 | 26271145 | 26271612 |
| HIST1H2BI | chr6 | 26273203 | 26273640 |
| HIST1H4H | chr6 | 26285353 | 26285727 |
| BTN2A3P | chr6 | 26421618 | 26430816 |
| BTN2A1 | chr6 | 26458152 | 26469866 |
| HMGN4 | chr6 | 26538571 | 26547164 |
| ABT1 | chr6 | 26597170 | 26600277 |
| HIST1H2BJ | chr6 | 27100094 | 27100575 |
| HIST1H2AG | chr6 | 27100816 | 27101314 |
| HIST1H2BK | chr6 | 27106071 | 27114637 |
| HIST1H2AH | chr6 | 27114860 | 27115341 |
| HIST1H2BL | chr6 | 27775256 | 27775709 |
| HIST1H3H | chr6 | 27775976 | 27778314 |
| HIST1H2AI | chr6 | 27775976 | 27776445 |
| HIST1H4J | chr6 | 27791902 | 27792258 |
| HIST1H4K | chr6 | 27798951 | 27799305 |
| HIST1H2AK | chr6 | 27805657 | 27806117 |
| HIST1H2BN | chr6 | 27806378 | 27819974 |
| HIST1H2AL | chr6 | 27833106 | 27833576 |
| HIST1H1B | chr6 | 27834569 | 27835359 |
| HIST1H3I | chr6 | 27839622 | 27840099 |
| HIST1H4L | chr6 | 27840925 | 27841289 |
| HIST1H2AM | chr6 | 27860476 | 27860963 |
| HIST1H2BO | chr6 | 27861202 | 27861669 |
| ZSCAN16 | chr6 | 28092386 | 28097856 |
| ZKSCAN8 | chr6 | 28109687 | 28127250 |
| ZSCAN9 | chr6 | 28193028 | 28201264 |
| ZSCAN26 | chr6 | 28234787 | 28245980 |
| ZKSCAN3 | chr6 | 28317690 | 28336954 |
| TRIM26 | chr6 | 30152231 | 30181271 |
| HCG17 | chr6 | 30201815 | 30293911 |
| HCG18 | chr6 | 30255173 | 30294933 |
| TRIM39 | chr6 | 30295007 | 30311506 |
| GNL1 | chr6 | 30509154 | 30525371 |
| PRR3 | chr6 | 30524485 | 30532473 |
| MRPS18B | chr6 | 30585485 | 30594174 |
| DHX16 | chr6 | 30620895 | 30640830 |
| NRM | chr6 | 30655823 | 30659197 |
| MDC1 | chr6 | 30667583 | 30685458 |
| FLOT1 | chr6 | 30695510 | 30710453 |
| MICB | chr6 | 31465854 | 31478901 |
| SNORD117 | chr6 | 31504150 | 31504226 |
| NFKBIL1 | chr6 | 31515352 | 31526606 |
| SNORA38 | chr6 | 31590855 | 31590987 |
| APOM | chr6 | 31623670 | 31625987 |
| LY6G5B | chr6 | 31634597 | 31640227 |
| ABHD16A | chr6 | 31654725 | 31681842 |
| DDAH2 | chr6 | 31694816 | 31697569 |
| VARS | chr6 | 31745296 | 31763712 |
| HSPA1L | chr6 | 31777395 | 31782835 |
| HSPA1A | chr6 | 31783290 | 31785719 |
| SNORD52 | chr6 | 31804852 | 31804916 |
| NELFE | chr6 | 31919863 | 31926864 |
| SKIV2L | chr6 | 31926580 | 31937532 |
| DOM3Z | chr6 | 31937587 | 31940032 |
| STK19 | chr6 | 31938951 | 31949223 |
| ATF6B | chr6 | 32083044 | 32096017 |
| FKBPL | chr6 | 32096483 | 32098067 |
| AGPAT1 | chr6 | 32135982 | 32143916 |
| RNF5 | chr6 | 32146161 | 32151930 |
| PBX2 | chr6 | 32152509 | 32157963 |
| GPSM3 | chr6 | 32158542 | 32160692 |
| HSD17B8 | chr6 | 33172413 | 33174608 |
| VPS52 | chr6 | 33218048 | 33239662 |
| B3GALT4 | chr6 | 33244916 | 33246602 |
| WDR46 | chr6 | 33246879 | 33257304 |
| PFDN6 | chr6 | 33257411 | 33258711 |
| RGL2 | chr6 | 33259430 | 33267165 |
| TAPBP | chr6 | 33267471 | 33282164 |
| ZBTB22 | chr6 | 33282181 | 33285512 |
| CUTA | chr6 | 33384318 | 33386065 |
| BAK1 | chr6 | 33540322 | 33548070 |
| ITPR3 | chr6 | 33589155 | 33664348 |
| MNF1 | chr6 | 33664537 | 33679528 |
| LEMD2 | chr6 | 33738989 | 33754719 |
| C6orf1 | chr6 | 34214156 | 34216904 |
| NUDT3 | chr6 | 34254972 | 34360457 |
| C6orf106 | chr6 | 34555065 | 34664625 |
| SNRPC | chr6 | 34724870 | 34741634 |
| TAF11 | chr6 | 34845554 | 34855848 |
| ANKS1A | chr6 | 34857037 | 35059190 |
| PPARD | chr6 | 35310334 | 35395968 |
| FKBP5 | chr6 | 35548333 | 35656719 |
| MIR5690 | chr6 | 35632493 | 35632566 |
| SRPK1 | chr6 | 35800810 | 35888842 |
| BRPF3 | chr6 | 36164549 | 36200567 |
| PXT1 | chr6 | 36358328 | 36410666 |
| KCTD20 | chr6 | 36410543 | 36458315 |
| STK38 | chr6 | 36461668 | 36508567 |
| CDKN1A | chr6 | 36645587 | 36655116 |
| PPIL1 | chr6 | 36822605 | 36842800 |
| TMEM217 | chr6 | 37185417 | 37225931 |
| TBC1D22B | chr6 | 37225547 | 37300746 |
| FTSJD2 | chr6 | 37400906 | 37449284 |
| GLO1 | chr6 | 38643701 | 38670952 |
| OARD1 | chr6 | 41036776 | 41040268 |
| TOMM6 | chr6 | 41754499 | 41757634 |
| MED20 | chr6 | 41873091 | 41888877 |
| CCND3 | chr6 | 41902670 | 41909552 |
| TAF8 | chr6 | 42018657 | 42048644 |
| UBR2 | chr6 | 42531759 | 42661243 |
| TBCC | chr6 | 42712233 | 42713884 |
| RPL7L1 | chr6 | 42847670 | 42855668 |
| CNPY3 | chr6 | 42896859 | 42907008 |
| MEA1 | chr6 | 42979966 | 42981618 |
| KLHDC3 | chr6 | 42981840 | 42989036 |
| RRP36 | chr6 | 42989384 | 42997337 |
| CUL7 | chr6 | 43005354 | 43021683 |
| MRPL2 | chr6 | 43021766 | 43027242 |
| KLC4 | chr6 | 43027142 | 43042833 |
| SRF | chr6 | 43138919 | 43149244 |
| YIPF3 | chr6 | 43479564 | 43484728 |
| POLR1C | chr6 | 43484776 | 43489246 |
| XPO5 | chr6 | 43490067 | 43543812 |
| POLH | chr6 | 43543877 | 43588260 |
| GTPBP2 | chr6 | 43588217 | 43596936 |
| MAD2L1BP | chr6 | 43597278 | 43608688 |
| MRPS18A | chr6 | 43638933 | 43655549 |
| TMEM63B | chr6 | 44095375 | 44123256 |
| SLC29A1 | chr6 | 44187241 | 44201888 |
| SLC35B2 | chr6 | 44221837 | 44225283 |
| MIR4647 | chr6 | 44221942 | 44222022 |
| NFKBIE | chr6 | 44225902 | 44233525 |
| CDC5L | chr6 | 44355250 | 44418161 |
| TNFRSF21 | chr6 | 47199267 | 47277680 |
| CD2AP | chr6 | 47445524 | 47594996 |
| MUT | chr6 | 49398072 | 49431041 |
| CENPQ | chr6 | 49431095 | 49460820 |
| MCM3 | chr6 | 52128811 | 52149679 |
| EFHC1 | chr6 | 52284993 | 52360583 |
| GSTA4 | chr6 | 52842745 | 52860178 |
| LRRC1 | chr6 | 53659533 | 53788919 |
| KIAA1586 | chr6 | 56911383 | 56920023 |
| ZNF451 | chr6 | 56954807 | 56974512 |
| PRIM2 | chr6 | 57182421 | 57513376 |
| SMAP1 | chr6 | 71377478 | 71571716 |
| OGFRL1 | chr6 | 71998476 | 72012230 |
| MIR30A | chr6 | 72113253 | 72113324 |
| MTO1 | chr6 | 74171453 | 74211179 |
| CD109 | chr6 | 74405507 | 74538041 |
| COX7A2 | chr6 | 75947390 | 75953644 |
| LOC100506804 | chr6 | 75994729 | 76001580 |
| MYO6 | chr6 | 76458908 | 76629254 |
| PHIP | chr6 | 79644135 | 79788011 |
| HMGN3 | chr6 | 79910961 | 79944455 |
| LCA5 | chr6 | 80194707 | 80247147 |
| TTK | chr6 | 80714321 | 80752244 |
| IBTK | chr6 | 82879955 | 82957448 |
| PGM3 | chr6 | 83874592 | 83903655 |
| RWDD2A | chr6 | 83903031 | 83906256 |
| ME1 | chr6 | 83920109 | 84140938 |
| NT5E | chr6 | 86159301 | 86205509 |
| SNX14 | chr6 | 86215214 | 86278201 |
| ZNF292 | chr6 | 87865268 | 87973406 |
| RARS2 | chr6 | 88224095 | 88299735 |
| ORC3 | chr6 | 88299784 | 88377172 |
| RNGTT | chr6 | 89319988 | 89673348 |
| PNRC1 | chr6 | 89791554 | 89794879 |
| LYRM2 | chr6 | 90341942 | 90348216 |
| MDN1 | chr6 | 90353230 | 90529442 |
| CASP8AP2 | chr6 | 90539618 | 90584155 |
| MAP3K7 | chr6 | 91223291 | 91297020 |
| MANEA | chr6 | 96025372 | 96057328 |
| UFL1 | chr6 | 96969701 | 97003151 |
| NDUFAF4 | chr6 | 97337186 | 97345767 |
| MMS22L | chr6 | 97590036 | 97731052 |
| CCNC | chr6 | 99990262 | 100016690 |
| ASCC3 | chr6 | 100956607 | 101329224 |
| PREP | chr6 | 105725441 | 105850999 |
| ATG5 | chr6 | 106632351 | 106773695 |
| QRSL1 | chr6 | 107077440 | 107116292 |
| SNX3 | chr6 | 108532716 | 108582464 |
| SESN1 | chr6 | 109307639 | 109415708 |
| CEP57L1 | chr6 | 109416355 | 109485115 |
| ZBTB24 | chr6 | 109783718 | 109804440 |
| WASF1 | chr6 | 110421021 | 110501207 |
| CDC40 | chr6 | 110501586 | 110553422 |
| RPF2 | chr6 | 111303290 | 111346794 |
| REV3L | chr6 | 111620233 | 111804432 |
| TRAF3IP2-AS1 | chr6 | 111804674 | 111923497 |
| TRAF3IP2 | chr6 | 111876580 | 111927477 |
| FYN | chr6 | 111981534 | 112194655 |
| TSPYL1 | chr6 | 116596021 | 116601280 |
| DSE | chr6 | 116601282 | 116759442 |
| FAM26E | chr6 | 116832807 | 116839709 |
| RWDD1 | chr6 | 116892582 | 116914436 |
| ZUFSP | chr6 | 116956780 | 116989973 |
| GOPC | chr6 | 117881432 | 117923705 |
| NUS1 | chr6 | 117996616 | 118031886 |
| CEP85L | chr6 | 118781934 | 118973020 |
| ASF1A | chr6 | 119215240 | 119230335 |
| HSF2 | chr6 | 122720695 | 122754264 |
| SERINC1 | chr6 | 122764492 | 122792952 |
| PKIB | chr6 | 122793061 | 123047518 |
| STL | chr6 | 125229391 | 125284173 |
| RNF217 | chr6 | 125304513 | 125404661 |
| NCOA7 | chr6 | 126102306 | 126253176 |
| TRMT11 | chr6 | 126307575 | 126360420 |
| CENPW | chr6 | 126661252 | 126669754 |
| RNF146 | chr6 | 127587882 | 127609705 |
| ECHDC1 | chr6 | 127609856 | 127664754 |
| L3MBTL3 | chr6 | 130339727 | 130462594 |
| EPB41L2 | chr6 | 131160487 | 131204234 |
| STX7 | chr6 | 132778662 | 132834337 |
| SNORA33 | chr6 | 133136445 | 133138490 |
| SNORD101 | chr6 | 133136445 | 133136518 |
| SNORD100 | chr6 | 133137940 | 133138016 |
| TBPL1 | chr6 | 134273307 | 134308638 |
| HBS1L | chr6 | 135281516 | 135376036 |
| AHI1 | chr6 | 135605109 | 135818903 |
| LINC00271 | chr6 | 135818938 | 136011976 |
| IFNGR1 | chr6 | 137518620 | 137540567 |
| NHSL1 | chr6 | 138743180 | 138893668 |
| FLJ46906 | chr6 | 139012804 | 139018425 |
| LOC100507462 | chr6 | 139046347 | 139094816 |
| CCDC28A | chr6 | 139094656 | 139114456 |
| REPS1 | chr6 | 139225619 | 139309398 |
| ABRACL | chr6 | 139349818 | 139364439 |
| VTA1 | chr6 | 142468409 | 142542085 |
| ADAT2 | chr6 | 143743968 | 143771841 |
| PEX3 | chr6 | 143771917 | 143811751 |
| LTV1 | chr6 | 144164507 | 144184943 |
| SF3B5 | chr6 | 144416017 | 144416754 |
| UTRN | chr6 | 144612872 | 145174170 |
| FBXO30 | chr6 | 146119271 | 146135921 |
| LOC100507557 | chr6 | 146136011 | 146207721 |
| SHPRH | chr6 | 146205944 | 146285233 |
| RAB32 | chr6 | 146864827 | 146876086 |
| STXBP5-AS1 | chr6 | 147162524 | 147525750 |
| STXBP5 | chr6 | 147525493 | 147711612 |
| TAB2 | chr6 | 149663820 | 149732747 |
| LATS1 | chr6 | 149979288 | 150039392 |
| NUP43 | chr6 | 150045456 | 150067688 |
| PCMT1 | chr6 | 150070830 | 150132557 |
| ZBTB2 | chr6 | 151685249 | 151712677 |
| RMND1 | chr6 | 151725896 | 151773316 |
| C6orf211 | chr6 | 151773421 | 151791232 |
| MTRF1L | chr6 | 153308399 | 153323925 |
| TFB1M | chr6 | 155577263 | 155635617 |
| MIR4466 | chr6 | 157100811 | 157100865 |
| TMEM242 | chr6 | 157710053 | 157745253 |
| SYNJ2-IT1 | chr6 | 158422138 | 158423415 |
| SYNJ2 | chr6 | 158438079 | 158520207 |
| DYNLT1 | chr6 | 159057506 | 159065804 |
| EZR | chr6 | 159186772 | 159239340 |
| SOD2 | chr6 | 160112026 | 160114353 |
| LOC100129518 | chr6 | 160181290 | 160183364 |
| ACAT2 | chr6 | 160183510 | 160200087 |
| SNORA29 | chr6 | 160206625 | 160206765 |
| MRPL18 | chr6 | 160211491 | 160219461 |
| MAP3K4 | chr6 | 161412821 | 161538417 |
| AGPAT4 | chr6 | 161551056 | 161695107 |
| CAHM | chr6 | 163834096 | 163834982 |
| SFT2D1 | chr6 | 166733516 | 166755991 |
| LOC100289495 | chr6 | 166756118 | 166764957 |
| RPS6KA2 | chr6 | 166822853 | 167040726 |
| MIR3939 | chr6 | 167411294 | 167411400 |
| FGFR1OP | chr6 | 167412815 | 167454066 |
| MLLT4 | chr6 | 168228314 | 168372700 |
| WDR27 | chr6 | 169857302 | 170102159 |
| C6orf120 | chr6 | 170102256 | 170106402 |
| PHF10 | chr6 | 170107131 | 170116184 |
| TCTE3 | chr6 | 170140214 | 170151638 |
| C6orf70 | chr6 | 170167110 | 170181617 |
| FAM120B | chr6 | 170615843 | 170714237 |
| TBP | chr6 | 170863420 | 170881958 |
| PDCD2 | chr6 | 170884659 | 170893780 |
| VEGFA | chr6 | 43737945 | 43754223 |
| MSH5-SAPCD1 | chr6_apd_hap1 | 3037482 | 3045696 |
| DDX39B | chr6_cox_hap2 | 3011436 | 3019439 |
| CLIC1 | chr6_cox_hap2 | 3207974 | 3214716 |
| ABCF1 | chr6_cox_hap2 | 2051220 | 2071356 |
| BAG6 | chr6_cox_hap2 | 3116415 | 3130096 |
| C6orf47 | chr6_cox_hap2 | 3135692 | 3138168 |
| DAXX | chr6_cox_hap2 | 4730148 | 4734611 |
| CSNK2B | chr6_dbb_hap3 | 2919235 | 2923423 |
| PPP1R11 | chr6_mann_hap4 | 1333523 | 1336701 |
| MICA | chr6_mann_hap4 | 2713996 | 2817998 |
| TUBB | chr6_qbl_hap6 | 1982353 | 1986127 |
| HSPA1B | chr6_qbl_hap6 | 3089162 | 3091686 |
| ZNRD1-AS1 | chr6_qbl_hap6 | 1261701 | 1321962 |
| ZNRD1 | chr6_qbl_hap6 | 1322036 | 1325666 |
| GPANK1 | chr6_qbl_hap6 | 2922642 | 2927703 |
| MSH5 | chr6_qbl_hap6 | 3016759 | 3023034 |
| C6orf10 | chr6_qbl_hap6 | 3521693 | 3600524 |
| RXRB | chr6_qbl_hap6 | 4393583 | 4400696 |
| BRD2 | chr6_ssto_hap7 | 4369432 | 4430060 |
| ZFAND2A | chr7 | 1192542 | 1199855 |
| EIF3B | chr7 | 2394473 | 2420377 |
| TTYH3 | chr7 | 2671602 | 2704436 |
| FOXK1 | chr7 | 4721929 | 4811074 |
| TNRC18 | chr7 | 5346422 | 5463177 |
| ACTB | chr7 | 5566778 | 5569294 |
| FSCN1 | chr7 | 5632435 | 5646287 |
| RAC1 | chr7 | 6414125 | 6443598 |
| KDELR2 | chr7 | 6500711 | 6523849 |
| MEOX2 | chr7 | 15650836 | 15726308 |
| BZW2 | chr7 | 16700838 | 16727179 |
| IGF2BP3 | chr7 | 23349827 | 23509995 |
| TRA2A | chr7 | 23544400 | 23571656 |
| HNRNPA2B1 | chr7 | 26229555 | 26240413 |
| CBX3 | chr7 | 26240830 | 26253227 |
| HOTAIRM1 | chr7 | 27135712 | 27139877 |
| HOXA2 | chr7 | 27139972 | 27142394 |
| HOXA3 | chr7 | 27145808 | 27159214 |
| HOXA5 | chr7 | 27180670 | 27183287 |
| HOXA6 | chr7 | 27185201 | 27187393 |
| HOXA-AS3 | chr7 | 27186781 | 27195547 |
| HOXA7 | chr7 | 27193337 | 27196296 |
| HOXA10-HOXA9 | chr7 | 27202056 | 27219880 |
| HOXA9 | chr7 | 27202056 | 27205149 |
| HOXA-AS4 | chr7 | 27208517 | 27211534 |
| MIR196B | chr7 | 27209098 | 27209182 |
| HOXA10 | chr7 | 27210209 | 27213955 |
| HOXA11 | chr7 | 27220775 | 27224835 |
| NOD1 | chr7 | 30464142 | 30518393 |
| GARS | chr7 | 30634180 | 30673648 |
| KBTBD2 | chr7 | 32907777 | 32931468 |
| 7-Sep | chr7 | 35840595 | 35946715 |
| ANLN | chr7 | 36429431 | 36493400 |
| STARD3NL | chr7 | 38217807 | 38270272 |
| RALA | chr7 | 39663151 | 39747723 |
| CDK13 | chr7 | 39989958 | 40136733 |
| PSMA2 | chr7 | 42956461 | 42971805 |
| STK17A | chr7 | 43622691 | 43666978 |
| PPIA | chr7 | 44836240 | 44842716 |
| H2AFV | chr7 | 44866487 | 44887725 |
| PURB | chr7 | 44915891 | 44924960 |
| SNHG15 | chr7 | 45022626 | 45026259 |
| GRB10 | chr7 | 50657759 | 50860666 |
| SEC61G | chr7 | 54819939 | 54826939 |
| VOPP1 | chr7 | 55538305 | 55640200 |
| CCT6A | chr7 | 56119377 | 56131682 |
| TMEM248 | chr7 | 66386202 | 66423538 |
| BAZ1B | chr7 | 72854727 | 72936615 |
| EIF4H | chr7 | 73588705 | 73611429 |
| GTF2I | chr7 | 74072029 | 74175022 |
| YWHAG | chr7 | 75956107 | 75988342 |
| PTPN12 | chr7 | 77166772 | 77269388 |
| DMTF1 | chr7 | 86781869 | 86825648 |
| DBF4 | chr7 | 87505543 | 87538856 |
| CYP51A1 | chr7 | 91741462 | 91808845 |
| FAM133B | chr7 | 92190071 | 92219706 |
| GNG11 | chr7 | 93551015 | 93555826 |
| BRI3 | chr7 | 97910978 | 97920839 |
| ARPC1A | chr7 | 98923495 | 98963885 |
| PDAP1 | chr7 | 98992297 | 99006305 |
| ATP5J2-PTCD1 | chr7 | 99014361 | 99063824 |
| ATP5J2 | chr7 | 99055783 | 99063824 |
| ZKSCAN1 | chr7 | 99613218 | 99635403 |
| EPHB4 | chr7 | 100400186 | 100423148 |
| SRRT | chr7 | 100472700 | 100486285 |
| SERPINE1 | chr7 | 100770369 | 100782547 |
| POLR2J3 | chr7 | 102178365 | 102213068 |
| LOC100216545 | chr7 | 104650988 | 104654588 |
| KMT2E | chr7 | 104654636 | 104754532 |
| NAMPT | chr7 | 105888731 | 105925638 |
| CBLL1 | chr7 | 107384278 | 107402083 |
| LAMB1 | chr7 | 107564245 | 107643804 |
| NRCAM | chr7 | 107788070 | 108096841 |
| DOCK4 | chr7 | 111366163 | 111846462 |
| IFRD1 | chr7 | 112090482 | 112117258 |
| TES | chr7 | 115850546 | 115898837 |
| CAV1 | chr7 | 115929905 | 116201239 |
| CAV2 | chr7 | 116139654 | 116148595 |
| CAPZA2 | chr7 | 116502562 | 116559313 |
| WASL | chr7 | 123321996 | 123389116 |
| SND1 | chr7 | 127292201 | 127732659 |
| CALU | chr7 | 128379345 | 128413477 |
| UBE2H | chr7 | 129470572 | 129592800 |
| LOC646329 | chr7 | 130565750 | 130598069 |
| CALD1 | chr7 | 134464163 | 134655480 |
| LUZP6 | chr7 | 135611502 | 135662204 |
| CREB3L2 | chr7 | 137559724 | 137686847 |
| LUC7L2 | chr7 | 139044591 | 139108203 |
| HIPK2 | chr7 | 139246315 | 139477693 |
| ZYX | chr7 | 143078359 | 143088206 |
| CUL1 | chr7 | 148395005 | 148498202 |
| PDIA4 | chr7 | 148700153 | 148725782 |
| GIMAP4 | chr7 | 150264457 | 150271041 |
| AGAP3 | chr7 | 150783825 | 150841523 |
| RHEB | chr7 | 151163097 | 151217010 |
| INSIG1 | chr7 | 155089485 | 155101945 |
| RBM33 | chr7 | 155437202 | 155574179 |
| UBE3C | chr7 | 156931654 | 157062066 |
| DNAJB6 | chr7 | 157129709 | 157210133 |
| NCAPG2 | chr7 | 158432487 | 158497520 |
| PDGFA | chr7 | 536896 | 559481 |
| SUN1 | chr7 | 872141 | 914557 |
| GET4 | chr7 | 891586 | 936071 |
| COX19 | chr7 | 1004485 | 1015235 |
| MICALL2 | chr7 | 1473994 | 1499109 |
| INTS1 | chr7 | 1509912 | 1544018 |
| MAFK | chr7 | 1570367 | 1582679 |
| MAD1L1 | chr7 | 1937324 | 2272583 |
| GNA12 | chr7 | 2767740 | 2883959 |
| AP5Z1 | chr7 | 4815261 | 4834026 |
| WIPI2 | chr7 | 5229834 | 5273486 |
| FBXL18 | chr7 | 5515427 | 5553399 |
| RNF216 | chr7 | 5659671 | 5821361 |
| AIMP2 | chr7 | 6048881 | 6063465 |
| EIF2AK1 | chr7 | 6061877 | 6098860 |
| USP42 | chr7 | 6150755 | 6201195 |
| DAGLB | chr7 | 6448746 | 6487643 |
| ZNF12 | chr7 | 6728063 | 6746566 |
| C1GALT1 | chr7 | 7222245 | 7288280 |
| MIOS | chr7 | 7606615 | 7647110 |
| NDUFA4 | chr7 | 10971579 | 10979813 |
| ARL4A | chr7 | 12726910 | 12730558 |
| ETV1 | chr7 | 13930855 | 14029642 |
| ANKMY2 | chr7 | 16639400 | 16685442 |
| AHR | chr7 | 17338275 | 17385775 |
| SNX13 | chr7 | 17830384 | 17980131 |
| HDAC9 | chr7 | 18548899 | 18708466 |
| TWISTNB | chr7 | 19735084 | 19748660 |
| MIR3146 | chr7 | 19744980 | 19745059 |
| ITGB8 | chr7 | 20370324 | 20455382 |
| CDCA7L | chr7 | 21940516 | 21985542 |
| STEAP1B | chr7 | 22478037 | 22539901 |
| LOC100506178 | chr7 | 22602955 | 22613617 |
| IL6 | chr7 | 22766818 | 22770157 |
| TOMM7 | chr7 | 22852250 | 22862471 |
| SNORD93 | chr7 | 22896231 | 22896305 |
| FAM126A | chr7 | 22980877 | 23053770 |
| KLHL7-AS1 | chr7 | 23140846 | 23145322 |
| KLHL7 | chr7 | 23145352 | 23215038 |
| NUPL2 | chr7 | 23234021 | 23240630 |
| MALSU1 | chr7 | 23338939 | 23349180 |
| OSBPL3 | chr7 | 24836163 | 24932240 |
| CYCS | chr7 | 25158269 | 25164980 |
| NFE2L3 | chr7 | 26191846 | 26226756 |
| SKAP2 | chr7 | 26706687 | 26904341 |
| HOXA1 | chr7 | 27132931 | 27135531 |
| HOXA3 | chr7 | 27145808 | 27166639 |
| HOXA4 | chr7 | 27168125 | 27170399 |
| HOXA11-AS | chr7 | 27225026 | 27228912 |
| TAX1BP1 | chr7 | 27779713 | 27869386 |
| CREB5 | chr7 | 28725720 | 28865511 |
| SCRN1 | chr7 | 29959718 | 30008887 |
| FKBP14 | chr7 | 30050198 | 30066417 |
| PLEKHA8 | chr7 | 30067976 | 30124278 |
| GGCT | chr7 | 30536236 | 30544457 |
| LSM5 | chr7 | 32524944 | 32530475 |
| AVL9 | chr7 | 32535175 | 32623779 |
| RP9P | chr7 | 32956426 | 32982782 |
| FKBP9 | chr7 | 32997004 | 33046543 |
| NT5C3A | chr7 | 33053724 | 33102409 |
| RP9 | chr7 | 33134409 | 33149002 |
| DPY19L1 | chr7 | 34968492 | 35077653 |
| HERPUD2 | chr7 | 35672269 | 35734745 |
| KIAA0895 | chr7 | 36363758 | 36429734 |
| MPLKIP | chr7 | 40172341 | 40174251 |
| C7orf10 | chr7 | 40174574 | 40900366 |
| SUGCT | chr7 | 40174574 | 40900366 |
| INHBA | chr7 | 41728600 | 41742706 |
| INHBA-AS1 | chr7 | 41733513 | 41818976 |
| MRPL32 | chr7 | 42971938 | 42977453 |
| COA1 | chr7 | 43670750 | 43769140 |
| POLR2J4 | chr7 | 43980493 | 44058748 |
| DBNL | chr7 | 44084238 | 44101315 |
| POLD2 | chr7 | 44154278 | 44163107 |
| YKT6 | chr7 | 44240577 | 44253893 |
| NUDCD3 | chr7 | 44421964 | 44530385 |
| DDX56 | chr7 | 44605015 | 44614137 |
| TMED4 | chr7 | 44619495 | 44621827 |
| OGDH | chr7 | 44646120 | 44748669 |
| ZMIZ2 | chr7 | 44788529 | 44809479 |
| MIR4657 | chr7 | 44921346 | 44921399 |
| SNORA9 | chr7 | 45024976 | 45025109 |
| CCM2 | chr7 | 45039786 | 45116069 |
| TBRG4 | chr7 | 45139698 | 45151346 |
| SEPT7P2 | chr7 | 45763385 | 45808617 |
| UPP1 | chr7 | 48128745 | 48148330 |
| FIGNL1 | chr7 | 50511831 | 50518088 |
| GRB10 | chr7 | 50657759 | 50773020 |
| LANCL2 | chr7 | 55433140 | 55501435 |
| PSPH | chr7 | 56078743 | 56119268 |
| SUMF2 | chr7 | 56131916 | 56148365 |
| CHCHD2 | chr7 | 56169265 | 56174187 |
| ZNF107 | chr7 | 64126510 | 64171401 |
| ERV3-1 | chr7 | 64450732 | 64467124 |
| ZNF92 | chr7 | 64838767 | 64865998 |
| CCT6P1 | chr7 | 65216091 | 65228662 |
| VKORC1L1 | chr7 | 65338256 | 65419800 |
| GUSB | chr7 | 65425672 | 65447301 |
| CRCP | chr7 | 65579804 | 65619553 |
| LOC493754 | chr7 | 66018552 | 66043498 |
| RABGEF1 | chr7 | 66204982 | 66276448 |
| SBDS | chr7 | 66452689 | 66460588 |
| TYW1 | chr7 | 66461791 | 66704507 |
| STAG3L4 | chr7 | 66767624 | 66786513 |
| TYW1B | chr7 | 72039491 | 72298813 |
| SBDSP1 | chr7 | 72299951 | 72307978 |
| POM121 | chr7 | 72349935 | 72421979 |
| STAG3L3 | chr7 | 72467988 | 72476448 |
| PMS2L2 | chr7 | 72476588 | 72519691 |
| BCL7B | chr7 | 72950682 | 72954898 |
| TBL2 | chr7 | 72983276 | 72993013 |
| DNAJC30 | chr7 | 73095247 | 73097781 |
| WBSCR22 | chr7 | 73097897 | 73112551 |
| STX1A | chr7 | 73113534 | 73134017 |
| MIR590 | chr7 | 73605527 | 73605624 |
| RFC2 | chr7 | 73645831 | 73668738 |
| STAG3L2 | chr7 | 74298262 | 74306731 |
| PMS2P5 | chr7 | 74306893 | 74322330 |
| STAG3L1 | chr7 | 74988446 | 74997085 |
| POM121C | chr7 | 75046064 | 75115568 |
| PMS2P3 | chr7 | 75137068 | 75157453 |
| STYXL1 | chr7 | 75627862 | 75677301 |
| MDH2 | chr7 | 75677392 | 75695930 |
| HSPB1 | chr7 | 75931874 | 75933614 |
| LOC100133091 | chr7 | 76178657 | 76257299 |
| POMZP3 | chr7 | 76239302 | 76256620 |
| RSBN1L-AS1 | chr7 | 77313167 | 77326662 |
| RSBN1L | chr7 | 77325742 | 77409120 |
| TMEM60 | chr7 | 77423044 | 77427747 |
| PHTF2 | chr7 | 77428108 | 77586821 |
| GNAI1 | chr7 | 79765070 | 79848725 |
| TMEM243 | chr7 | 86825477 | 86849031 |
| SLC25A40 | chr7 | 87463813 | 87505692 |
| SRI | chr7 | 87834431 | 87849399 |
| STEAP1 | chr7 | 89783688 | 89794141 |
| GTPBP10 | chr7 | 89975978 | 90020769 |
| AKAP9 | chr7 | 91570188 | 91739987 |
| CYP51A1 | chr7 | 91741462 | 91763840 |
| KRIT1 | chr7 | 91828282 | 91875414 |
| ANKIB1 | chr7 | 91875547 | 92030698 |
| PEX1 | chr7 | 92116336 | 92157845 |
| RBM48 | chr7 | 92158086 | 92166823 |
| CDK6 | chr7 | 92234234 | 92465941 |
| BET1 | chr7 | 93620999 | 93633690 |
| PON2 | chr7 | 95034173 | 95064384 |
| SHFM1 | chr7 | 96302236 | 96339203 |
| TECPR1 | chr7 | 97844754 | 97881563 |
| TRRAP | chr7 | 98476112 | 98610866 |
| MIR3609 | chr7 | 98479272 | 98479352 |
| ARPC1B | chr7 | 98972297 | 98992404 |
| BUD31 | chr7 | 99006600 | 99015494 |
| PTCD1 | chr7 | 99014361 | 99036462 |
| CPSF4 | chr7 | 99036562 | 99054996 |
| ZNF789 | chr7 | 99081245 | 99085217 |
| ZNF394 | chr7 | 99090853 | 99097877 |
| ZKSCAN5 | chr7 | 99102572 | 99131445 |
| TRIM4 | chr7 | 99488029 | 99517223 |
| ZNF3 | chr7 | 99667593 | 99679387 |
| COPS6 | chr7 | 99686582 | 99689822 |
| MCM7 | chr7 | 99690403 | 99698380 |
| MIR25 | chr7 | 99691182 | 99691266 |
| MIR93 | chr7 | 99691390 | 99691470 |
| MIR106B | chr7 | 99691615 | 99691697 |
| AP4M1 | chr7 | 99699129 | 99704803 |
| PMS2P1 | chr7 | 99918262 | 99933930 |
| STAG3L5P-PVRIG2P-PILRB | chr7 | 99933687 | 99965454 |
| ZCWPW1 | chr7 | 99998494 | 100026431 |
| MEPCE | chr7 | 100027253 | 100031749 |
| PPP1R35 | chr7 | 100032911 | 100034094 |
| TSC22D4 | chr7 | 100064141 | 100076902 |
| LRCH4 | chr7 | 100171633 | 100183776 |
| GNB2 | chr7 | 100271362 | 100276792 |
| POP7 | chr7 | 100303675 | 100305123 |
| TRIP6 | chr7 | 100464949 | 100471076 |
| UFSP1 | chr7 | 100486343 | 100487339 |
| TRIM56 | chr7 | 100728785 | 100733889 |
| PLOD3 | chr7 | 100849257 | 100861011 |
| ZNHIT1 | chr7 | 100860984 | 100867471 |
| FIS1 | chr7 | 100882892 | 100888371 |
| LOC100630923 | chr7 | 102004307 | 102067129 |
| LOC100289561 | chr7 | 102004329 | 102036769 |
| PRKRIP1 | chr7 | 102036803 | 102067129 |
| ORAI2 | chr7 | 102073976 | 102097268 |
| POLR2J | chr7 | 102113547 | 102119381 |
| POLR2J2 | chr7 | 102277194 | 102312176 |
| ARMC10 | chr7 | 102715327 | 102740210 |
| NAPEPLD | chr7 | 102740022 | 102789569 |
| DNAJC2 | chr7 | 102952920 | 102985320 |
| PSMC2 | chr7 | 102987970 | 103009842 |
| ORC5 | chr7 | 103766787 | 103848495 |
| LOC100216546 | chr7 | 104622193 | 104631612 |
| SRPK2 | chr7 | 104756820 | 105029377 |
| PUS7 | chr7 | 105096959 | 105162685 |
| RINT1 | chr7 | 105172531 | 105208124 |
| SYPL1 | chr7 | 105730813 | 105753093 |
| CCDC71L | chr7 | 106297210 | 106301634 |
| PRKAR2B | chr7 | 106685177 | 106802256 |
| HBP1 | chr7 | 106810217 | 106842974 |
| COG5 | chr7 | 106848297 | 107204959 |
| DUS4L | chr7 | 107204401 | 107218968 |
| BCAP29 | chr7 | 107220421 | 107263762 |
| DLD | chr7 | 107531585 | 107561643 |
| PNPLA8 | chr7 | 108110865 | 108166762 |
| THAP5 | chr7 | 108202670 | 108209897 |
| DNAJB9 | chr7 | 108210188 | 108215294 |
| ZNF277 | chr7 | 111846642 | 111983989 |
| IFRD1 | chr7 | 112092112 | 112117258 |
| TMEM168 | chr7 | 112405786 | 112430478 |
| TES | chr7 | 115863004 | 115898837 |
| MET | chr7 | 116312458 | 116438440 |
| ST7-AS1 | chr7 | 116592500 | 116594388 |
| ST7 | chr7 | 116593380 | 116863961 |
| ST7-OT4 | chr7 | 116593952 | 116599867 |
| NAA38 | chr7 | 117824085 | 117844093 |
| ING3 | chr7 | 120590816 | 120615711 |
| FAM3C | chr7 | 120988904 | 121036422 |
| AASS | chr7 | 121713597 | 121784344 |
| NDUFA5 | chr7 | 123181082 | 123197958 |
| POT1 | chr7 | 124462439 | 124570037 |
| ZNF800 | chr7 | 127010353 | 127032767 |
| GCC1 | chr7 | 127220681 | 127225654 |
| RBM28 | chr7 | 127950435 | 127983962 |
| IMPDH1 | chr7 | 128032330 | 128050036 |
| HILPDA | chr7 | 128095883 | 128098472 |
| METTL2B | chr7 | 128116782 | 128142978 |
| FLNC | chr7 | 128470482 | 128499328 |
| ATP6V1F | chr7 | 128502856 | 128505903 |
| TNPO3 | chr7 | 128594233 | 128695227 |
| TPI1P2 | chr7 | 128695276 | 128697293 |
| NRF1 | chr7 | 129251554 | 129396922 |
| KLHDC10 | chr7 | 129710348 | 129775560 |
| TMEM209 | chr7 | 129804554 | 129845338 |
| MIR29A | chr7 | 130561505 | 130561569 |
| MIR29B1 | chr7 | 130562217 | 130562298 |
| FLJ43663 | chr7 | 130628918 | 130793562 |
| MKLN1 | chr7 | 130794854 | 131181398 |
| PODXL | chr7 | 131185020 | 131241376 |
| CHCHD3 | chr7 | 132469622 | 132766828 |
| BPGM | chr7 | 134331530 | 134364567 |
| CALD1 | chr7 | 134576150 | 134655480 |
| C7orf49 | chr7 | 134850531 | 134855578 |
| CNOT4 | chr7 | 135071821 | 135194875 |
| NUP205 | chr7 | 135242661 | 135333499 |
| C7orf73 | chr7 | 135347220 | 135361160 |
| LOC100130880 | chr7 | 137638093 | 137642712 |
| TRIM24 | chr7 | 138145078 | 138270332 |
| ZC3HAV1 | chr7 | 138728265 | 138794465 |
| C7orf55-LUC7L2 | chr7 | 139025877 | 139108203 |
| C7orf55 | chr7 | 139025877 | 139031065 |
| LUC7L2 | chr7 | 139059418 | 139108203 |
| TBXAS1 | chr7 | 139478046 | 139720125 |
| PARP12 | chr7 | 139723548 | 139763521 |
| NDUFB2 | chr7 | 140396480 | 140406446 |
| BRAF | chr7 | 140433812 | 140624564 |
| MRPS33 | chr7 | 140705960 | 140714479 |
| WEE2-AS1 | chr7 | 141404137 | 141438030 |
| SSBP1 | chr7 | 141438120 | 141446326 |
| CASP2 | chr7 | 142985307 | 143004789 |
| FAM115C | chr7 | 143318044 | 143422176 |
| LOC154761 | chr7 | 143339348 | 143533810 |
| FAM115A | chr7 | 143548460 | 143599278 |
| EZH2 | chr7 | 148504463 | 148581441 |
| ZNF425 | chr7 | 148799877 | 148823438 |
| ZNF398 | chr7 | 148823507 | 148880118 |
| ZNF282 | chr7 | 148892576 | 148923339 |
| ZNF746 | chr7 | 149169883 | 149194898 |
| ZNF767 | chr7 | 149244244 | 149321881 |
| RNU6-34P | chr7 | 150064035 | 150064063 |
| RNU6-33P | chr7 | 150064035 | 150064063 |
| REPIN1 | chr7 | 150065878 | 150071133 |
| ZNF775 | chr7 | 150076405 | 150095719 |
| GIMAP8 | chr7 | 150147961 | 150176483 |
| GIMAP7 | chr7 | 150211944 | 150218161 |
| GIMAP6 | chr7 | 150322906 | 150329736 |
| GIMAP2 | chr7 | 150382793 | 150390728 |
| GIMAP1 | chr7 | 150413644 | 150421368 |
| GIMAP1-GIMAP5 | chr7 | 150413644 | 150440737 |
| CDK5 | chr7 | 150750898 | 150755052 |
| SLC4A2 | chr7 | 150755298 | 150773614 |
| FASTK | chr7 | 150773707 | 150777970 |
| TMUB1 | chr7 | 150778171 | 150780413 |
| ABCF2 | chr7 | 150909575 | 150924317 |
| CHPF2 | chr7 | 150929584 | 150935905 |
| PRKAG2 | chr7 | 151253209 | 151292537 |
| KMT2C | chr7 | 151832009 | 152133090 |
| XRCC2 | chr7 | 152343586 | 152373250 |
| PAXIP1 | chr7 | 154735399 | 154794682 |
| LOC202781 | chr7 | 154795142 | 154797413 |
| LMBR1 | chr7 | 156473569 | 156685902 |
| NOM1 | chr7 | 156742416 | 156765876 |
| ESYT2 | chr7 | 158523688 | 158622319 |
| ANGPT2 | chr8 | 6357174 | 6420784 |
| PPP1R3B | chr8 | 8993763 | 9009152 |
| SOX7 | chr8 | 10581277 | 10697299 |
| FDFT1 | chr8 | 11660520 | 11696818 |
| CTSB | chr8 | 11700033 | 11725646 |
| DLC1 | chr8 | 12940871 | 12990809 |
| MTUS1 | chr8 | 17501302 | 17555246 |
| TNFRSF10B | chr8 | 22877647 | 22926700 |
| TNFRSF10D | chr8 | 22993100 | 23021543 |
| LOXL2 | chr8 | 23154409 | 23261722 |
| SLC25A37 | chr8 | 23386362 | 23430063 |
| PPP2R2A | chr8 | 26149006 | 26230195 |
| DUSP4 | chr8 | 29190578 | 29206322 |
| PPP2CB | chr8 | 30643125 | 30670352 |
| NRG1 | chr8 | 32405727 | 32622558 |
| WHSC1L1 | chr8 | 38132560 | 38239790 |
| TACC1 | chr8 | 38585703 | 38710546 |
| ADAM9 | chr8 | 38854504 | 38962779 |
| KAT6A | chr8 | 41786996 | 41909505 |
| HOOK3 | chr8 | 42752032 | 42885682 |
| FNTA | chr8 | 42911441 | 42940932 |
| SDCBP | chr8 | 59465727 | 59495419 |
| RAB2A | chr8 | 61429468 | 61536203 |
| YTHDF3 | chr8 | 64081111 | 64125346 |
| VCPIP1 | chr8 | 67542487 | 67579452 |
| PREX2 | chr8 | 68864602 | 69143897 |
| TCEB1 | chr8 | 74857372 | 74884522 |
| PKIA | chr8 | 79428335 | 79517502 |
| ZBTB10 | chr8 | 81398447 | 81438500 |
| RBM12B | chr8 | 94743730 | 94753224 |
| KIAA1429 | chr8 | 95500004 | 95565746 |
| MTDH | chr8 | 98656406 | 98742488 |
| RNF19A | chr8 | 101269287 | 101322379 |
| PABPC1 | chr8 | 101715143 | 101734315 |
| YWHAZ | chr8 | 101930803 | 101964357 |
| UBR5 | chr8 | 103264501 | 103424917 |
| AZIN1 | chr8 | 103838535 | 103876397 |
| SLC25A32 | chr8 | 104410865 | 104427563 |
| EIF3E | chr8 | 109213971 | 109255062 |
| NUDCD1 | chr8 | 110253147 | 110346350 |
| RAD21 | chr8 | 117858172 | 117887105 |
| MIR3610 | chr8 | 117886966 | 117887039 |
| EXT1 | chr8 | 118811601 | 119124058 |
| DERL1 | chr8 | 124026388 | 124054663 |
| FAM91A1 | chr8 | 124780881 | 124827690 |
| RNF139 | chr8 | 125487007 | 125500859 |
| SQLE | chr8 | 126010719 | 126034525 |
| TRIB1 | chr8 | 126442562 | 126450644 |
| MYC | chr8 | 128748314 | 128753680 |
| MIR1204 | chr8 | 128808207 | 128808274 |
| PVT1 | chr8 | 128902834 | 129113499 |
| ASAP1 | chr8 | 131064350 | 131455906 |
| ST3GAL1 | chr8 | 134467090 | 134584183 |
| AGO2 | chr8 | 141541263 | 141645646 |
| PTK2 | chr8 | 141668480 | 142011412 |
| DENND3 | chr8 | 142138719 | 142205900 |
| EEF1D | chr8 | 144661866 | 144679845 |
| PUF60 | chr8 | 144899079 | 144905452 |
| HSF1 | chr8 | 145515269 | 145538385 |
| RPL8 | chr8 | 146015153 | 146017729 |
| ZNF596 | chr8 | 182383 | 197339 |
| CLN8 | chr8 | 1711869 | 1734736 |
| ARHGEF10 | chr8 | 1772148 | 1906807 |
| MCPH1 | chr8 | 6264112 | 6304940 |
| AGPAT5 | chr8 | 6565877 | 6619021 |
| SGK223 | chr8 | 8175257 | 8239257 |
| ERI1 | chr8 | 8860313 | 8890849 |
| TNKS | chr8 | 9413444 | 9639856 |
| PINX1 | chr8 | 10622883 | 10697299 |
| MTMR9 | chr8 | 11141999 | 11185654 |
| DLC1 | chr8 | 12940871 | 13372429 |
| ZDHHC2 | chr8 | 17013835 | 17080241 |
| CNOT7 | chr8 | 17086739 | 17104387 |
| VPS37A | chr8 | 17104400 | 17155533 |
| SLC7A2 | chr8 | 17354596 | 17428077 |
| PCM1 | chr8 | 17780365 | 17887457 |
| PSD3 | chr8 | 18384812 | 18871196 |
| ATP6V1B2 | chr8 | 20054703 | 20079207 |
| XPO7 | chr8 | 21777179 | 21864096 |
| REEP4 | chr8 | 21995532 | 21999448 |
| POLR3D | chr8 | 22102618 | 22108680 |
| SLC39A14 | chr8 | 22225049 | 22280249 |
| PPP3CC | chr8 | 22298482 | 22398657 |
| SORBS3 | chr8 | 22423178 | 22433008 |
| C8orf58 | chr8 | 22457113 | 22461662 |
| CCAR2 | chr8 | 22462144 | 22477983 |
| KIAA1967 | chr8 | 22462538 | 22477983 |
| BIN3 | chr8 | 22477946 | 22526661 |
| LOC286059 | chr8 | 22925741 | 22941132 |
| TNFRSF10A | chr8 | 23048969 | 23082680 |
| LOC100507156 | chr8 | 23193720 | 23223638 |
| ENTPD4 | chr8 | 23286664 | 23315244 |
| KCTD9 | chr8 | 25285363 | 25315984 |
| CDCA2 | chr8 | 25316512 | 25365425 |
| DPYSL2 | chr8 | 26435339 | 26515693 |
| TRIM35 | chr8 | 27142403 | 27168834 |
| CCDC25 | chr8 | 27590832 | 27630170 |
| ESCO2 | chr8 | 27632057 | 27662424 |
| PBK | chr8 | 27667137 | 27695572 |
| ELP3 | chr8 | 27950583 | 28048669 |
| EXTL3 | chr8 | 28558989 | 28611207 |
| INTS9 | chr8 | 28625174 | 28747698 |
| HMBOX1 | chr8 | 28748224 | 28910242 |
| TMEM66 | chr8 | 29920630 | 29940649 |
| LEPROTL1 | chr8 | 29952921 | 29965587 |
| DCTN6 | chr8 | 30013812 | 30041155 |
| GTF2E2 | chr8 | 30436030 | 30515738 |
| GSR | chr8 | 30535579 | 30585486 |
| PURG | chr8 | 30853320 | 30891231 |
| WRN | chr8 | 30890777 | 31031277 |
| FUT10 | chr8 | 33228343 | 33330664 |
| MAK16 | chr8 | 33342684 | 33358778 |
| TTI2 | chr8 | 33356026 | 33370703 |
| ZNF703 | chr8 | 37553300 | 37556396 |
| PROSC | chr8 | 37620100 | 37637286 |
| BRF2 | chr8 | 37701397 | 37707431 |
| EIF4EBP1 | chr8 | 37888019 | 37917883 |
| ASH2L | chr8 | 37963310 | 37997598 |
| LSM1 | chr8 | 38020838 | 38034248 |
| BAG4 | chr8 | 38034105 | 38070819 |
| DDHD2 | chr8 | 38089008 | 38120287 |
| PPAPDC1B | chr8 | 38120649 | 38126658 |
| LETM2 | chr8 | 38243958 | 38251951 |
| FGFR1 | chr8 | 38268655 | 38326352 |
| C8orf86 | chr8 | 38368351 | 38386180 |
| RNF5P1 | chr8 | 38457692 | 38458775 |
| TM2D2 | chr8 | 38846326 | 38854041 |
| C8orf4 | chr8 | 40010986 | 40012827 |
| GOLGA7 | chr8 | 41348172 | 41368499 |
| AGPAT6 | chr8 | 41435706 | 41482520 |
| AP3M2 | chr8 | 42010463 | 42028701 |
| PLAT | chr8 | 42032235 | 42065194 |
| IKBKB | chr8 | 42128819 | 42190171 |
| VDAC3 | chr8 | 42249278 | 42263455 |
| SLC20A2 | chr8 | 42273979 | 42397356 |
| SMIM19 | chr8 | 42396764 | 42408140 |
| THAP1 | chr8 | 42691816 | 42698474 |
| RNF170 | chr8 | 42708437 | 42751866 |
| MIR4469 | chr8 | 42751339 | 42751418 |
| SGK196 | chr8 | 42948648 | 42978323 |
| PRKDC | chr8 | 48685668 | 48872743 |
| MCM4 | chr8 | 48872762 | 48890719 |
| UBE2V2 | chr8 | 48920994 | 48974454 |
| PCMTD1 | chr8 | 52730139 | 52811735 |
| RB1CC1 | chr8 | 53535017 | 53627026 |
| ATP6V1H | chr8 | 54628102 | 54755871 |
| TCEA1 | chr8 | 54879115 | 54935008 |
| LYPLA1 | chr8 | 54958937 | 55014577 |
| MRPL15 | chr8 | 55047780 | 55061074 |
| SOX17 | chr8 | 55370494 | 55373456 |
| TMEM68 | chr8 | 56651319 | 56675587 |
| TGS1 | chr8 | 56685790 | 56738005 |
| RPS20 | chr8 | 56980738 | 56987140 |
| SNORD54 | chr8 | 56986397 | 56986460 |
| PLAG1 | chr8 | 57073467 | 57123859 |
| CHCHD7 | chr8 | 57124314 | 57131176 |
| IMPAD1 | chr8 | 57870487 | 57906430 |
| NSMAF | chr8 | 59496063 | 59572404 |
| TOX | chr8 | 59717976 | 60031767 |
| ASPH | chr8 | 62413114 | 62627199 |
| MIR4470 | chr8 | 62627346 | 62627418 |
| GGH | chr8 | 63927638 | 63951610 |
| ARMC1 | chr8 | 66514690 | 66546452 |
| PDE7A | chr8 | 66626568 | 66753969 |
| LOC100505676 | chr8 | 67331821 | 67341212 |
| RRS1 | chr8 | 67341262 | 67342968 |
| ADHFE1 | chr8 | 67344717 | 67381044 |
| C8orf44-SGK3 | chr8 | 67579786 | 67774257 |
| C8orf44 | chr8 | 67588453 | 67593377 |
| SNHG6 | chr8 | 67834164 | 67837777 |
| COPS5 | chr8 | 67955314 | 67974562 |
| CSPP1 | chr8 | 67976602 | 68108849 |
| ARFGEF1 | chr8 | 68109883 | 68255912 |
| TRAM1 | chr8 | 71485452 | 71520694 |
| LOC286190 | chr8 | 71520811 | 71575513 |
| RPL7 | chr8 | 74202873 | 74205869 |
| RDH10 | chr8 | 74206836 | 74237520 |
| UBE2W | chr8 | 74698454 | 74791145 |
| TMEM70 | chr8 | 74888376 | 74895018 |
| LY96 | chr8 | 74903563 | 74941307 |
| PEX2 | chr8 | 77892493 | 77913280 |
| MRPS28 | chr8 | 80831094 | 80942506 |
| FABP5 | chr8 | 82192717 | 82197012 |
| ZFAND1 | chr8 | 82613565 | 82633539 |
| WWP1 | chr8 | 87354993 | 87480178 |
| MMP16 | chr8 | 89049459 | 89339717 |
| RIPK2 | chr8 | 90769974 | 90803292 |
| OSGIN2 | chr8 | 90914761 | 90940095 |
| NBN | chr8 | 90945563 | 90996899 |
| DECR1 | chr8 | 91013579 | 91064227 |
| TMEM64 | chr8 | 91634222 | 91658133 |
| OTUD6B | chr8 | 92082423 | 92099323 |
| RUNX1T1 | chr8 | 92967194 | 93107882 |
| RBM12B-AS1 | chr8 | 94752338 | 94753047 |
| PDP1 | chr8 | 94929174 | 94938296 |
| RAD54B | chr8 | 95439939 | 95487343 |
| DPY19L4 | chr8 | 95732102 | 95806076 |
| TP53INP1 | chr8 | 95938199 | 95961615 |
| PLEKHF2 | chr8 | 96145948 | 96168913 |
| UQCRB | chr8 | 97238903 | 97247862 |
| MTERFD1 | chr8 | 97251644 | 97273796 |
| PTDSS1 | chr8 | 97274166 | 97346774 |
| RPL30 | chr8 | 99037078 | 99057818 |
| SNORA72 | chr8 | 99054313 | 99054445 |
| HRSP12 | chr8 | 99114566 | 99129418 |
| POP1 | chr8 | 99129520 | 99172069 |
| VPS13B | chr8 | 100025493 | 100889814 |
| COX6C | chr8 | 100890222 | 100906242 |
| POLR2K | chr8 | 101162838 | 101166230 |
| RNF19A | chr8 | 101269287 | 101315487 |
| ZNF706 | chr8 | 102209265 | 102217960 |
| RRM2B | chr8 | 103216728 | 103251346 |
| KLF10 | chr8 | 103661004 | 103666168 |
| ATP6V1C1 | chr8 | 104033247 | 104085285 |
| DCAF13 | chr8 | 104426941 | 104455680 |
| ZFPM2 | chr8 | 106331146 | 106816767 |
| OXR1 | chr8 | 107670035 | 107764921 |
| EMC2 | chr8 | 109455852 | 109499136 |
| NUDCD1 | chr8 | 110253147 | 110342162 |
| ENY2 | chr8 | 110346551 | 110358189 |
| EIF3H | chr8 | 117657054 | 117778494 |
| UTP23 | chr8 | 117778741 | 117786921 |
| RAD21-AS1 | chr8 | 117886662 | 117889107 |
| TAF2 | chr8 | 120743013 | 120845074 |
| MRPL13 | chr8 | 121408082 | 121457647 |
| MTBP | chr8 | 121457637 | 121535875 |
| C8orf76 | chr8 | 124232195 | 124253638 |
| ATAD2 | chr8 | 124332090 | 124408705 |
| TMEM65 | chr8 | 125323158 | 125384940 |
| TRMT12 | chr8 | 125463047 | 125465266 |
| TATDN1 | chr8 | 125500734 | 125551329 |
| NDUFB9 | chr8 | 125551342 | 125580751 |
| MTSS1 | chr8 | 125563027 | 125740730 |
| KIAA0196 | chr8 | 126036502 | 126104061 |
| NSMCE2 | chr8 | 126104082 | 126379367 |
| MIR1205 | chr8 | 128972878 | 128972941 |
| MIR1207 | chr8 | 129061397 | 129061484 |
| ASAP1-IT1 | chr8 | 131307600 | 131308779 |
| EFR3A | chr8 | 132916355 | 133025886 |
| PHF20L1 | chr8 | 133787603 | 133861052 |
| CHRAC1 | chr8 | 141521396 | 141527252 |
| JRK | chr8 | 143738873 | 143751401 |
| PSCA | chr8 | 143751725 | 143764145 |
| ZC3H3 | chr8 | 144519824 | 144623620 |
| TIGD5 | chr8 | 144680073 | 144682485 |
| ZNF623 | chr8 | 144718331 | 144735900 |
| SCRIB | chr8 | 144873089 | 144897549 |
| PLEC | chr8 | 144989320 | 145025044 |
| MIR661 | chr8 | 145019358 | 145019447 |
| CYC1 | chr8 | 145149959 | 145152428 |
| SHARPIN | chr8 | 145153535 | 145159140 |
| MAF1 | chr8 | 145159304 | 145162515 |
| BOP1 | chr8 | 145486055 | 145515120 |
| CPSF1 | chr8 | 145618445 | 145634733 |
| VPS28 | chr8 | 145648999 | 145653927 |
| LRRC14 | chr8 | 145743348 | 145750559 |
| ZNF251 | chr8 | 145946293 | 145980970 |
| ZNF34 | chr8 | 145998500 | 146012725 |
| ZNF7 | chr8 | 146059117 | 146068606 |
| ZNF250 | chr8 | 146102335 | 146126846 |
| C8orf33 | chr8 | 146277823 | 146281416 |
| KIAA0020 | chr9 | 2804154 | 2844130 |
| CD274 | chr9 | 5450502 | 5470567 |
| UHRF2 | chr9 | 6413150 | 6507051 |
| NFIB | chr9 | 14081841 | 14314045 |
| RPS6 | chr9 | 19376253 | 19380235 |
| KLHL9 | chr9 | 21331017 | 21335429 |
| MIR31HG | chr9 | 21454266 | 21559697 |
| PLAA | chr9 | 26903367 | 26947468 |
| DNAJA1 | chr9 | 33025208 | 33039062 |
| B4GALT1 | chr9 | 33110638 | 33167356 |
| ANXA2P2 | chr9 | 33624222 | 33625532 |
| UBE2R2 | chr9 | 33817181 | 33920401 |
| UBAP2 | chr9 | 33921690 | 34048947 |
| VCP | chr9 | 35056064 | 35072739 |
| RUSC2 | chr9 | 35490006 | 35561895 |
| TLN1 | chr9 | 35697333 | 35732392 |
| GBA2 | chr9 | 35736862 | 35749225 |
| CLTA | chr9 | 36190852 | 36212059 |
| MELK | chr9 | 36572858 | 36677680 |
| TOMM5 | chr9 | 37588411 | 37592636 |
| SHB | chr9 | 37915894 | 38069210 |
| SMC5 | chr9 | 72873877 | 72969789 |
| KLF9 | chr9 | 72999512 | 73029573 |
| ZFAND5 | chr9 | 74966340 | 74980163 |
| ANXA1 | chr9 | 75772569 | 75785307 |
| GNAQ | chr9 | 80335190 | 80646219 |
| UBQLN1 | chr9 | 86274877 | 86323168 |
| HNRNPK | chr9 | 86582997 | 86595569 |
| CKS2 | chr9 | 91926112 | 91931618 |
| MIR3153 | chr9 | 91927139 | 91927221 |
| IARS | chr9 | 94972489 | 95056038 |
| IPPK | chr9 | 95375465 | 95432547 |
| BICD2 | chr9 | 95473644 | 95527083 |
| FAM120A | chr9 | 96214172 | 96328397 |
| HIATL1 | chr9 | 97136832 | 97223202 |
| HIATL2 | chr9 | 99708326 | 99775862 |
| NCBP1 | chr9 | 100395704 | 100436029 |
| ANP32B | chr9 | 100745488 | 100778224 |
| SEC61B | chr9 | 101984569 | 101992901 |
| MSANTD3 | chr9 | 103189494 | 103214016 |
| RAD23B | chr9 | 110045516 | 110094475 |
| PALM2-AKAP2 | chr9 | 112542576 | 112900819 |
| AKAP2 | chr9 | 112810877 | 112934791 |
| TXN | chr9 | 113006091 | 113018920 |
| UGCG | chr9 | 114659205 | 114695433 |
| RGS3 | chr9 | 116225979 | 116360023 |
| ZNF618 | chr9 | 116638561 | 116818875 |
| ATP6V1G1 | chr9 | 117349993 | 117361152 |
| TLR4 | chr9 | 120466452 | 120479769 |
| RAB14 | chr9 | 123940414 | 123964365 |
| RC3H2 | chr9 | 125611731 | 125667562 |
| NEK6 | chr9 | 127020195 | 127114719 |
| MIR181A2HG | chr9 | 127420714 | 127460907 |
| RPL35 | chr9 | 127620157 | 127624240 |
| ARPC5L | chr9 | 127631483 | 127639696 |
| HSPA5 | chr9 | 127997126 | 128003666 |
| RPL12 | chr9 | 130209952 | 130213711 |
| FAM129B | chr9 | 130267622 | 130331355 |
| SH2D3C | chr9 | 130500595 | 130533648 |
| MIR3960 | chr9 | 130548111 | 130548286 |
| CDK9 | chr9 | 130548304 | 130553052 |
| ENG | chr9 | 130577290 | 130617052 |
| SPTAN1 | chr9 | 131314836 | 131395944 |
| SET | chr9 | 131451508 | 131458675 |
| LRRC8A | chr9 | 131644780 | 131680317 |
| NTMT1 | chr9 | 132388434 | 132397879 |
| ABL1 | chr9 | 133710830 | 133763062 |
| RAPGEF1 | chr9 | 134452156 | 134612925 |
| GTF3C4 | chr9 | 135545727 | 135565470 |
| SURF4 | chr9 | 136228339 | 136242970 |
| WDR5 | chr9 | 137001209 | 137025094 |
| CAMSAP1 | chr9 | 138700332 | 138799005 |
| SEC16A | chr9 | 139334547 | 139377507 |
| NOTCH1 | chr9 | 139388895 | 139440238 |
| MIR4674 | chr9 | 139440624 | 139440711 |
| EGFL7 | chr9 | 139553307 | 139567130 |
| SNHG7 | chr9 | 139619683 | 139622636 |
| KANK1 | chr9 | 706805 | 746106 |
| RFX3 | chr9 | 3247036 | 3525983 |
| CDC37L1 | chr9 | 4679565 | 4706594 |
| AK3 | chr9 | 4709556 | 4741309 |
| RCL1 | chr9 | 4792833 | 4861064 |
| JAK2 | chr9 | 4985244 | 5128183 |
| PLGRKT | chr9 | 5357966 | 5630071 |
| PDCD1LG2 | chr9 | 5510544 | 5571282 |
| KIAA1432 | chr9 | 5629118 | 5776556 |
| KIAA2026 | chr9 | 5919007 | 6008003 |
| MIR4665 | chr9 | 6007825 | 6007904 |
| RANBP6 | chr9 | 6011018 | 6015640 |
| KDM4C | chr9 | 6757640 | 7175648 |
| MPDZ | chr9 | 13105702 | 13279563 |
| ZDHHC21 | chr9 | 14611068 | 14693480 |
| SNAPC3 | chr9 | 15422781 | 15461627 |
| PSIP1 | chr9 | 15464064 | 15511003 |
| CNTLN | chr9 | 17135037 | 17503917 |
| ADAMTSL1 | chr9 | 18474078 | 18910947 |
| RRAGA | chr9 | 19049371 | 19051021 |
| HAUS6 | chr9 | 19053134 | 19102940 |
| PLIN2 | chr9 | 19115758 | 19127604 |
| DENND4C | chr9 | 19230762 | 19374137 |
| MTAP | chr9 | 21802634 | 21865969 |
| CAAP1 | chr9 | 26840682 | 26892826 |
| IFT74 | chr9 | 26947309 | 27062931 |
| TEK | chr9 | 27109146 | 27230172 |
| ACO1 | chr9 | 32384600 | 32450832 |
| DDX58 | chr9 | 32455299 | 32502734 |
| TOPORS | chr9 | 32540541 | 32552626 |
| TOPORS-AS1 | chr9 | 32551141 | 32553015 |
| NDUFB6 | chr9 | 32553523 | 32573182 |
| APTX | chr9 | 32972603 | 33001574 |
| SMU1 | chr9 | 33041849 | 33076714 |
| BAG1 | chr9 | 33252469 | 33264759 |
| CHMP5 | chr9 | 33264876 | 33282067 |
| NFX1 | chr9 | 33290417 | 33371155 |
| NOL6 | chr9 | 33461350 | 33473941 |
| SUGT1P1 | chr9 | 33504534 | 33511164 |
| DCAF12 | chr9 | 34086380 | 34126771 |
| UBAP1 | chr9 | 34179002 | 34252521 |
| KIF24 | chr9 | 34252377 | 34329198 |
| NUDT2 | chr9 | 34329503 | 34343711 |
| FAM219A | chr9 | 34398181 | 34458568 |
| SIGMAR1 | chr9 | 34634718 | 34637729 |
| FANCG | chr9 | 35073834 | 35080013 |
| STOML2 | chr9 | 35099888 | 35103154 |
| FAM214B | chr9 | 35104117 | 35111571 |
| UNC13B | chr9 | 35161988 | 35405332 |
| RUSC2 | chr9 | 35538628 | 35561895 |
| CCDC107 | chr9 | 35658286 | 35661500 |
| TPM2 | chr9 | 35682922 | 35690053 |
| CREB3 | chr9 | 35732316 | 35737005 |
| RGP1 | chr9 | 35749276 | 35753264 |
| MSMP | chr9 | 35752987 | 35754274 |
| HRCT1 | chr9 | 35906188 | 35907138 |
| LOC158376 | chr9 | 35909479 | 35911617 |
| RECK | chr9 | 36036909 | 36124452 |
| RNF38 | chr9 | 36336398 | 36400920 |
| ZCCHC7 | chr9 | 37120632 | 37358145 |
| GRHPR | chr9 | 37422706 | 37436986 |
| ZBTB5 | chr9 | 37438099 | 37465407 |
| EXOSC3 | chr9 | 37779710 | 37785089 |
| DCAF10 | chr9 | 37800789 | 37867665 |
| SLC25A51 | chr9 | 37877571 | 37904350 |
| CNTNAP3B | chr9 | 43684884 | 43922473 |
| FAM27C | chr9 | 44990235 | 44991492 |
| FAM27B | chr9 | 67792928 | 67794189 |
| LOC100132352 | chr9 | 68726540 | 68748372 |
| CBWD6 | chr9 | 69204537 | 69262593 |
| CBWD3 | chr9 | 70856838 | 70914932 |
| TJP2 | chr9 | 71788970 | 71870124 |
| PTAR1 | chr9 | 72324437 | 72374876 |
| LOC100507299 | chr9 | 72830974 | 72873790 |
| TMEM2 | chr9 | 74298281 | 74383800 |
| ABHD17B | chr9 | 74480067 | 74526148 |
| C9orf85 | chr9 | 74526422 | 74588371 |
| NMRK1 | chr9 | 77676115 | 77703133 |
| OSTF1 | chr9 | 77703397 | 77762114 |
| RFK | chr9 | 79000432 | 79009444 |
| VPS13A | chr9 | 79792360 | 80032399 |
| TLE4 | chr9 | 82186877 | 82341656 |
| TLE1 | chr9 | 84202837 | 84303596 |
| FRMD3 | chr9 | 85857904 | 85882202 |
| RMI1 | chr9 | 86595636 | 86618987 |
| NAA35 | chr9 | 88556056 | 88637217 |
| GOLM1 | chr9 | 88641057 | 88714510 |
| ISCA1 | chr9 | 88879462 | 88897490 |
| ZCCHC6 | chr9 | 88902647 | 88969402 |
| DAPK1 | chr9 | 90112755 | 90323549 |
| SPIN1 | chr9 | 91003296 | 91093622 |
| C9orf47 | chr9 | 91605777 | 91611057 |
| S1PR3 | chr9 | 91606323 | 91620069 |
| SECISBP2 | chr9 | 91933765 | 91974561 |
| NFIL3 | chr9 | 94171326 | 94186144 |
| SPTLC1 | chr9 | 94814978 | 94877690 |
| SNORA84 | chr9 | 95054739 | 95054875 |
| MIR3651 | chr9 | 95054739 | 95054829 |
| NOL8 | chr9 | 95059639 | 95087876 |
| CENPP | chr9 | 95087740 | 95377437 |
| ZNF484 | chr9 | 95607312 | 95640320 |
| C9orf89 | chr9 | 95858449 | 95875565 |
| FAM120AOS | chr9 | 96208781 | 96215874 |
| MIRLET7A1 | chr9 | 96938238 | 96938318 |
| MIRLET7F1 | chr9 | 96938628 | 96938715 |
| MIRLET7D | chr9 | 96941115 | 96941202 |
| FANCC | chr9 | 97861335 | 98079991 |
| LINC00476 | chr9 | 98568369 | 98638259 |
| ERCC6L2 | chr9 | 98638424 | 98731122 |
| SLC35D2 | chr9 | 99075725 | 99145992 |
| ZNF367 | chr9 | 99148224 | 99180669 |
| TSTD2 | chr9 | 100362361 | 100395962 |
| XPA | chr9 | 100437190 | 100459691 |
| C9orf156 | chr9 | 100666771 | 100684852 |
| NANS | chr9 | 100840474 | 100844448 |
| ALG2 | chr9 | 101978706 | 101984246 |
| ERP44 | chr9 | 102741462 | 102861330 |
| INVS | chr9 | 102861501 | 103063426 |
| TEX10 | chr9 | 103064356 | 103115259 |
| MSANTD3 | chr9 | 103204187 | 103214016 |
| MSANTD3-TMEFF1 | chr9 | 103204187 | 103339918 |
| MRPL50 | chr9 | 104151121 | 104160919 |
| ZNF189 | chr9 | 104168408 | 104172942 |
| RNF20 | chr9 | 104296132 | 104325626 |
| SMC2 | chr9 | 106856830 | 106903700 |
| SLC44A1 | chr9 | 108006928 | 108153682 |
| FSD1L | chr9 | 108210314 | 108311385 |
| IKBKAP | chr9 | 111629799 | 111696608 |
| FAM206A | chr9 | 111696672 | 111703237 |
| CTNNAL1 | chr9 | 111704850 | 111775764 |
| TMEM245 | chr9 | 111777414 | 111882225 |
| KIAA0368 | chr9 | 114122972 | 114247025 |
| PTGR1 | chr9 | 114332018 | 114362135 |
| DNAJC25 | chr9 | 114393631 | 114416631 |
| DNAJC25-GNG10 | chr9 | 114393631 | 114432526 |
| PTBP3 | chr9 | 114979994 | 115095306 |
| HSDL2 | chr9 | 115142188 | 115234685 |
| FKBP15 | chr9 | 115927799 | 115983676 |
| SLC31A1 | chr9 | 115983807 | 116026772 |
| CDC26 | chr9 | 116029289 | 116037869 |
| PRPF4 | chr9 | 116037913 | 116055185 |
| POLE3 | chr9 | 116169517 | 116172955 |
| C9orf43 | chr9 | 116173021 | 116191879 |
| RGS3 | chr9 | 116343915 | 116360023 |
| COL27A1 | chr9 | 116918230 | 117072975 |
| TNFSF15 | chr9 | 117546914 | 117568408 |
| CDK5RAP2 | chr9 | 123151146 | 123342448 |
| FBXW2 | chr9 | 123519253 | 123555669 |
| LOC100288842 | chr9 | 123555774 | 123561009 |
| PSMD5 | chr9 | 123578331 | 123605299 |
| LOC253039 | chr9 | 123605319 | 123616651 |
| PHF19 | chr9 | 123617930 | 123639606 |
| GSN | chr9 | 124048857 | 124095120 |
| STOM | chr9 | 124101265 | 124132582 |
| DAB2IP | chr9 | 124530710 | 124547809 |
| RBM18 | chr9 | 125001833 | 125027143 |
| MRRF | chr9 | 125026881 | 125085742 |
| PDCL | chr9 | 125580375 | 125590935 |
| ZBTB6 | chr9 | 125670334 | 125675607 |
| ZBTB26 | chr9 | 125680377 | 125693544 |
| RABGAP1 | chr9 | 125703287 | 125867147 |
| NEK6 | chr9 | 127054249 | 127114719 |
| PSMB7 | chr9 | 127115743 | 127177752 |
| GOLGA1 | chr9 | 127640572 | 127703386 |
| PPP6C | chr9 | 127908851 | 127952218 |
| MAPKAP1 | chr9 | 128199672 | 128469513 |
| PBX3 | chr9 | 128509616 | 128729655 |
| ZBTB43 | chr9 | 129567284 | 129600487 |
| ZBTB34 | chr9 | 129622943 | 129648156 |
| ZNF79 | chr9 | 130186652 | 130207651 |
| SNORA65 | chr9 | 130210780 | 130210916 |
| LRSAM1 | chr9 | 130213764 | 130265780 |
| FAM129B | chr9 | 130267617 | 130341268 |
| SH2D3C | chr9 | 130500595 | 130524697 |
| FPGS | chr9 | 130565153 | 130576556 |
| DPM2 | chr9 | 130697373 | 130700763 |
| NAIF1 | chr9 | 130823511 | 130829599 |
| SLC25A25 | chr9 | 130853742 | 130871537 |
| GOLGA2 | chr9 | 131018107 | 131038268 |
| SWI5 | chr9 | 131038424 | 131048813 |
| TRUB2 | chr9 | 131071395 | 131084697 |
| COQ4 | chr9 | 131084790 | 131096351 |
| URM1 | chr9 | 131133597 | 131154295 |
| WDR34 | chr9 | 131395939 | 131419129 |
| SET | chr9 | 131445933 | 131458675 |
| CCBL1 | chr9 | 131595391 | 131644354 |
| DOLK | chr9 | 131707808 | 131710012 |
| NUP188 | chr9 | 131709971 | 131769375 |
| PPP2R4 | chr9 | 131873592 | 131911225 |
| IER5L | chr9 | 131937830 | 131940540 |
| LOC100506190 | chr9 | 132250938 | 132275965 |
| ASB6 | chr9 | 132396882 | 132403805 |
| GPR107 | chr9 | 132815984 | 132902448 |
| NCS1 | chr9 | 132934856 | 132999583 |
| LOC100272217 | chr9 | 133452736 | 133454881 |
| FUBP3 | chr9 | 133454959 | 133513739 |
| EXOSC2 | chr9 | 133569157 | 133580452 |
| ABL1 | chr9 | 133589267 | 133763062 |
| NUP214 | chr9 | 134000980 | 134109091 |
| FAM78A | chr9 | 134133464 | 134151906 |
| PRRC2B | chr9 | 134305476 | 134375575 |
| RAPGEF1 | chr9 | 134452156 | 134585229 |
| MED27 | chr9 | 134813314 | 134955274 |
| SETX | chr9 | 135136826 | 135230372 |
| TTF1 | chr9 | 135250936 | 135282238 |
| DDX31 | chr9 | 135469675 | 135545788 |
| TSC1 | chr9 | 135766734 | 135820020 |
| GTF3C5 | chr9 | 135906061 | 135933890 |
| SURF6 | chr9 | 136197551 | 136203047 |
| MED22 | chr9 | 136207754 | 136214972 |
| RPL7A | chr9 | 136215068 | 136218280 |
| SNORD24 | chr9 | 136216250 | 136216325 |
| SNORD36B | chr9 | 136216948 | 136217019 |
| SNORD36A | chr9 | 136217310 | 136217382 |
| SNORD36C | chr9 | 136217700 | 136217767 |
| C9orf96 | chr9 | 136243283 | 136271220 |
| REXO4 | chr9 | 136271185 | 136283164 |
| ADAMTS13 | chr9 | 136279458 | 136324508 |
| SLC2A6 | chr9 | 136336215 | 136344276 |
| VAV2 | chr9 | 136627015 | 136857446 |
| BRD3 | chr9 | 136895445 | 136933141 |
| WDR5 | chr9 | 137004913 | 137025094 |
| RXRA | chr9 | 137298427 | 137332431 |
| COL5A1 | chr9 | 137533650 | 137736688 |
| MRPS2 | chr9 | 138392476 | 138396519 |
| NACC2 | chr9 | 138898382 | 138987131 |
| C9orf69 | chr9 | 139006426 | 139010277 |
| DKFZP434A062 | chr9 | 139216997 | 139221779 |
| GPSM1 | chr9 | 139221931 | 139254057 |
| SDCCAG3 | chr9 | 139296373 | 139305054 |
| PMPCA | chr9 | 139305115 | 139318213 |
| INPP5E | chr9 | 139323066 | 139334256 |
| C9orf163 | chr9 | 139377946 | 139380519 |
| EGFL7 | chr9 | 139553307 | 139567130 |
| MIR126 | chr9 | 139565053 | 139565138 |
| AGPAT2 | chr9 | 139567594 | 139581911 |
| FAM69B | chr9 | 139615336 | 139619170 |
| SNORA43 | chr9 | 139620555 | 139620689 |
| SNORA17 | chr9 | 139621198 | 139621331 |
| KIAA1984-AS1 | chr9 | 139698378 | 139703300 |
| RABL6 | chr9 | 139729622 | 139734664 |
| EDF1 | chr9 | 139756570 | 139760738 |
| TRAF2 | chr9 | 139776384 | 139821067 |
| MIR4479 | chr9 | 139781184 | 139781255 |
| FBXW5 | chr9 | 139834884 | 139839206 |
| NPDC1 | chr9 | 139933908 | 139938544 |
| ANAPC2 | chr9 | 140069235 | 140083057 |
| SSNA1 | chr9 | 140083053 | 140084822 |
| TMEM203 | chr9 | 140098534 | 140100090 |
| NDOR1 | chr9 | 140100118 | 140113813 |
| TUBB4B | chr9 | 140135710 | 140138158 |
| NELFB | chr9 | 140149758 | 140168000 |
| TOR4A | chr9 | 140172279 | 140177093 |
| NRARP | chr9 | 140194082 | 140196703 |
| WDR85 | chr9 | 140449360 | 140473387 |
| C9orf37 | chr9 | 140509783 | 140513308 |
| EHMT1 | chr9 | 140513443 | 140730578 |
| SLC25A6 | chrX | 1505044 | 1511039 |
| CD99 | chrX | 2609227 | 2659350 |
| DDX3X | chrX | 41192650 | 41209524 |
| CDK16 | chrX | 47077527 | 47089394 |
| KDM5C | chrX | 53220502 | 53254604 |
| MSN | chrX | 64887510 | 64961793 |
| OGT | chrX | 70752911 | 70795747 |
| PGK1 | chrX | 77361858 | 77382324 |
| SRPX2 | chrX | 99899162 | 99926296 |
| HCFC1 | chrX | 153213007 | 153236819 |
| AKAP17A | chrX | 1710485 | 1721411 |
| ASMT | chrX | 1714276 | 1761974 |
| DHRSX | chrX | 2137554 | 2419015 |
| ZBED1 | chrX | 2404454 | 2418580 |
| CD99P1 | chrX | 2527305 | 2575270 |
| LINC00102 | chrX | 2531031 | 2533388 |
| WWC3 | chrX | 9983794 | 10112518 |
| CLCN4 | chrX | 10124984 | 10205699 |
| MID1 | chrX | 10413349 | 10544957 |
| HCCS | chrX | 11129405 | 11141204 |
| MSL3 | chrX | 11776708 | 11793872 |
| TMSB4X | chrX | 12993225 | 12995346 |
| RAB9A | chrX | 13707239 | 13727944 |
| PIR-FIGF | chrX | 15363712 | 15509432 |
| PIR | chrX | 15402923 | 15511711 |
| BMX | chrX | 15482368 | 15574652 |
| CA5BP1 | chrX | 15693038 | 15721474 |
| SYAP1 | chrX | 16737706 | 16780807 |
| RBBP7 | chrX | 16862774 | 16888534 |
| EIF1AX | chrX | 20142635 | 20159966 |
| SMS | chrX | 21958690 | 22012955 |
| PRDX4 | chrX | 23685644 | 23704514 |
| ACOT9 | chrX | 23721776 | 23761407 |
| SAT1 | chrX | 23801274 | 23804327 |
| EIF2S3 | chrX | 24073064 | 24096927 |
| ZFX-AS1 | chrX | 24164341 | 24167771 |
| ZFX | chrX | 24167761 | 24234372 |
| TMEM47 | chrX | 34645180 | 34675405 |
| PRRG1 | chrX | 37208582 | 37316548 |
| RPGR | chrX | 38143701 | 38186788 |
| MID1IP1 | chrX | 38663072 | 38665783 |
| CXorf38 | chrX | 40486172 | 40506819 |
| MED14 | chrX | 40508794 | 40594804 |
| USP9X | chrX | 40944887 | 41095832 |
| KDM6A | chrX | 44732422 | 44971845 |
| ZNF674 | chrX | 46357159 | 46404892 |
| ZNF674-AS1 | chrX | 46404924 | 46407910 |
| RP2 | chrX | 46696346 | 46741791 |
| NDUFB11 | chrX | 47001614 | 47004609 |
| RBM10 | chrX | 47004616 | 47046214 |
| UBA1 | chrX | 47053200 | 47074527 |
| CDK16 | chrX | 47082416 | 47089394 |
| ZNF41 | chrX | 47305560 | 47342345 |
| FTSJ1 | chrX | 48334548 | 48344752 |
| RBM3 | chrX | 48432740 | 48439553 |
| TIMM17B | chrX | 48750729 | 48755426 |
| PQBP1 | chrX | 48755194 | 48760422 |
| OTUD5 | chrX | 48779302 | 48814893 |
| TFE3 | chrX | 48886241 | 48900990 |
| SHROOM4 | chrX | 50334642 | 50557044 |
| MAGED1 | chrX | 51636697 | 51645450 |
| GPR173 | chrX | 53078505 | 53109796 |
| SMC1A | chrX | 53401069 | 53449618 |
| HUWE1 | chrX | 53559056 | 53713697 |
| FAM120C | chrX | 54094756 | 54209714 |
| TSR2 | chrX | 54466852 | 54471731 |
| GNL3L | chrX | 54556643 | 54593720 |
| APEX2 | chrX | 55026755 | 55034306 |
| UBQLN2 | chrX | 56590025 | 56593443 |
| ZXDB | chrX | 57618268 | 57623910 |
| ZXDA | chrX | 57931863 | 57937067 |
| LAS1L | chrX | 64732461 | 64754686 |
| YIPF6 | chrX | 67718623 | 67757127 |
| KIF4A | chrX | 69509932 | 69640774 |
| NONO | chrX | 70503041 | 70521018 |
| TAF1 | chrX | 70586113 | 70685855 |
| RPS4X | chrX | 71494331 | 71497141 |
| JPX | chrX | 73164158 | 73290217 |
| FTX | chrX | 73247970 | 73513409 |
| MIR545 | chrX | 73506938 | 73507044 |
| MIR374A | chrX | 73507120 | 73507192 |
| RLIM | chrX | 73802810 | 73834461 |
| PBDC1 | chrX | 75392770 | 75398033 |
| ATRX | chrX | 76760355 | 77041719 |
| MAGT1 | chrX | 77081861 | 77151065 |
| COX7B | chrX | 77154960 | 77160881 |
| SH3BGRL | chrX | 80457302 | 80554046 |
| CHM | chrX | 85116184 | 85302566 |
| KLHL4 | chrX | 86772714 | 86925050 |
| DIAPH2 | chrX | 95939661 | 96855597 |
| TIMM8A | chrX | 100600643 | 100603957 |
| RPL36A-HNRNPH2 | chrX | 100645877 | 100669128 |
| RPL36A | chrX | 100645877 | 100648840 |
| GLA | chrX | 100652778 | 100663001 |
| HNRNPH2 | chrX | 100663120 | 100669128 |
| ARMCX3 | chrX | 100878119 | 100882831 |
| WBP5 | chrX | 102611379 | 102613397 |
| MORF4L2 | chrX | 102930425 | 102941155 |
| MORF4L2-AS1 | chrX | 102942211 | 102947484 |
| FAM199X | chrX | 103411155 | 103440582 |
| PRPS1 | chrX | 106871653 | 106894256 |
| PSMD10 | chrX | 107327434 | 107334874 |
| ATG4A | chrX | 107334898 | 107397901 |
| ACSL4 | chrX | 108884563 | 108976621 |
| MIR3978 | chrX | 109325345 | 109325446 |
| PLS3 | chrX | 114795176 | 114885179 |
| WDR44 | chrX | 117480035 | 117583923 |
| NKRF | chrX | 118722299 | 118739846 |
| RPL39 | chrX | 118920466 | 118925622 |
| SNORA69 | chrX | 118921315 | 118921447 |
| RNF113A | chrX | 119004494 | 119005791 |
| NDUFA1 | chrX | 119005733 | 119010629 |
| ZBTB33 | chrX | 119384606 | 119392251 |
| LAMP2 | chrX | 119560002 | 119603204 |
| CUL4B | chrX | 119658445 | 119694817 |
| MCTS1 | chrX | 119737743 | 119755016 |
| THOC2 | chrX | 122734411 | 122866904 |
| XIAP | chrX | 122994016 | 123047829 |
| STAG2 | chrX | 123094474 | 123236505 |
| APLN | chrX | 128779235 | 128788933 |
| ELF4 | chrX | 129198894 | 129244475 |
| PHF6 | chrX | 133507341 | 133562822 |
| MMGT1 | chrX | 135044230 | 135056134 |
| RBMX | chrX | 135954426 | 135962939 |
| SNORD61 | chrX | 135961357 | 135961430 |
| MIR505 | chrX | 139006306 | 139006390 |
| VMA21 | chrX | 150565656 | 150577836 |
| NSDHL | chrX | 151999510 | 152037907 |
| BCAP31 | chrX | 152965946 | 152989556 |
| IDH3G | chrX | 153051220 | 153059978 |
| SSR4 | chrX | 153059903 | 153063967 |
| TMEM187 | chrX | 153237990 | 153248646 |
| IRAK1 | chrX | 153275956 | 153285342 |
| MIR718 | chrX | 153285370 | 153285440 |
| MECP2 | chrX | 153287263 | 153363188 |
| EMD | chrX | 153607596 | 153609883 |
| RPL10 | chrX | 153626570 | 153630680 |
| SNORA70 | chrX | 153628621 | 153628756 |
| GDI1 | chrX | 153665258 | 153671814 |
| FAM50A | chrX | 153672472 | 153679002 |
| G6PD | chrX | 153759605 | 153775233 |
| IKBKG | chrX | 153775561 | 153793261 |
| DKC1 | chrX | 153991030 | 154005964 |
| VBP1 | chrX | 154444700 | 154468098 |
| DDX3Y | chrY | 15016018 | 15032390 |
| RPS4Y1 | chrY | 2709526 | 2734997 |
| ZFY | chrY | 2803517 | 2850547 |
| PRKY | chrY | 7142012 | 7249588 |
| TTTY15 | chrY | 14774297 | 14804153 |
| UTY | chrY | 15360258 | 15592550 |
| EIF1AY | chrY | 22737610 | 22755040 |
